# Baseline Kidney Function, Albuminuria, and Urine Albumin-Creatinine Ratio Reduction with Finerenone, Empagliflozin, or Both: *Post Hoc* Analyses of CONFIDENCE Trial

**DOI:** 10.1681/ASN.0000000928

**Published:** 2025-11-06

**Authors:** Amy Mottl, Charlie Scott, Jennifer B. Green, Hiddo J.L. Heerspink, Johannes F.E. Mann, Janet B. McGill, Masaomi Nangaku, Julio Rosenstock, Peter Rossing, Li Li, Na Li, Muthiah Vaduganathan, Rajiv Agarwal

**Affiliations:** 1 University of North Carolina School of Medicine, Chapel Hill, North Carolina; 2 Bayer Healthcare Inc., Whippany, New Jersey; 3 Duke University School of Medicine, Durham, North Carolina; 4 University of Groningen and University Medical Center Groningen, Groningen, The Netherlands; 5 KfH Kidney Centre Munich and Friedrich Alexander University, Erlangen, Germany; 6 Division of Endocrinology, Metabolism and Lipid Research, Washington University in St. Louis, St. Louis, Missouri; 7 The University of Tokyo Graduate School of Medicine, Tokyo, Japan; 8 Velocity Clinical Research at Medical City, Dallas, Texas; 9 Steno Diabetes Center Copenhagen and University of Copenhagen, Copenhagen, Denmark; 10 Bayer AG, Berlin, Germany; 11 Bayer Healthcare, Beijing, China; 12 Brigham and Women's Hospital and Harvard Medical School, Boston, Massachusetts; 13 Indiana University School of Medicine, Indianapolis, Indiana

**Keywords:** albuminuria, CKD, clinical trial, GFR

## Abstract

**Key Points:**

- We assessed whether baseline urine albumin-creatinine ratio (UACR) and eGFR modify treatment effects on UACR reduction and safety in the CONFIDENCE trial.
- Higher baseline eGFR or UACR, age, female sex, and atherosclerotic cardiovascular disease were associated with greater albuminuria lowering at 6 months (180 days).
- Treatment effects on efficacy and safety were consistent, with no significant heterogeneity across baseline UACR or eGFR groups.

**Background:**

In the CONFIDENCE trial, simultaneous initiation of finerenone (10 or 20 mg) and empagliflozin (10 mg) was superior to either monotherapy in reducing albuminuria at 180 days in participants with type 2 diabetes and CKD. In this analysis, we evaluated whether baseline urine albumin-creatinine ratio (UACR) and eGFR were associated with the magnitude of UACR reduction, and whether baseline UACR and eGFR modify the effect of combination treatment.

**Methods:**

In this *post hoc* analysis of the CONFIDENCE trial (*n*=796), linear mixed-effects models were used to evaluate the effects of baseline UACR and eGFR on the change in UACR from baseline using subgroups on the basis of clinically relevant thresholds of UACR (<300 versus ≥300 mg/g) and eGFR (<60 versus ≥60 ml/min per 1.73 m^2^). Logistic regression models were used to assess the odds of achieving UACR reductions at day 180.

**Results:**

At day 180, higher baseline eGFR (per 10 ml/min per 1.73 m^2^) was independently associated with a −7% (95% confidence interval [CI], −11 to −4) greater reduction in UACR (*P* < 0.001), and higher baseline UACR (per log change of UACR) was independently associated with a −9% (95% CI, −14 to −3) greater reduction in UACR (*P* < 0.001). However, the treatment effect of finerenone, empagliflozin, or their combination on changes in UACR levels was not modified by baseline levels of eGFR or UACR. Besides the use of combination therapy, independent predictors of >30% reduction in UACR were age (odds ratio [OR], 1.23 per 10 years [95% CI, 1.05 to 1.45]), female sex (OR, 1.95 [95% CI, 1.32 to 2.86]), and atherosclerotic cardiovascular disease (OR, 1.63 [95% CI, 1.13 to 2.35]). The safety end points showed no significant heterogeneity across UACR or eGFR.

**Conclusions:**

Overall, greater albuminuria lowering was seen with a higher baseline eGFR or UACR, older age, in female patients, and those with atherosclerotic cardiovascular disease, irrespective of the treatment. Albuminuria-lowering with combination therapy, finerenone, or empagliflozin treatment effects were independent of baseline eGFR or UACR.

**Clinical Trial registry name and registration number:**

ClinicalTrials.gov, NCT05254002; European Union Drug Regulating Authorities Clinical Trials Database 2021-003037-11.

## Visual Abstract

**Figure:**
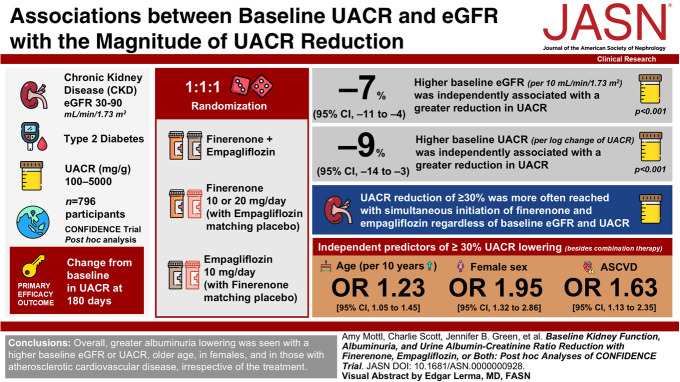

## Introduction

CKD affects approximately 40% of adults with diabetes in the United States and carries significant risks of cardiovascular morbidity, kidney failure, and mortality.^1^ The last 10 years have seen a watershed with several novel therapies approved for the treatment of CKD in type 2 diabetes, including a glucagon-like peptide-1 receptor agonist,^2^ sodium-glucose cotransporter 2 inhibitors (SGLT2i), and the nonsteroidal mineralocorticoid antagonist finerenone.^3–6^ Benefits of these drug classes include reductions in the risk for meaningful eGFR decline, kidney failure, kidney death, hospitalization for heart failure, and cardiovascular death. Findings from the CONFIDENCE trial demonstrated that simultaneous initiation of finerenone and the SGLT2i empagliflozin was not only superior compared with either monotherapy in the magnitude of albuminuria reduction at 180 days but also that it was well-tolerated, with low rates of clinically significant hyperkalemia and hypotension.^7^ This salient finding brings goal-directed medical therapy, a strategy implemented years ago by the cardiology community, to the care of people with type 2 diabetes and CKD.

Albuminuria and eGFR are independent and additive risk factors for progression of CKD and mortality and may provide insight into potential treatment response.^8,9^ In a participant-level meta-analysis of populations with diabetes, compared with individuals with urine albumin-creatinine ratio (UACR) <30 mg/g, the hazard ratio for kidney failure in those with UACR 30–299, 300–999, and ≥1000 mg/g was 1.6, 3.6, and 6.8, respectively.^9^ Similarly, this risk also rises with lower eGFR; compared with eGFR 45–74 ml/min per 1.73 m^2^, the hazard ratio for those with eGFR 30–44 and 15–30 ml/min per 1.73 m^2^ was 1.9 and 5.2, respectively. SGLT2i and finerenone have generally shown similar relative risk reductions in pivotal trials for major adverse kidney events according to albuminuria and eGFR categories,^10,11^ yet larger absolute risk reductions because of higher event rates in people with more severe kidney markers.^10^

We sought to determine whether baseline UACR and/or eGFR affect the magnitude of albuminuria reduction and whether albuminuria-lowering with finerenone, empagliflozin, or the combination is modified by UACR and/or eGFR using data from the CONFIDENCE trial. Furthermore, we assessed whether baseline UACR and eGFR alter the risk for adverse events and whether this safety profile varies across the three trial arms of the CONFIDENCE trial.

## Methods

### Trial Design and Participants

The study design and baseline characteristics of the CONFIDENCE trial have been previously published.^12,13^ In brief, the CONFIDENCE trial was a randomized, three-arm, double-dummy, double-blinded clinical trial that enrolled 818 participants from 14 countries. Randomization was 1:1:1 to finerenone (10 or 20 mg once daily with a starting dose based on eGFR and titrated according to potassium levels) with placebo matched to empagliflozin, empagliflozin 10 mg once daily with placebo matched to finerenone, or combination therapy. Inclusion criteria included age 18 years or older, eGFR 30–90 ml/min per 1.73 m^2^, type 2 diabetes, glycated hemoglobin (HbA1c) <11%, UACR 100 to <5000 mg/g on first morning void, and treatment with maximally tolerated angiotensin-converting enzyme inhibitor or angiotensin receptor blocker. Exclusion criteria included BP >160/100 mm Hg or systolic BP <90 mm Hg, type 1 diabetes, serum potassium >4.8 mmol/L, chronic heart failure with reduced ejection fraction, or cardiovascular events in the prior 90 days. All participants provided written informed consent, and the study was approved by local institutional review boards or ethics committees.

### Procedures and End Points

First morning urine void samples were collected and UACR measured at a central laboratory along with serum creatinine and potassium levels at every visit, which occurred at baseline and 14, 30, 90, and 180 days. eGFR was calculated using the CKD Epidemiology Collaboration 2009 creatinine-based equation with modification for Japanese participants; the CKD Epidemiology Collaboration 2009 creatinine-based equation was prespecified in the study protocol because the trial was initiated before widespread use of the 2021 equation.^14,15^ Standardized office BPs (after 5 minutes of rest) were averaged across three measurements. The primary end points were the relative change in UACR from baseline to 180 days compared between the combination treatment arm and each of the two monotherapy arms. Secondary end points included UACR reduction of >30%, >40%, or >50% at 180 days, and safety end points included investigator-reported hyperkalemia (hyperkalemia defined by central laboratory measurement of potassium >5.5 or >6.0 mmol/L), acute drop in eGFR >30% at day 30, and symptomatic hypotension.

### Statistical Analyses

#### Datasets

Efficacy and safety analyses were conducted using the safety analysis set, which included all randomized participants who took at least one dose of study drug, excluding those who experienced significant violations of Good Clinical Practice guidelines or were misrandomized (*n*=798; Supplemental Figure 1). An additional criterion was applied for the evaluable cohort; participants who had missing baseline and screening UACR and eGFR data were excluded (*n*=796; safety analysis set excluding two participants who had missing measurements; Supplemental Figure 1). Treatment-emergent adverse events were reported for participants who had received at least one dose of a trial drug and had an adverse event that had started or worsened after the first dose and up to 3 days after any temporary or permanent interruption of the trial treatment.

#### Categorical Baseline UACR/eGFR and Logistic Regression Analyses

Analysis of UACR reduction at day 180 by subgroups of baseline UACR (<850 and ≥850 mg/g) and baseline eGFR (<60 and ≥60 ml/min per 1.73 m^2^) individually were prespecified. For the purposes of this *post hoc* analysis, we stratified the study sample into four subgroups according to baseline eGFR and UACR. The defined subgroups were based on clinically relevant thresholds of UACR and eGFR in line with the Kidney Disease Improving Global Outcomes guidelines,^16^ as follows: (*1*) UACR <300 mg/g and eGFR <60 ml/min per 1.73 m^2^; (*2*) UACR <300 mg/g and eGFR ≥60 ml/min per 1.73 m^2^; (*3*) UACR ≥300 mg/g and eGFR <60 ml/min per 1.73 m^2^; and (*4*) UACR ≥300 mg/g and eGFR ≥60 ml/min per 1.73 m^2^. In cases where baseline measures of UACR and eGFR were unavailable, screening values were used to assign participants to the appropriate subgroup. Baseline demographics and characteristics—including vital signs, laboratory measures, medical history, and concomitant medications—were summarized using descriptive statistics, reporting means, and standard deviations for continuous variables and counts and percentages for categorical variables.

Logistic regression models were employed to assess the odds of achieving these reductions across treatment groups. The outcome variable was UACR reduction of >30%, >40%, or >50% at day 180. The independent variables were treatment, UACR/eGFR category at baseline, age, sex, region, body mass index, history of atherosclerotic cardiovascular disease, and HbA1c. In addition, the analyses were adjusted for the four eGFR and UACR categories as described above.

Treatment-emergent adverse events were summarized descriptively within each subgroup. Treatment-emergent adverse events were defined as adverse events occurring within 3 days of the last intake of study medication. Adverse events, including hyperkalemia, hypotension, and AKI, were also summarized descriptively. Statistical analyses were conducted using SAS version 9.4, with results independently validated using Stata version 19.5 by the study steering committee chair.

#### Linear Regression Analyses

To evaluate the effects of baseline UACR and eGFR on change in UACR, we used a linear mixed-effects model for repeated measures, with a missing-at-random assumption to handle missing data. Time was modeled as a categorical variable (visit) in the linear mixed-effects models to account for nonlinear changes in the outcome over time, and an unstructured covariance matrix was used to model within-participant variability between repeated measures. The random components of the model were participants and visits (representing time, modeled as a categorical variable). The dependent variable was the log-transformed ratio of the UACR at a given visit to baseline. Models were progressively adjusted for fixed effects (Supplemental Table 1). Covariates for multivariable adjustment were selected *a priori* (either on the basis of the original trial protocol or on the basis of established literature) because of their known clinical relevance as strong predictors of albuminuria progression or potential confounders in this patient population, drawing from prior landmark trials.

The nested models were tested using a likelihood ratio test.

To visualize the relationships of baseline UACR and eGFR with change in UACR, we devised four plots representing four theoretical participants with different combinations of baseline UACR and eGFR in the third and fourth quartiles of the trial population and mean values (marginal means) for continuous variables and referent values for categorical variables. The marginal means and their 95% confidence intervals (CIs) were estimated after fitting the linear mixed model 5 for estimating the effect of eGFR change and UACR change on percent UACR reduction while adjusting for the baseline covariates. To facilitate clinical interpretation of percent UACR reductions by treatment over time, the marginal means were plotted as observed at 100, 300, 1000, and 3000 mg/g for UACR and 30, 45, 60, and 75 ml/min per 1.73 m^2^ for eGFR. These values are similar to the first and third quartiles of UACR and eGFR found in the study.

Scatter plots were generated to visualize the associations between baseline eGFR, baseline UACR, and corresponding UACR change at 6 months. Three plots were produced for each treatment group, with each point representing an individual patient. The color of the points corresponded to the UACR change at day 180, ranging from −100% to 100%.

## Results

### Study Population Characteristics

The proportion of missing data for baseline eGFR and UACR was minimal. Of the 798 participants in the safety analysis set, two had missing baseline eGFR and UACR measurements (one in the empagliflozin group and one in the combination therapy group). Therefore, a total of 796 participants (264 in the finerenone group, 265 in the empagliflozin group, and 267 in the combination therapy group) with baseline values of eGFR and UACR and who received at least one dose of the study drug were included in the current *post hoc* analyses (Supplemental Figure 1 and Supplemental Table 2). Baseline characteristics of the CONFIDENCE trial participants, stratified by baseline eGFR/UACR category, are displayed in Table 1. Most participants were in the group with eGFR <60 ml/min per 1.73 m^2^ and UACR ≥300 mg/g (*N*=400; 50%), followed by the group with eGFR ≥60 ml/min per 1.73 m^2^ and UACR ≥300 mg/g (*N*=186; 23%). Body mass index, systolic BP, HbA1c, and prevalence of atherosclerotic cardiovascular disease were similar across groups, as was uniform treatment with angiotensin-converting enzyme inhibitor/angiotensin receptor blocker required by the study inclusion criteria.

**Table 1:** Baseline characteristics of 796 participants in the CONFIDENCE trial stratified by baseline urine albumin-creatinine ratio and eGFR subgroups

Clinical Characteristic	eGFR ≥60 ml/min per 1.73 m^2^		eGFR <60 ml/min per 1.73 m^2^		Total
	UACR, mg/g		UACR, mg/g		
	<300	≥300	<300	≥300	
Sample size, *n* (%)	89 (11)	186 (23)	121 (15)	400 (50)	796 (100)
**Treatment group, *n* (%)**					
Combination	33 (37)	60 (32)	40 (33)	134 (34)	267 (34)
Finerenone	24 (27)	72 (39)	46 (38)	122 (30)	264 (33)
Empagliflozin	32 (36)	54 (29)	35 (29)	144 (36)	265 (33)
Age, yr, mean±SD	66±10	64±11	68±10	67±10	66±10
Male sex, *n* (%)	70 (79)	137 (74)	81 (67)	311 (78)	599 (75)
**Geographic region, *n* (%)**					
Asia	22 (25)	89 (48)	57 (47)	191 (48)	359 (45)
Europe	28 (31)	57 (31)	20 (17)	108 (27)	213 (27)
North America	39 (44)	40 (22)	44 (36)	101 (25)	224 (28)
Body mass index, kg/m^2^, mean±SD	30.4±6.7	29.7±6.3	28.5±5.6	29.1±5.9	29.3±6.1
Serum potassium value, mmol/L, mean±SD	4.4±0.4	4.4±0.4	4.6±0.4	4.5±0.4	4.5±0.4
Systolic BP, mm Hg, mean±SD	134±13	138±13	132±12	135±14	135±13
History of atherosclerotic cardiovascular disease, *n* (%)	22 (25)	44 (24)	32 (26)	125 (32)	223 (29)
eGFR values, ml/min per 1.73 m^2^, mean±SD	73±11	74±11	43±9	44±9	54±17
**KDIGO CKD stage, *n* (%)**					
G1 or G2	89 (100)	186 (100)			275 (35)
G3A			47 (39)	187 (47)	234 (29)
G3B			67 (55)	194 (48)	261 (33)
G4			7 (6)	19 (5)	26 (3)
UACR, mg/g, median (25–75 percentiles)	194 (124–256)	725 (442–1338)	177 (124–239)	787 (522–1469)	575 (291–1092)
**Severity of albuminuria, *n* (%)**					
<300 mg/g	89 (100)		121 (100)		210 (26)
300 to <1000 mg/g		121 (65)		243 (61)	364 (46)
>1000 mg/g		65 (35)		157 (39)	222 (28)
HbA1c, %, mean±SD	7.0±1.1	7.5±1.3	7.2±1.2	7.3±1.2	7.3±1.2
**Concomitant medications, *n* (%)**					
ACEi or ARB	88 (99)	184 (99)	117 (97)	394 (98)	783 (98)
Statins	78 (88)	142 (76)	91 (75)	284 (71)	595 (75)
Diuretics	31 (35)	56 (30)	41 (34)	160 (40)	288 (36)
Insulin	36 (40)	83 (45)	44 (36)	153 (38)	316 (40)
GLP-1 RA	20 (22)	50 (27)	29 (24)	81 (20)	180 (23)

ACEi, angiotensin-converting enzyme inhibitor; ARB, angiotensin receptor blocker; GLP-1 RA, glucagon-like peptide-1 receptor agonist; HbA1c, glycated hemoglobin; KDIGO, Kidney Disease Improving Global Outcomes; UACR, urine albumin-creatinine ratio.

### Efficacy

The proportion of observations included in the analyses at each visit is shown in Supplemental Table 2.

#### Logistic Regression Models

In the adjusted logistic regression model, combination therapy was associated with higher odds of achieving a >30% reduction in UACR from baseline to day 180 compared with monotherapy with finerenone (Table 2; odds ratio [OR], 2.14 [95% CI, 1.45 to 3.17]) or empagliflozin (OR, 2.21 [95% CI, 1.50 to 3.27]). The results were similar for >40% and >50% reductions in UACR. UACR/eGFR categories were not significantly associated with either >30%, >40%, or >50% UACR reduction. The odds of achieving a >30%, >40%, and >50% reduction in UACR were significantly higher with female sex (1.95 [95% CI, 1.32 to 2.86], 1.80 [95% CI, 1.24 to 2.61], and 1.77 [95% CI, 1.22 to 2.57], respectively) and history of atherosclerotic cardiovascular disease (1.63 [95% CI, 1.13 to 2.33], 1.50 [95% CI, 1.05 to 2.14], and 1.86 [95% CI, 1.30 to 2.67], respectively). The odds of achieving a >30% reduction in UACR were significantly greater with older age per 10 years (1.23 [95% CI, 1.05 to 1.45]). Body mass index and HbA1c were not associated with the odds of achieving a >30%, >40%, or >50% reduction in UACR (Table 2).

**Table 2:** Odds of >30%, >40%, and >50% urine albumin-creatinine ratio reduction from baseline to day 180 by baseline urine albumin-creatinine ratio and eGFR subgroups in 796 participants of the CONFIDENCE trial

Participant Subgroups from the CONFIDENCE Trial	>30% Reduction in UACR		>40% Reduction in UACR		>50% Reduction in UACR	
	*n*/*N*	OR (95% CI)	*n*/*N*	OR (95% CI)	*n*/*N*	OR (95% CI)
**Treatment group**						
Combination versus empagliflozin	163/233 versus 122/235	2.21 (1.50 to 3.27)	151/233 versus 103/235	2.40 (1.64 to 3.51)	128/233 versus 76/235	2.63 (1.79 to 3.87)
Combination versus finerenone	163/233 versus 119/231	2.14 (1.45 to 3.17)	151/233 versus 102/231	2.28 (1.56 to 3.35)	128/233 versus 82/231	2.18 (1.48 to 3.20)
**UACR eGFR combinations**						
eGFR ≥60 ml/min per 1.73 m^2^ and UACR <300 mg/g	43/78	Reference	40/78		32/78	
eGFR ≥60 ml/min per 1.73 m^2^ and UACR ≥300 mg/g	96/166	1.11 (0.62 to 1.98)	88/166	1.09 (0.62 to 1.94)	71/166	1.12 (0.62 to 2.01)
eGFR <60 ml/min per 1.73 m^2^ and UACR <300 mg/g	54/105	0.78 (0.42 to 1.45)	47/105	0.73 (0.39 to 1.36)	37/105	0.77 (0.41 to 1.47)
eGFR <60 ml/min per 1.73 m^2^ and UACR ≥300 mg/g	211/350	1.15 (0.68 to 1.95)	181/350	0.99 (0.59 to 1.66)	146/350	1.02 (0.60 to 1.74)
Age—per 10 yr		1.23 (1.05 to 1.45)		1.15 (0.98 to 1.36)		1.12 (0.95 to 1.33)
Male sex	290/530	Reference	254/530		203/530	
Female sex	114/169	1.95 (1.32 to 2.86)	102/169	1.80 (1.24 to 2.61)	83/169	1.77 (1.22 to 2.57)
**Geographic region**						
Asia	182/324	1.08 (0.70 to 1.68)	156/324	0.98 (0.64 to 1.51)	119/324	0.99 (0.63 to 1.54)
Europe	115/190	1.07 (0.69 to 1.65)	103/190	1.05 (0.68 to 1.62)	92/190	1.34 (0.86 to 2.07)
North America	107/185	Reference	97/185		75/185	
Body mass index, kg/cm^2^—per unit change		0.99 (0.96 to 1.02)		1.00 (0.97 to 1.03)		1.00 (0.97 to 1.03)
No history of atherosclerotic cardiovascular disease	276/507	Reference	244/507		186/321	
History of atherosclerotic cardiovascular disease	128/192	1.63 (1.13 to 2.35)	112/192	1.50 (1.05 to 2.14)	100/192	1.86 (1.30 to 2.67)
HbA1c, %—per unit change		1.06 (0.93 to 1.21)		1.05 (0.92 to 1.19)		1.00 (0.88 to 1.14)

Odds ratios were derived from multivariable adjusted logistic regression models. Covariates were all the factors indicated in the table above: treatment, UACR/eGFR category at baseline, age, sex, region, body mass index, history of atherosclerotic cardiovascular disease, and HbA1c. CI, confidence interval; HbA1c, glycated hemoglobin; OR, odds ratio; UACR, urine albumin-creatinine ratio.

#### Linear Mixed Models

Model coefficients and their 95% CIs of the progressively adjusted linear mixed models to assess the change from baseline in UACR over visits are shown in Supplemental Table 3. The statistical significance of the parameters, the models, and the comparison of the nested models is shown in Supplemental Table 4. We formally tested for higher-order interactions (*e.g*., three-way interactions between treatment, baseline eGFR, and baseline UACR); however, these terms were not statistically significant (*P* > 0.05) and showed evidence of model instability (*i.e*., wide CIs), consistent with overparameterization. Therefore, the more parsimonious and stable models are presented as the primary analysis. The unadjusted treatment model (model 1) showed significant effects of treatment, treatment by visit, and overall treatment effect. Model 1, adjusted for baseline log UACR, improved model fit and showed a significant effect of log UACR on the outcome variable (model 2). Model 1, adjusted for baseline eGFR, improved model fit and showed a significant effect of baseline eGFR on the outcome variable (model 3). Model 1, adjusted both for log UACR and baseline eGFR, showed a significant effect of both these factors on the model fit (model 4). A likelihood ratio test demonstrated that model 4 was significantly superior to model 2 (chi-squared 25.7, *P* < 0.001) and to model 3 (chi-squared 20.2, *P* = 0.001). Adding covariates to model 4 further improved model fit (chi-squared 58.1, *P* = 0.008, model 5 versus model 4). Interacting the baseline eGFR and baseline log UACR with treatments did not improve model fit any further (chi-squared 23.9, *P* = 0.25, model 6 versus model 5). Model 5 showed a greater UACR reduction with age and in female participants. Model 6 shows that there was no evidence for effect modification by baseline eGFR (*P* = 0.3) or baseline log UACR (*P* = 0.2) by treatment; therefore, the model did not improve upon prediction of UACR reduction compared with model 5. Consequently, the findings based on model 5 are presented in Table 3, which shows the change in UACR at specific time points (visits) as a function of baseline eGFR and baseline UACR. In a fully adjusted model (model 5), at day 180, a 10 ml/min per 1.73 m^2^ higher baseline eGFR was associated with −7 (95% CI, −11 to −4) percentage point change in UACR at day 180 (*P* value for eGFR effect < 0.001). Furthermore, one log change in baseline UACR was associated with −9 (95% CI, −14 to −3) percentage point change in UACR (*P* value for UACR effect < 0.001). These effects were independent of treatment. Per 10-year older age, at day 180, was associated with −11 (95% CI, −16 to −5) percentage point change in UACR (*P* value for age effect < 0.01). Compared to male participants, female participants at day 180 had −21 (95% CI, −31 to −9) percentage point change in UACR (*P* value for sex effect < 0.01). The mean percentage change in UACR from baseline is shown in Supplemental Table 5.

**Table 3:** Percent change in urine albumin-creatinine ratio from baseline at various time points by baseline eGFR and baseline log urine albumin-creatinine ratio, all three treatment groups combined

Time Points	Change in UACR from Baseline per Log Baseline UACR	Change in UACR from Baseline per 10 ml/min per 1.73 m^2^ Baseline eGFR
	Percent (95% CI)	Percent (95% CI)
Day 14	−4 (−8 to −0.5)	−4 (−6 to −2)
Day 30	−3 (−6 to 1)	−5 (−7 to −3)
Day 90	−7 (−11 to −2)	−3 (−6 to −0.7)
Day 180	−9 (−14 to −3)	−7 (−11 to −4)
Day 210	−12 (−18 to −7)	−1 (−5 to 2)

Marginal means and their 95% CIs were estimated after fitting the linear mixed model 5 for estimating the effect of eGFR change and UACR change on percent UACR reduction. Covariates were treatment, visits, treatment×visit, log baseline UACR, UACR, baseline eGFR, baseline eGFR×visit, treatment×visit, age, age×visit, sex, sex×visit, region, region×visit, HbA1c, baseline HbA1c×visit, baseline history of atherosclerotic cardiovascular disease, baseline history of atherosclerotic cardiovascular disease×visit, baseline body mass index, baseline body mass index×visit. Percent changes were calculated by exponentiating the ratio, subtracting 1, and multiplying the result by 100. Data are marginal means computed from mixed model 5 (which used the covariates listed above; Supplemental Tables 2 and 3). Baseline urine albumin-creatinine ratio effect *P* < 0.001 and eGFR effect *P* < 0.001. See text for details. CI, confidence interval; HbA1c, glycated hemoglobin; UACR, urine albumin-creatinine ratio.

**Figure 1:**
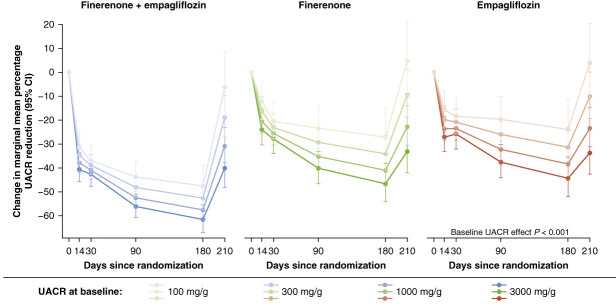
**Percent change in UACR from baseline to various time points in the CONFIDENCE trial for participants with higher levels of baseline UACR.** A fully adjusted model was fitted, and marginal means were computed for each level of baseline UACR. The UACR effect was computed by testing the joint probability of the log baseline UACR and log baseline UACR×visit interaction being different from 0 using a Wald test. CI, confidence interval; UACR, urine albumin-creatinine ratio.

Figure 1 shows the change in UACR from baseline to various time points with higher levels of baseline UACR. Regardless of the treatment, higher levels of baseline UACR was associated with a greater percent reduction in UACR (*P* < 0.001 for baseline UACR effect).

**Figure 2:**
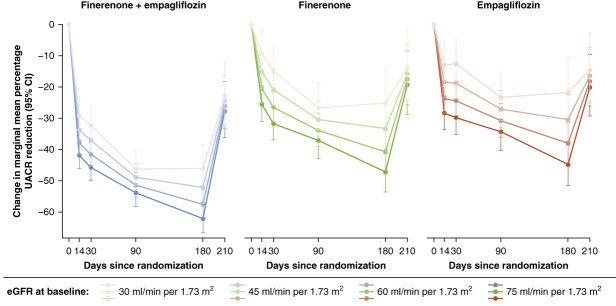
**Percent change in UACR from baseline to various time points in the CONFIDENCE trial for participants with higher levels of baseline eGFR.** A fully adjusted model was fitted, and marginal means were computed for each level of baseline eGFR. The eGFR effect was computed by testing the joint probability of the baseline eGFR and baseline eGFR×visit interaction being different from 0 using a Wald test.

Figure 2 shows the change in UACR from baseline to various time points with higher levels of baseline eGFR. Regardless of the treatment, higher levels of baseline eGFR was associated with a greater percent reduction in UACR (*P* < 0.001 for baseline eGFR effect).

**Figure 3:**
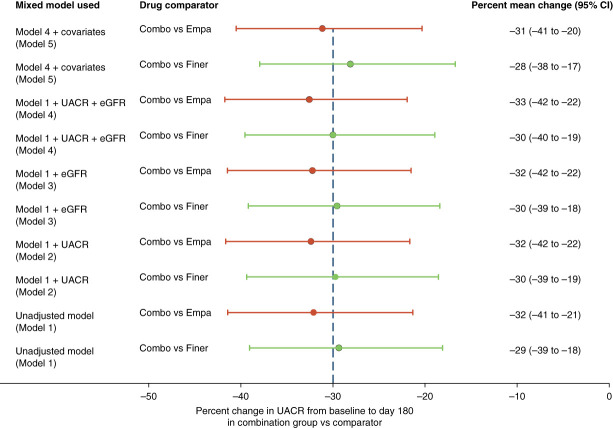
**Forest plots of mixed models for percent mean difference from baseline UACR to day 180 in the combination versus monotherapy treatment arms (finerenone and empagliflozin) in the CONFIDENCE trial.** A vertical dotted line at −30% is shown as a visual reference to facilitate comparison of treatment effects across therapies. combo, combination therapy; empa, empagliflozin; finer, finerenone.

**Figure 4:**
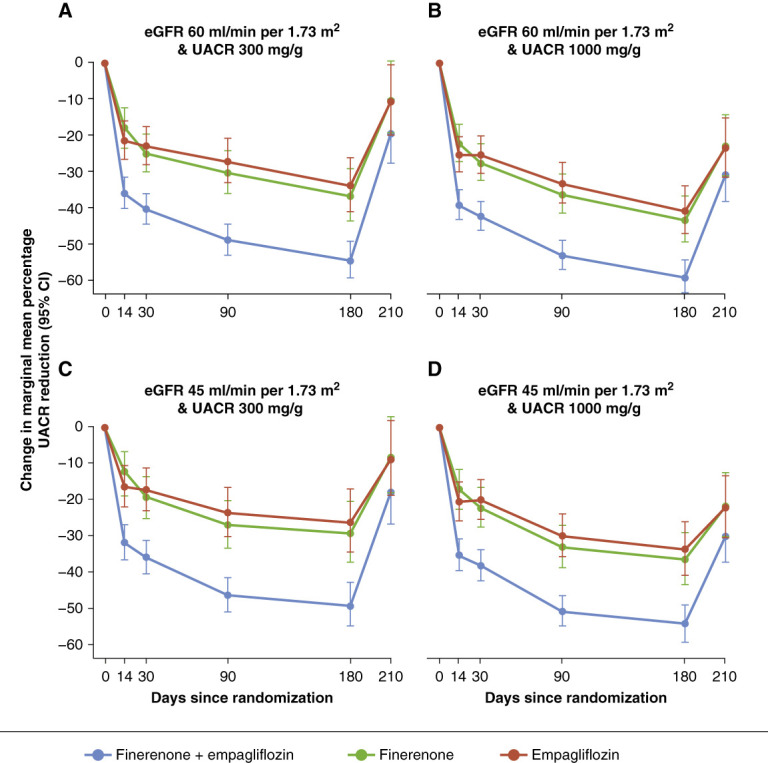
**Expected change in mean percentage UACR reduction from baseline to day 180 (95% CI) by eGFR and UACR category in the CONFIDENCE trial.** (A) eGFR 60 ml/min per 1.73 m^2^ and UACR 300 mg/g. (B) eGFR 60 ml/min per 1.73 m^2^ and UACR 1000 mg/g. (C) eGFR 45 ml/min per 1.73 m^2^ and UACR 300 mg/g. (D) eGFR 45 ml/min per 1.73 m^2^ and UACR 1000 mg/g. A linear mixed-effects model was fitted and marginal means were computed for each specified baseline UACR and eGFR category for the average participant (*i.e*., holding all other covariates constant [at their mean values]).

Figure 3 shows a consistent treatment effect of combination therapy compared with finerenone or empagliflozin alone. This effect is further visualized in Figure 4, which compares baseline UACR 1000 versus 300 mg/g and baseline eGFR 60 versus 45 ml/min per 1.73 m^2^. Those with UACR 1000 mg/g and eGFR 60 ml/min per 1.73 m^2^ have a greater percent UACR reduction compared with those with UACR 300 mg/g and eGFR of 45 ml/min per 1.73 m^2^.

Supplemental Figure 2 shows a scatter plot of eGFR and UACR, with the individual reduction in UACR shown by the color of the dot. Cooler colors indicate a greater reduction in UACR from baseline to day 180. Most of the warmer colors appear below an eGFR of 60 and UACR <300 for all three treatment groups.

#### Safety

Symptomatic hypotension was uncommon, occurring in only two individuals, both in the combination therapy group and eGFR <60 ml/min per 1.73 m^2^ stratum (Table 4). Acute eGFR drop >30% at 30 days was more common in the combination therapy and finerenone treatment groups and among those with eGFR >60 ml/min per 1.73 m^2^. Investigator-reported hyperkalemia and hyperkalemia by central measurements tended to be higher in lower eGFR strata.

**Table 4:** Hypotension, acute eGFR decline, and hyperkalemia events by baseline urine albumin-creatinine ratio and eGFR subgroups in the CONFIDENCE trial

Adverse Events and Treatment Groups	eGFR ≥60 ml/min per 1.73 m^2^		eGFR <60 ml/min per 1.73 m^2^		Total
	UACR, mg/g		UACR, mg/g		
	<300	≥300	<300	≥300	
**Symptomatic hypotension, *n*/*N* (%)**					
Combination	0	0	1/40 (3)	1/134 (0.7)	2/267 (0.7)
Finerenone	0	0	0	0	0
Empagliflozin	0	0	0	0	0
Total	0	0	1/121 (0.8)	1/400 (0.3)	2/796 (0.3)
**>30% decline in eGFR from baseline to day 30, *n*/*N* (%)**					
Combination	4/33 (12)	6/60 (10)	2/40 (5)	5/134 (4)	17/267 (6)
Finerenone	1/24 (4)	5/72 (7)	0	4/122 (3)	10/264 (4)
Empagliflozin	0	1/54 (2)	0	2/144 (1)	3/265 (1)
Total	5/89 (6)	12/186 (6)	2/121 (2)	11/400 (3)	30/796 (4)
**Hyperkalemia reported by investigator, *n*/*N* (%)**					
Combination	2/33 (6)	5/60 (8)	3/40 (8)	15/134 (11)	25/267 (9)
Finerenone	2/24 (8)	6/72 (8)	4/46 (9)	18/122 (15)	30/264 (11)
Empagliflozin	0	1/54 (2)	2/35 (6)	7/144 (5)	10/265 (4)
Total	4/89 (4)	12/186 (6)	9/121 (7)	40/400 (10)	65/796 (8)
**Serum potassium level >5.5 mmol/L, *n*/*N* (%)**					
Combination	4/33 (12)	5/59 (8)	3/40 (8)	28/132 (21)	40/264 (15)
Finerenone	3/24 (13)	8/71 (11)	10/45 (22)	27/115 (23)	48/255 (19)
Empagliflozin	1/31 (3)	6/52 (12)	3/35 (9)	15/140 (11)	25/258 (10)
Total	8/88 (9)	19/182 (10)	16/120 (13)	70/387 (18)	113/777 (15)
**Serum potassium level >6.0 mmol/L, *n*/*N* (%)**					
Combination	1/33 (3)	2/60 (3)	1/40 (3)	8/134 (6)	12/267 (4)
Finerenone	0	3/72 (4)	2/46 (4)	7/122 (6)	12/264 (5)
Empagliflozin	0	1/54 (2)	0	6/144 (4)	7/265 (3)
Total	1/89 (1)	6/186 (3)	3/121 (2)	21/400 (5)	31/796 (4)

UACR, urine albumin-creatinine ratio.

Adverse events, serious adverse events, and deaths are shown in Supplemental Table 6. These events appeared to be evenly distributed across the eGFR and UACR strata.

## Discussion

The current *post hoc* analyses demonstrate the following: baseline eGFR and the level of albuminuria independently influence the reduction in UACR at day 180, regardless of treatment, and regardless of the level of baseline albuminuria or eGFR the relative benefit of combination therapy versus monotherapy is similar. Those with higher eGFR and higher UACR were more likely to have a more pronounced reduction in albuminuria from baseline to day 180 with any treatment. Other novel findings of our study are that older age, female sex, and the presence of atherosclerotic cardiovascular disease were associated with higher odds of achieving a clinically relevant reduction in UACR. The safety differences among treatments were not influenced by baseline UACR or baseline eGFR, although this result should be interpreted in the context of the 180-day treatment duration and study cohort size.

A >30% reduction in albuminuria is a strong predictor of kidney outcomes based on evidence from observational studies and clinical trials. This threshold was more often reached with combination therapy compared with each therapy alone in CONFIDENCE.

Studies on finerenone and SGLT2i have explored how kidney outcomes are affected by baseline eGFR and UACR, and the data support treating people during early stages of CKD. Furthermore, these studies lend further support to the hypothesis that albuminuria is a strong and modifiable risk factor for kidney disease progression and cardiovascular disease.

The Efficacy and Safety of Finerenone in Subjects With Type 2 Diabetes Mellitus and Diabetic Kidney Disease (FIDELIO-DKD) (https://clinicaltrials.gov/study/NCT02540993) trial, which predominantly enrolled high-risk patients (88% with a UACR ≥300 mg/g and 88% with an eGFR <60 ml/min per 1.73 m^2^), stratified results by baseline eGFR and UACR for a composite kidney end point (sustained eGFR decline ≥40%, kidney failure, or kidney death). It found similar risk reductions afforded by finerenone across eGFR strata, but CIs were notably wide in lower-risk groups with fewer events reported in these groups. A further analysis (a prespecified pooled analysis of the FIDELIO-DKD and FIGARO-DKD studies) supported that albuminuria across all strata is also a strong and modifiable risk factor for cardiovascular disease in type 2 diabetes.^17^

In the Evaluation of the Effects of Canagliflozin on Renal and Cardiovascular Outcomes in Participants With Diabetic Nephropathy (https://clinicaltrials.gov/study/NCT02065791) trial with the SGLT2i canagliflozin, a secondary analysis stratified by eGFR showed similar effect estimates of canagliflozin for the primary composite end point (kidney failure, doubling of serum creatinine, or kidney death) across eGFR strata. Slope analyses found no difference in the magnitude of eGFR decline by baseline eGFR and no differences in the benefit of canagliflozin on blunting eGFR decline. However, when the benefit of canagliflozin on the primary kidney end point was stratified by baseline UACR (<1000, 1000–2999, and ≥3000 mg/g), the highest benefit was found in the group with a UACR ≥3000 mg/g.

The Study of Heart and Kidney Protection With Empagliflozin (https://clinicaltrials.gov/study/NCT03594110) trial with the SGLT2i empagliflozin found greater reductions in both total and chronic eGFR slope in subgroups with higher baseline eGFR when the drug was compared with placebo.^18^ The greatest effect of empagliflozin on eGFR slope was seen in the eGFR ≥45 ml/min per 1.73 m^2^ subgroup (notably, participants with an eGFR ≥45 ml/min per 1.73 m^2^ had to have a UACR ≥200 mg/g), while there was no difference compared with placebo in the eGFR <20 ml/min per 1.73 m^2^ group. The difference in eGFR slope between the empagliflozin and control groups was greatest in strata with a higher UACR, with the maximal effect observed in the UACR >1000 mg/g subgroup.

An unexpected finding in this exploratory analysis was that participants of older age experienced a greater benefit than their younger counterparts, which is of great importance given that people over the age of 70 years comprise nearly 40% of the CKD population in the United States.^19^ Polypharmacy is a major issue in geriatrics, which may lead to underutilization of CKD medications in the elderly, especially when the risk of death often exceeds that of CKD progression in people older than 75 years.^20^ Despite the high mortality in people 75–84 years with CKD, progressive kidney disease does occur in over 50% with stage 3 CKD and moderate-severe albuminuria, and kidney failure occurs in 45% with stage 4 CKD and moderate-severe albuminuria.^20^ The finding that simultaneous initiation of finerenone and SGLT2i is more beneficial in the elderly may help to limit clinical inertia in this population. A meta-analysis of the CANagliflozin cardioVascular Assessment Study (https://clinicaltrials.gov/study/NCT01032629) and Evaluation of the Effects of Canagliflozin on Renal and Cardiovascular Outcomes in Participants With Diabetic Nephropathy (https://clinicaltrials.gov/study/NCT02065791) trials did not find heterogeneity of kidney benefit with canagliflozin in older age groups, and confirmation of our finding is needed.^21^

The current exploratory analysis also found that female participants had greater albuminuria reduction with combination treatment versus monotherapy compared with male participants; this will require confirmation in future trials. Large trials of finerenone and SGLT2i have not found heterogeneity in kidney benefit by sex.^4,22^ While female participants have higher rates of CKD 3–5, male participants are more likely to have albuminuria and progression to kidney failure.^23,24^ The underlying reasons for this are not completely understood, but likely relate to sex hormones, differences in kidney hemodynamics, cardiovascular disease morbidity, healthy lifestyles, and access to health care.^24,25^ A large study of commercially insured patients with type 2 diabetes found that female patients with CKD and type 2 diabetes were less likely than male patients to be prescribed SGLT2i (adjusted OR, 0.84 [95% CI, 0.82 to 0.85]).^26^

Our finding of greater benefit in UACR reduction with combination versus monotherapy in those with a history of atherosclerotic cardiovascular disease after 180 days is counter to meta-analyses of longer trials with SGLT2i or finerenone trials where clinical benefits on hard end points are similar regardless of the presence or absence of atherosclerotic cardiovascular disease. For example, a meta-analysis of the SGLT2i outcome trials showed similar reductions in the composite kidney end point of worsening eGFR, kidney failure, or kidney death in those with and without evidence for atherosclerotic cardiovascular disease.^27^ A prespecified pooled analysis of the FIDELIO-DKD and FIGARO-DKD studies, a combined analysis of two large trials of finerenone in people with type 2 diabetes and CKD of varying severity, also did not find heterogeneity in kidney or cardiovascular benefits according to history of atherosclerotic cardiovascular disease.^28^

Results of our analyses do need to be interpreted within the limitations of the study, including the use of albuminuria reduction rather than clinical kidney end points and treatment duration of 6 months. There was no placebo group; however, the reduction in UACR can still be considered as a strong treatment effect and should not be interpreted as regression to the mean. This is because we did not see an increase in those with a UACR of <300 mg/g at baseline. Furthermore, in the large placebo-controlled finerenone trials, the change in UACR from baseline to 4 months on placebo was 3% only,^29^ an order of magnitude less than what was observed in CONFIDENCE. Baseline systolic BP is a potential prognostic factor or confounder for change in albuminuria.^30,31^ As the current analysis aimed to evaluate baseline eGFR and UACR as modifiers of the treatment's effect on UACR reduction, we chose to omit baseline BP from the model to avoid bias of our estimate of the total effect modification by baseline kidney function; however, this is a limitation of the study. The interventions (finerenone, empagliflozin, or the combination) modify systolic BP; therefore, change in systolic BP is a postbaseline variable that might be on the causal pathway between the intervention and the outcome (UACR reduction), to varying degrees across treatment arms. In this context, change in systolic BP functions as a mediator, not a confounder, and inclusion in the model would control away a significant portion of the treatment effect we are seeking to understand; therefore, we chose not to include change in systolic BP as a factor in the model. The complex, trilateral relationship between combination treatment with finerenone and empagliflozin, its hemodynamic effects (*i.e*., BP mediation), and its direct kidney effects assessed by change in UACR is a distinct and complex research question that is beyond the scope of the current analyses. Inclusion and exclusion criteria also limit the generalizability of our findings to other populations, particularly in those with low-risk CKD. Verification of our results, as well as an understanding of prescribing practices in patients with type 2 diabetes and CKD, will be investigated in A Study Called FINE-REAL to Learn More About the Use of the Drug Finerenone in a Routine Medical Care Setting (https://clinicaltrials.gov/study/NCT05348733), a prospective, observational analysis of 5500 participants with type 2 diabetes and CKD.^32^ While the overall treatment effect modification observed is robust, caution should be used when interpreting specific subgroups. CONFIDENCE was not powered to analyze clinical outcomes by UACR and eGFR subgroups. The current analyses were *post hoc* and should be considered exploratory in nature only. In addition, the multiple statistical comparisons performed increases the risk of chance findings, especially for the dichotomous outcomes; however, the findings were consistent across the different linear fixed-effect models used Furthermore, our analysis using linear mixed models assumes data are missing at random. While the overall degree of missingness was low and balanced, we did not conduct formal sensitivity analyses, such as pattern-mixture models, to stringently test this assumption. In addition, we did not formally explore potential nonlinear relationships for baseline eGFR and UACR using spline functions, although our use of categorical analyses provides some insight into this. Furthermore, the scatterplots of change from baseline in UACR to day 180, baseline UACR, and baseline eGFR did not reveal any overt nonlinearity. The baseline UACR may be mathematically correlated with the absolute change in UACR during the study. We addressed this by evaluating UACR change as a percentage reduction, which lessens this inherent mathematical coupling compared with absolute change. Furthermore, we focused our primary analysis on the treatment effect modification by baseline UACR, rather than the simple relationship between baseline UACR and UACR change. Specifically, although baseline eGFR and UACR had a modest influence on the magnitude of benefit across treatment groups, the effect of combination therapy versus monotherapy on UACR reduction was not dependent on baseline UACR and eGFR. Finally, as with any multivariable model, the potential for model overfitting or residual confounding from unmeasured variables cannot be entirely excluded, though our core findings were robust to adjustment.

In conclusion, we found that simultaneous initiation of finerenone and SGLT2i conferred greater reductions in albuminuria at 180 days compared with monotherapy, and this benefit was consistent across baseline eGFR or UACR. A higher baseline eGFR or UACR, older age, female sex, and atherosclerotic cardiovascular disease history were associated with a greater reduction in albuminuria across all treatment groups in the CONFIDENCE study. Compared with either monotherapy, the effects of combination therapy on eGFR and safety end points were consistent across baseline UACR and eGFR groups.

## Supplementary Material

## Data Availability

Availability of the data underlying this publication will be determined according to Bayer's commitment to the European Federation of Pharmaceutical Industries and Associations/Pharmaceutical Research and Manufacturers of America “Principles for responsible clinical trial data sharing.” This pertains to scope, time point, and process of data access. As such, Bayer commits to sharing upon request from qualified scientific and medical researchers, patient-level clinical trial data, study-level clinical trial data, and protocols from clinical trials in patients for medicines and indications approved in the US and European Union as necessary for conducting legitimate research. This applies to data on new medicines and indications that have been approved by US and European Union regulatory agencies on or after January 1, 2014. Interested researchers can use www.vivli.org to request access to anonymized patient-level data and supporting documents from clinical studies to conduct further research that can help advance medical science or improve patient care. Information on the Bayer criteria for listing studies and other relevant information is provided in the member section of the portal. Data access will be granted to anonymized patient-level data, protocols, and clinical study reports after approval by an independent scientific review panel. Bayer is not involved in the decisions made by the independent review panel. Bayer will take all necessary measures to ensure that patient privacy is safeguarded.

## Declarative Statements

This study includes clinical experimentation and received Institutional Review Board or Ethics Committee approval. All patients provided written informed consent. This study includes clinical experimentation and complies with consensus ethical principles derived from international guidelines including the Declaration of Helsinki.

## Supplemental Material

Supplemental Summary 1. CONFIDENCE trial investigators.

Supplemental Table 1. Fixed effects in the linear mixed-effects models.

Supplemental Table 2. Number of participants with UACR and eGFR baseline data and UACR data at each time point included in the efficacy analyses.

Supplemental Table 3. Model parameters, mean coefficients, and their 95% CIs.

Supplemental Table 4. Statistical significance of model parameters and comparison of the various models.

Supplemental Table 5. Percentage change in UACR from baseline.

Supplemental Table 6. Advanced and serious adverse events by baseline UACR and eGFR subgroups.

Supplemental Figure 1. Participant disposition.

Supplemental Figure 2. Plot of percent reduction from baseline UACR according to baseline eGFR versus UACR across the three treatment arms in the CONFIDENCE trial.

Statistical Analysis Plan
