# Supplementary material for: Baseline Kidney Function, Albuminuria, and Urine Albumin-Creatinine Ratio Reduction with Finerenone, Empagliflozin, or Both: Post Hoc Analyses of CONFIDENCE Trial
Source: J Am Soc Nephrol. 2025 Nov 6;37(4):764–76. doi: 10.1681/ASN.0000000928 (PMC13065124; doi:10.1681/ASN.0000000928)
Supplement: Supplementary file 1 [file jasn-37-764-s001.pdf]

ASN American Society of Nephrology | JASN Journal of the American Society of Nephrology | KIDNEY360 Accessing Our World From Every Angle | CJASN Clinical Journal of the American Society of Nephrology

ASN Journal Disclosure Form

As per ASN journal policy, I have disclosed any financial relationships or commitments I have held in the past 36 months as included below. I have listed my Current Employer below to indicate there is a relationship requiring disclosure. If no relationship exists, my Current Employer is not listed.

R. Agarwal reports the following:
Employer: Veteran's Administration (part time); Consultancy: Akebia, Bayer, Boehringer Ingelheim, Eli Lilly, Chinook, Alnylam, Vertex, Intercept Pharmaceuticals, Novartis; Honoraria: Akebia, Bayer, Boehringer Ingelheim, Chinook, Vertex, Intercept Pharmaceuticals, Novartis, Alnylam; Patents or Royalties: UpToDate; and Advisory or Leadership Role: Hypertension, NDT, Akebia, Bayer, Boehringer Ingelheim, Chinook, Vertex, Novartis, Alnylam.

I understand that the information above will be published within the journal article, if accepted, and that failure to comply and/or to accurately and completely report the potential financial conflicts of interest could lead to the following: 1) Prior to publication, article rejection, or 2) Post-publication, sanctions ranging from, but not limited to, issuing a correction, reporting the inaccurate information to the authors' institution, banning authors from submitting work to ASN journals for varying lengths of time, and/or retraction of the published work.

Name: Rajiv Agarwal
Manuscript ID: JASN-2025-001273
Manuscript Title: Baseline kidney function and albuminuria predict greater UACR reduction with finerenone, empagliflozin, or both in the CONFIDENCE Trial
Date of Completion: September 26, 2025
Disclosure Updated Date: July 25, 2025

ASN American Society of Nephrology | JASN Journal of the American Society of Nephrology | KIDNEY360 Accessing Our World From Every Angle | CJASN Clinical Journal of the American Society of Nephrology

ASN Journal Disclosure Form

As per ASN journal policy, I have disclosed any financial relationships or commitments I have held in the past 36 months as included below. I have listed my Current Employer below to indicate there is a relationship requiring disclosure. If no relationship exists, my Current Employer is not listed.

J. Green reports the following:
Employer: Duke University School of Medicine; Consultancy: AstraZeneca, NovoNordisk, Bayer, Anji, Boehringer Ingelheim, Valo, Lilly, Vertex, Corcept, Mineralys, Zealand; Research Funding: Merck, Roche, Lilly, Boehringer Ingelheim, Bluedrop, GentiBio, COUR; and Honoraria: Same listing as for consulting: AstraZeneca, NovoNordisk, Bayer, Anji, Boehringer Ingelheim, Valo, Lilly, Vertex, Corcept, Mineralys, Zealand.

I understand that the information above will be published within the journal article, if accepted, and that failure to comply and/or to accurately and completely report the potential financial conflicts of interest could lead to the following: 1) Prior to publication, article rejection, or 2) Post-publication, sanctions ranging from, but not limited to, issuing a correction, reporting the inaccurate information to the authors' institution, banning authors from submitting work to ASN journals for varying lengths of time, and/or retraction of the published work.

Name: Jennifer B. Green
Manuscript ID: JASN-2025-001273
Manuscript Title: Baseline kidney function and albuminuria predict greater UACR reduction with finerenone, empagliflozin, or both in the CONFIDENCE Trial
Date of Completion: October 2, 2025
Disclosure Updated Date: October 2, 2025

ASN American Society of Nephrology
JASN Journal of the American Society of Nephrology
KIDNEY360 Accessing Our World From Every Angle
CJASN Clinical Journal of the American Society of Nephrology

ASN Journal Disclosure Form

As per ASN journal policy, I have disclosed any financial relationships or commitments I have held in the past 36 months as included below. I have listed my Current Employer below to indicate there is a relationship requiring disclosure. If no relationship exists, my Current Employer is not listed.

H. Heerspink reports the following:
Employer: University Medical Center Groningen; Consultancy: Ongoing consultancy agreements with AstraZeneca, Alexion, Bayer, Boehringer Ingelheim, Biocity Therapeutic, Dimerix, Eli-Lilly, Gilead, Janssen, Novartis, NovoNordisk, Roche, Travere Therapeutics; Research Funding: AstraZeneca, Bayer, Boehringer Ingelheim, NovoNordisk and Janssen research support (grant funding directed to employer); Honoraria: Lecture fees from AstraZeneca and NovoNordisk; and Speakers Bureau: AstraZeneca, Bayer, Novo Nordisk.

I understand that the information above will be published within the journal article, if accepted, and that failure to comply and/or to accurately and completely report the potential financial conflicts of interest could lead to the following: 1) Prior to publication, article rejection, or 2) Post-publication, sanctions ranging from, but not limited to, issuing a correction, reporting the inaccurate information to the authors' institution, banning authors from submitting work to ASN journals for varying lengths of time, and/or retraction of the published work.

Name: Hiddo Jan L. Heerspink
Manuscript ID: JASN-2025-001273
Manuscript Title: Baseline kidney function and albuminuria predict greater UACR reduction with finerenone, empagliflozin, or both in the CONFIDENCE Trial
Date of Completion: September 27, 2025
Disclosure Updated Date: June 7, 2025

ASN American Society of Nephrology JASN Journal of the American Society of Nephrology KIDNEY360 Accessing Our World From Every Angle CJASN Clinical Journal of the American Society of Nephrology

ASN Journal Disclosure Form

As per ASN journal policy, I have disclosed any financial relationships or commitments I have held in the past 36 months as included below. I have listed my Current Employer below to indicate there is a relationship requiring disclosure. If no relationship exists, my Current Employer is not listed.

L. Li reports the following:
Employer: Bayer AG; and Ownership Interest: Bayer AG.

I understand that the information above will be published within the journal article, if accepted, and that failure to comply and/or to accurately and completely report the potential financial conflicts of interest could lead to the following: 1) Prior to publication, article rejection, or 2) Post-publication, sanctions ranging from, but not limited to, issuing a correction, reporting the inaccurate information to the authors' institution, banning authors from submitting work to ASN journals for varying lengths of time, and/or retraction of the published work.

Name: Li Li
Manuscript ID: JASN-2025-001273
Manuscript Title: Baseline kidney function and albuminuria predict greater UACR reduction with finerenone, empagliflozin, or both in the CONFIDENCE Trial
Date of Completion: September 29, 2025
Disclosure Updated Date: July 19, 2025

ASN Journal Disclosure Form

As per ASN journal policy, I have disclosed any financial relationships or commitments I have held in the past 36 months as included below. I have listed my Current Employer below to indicate there is a relationship requiring disclosure. If no relationship exists, my Current Employer is not listed.

N. Li reports the following:
Employer: R&D, Bayer Healthcare

I understand that the information above will be published within the journal article, if accepted, and that failure to comply and/or to accurately and completely report the potential financial conflicts of interest could lead to the following: 1) Prior to publication, article rejection, or 2) Post-publication, sanctions ranging from, but not limited to, issuing a correction, reporting the inaccurate information to the authors' institution, banning authors from submitting work to ASN journals for varying lengths of time, and/or retraction of the published work.

Name: Na Li
Manuscript ID: ASN-2025-001273
Manuscript Title: Baseline kidney function and albuminuria predict greater UACR reduction with finerenone, empagliflozin, or both in the CONFIDENCE Trial
Date of Completion: September 28, 2025
Disclosure Updated Date: July 30, 2025

ASN American Society of Nephrology JASN Journal of the American Society of Nephrology KIDNEY360 Accessing Our World From Every Angle CJASN Clinical Journal of the American Society of Nephrology

ASN Journal Disclosure Form

As per ASN journal policy, I have disclosed any financial relationships or commitments I have held in the past 36 months as included below. I have listed my Current Employer below to indicate there is a relationship requiring disclosure. If no relationship exists, my Current Employer is not listed.

J. Mann reports the following:
Employer: KfH Nierenzentrum; Consultancy: Bayer, Novo Nordisk; Research Funding: Novo Nordisk, Sanofi, Bayer, ICON, Parexel, Cytel, Iqvia, Fortrea; Honoraria: Bayer, Novo Nordisk, Sanofi, Hexal, ICON, Parexel, Cytel, Iqvia, WCG; and Speakers Bureau: Bayer, Hexal, Novo Nordisk.

I understand that the information above will be published within the journal article, if accepted, and that failure to comply and/or to accurately and completely report the potential financial conflicts of interest could lead to the following: 1) Prior to publication, article rejection, or 2) Post-publication, sanctions ranging from, but not limited to, issuing a correction, reporting the inaccurate information to the authors' institution, banning authors from submitting work to ASN journals for varying lengths of time, and/or retraction of the published work.

Name: Johannes F. Mann
Manuscript ID: JASN-2025-001273
Manuscript Title: Baseline kidney function and albuminuria predict greater UACR reduction with finerenone, empagliflozin, or both in the CONFIDENCE Trial
Date of Completion: September 27, 2025
Disclosure Updated Date: July 23, 2025

ASN
American Society of Nephrology
JASN
Journal of the American Society of Nephrology
KIDNEY360
Accessing Our World From Every Angle
CJASN
Clinical Journal of the American Society of Nephrology

ASN Journal Disclosure Form

As per ASN journal policy, I have disclosed any financial relationships or commitments I have held in the past 36 months as included below. I have listed my Current Employer below to indicate there is a relationship requiring disclosure. If no relationship exists, my Current Employer is not listed.

J. McGill reports the following:
Employer: Washington University School of Medicine; Consultancy: Bayer, Lilly, Mannkind, NovoNordisk, Pfizer; Research Funding: Novo Nordisk, Diamyd, Biomea, Lexicon; Patents or Royalties: Washington University; and Other Interests or Relationships: NIH (grant funding, DSMB member); Jaeb Center for Health Resarch (DSMB), Breakthrough T1D (grant funding).

I understand that the information above will be published within the journal article, if accepted, and that failure to comply and/or to accurately and completely report the potential financial conflicts of interest could lead to the following: 1) Prior to publication, article rejection, or 2) Post-publication, sanctions ranging from, but not limited to, issuing a correction, reporting the inaccurate information to the authors' institution, banning authors from submitting work to ASN journals for varying lengths of time, and/or retraction of the published work.

Name: Janet B. McGill
Manuscript ID: JASN-2025-001273
Manuscript Title: Baseline kidney function and albuminuria predict greater UACR reduction with finerenone, empagliflozin, or both in the CONFIDENCE Trial
Date of Completion: October 2, 2025
Disclosure Updated Date: October 2, 2025

ASN American Society of Nephrology | JASN Journal of the American Society of Nephrology | KIDNEY360 Accessing Our World From Every Angle | CJASN Clinical Journal of the American Society of Nephrology

ASN Journal Disclosure Form

As per ASN journal policy, I have disclosed any financial relationships or commitments I have held in the past 36 months as included below. I have listed my Current Employer below to indicate there is a relationship requiring disclosure. If no relationship exists, my Current Employer is not listed.

A. Mottl reports the following:
Employer: University of North Carolina at Chapel Hill; Consultancy: Bayer, Chinook, Otsuka, Novartis, Vera; Research Funding: Alexion, Bayer, Boehringer Ingelheim, Chinook, Duke Clinical Research Institute, Novartis, University of Pennsylvania; Honoraria: UpToDate; and Advisory or Leadership Role: Bayer, Chinook, Novartis, Otsuka, Vera.

I understand that the information above will be published within the journal article, if accepted, and that failure to comply and/or to accurately and completely report the potential financial conflicts of interest could lead to the following: 1) Prior to publication, article rejection, or 2) Post-publication, sanctions ranging from, but not limited to, issuing a correction, reporting the inaccurate information to the authors' institution, banning authors from submitting work to ASN journals for varying lengths of time, and/or retraction of the published work.

Name: Amy K. Mottl
Manuscript ID: JASN-2025-001273
Manuscript Title: Baseline kidney function and albuminuria predict greater UACR reduction with finerenone, empagliflozin, or both in the CONFIDENCE Trial
Date of Completion: September 29, 2025
Disclosure Updated Date: July 18, 2025

ASN American Society of Nephrology | JASN Journal of the American Society of Nephrology | KIDNEY360 Accessing Our World From Every Angle | CJASN Clinical Journal of the American Society of Nephrology

ASN Journal Disclosure Form

As per ASN journal policy, I have disclosed any financial relationships or commitments I have held in the past 36 months as included below. I have listed my Current Employer below to indicate there is a relationship requiring disclosure. If no relationship exists, my Current Employer is not listed.

M. Nangaku reports the following:
Employer: the University of Tokyo Graduate School of Medicine; Consultancy: Kyowa-Kirin, Tanabe-Mitsubishi, Boehringer-Ingelheim; Research Funding: Kyowa-Kirin, Chugai, Boehringer-Ingelheim; and Honoraria: Kyowa-Kirin, Tanabe-Mitsubishi.

I understand that the information above will be published within the journal article, if accepted, and that failure to comply and/or to accurately and completely report the potential financial conflicts of interest could lead to the following: 1) Prior to publication, article rejection, or 2) Post-publication, sanctions ranging from, but not limited to, issuing a correction, reporting the inaccurate information to the authors' institution, banning authors from submitting work to ASN journals for varying lengths of time, and/or retraction of the published work.

Name: Masaomi Nangaku
Manuscript ID: JASN-2025-001273
Manuscript Title: Baseline kidney function and albuminuria predict greater UACR reduction with finerenone, empagliflozin, or both in the CONFIDENCE Trial
Date of Completion: September 26, 2025
Disclosure Updated Date: May 15, 2025

ASN
American Society of Nephrology
JASN
Journal of the American Society of Nephrology
KIDNEY360
Accessing Our World From Every Angle
CJASN
Clinical Journal of the American Society of Nephrology

ASN Journal Disclosure Form

As per ASN journal policy, I have disclosed any financial relationships or commitments I have held in the past 36 months as included below. I have listed my Current Employer below to indicate there is a relationship requiring disclosure. If no relationship exists, my Current Employer is not listed.

J. Rosenstock reports the following:
Employer: Dallas Diabetes Research Consulting; Consultancy: Amgen, Applied Therapeutics, Biomea Fusion, Boehringer Ingelheim, Eli Lilly, Hanmi, Novo Nordisk, Oramed, Regeneron, Roche, Sanofi, Structure Therapeutics, and Terns.; Research Funding: Amgen, Applied Therapeutics, AstraZeneca, Biomea Fusion, Boehringer Ingelheim, Carmot, Corcept, Corxel, Eli Lilly, Hanmi, Merck, Novartis, Novo Nordisk, Pfizer, Regeneron, Roche, Regor, Sanofi, Structure Therapeutics, and Terns.; and Honoraria: Novo Nordisk, Sanofi, Eli Lilly.

I understand that the information above will be published within the journal article, if accepted, and that failure to comply and/or to accurately and completely report the potential financial conflicts of interest could lead to the following: 1) Prior to publication, article rejection, or 2) Post-publication, sanctions ranging from, but not limited to, issuing a correction, reporting the inaccurate information to the authors' institution, banning authors from submitting work to ASN journals for varying lengths of time, and/or retraction of the published work.

Name: Julio Rosenstock
Manuscript ID: JASN-2025-001273
Manuscript Title: Baseline kidney function and albuminuria predict greater UACR reduction with finerenone, empagliflozin, or both in the CONFIDENCE Trial
Date of Completion: October 7, 2025
Disclosure Updated Date: October 7, 2025

ASN American Society of Nephrology
JASN Journal of the American Society of Nephrology
KIDNEY360 Accessing Our World From Every Angle
CJASN Clinical Journal of the American Society of Nephrology

ASN Journal Disclosure Form

As per ASN journal policy, I have disclosed any financial relationships or commitments I have held in the past 36 months as included below. I have listed my Current Employer below to indicate there is a relationship requiring disclosure. If no relationship exists, my Current Employer is not listed.

P. Rossing reports the following:
Employer: Steno Diabetes Center Copenhagen; Research Funding: Novo Nordisk , AstraZeneca, Bayer, Lexicon Pharma; Honoraria: Boehringer Ingelheim, AstraZeneca, Abbott, Novo Nordisk, all honoraria to institution; and Advisory or Leadership Role: Astra Zeneca Bayer , Novo Nordisk, Gilead all honoraria to institution.

I understand that the information above will be published within the journal article, if accepted, and that failure to comply and/or to accurately and completely report the potential financial conflicts of interest could lead to the following: 1) Prior to publication, article rejection, or 2) Post-publication, sanctions ranging from, but not limited to, issuing a correction, reporting the inaccurate information to the authors' institution, banning authors from submitting work to ASN journals for varying lengths of time, and/or retraction of the published work.

Name: Peter Rossing
Manuscript ID: JASN-2025-001273
Manuscript Title: Baseline kidney function and albuminuria predict greater UACR reduction with finerenone, empagliflozin, or both in the CONFIDENCE Trial
Date of Completion: September 27, 2025
Disclosure Updated Date: March 19, 2025

ASN
American Society of Nephrology
JASN
Journal of the American Society of Nephrology
KIDNEY360
Accessing Our World From Every Angle
CJASN
Clinical Journal of the American Society of Nephrology

ASN Journal Disclosure Form

As per ASN journal policy, I have disclosed any financial relationships or commitments I have held in the past 36 months as included below. I have listed my Current Employer below to indicate there is a relationship requiring disclosure. If no relationship exists, my Current Employer is not listed.

C. Scott reports the following:
Employer: Bayer Healthcare Inc

I understand that the information above will be published within the journal article, if accepted, and that failure to comply and/or to accurately and completely report the potential financial conflicts of interest could lead to the following: 1) Prior to publication, article rejection, or 2) Post-publication, sanctions ranging from, but not limited to, issuing a correction, reporting the inaccurate information to the authors' institution, banning authors from submitting work to ASN journals for varying lengths of time, and/or retraction of the published work.

Name: Charlie Scott
Manuscript ID: JASN-2025-001273
Manuscript Title: Baseline kidney function and albuminuria predict greater UACR reduction with finerenone, empagliflozin, or both in the CONFIDENCE Trial
Date of Completion: October 1, 2025
Disclosure Updated Date: February 19, 2025

ASN
American Society of Nephrology
JASN
Journal of the American Society of Nephrology
KIDNEY360
Accessing Our World From Every Angle
CJASN
Clinical Journal of the American Society of Nephrology

ASN Journal Disclosure Form

As per ASN journal policy, I have disclosed any financial relationships or commitments I have held in the past 36 months as included below. I have listed my Current Employer below to indicate there is a relationship requiring disclosure. If no relationship exists, my Current Employer is not listed.

M. Vaduganathan reports the following:
Employer: Brigham and Women's Hospital, Harvard Medical School; Consultancy: Alnylam Pharmaceuticals, American Regent, Amgen, AstraZeneca, Bayer AG, Baxter Healthcare, BMS, Boehringer Ingelheim, Chiesi, Cytokinetics, Esperion, Fresenius Medical Care, Idorsia Pharmaceuticals, Lexicon Pharmaceuticals, Merck, Milestone Pharmaceuticals, Novartis, Novo Nordisk, Pharmacosmos, Recordati, Relypsa, Roche Diagnostics, Sanofi, and Tricog Health; Research Funding: Amgen, AstraZeneca, Boehringer Ingelheim, Galmed, Novartis, Bayer AG, Occlutech, Pharmacosmos, and Impulse Dynamics.; and Speakers Bureau: AstraZeneca, Boehringer Ingelheim, Novartis, Roche Diagnostics, Lexicon Pharmaceuticals, Cytokinetics.

I understand that the information above will be published within the journal article, if accepted, and that failure to comply and/or to accurately and completely report the potential financial conflicts of interest could lead to the following: 1) Prior to publication, article rejection, or 2) Post-publication, sanctions ranging from, but not limited to, issuing a correction, reporting the inaccurate information to the authors' institution, banning authors from submitting work to ASN journals for varying lengths of time, and/or retraction of the published work.

Name: Muthiah Vaduganathan
Manuscript ID: JASN-2025-001273
Manuscript Title: Baseline kidney function and albuminuria predict greater UACR reduction with finerenone, empagliflozin, or both in the CONFIDENCE Trial
Date of Completion: September 26, 2025
Disclosure Updated Date: August 4, 2025
