# Supplementary material for: Baseline Kidney Function, Albuminuria, and Urine Albumin-Creatinine Ratio Reduction with Finerenone, Empagliflozin, or Both: Post Hoc Analyses of CONFIDENCE Trial
Source: J Am Soc Nephrol. 2025 Nov 6;37(4):764–76. doi: 10.1681/ASN.0000000928 (PMC13065124; doi:10.1681/ASN.0000000928)
Supplement: Supplementary file 2 [file jasn-37-764-s002.pdf]

CONFIDENCE INVESTIGATORS BY COUNTRY

Belgium
An Nollet, Jan Yperman Ziekenhuis, Ieper
Bruno Van Vlem, Onze-Lieve-Vrouwziekenhuis VZW, Aalst
Francis Duyck, AZ Delta - Campus Rumbeke, Roeselare
Marijn Speeckaert, Universitair Ziekenhuis Gent, Gent
Peter Doubel, AZ Groeninge, Kortrijk
Pieter Gillard, Universitair Ziekenhuis Leuven, Leuven

Canada
Elie Sahyouni, LMC Diabetes & Endocrinology Ville St Laurent, Saint-Laurent
Giuseppe Mazza, GCP Trials, Montreal
Hitesh Mehta, Regional Kidney Wellness Centre, Brampton
Richard Tytus, Hamilton Medical Research Gr, Hamilton
Sameh Fikry, Dr. Sameh Fikry Medicine Professional Corporation, Waterloo
Sean Peterson, Bluewater Clinical Research Group, Sarnia
Shivinder Jolly, Clinical Research Solutions, Waterloo
William Beaubien-Souligny, Centre hospitalier de l'Université de Montréal, Montreal

Denmark
Claus Juhl, Sydvestjysk sygehus, Esbjerg
Jesper Nørgaard Bech, Regionshospitalet Gødstrup, Holstebro
Peter Rossing, Steno Diabetes Center, Herlev
Thure Krarup, Bispebjerg Hospital, København Nv

France
Bruno Guerci, Centre Hospitalier Régional Universitaire de Nancy - Hôpitaux de Brabois, Vandoeuvre-lès-Nancy
Bruno Verges, Centre Hospitalier Universitaire Dijon, Dijon
Jean-Pierre Fauvel, Centre Hospitalier Universitaire de Lyon, Lyon
Olivier Dupuy, Hôpital Paris Saint Joseph, Paris
Olivier Moranne, Hôpital Universitaire Carémeau, Nîmes, and Hôpital Robert Debré AP-HP, Paris
Pierre-Louis Carron, Centre Hospitalier Universitaire Grenoble Alpes-Hôpital Nord Michallon, La Tronche

Germany
Bernhard Winkelmann, ClinPhenomics GmbH & Co, Frankfurt
Christof Kloos, Universitätsklinikum Jena Klinik, Jena
Christoph Axthelm, Cardiologicum Dresden and Pirna, Dresden
Klaus Busch, Gemeinschaftspraxis Diabetesz., Dortmund
Lutz Stemler, Die Praxis am Ludwigsplatz, Ludwigshafen
Markus van der Giet, Charité – Universitätsmedizin Berlin, Berlin
Thorsten Koch, Diabetes Zentrum Wandsbek, Hamburg

India
Architkumar Patel, Vedanta Kidney Care, Vadodara
Balasubramaniyan Thopplan, Government Kilpauk Medical College Hospital, Chennai
Chandrashekar Matad, Bangalore Medical College & Research Institute, Bangalore
Dinesh Khullar, Max Super Speciality Hospital Saket, Delhi
Ganapathi Bantwal, St. John's Medical College and Hospital, Bengaluru
Hansraj Alva, Vinaya Hospital, Mangalore
Jayakumar EK, Government Medical College, Kozhikode
Jugal Bihari Gupta, Eternal Heart Care Centre and Research Institute, Jaipur
Keshavamurthy CB, Manipal Hospital Mysore, Mysore
Manisha Sahay, Osmania General Hospital, Hyderabad
Atanu Pal, Institute of Post-Graduate Medical Education and Research and Seth Sukhlal Karnani Memorial Hospital, Kolkata
Prabha Dadala Ratna, King George Hospital, Visakhapatnam
Sameer Chaubey, Asian Kidney Hospital & Medical Centre, Nagpur
Sanjay Chunilal Agarwal, Grant Medical Foundation, Pune
Saurabh Agarwal, Ganesh Shankar Vidyarthi Memorial Medical College - Kanpur, Kanpur
Sharma Balram, Sawai Man Singh Medical College Hospital, Jaipur
Siddharth Mavani, Mavani Research Center, Ahmedabad
Sree Bhushan Raju, Nizam's Institute of Medical Sciences, Hyderabad
Sreedhar Reddy, Krishna Institute of Medical Sciences, Secunderabad
Subhash Wangnoo, Indraprastha Apollo Hospital, New Delhi
Tushar Bandgar, King Edward Memorial Hospital, Mumbai
Vernekar Ritesh, KLES Dr Prabhakar Kore Hospital, Belagavi

Israel
Faiad Adawi, Ziv Medical Center, Safed
Genya Aharon-Hananel, Hadassah Medical Center, Jerusalem
Idit Liberty, Soroka Medical Center, Be'er-Sheva
Julio Wainstein, Edith Wolfson Medical Center, Holon

Mazen Elias, Emek Medical Center, Afula
Nimer Assy, Galilee Medical Center, Nahariya
Nomy Levin-Iaina, Barzilai Medical Center, Ashkelon

Italy
Agostino Consoli, Centro di Ricerca Clinica, Chieti
Anna Maria Grazia Veronelli, ASST Santi Paolo e Carlo, Milan
Emanuele Bosi, IRCCS Ospedale San Raffaele, Milan
Enrico Fiaccadori, Azienda Ospedaliero-Universitaria di Parma, Parma
Giancarlo Tonolo, Azienda Sanitaria Locale Olbia – Ospedale S. Giovanni di Dio, Olbia
Giuseppe Penno, Azienda Ospedaliero Universitaria Pisana - Stabilimento di Santa Chiara, Pisa
Paola Ponzani, Azienda Sanitaria Locale, Chiavari, Genoa
Roberta Poli, San Luigi Gonzaga University Hospital, Orbassano
Roberto Cimino, Ospedale Garbagnate Milanese, Garbagnate Milanese
Roberto Trevisan, ASST Papa Giovanni XXIII, Bergamo
Salvatore De Cosmo, Fondazione Casa Sollievo della Sofferenza, San Giovanni Rotondo
Veronica Resi, Policlinico Maggiore di Milano, Milan

Japan
Daishiro Yamada, Jiyugaoka Yamada International Medicine Clinic, Obihiro-Shi
Fumi Umeoka, Saiseikai Matsuyama Hospital, Matsuyama-Shi
Hideo Kanehara, Fukui-ken Saiseikai Hospital, Fukui-Shi
Hidetoshi Kanai, Kokura Memorial Hospital, Kitakyushu-Shi
Kunihisa Kobayashi, Fukuoka University, Chikushino
Masahiko Ochi, Public Central Hospital of Matto Ishikawa, Ishikawa
Masao Ishii, Fukuoka Wajiro Hospital, Fukuoka-shi
Takeshi Osonoi, Nakakinen Clinic Med Corporation, Naka-Shi
Terumasa Hayashi, Osaka General Medical Center, Osaka-Shi
Yoshihide Hirohata, Hirohata Naika Clinic, Kitakyushu-Shi
Yoshimitsu Yamasaki, Kyosokai AMC Nishi-Umeda Clinic, Osaka-Shi

The Netherlands
Jeroen van der Net, Albert Schweitzer Ziekenhuis, Dordrecht
Mirjam Lips, Onze Lieve Vrouwe Gasthuis, Amsterdam
Paul Rootjes, Gelre Ziekenhuizen, Apeldoorn
Peter Luik, Meander Medisch Centrum, Amersfoort
Republic of Korea
Byung Wan Lee, Severance Hospital, Yonsei University College of Medicine, Seoul
Chang Beom Lee, Hanyang University Guri Hospital, Guri-si

Choon-Hee Chung, Yonsei University Wonju Severance Christian Hospital, Wonju-si
Eun Young Lee, Soon Chun Hyang University Hospital Cheonan, Cheonan-si
Jae Myung Yu, Hallym University Kangnam Sacred Heart Hospital, Seoul
Seok Joon Shin, Incheon St. Mary's Hospital, The Catholic University of Korea, Incheon
Soo Lim, Seoul National Bundang Hospital, Seongnam-si
Sung-Gyun Kim, Hallym University Sacred Heart Hospital, Anyang-si
Woo-Je Lee, Asan Medical Center, Seoul
You-Cheol Hwang, Kyung-Hee University Hospital Gangdong, Seoul
Young Min Cho, Seoul National University Hospital, Seoul
Young Sun Kang, Korea University Ansan Hospital, Ansan-si

Spain
Alberto Ortiz Arduan, Hospital Universitario Fundación Jiménez Díaz, Madrid
Alfonso Soto, Complexo Hospital University A Coruña, A Coruña
Cristina Castro, Hospital Universitario Dr. Peset, Valencia
Cristobal Morales, Hospital Vithas Sevilla, Castilleja De La Cuesta
Fernando Cereto Castro, Hospital Quirónsalud Barcelona, Barcelona
Francisco Martinez Deben, Complexo Hospitalario Universitario de Ferrol, Ferrol
Francisco Tinahones Madueno, Hospital Universitario Virgen de la Victoria, Malaga
Hanane Bourarich, Hospital Universitario Príncipe de Asturias, Alcala de Henares
Jose Luis Górriz Teruel, Hospital Clínico Universitario de Valencia, Valencia
Juan Diego Mediavilla, Hospital Universitario Virgen de las Nieves, Granada
Maria Jose Soler Romeo, Hospital Universitario Vall d'Hebron, Barcelona
Maria Marques Vidas, Hospital Universitario Puerta de Hierro Majadahonda, Majadahonda

Taiwan
Chien-Te Lee, Chang Gung Memorial Hospital, Kaohsiung City
Chiz-Tzung Chang, China Medical University Hospital, Taichung
Der-Cherng Tarng, Taipei Veterans General Hospital, Taipei
Ju-Ying Jiang, Far Eastern Memorial Hospital, New Taipei City
Mai-Szu Wu, Taipei Medical University Shuang Ho Hospital, New Taipei City
Ming Ju Wu, Taichung Veterans General Hospital, Taichung
Shih-Te Tu, Changhua Christian Hospital, Changhua

United States of America
Ahmed Awad, Clinical Research Consultants, LCC, Kansas City
Ali Iranmanesh, Salem VA Medical Center, Salem
Amy Mottl, University of North Carolina Kidney Center, Chapel Hill
Ankur Doshi, Victorium Clinical Research, Houston

Ashar Luqman, Renal Associates PC, Dakota Dunes
Bruce Baker, North Texas Kidney Disease Ass, Lewisville
Carol Wysham, MultiCare Rockwood Main Clinic, Spokane
Carolina Solis-Herrera, First Outpatient Research Unit, San Antonio
Csaba Kovesdy, University of Tennessee Health Science Center, Memphis
Dana Mitchell, Global Kidney Center, Houston
David Gaskin, Meridian Clinical Research, Savannah
David LaMond, Blue Sky MD, Hendersonville
German Hernandez, DaVita Clinical Research, El Paso
Gloria Ortiz, Biopharma Informatic LLC - McAllen, McAllen
Guillermo Umpierrez, Emory University School of Medicine, Atlanta
Harold Miller, Crescent City Clinical Research, Metairie
Harvey Serota, St. Louis Heart and Vascular, St. Louis
Iqbal Khalid, Southeast Kidney Associates, East Point
Jared Probst, Olympus Family Medicine, Holladay
Jay Sandberg, Oakland Medical Research, Troy
Jay Shubrook, Touro University California, Vallejo
Jose Mandry, West Orange Endocrinology, Ocoee
Joseph Ravid, Innovative Research Institute, Port Charlotte
Julie Silverstein, Washington University in St. Louis School of Medicine, St. Louis
Keung Lee, Carolina Clinical Research & Consulting at Triad Internal Medicine, Asheboro
Leslie Spry, Somnos Sleep Disorders Center, Lincoln
Mariana Garcia-Touza, Kansas City VA Medical Center, Kansas City
Minesh Rajpal, Southwest Kidney Institute, Surprise
Mohamed El-Shahawy, Academic Medical Research Institute, Los Angeles
Nauman Shahid, Eastern Nephrology Associates - Greenville, Greenville
Osvaldo Brusco, Office of Osvaldo A. Brusco, Corpus Christi
Pablo Pergola, Clinical Advancement Center, San Antonio
Pedro Velasquez Mieyer, Dar Salud Care, PLLC - LifeDoc, Memphis
Piotr Lazowski, South Shore Nephrology, Plymouth
Raj Singh, HEALOR Primary Care, Las Vegas
Rekha John, Eastern Nephrology Associates - Kinston Office, Kinston
Richard Powell, Velocity Clinical Research, Cincinnati
Scott Hines, Crystal Run Healthcare, Middletown
Steve Fordan, Thyroid, Endocrinology, and Diabetes, Dallas
Syed Pervaiz, Santa Rosa Medical Center of Nevada, Las Vegas
Tuan-Huy Tran, Crescent City Clinical Research, Metairie
Usha Peri, North Texas Kidney Disease Associate, Lewisville
Wajdi Al-Shweiat, Lake Michigan Nephrology, PLC, Saint Joseph
Wayne Kotzker, Florida Kidney Physicians, Boca Raton

William Kaye, Metabolic Research Institute Inc., West Palm Beach
William Yang, Northridge Hospital Medical Center, Granada Hills
