# Supplementary material for: Baseline Kidney Function, Albuminuria, and Urine Albumin-Creatinine Ratio Reduction with Finerenone, Empagliflozin, or Both: Post Hoc Analyses of CONFIDENCE Trial
Source: J Am Soc Nephrol. 2025 Nov 6;37(4):764–76. doi: 10.1681/ASN.0000000928 (PMC13065124; doi:10.1681/ASN.0000000928)
Supplement: Supplementary file 3 [file jasn-37-764-s003.pdf]

Supplemental Material Table of Contents

Supplemental Table 1. Fixed effects in the linear mixed-effects models 2

Supplemental Table 2. Number of participants with UACR and eGFR baseline data and UACR data at each timepoint included in the efficacy analyses 3

Supplemental Table 3. Model parameters, mean coefficients and their 95% confidence intervals 4

Supplemental Table 4. Statistical significance of model parameters and comparison of the various models 13

Supplemental Table 5. Percentage change in UACR from baseline 15

Supplemental Table 6. Advanced and serious adverse events by baseline UACR and eGFR subgroups 16

Supplemental Figure 1. Participant disposition 18

Supplemental Figure 2. Plot of percent reduction from baseline UACR according to baseline eGFR versus UACR across the 3 treatment arms in the CONFIDENCE trial. 19

Statistical Analysis Plan

Supplemental Table 1. Fixed effects in the linear mixed-effects models

	**Fixed effects in model**
Model 1	Treatment, visit, treatment × visit.
Model 2	Model 1 plus<br>log baseline UACR, log baseline UACR × visit.
Model 3	Model 2 plus<br>baseline eGFR, baseline eGFR × visit.
Model 4	Model 3 plus<br>treatment, treatment × visit.
Model 5	Model 4 plus<br>age, age × visit, sex, sex × visit; region, region × visit, baseline HbA1c, baseline HbA1c × visit, baseline history of atherosclerotic cardiovascular disease, baseline history of atherosclerotic cardiovascular disease × visit, baseline body mass index, baseline body mass index × visit.
Model 6	Model 5 plus<br>baseline eGFR × treatment, baseline eGFR × treatment x visit, log baseline UACR × treatment, log baseline UACR × treatment x visit.

eGFR, estimated glomerular filtration rate; HbA1c, glycated hemoglobin; UACR, urine albumin-to-urine creatinine ratio.

Supplemental Table 2. Number of participants with UACR and eGFR baseline data and UACR data at each timepoint included in the efficacy analyses

Observations, n	eGFR ≥60 ml/min/1.73 m^2		eGFR <60 ml/min/1.73 m^2		
	UACR		UACR		
	<300 mg/g	≥300 mg/g	<300 mg/g	≥300 mg/g	Total
**Baseline**	89	186	121	400	796
Day 14	86	178	112	372	748
Day 30	83	179	111	379	752
Day 90	81	176	110	362	729
Day 180	80	172	105	356	713
Day 210	73	170	100	352	695

eGFR, estimated glomerular filtration rate; UACR, urine albumin-to-urine creatinine ratio.

Supplemental Table 3. Model parameters, mean coefficients and their 95% confidence intervals

	Model 1 Mean (95% CI)	Model 2 Mean (95% CI)	Model 3 Mean (95% CI)	Model 4 Mean (95% CI)	Model 5 Mean (95% CI)	Model 6 Mean (95% CI)
**Treatment**						
Combination	Ref level	Ref level	Ref level	Ref level	Ref level	Ref level
Finerenone	0.254 (0.164 to 0.344)	0.256 (0.166 to 0.346)	0.254 (0.165 to 0.343)	0.256 (0.167 to 0.345)	0.246 (0.158 to 0.334)	0.453 (–0.201 to 1.107)
Empagliflozin	0.209 (0.119 to 0.298)	0.210 (0.121 to 0.299)	0.207 (0.119 to 0.296)	0.209 (0.121 to 0.297)	0.205 (0.118 to 0.292)	0.220 (–0.481 to 0.920)
**Visit**						
Day 14	Ref level	Ref level	Ref level	Ref level	Ref level	Ref level
Day 30	–0.061 (to 0.115 to –0.008)	–0.174 (–0.378 to 0.031)	–0.023 (–0.134 to 0.087)	–0.134 (–0.369 to 0.101)	–0.029 (–0.464 to 0.406)	0.041 (–0.526 to 0.608)
Day 90	–0.256 (–0.326 to –0.186)	–0.086 (–0.356 to 0.183)	–0.312 (–0.458 to –0.166)	–0.144 (–0.454 to 0.165)	–0.016 (–0.587 to 0.554)	–0.081 (–0.817 to 0.655)
Day 180	–0.364 (–0.463 to –0.266)	–0.148 (–0.534 to 0.238)	–0.212 (–0.417 to –0.006)	0.050 (–0.392 to 0.492)	0.535 (–0.270 to 1.340)	0.547 (–0.514 to 1.609)
Day 210	0.151 (0.057 to 0.246)	0.688 (0.314 to 1.063)	–0.037 (–0.237 to 0.163)	0.490 (0.066 to 0.915)	0.729 (–0.051 to 1.510)	0.384 (–0.627 to 1.395)
**Treatment x visit**						
Combination # day 14	Ref level	Ref level	Ref level	Ref level	Ref level	Ref level
Combination # day 30	Ref level	Ref level	Ref level	Ref level	Ref level	Ref level
Combination # day 90	Ref level	Ref level	Ref level	Ref level	Ref level	Ref level
Combination # day 180	Ref level	Ref level	Ref level	Ref level	Ref level	Ref level
Combination # day 210	Ref level	Ref level	Ref level	Ref level	Ref level	Ref level

Finerenone # day 14	Ref level	Ref level	Ref level	Ref level	Ref level	Ref level
Finerenone # day 30	–0.011 (–0.087 to 0.064)	–0.013 (–0.088 to 0.063)	–0.011 (–0.086 to 0.064)	–0.012 (–0.087 to 0.063)	–0.017 (–0.092 to 0.059)	–0.474 (–1.034 to 0.085)
Finerenone # day 90	0.071 (–0.028 to 0.170)	0.072 (–0.027 to 0.170)	0.071 (–0.028 to 0.170)	0.072 (–0.027 to 0.171)	0.063 (–0.036 to 0.162)	–0.058 (–0.793 to 0.676)
Finerenone # day 180	0.102 (–0.038 to 0.241)	0.103 (–0.036 to 0.242)	0.102 (–0.037 to 0.240)	0.103 (–0.036 to 0.242)	0.084 (–0.054 to 0.222)	0.126 (–0.922 to 1.175)
Finerenone # day 210	–0.125 (–0.260 to 0.010)	–0.122 (–0.256 to 0.011)	–0.123 (–0.258 to 0.011)	–0.121 (–0.255 to 0.012)	–0.136 (–0.269 to – 0.002)	0.574 (–0.423 to 1.571)
Empagliflozin # day 14	Ref level	Ref level	Ref level	Ref level	Ref level	Ref level
Empagliflozin # day 30	0.056 (–0.018 to 0.131)	0.056 (–0.019 to 0.130)	0.057 (–0.018 to 0.131)	0.056 (–0.018 to 0.130)	0.052 (–0.022 to 0.127)	0.400 (–0.197 to 0.997)
Empagliflozin # day 90	0.154 (0.056 to 0.252)	0.155 (0.057 to 0.252)	0.154 (0.057 to 0.252)	0.155 (0.057 to 0.252)	0.149 (0.051 to 0.247)	0.438 (–0.349 to 1.225)
Empagliflozin # day 180	0.177 (0.038 to 0.315)	0.178 (0.040 to 0.317)	0.177 (0.039 to 0.315)	0.179 (0.040 to 0.317)	0.168 (0.031 to 0.305)	0.076 (–1.063 to 1.214)
Empagliflozin # day 210	–0.099 (–0.233 to 0.034)	–0.097 (–0.229 to 0.036)	–0.100 (–0.233 to 0.033)	–0.097 (–0.230 to 0.035)	–0.103 (–0.235 to 0.028)	0.086 (–1.004 to 1.176)
**Baseline UACR and its interaction with visit**						
Baseline Ln (UACR) mg/mg		–0.031 (–0.068 to 0.007)		–0.037 (–0.074 to 0.000)	–0.042 (–0.080 to – 0.005)	–0.026 (–0.096 to 0.044)
Day 14 # baseline Ln (UACR) mg/mg		Ref level		Ref level	Ref level	Ref level
Day 30 # baseline Ln (UACR) mg/mg		0.018 (–0.013 to 0.049)		0.017 (–0.015 to 0.048)	0.015 (–0.018 to 0.047)	0.006 (–0.054 to 0.065)
Day 90 # baseline Ln (UACR) mg/mg		–0.027 (–0.068 to 0.014)		–0.025 (–0.067 to 0.016)	–0.031 (–0.073 to 0.012)	–0.002 (–0.080 to 0.077)

Day 180 # baseline Ln(UACR) mg/mg	–0.034 (–0.093 to 0.025)		–0.039 (–0.099 to 0.020)	–0.049 (–0.109 to 0.011)	–0.053 (–0.167 to 0.061)
Day 210 # baseline Ln(UACR) mg/mg	–0.085 (–0.142 to –0.028)		–0.081 (–0.138 to –0.023)	–0.089 (–0.148 to –0.031)	–0.040 (–0.148 to 0.069)
**Baseline eGFR and its interaction with visit**					
Baseline eGFR values (ml/min/1.73 m^2)		–0.004 (–0.006 to –0.002)	–0.004 (–0.006 to –0.002)	–0.004 (–0.006 to –0.002)	–0.004 (–0.008 to –0.001)
Day 14 # baseline eGFR values (ml/min/1.73 m^2)		Ref level	Ref level	Ref level	Ref level
Day 30 # baseline eGFR values (ml/min/1.73 m^2)		–0.001 (–0.003 to 0.001)	–0.001 (–0.002 to 0.001)	–0.001 (–0.002 to 0.001)	–0.001 (–0.004 to 0.002)
Day 90 # baseline eGFR values (ml/min/1.73 m^2)		0.001 (–0.001 to 0.003)	0.001 (–0.001 to 0.003)	0.001 (–0.002 to 0.003)	–0.002 (–0.006 to 0.003)
Day 180 # baseline eGFR values (ml/min/1.73 m^2)		–0.003 (–0.006 to 0.001)	–0.003 (–0.006–0.000)	–0.003 (–0.007 to –0.000)	–0.003 (–0.009 to 0.003)
Day 210 # baseline eGFR values (ml/min/1.73 m^2)		0.003 (0.000 to 0.007)	0.003 (–0.000 to 0.006)	0.003 (–0.000 to 0.006)	0.004 (–0.002 to 0.009)
**Covariates and their interactions with visits**					
Age				–0.004 (–0.008 to –0.000)	–0.004 (–0.008 to –0.000)
Males				0.074 (–0.009 to 0.158)	0.073 (–0.011 to 0.156)
North America				Ref level	Ref level
Europe				0.019 (–0.080 to 0.119)	0.014 (–0.085 to 0.114)
Asia				0.063 (–0.036 to 0.161)	0.060 (–0.039 to 0.159)

Baseline HbA1c (%)	–0.014 (–0.044 to 0.015)	–0.015 (–0.045 to 0.014)
Baseline BMI (kg/cm^2)	0.003 (–0.004 to 0.009)	0.002 (–0.005 to 0.009)
ASCVD	–0.085 (–0.167 to –0.003)	–0.082 (–0.164 to –0.001)
Day 14 # age	Ref level	Ref level
Day 30 # age	–0.001 (–0.004 to 0.002)	–0.001 (–0.004 to 0.002)
Day 90 # age	–0.002 (–0.006 to 0.002)	–0.002 (–0.006 to 0.002)
Day 180 # age	–0.007 (–0.013 to –0.001)	–0.007 (–0.013 to –0.001)
Day 210 # age	–0.005 (–0.011 to 0.000)	–0.005 (–0.011 to 0.000)
Males # day 14	Ref level	
Males # day 30	–0.045 (–0.116 to 0.027)	–0.040 (–0.112 to 0.031)
Males # day 90	0.037 (–0.058 to 0.131)	0.032 (–0.063 to 0.127)
Males # day 180	0.157 (0.024 to 0.290)	0.158 (0.025 to 0.291)
Males # day 210	0.125 (–0.003 to 0.253)	0.123 (–0.005 to 0.251)
North America # day 14	Ref level	Ref level
North America # day 30	Ref level	Ref level
North America # day 90	Ref level	Ref level
North America # day 180	Ref level	Ref level

North America # day 210	Ref level	Ref level
Europe # day 14	Ref level	Ref level
Europe # day 30	–0.044 (–0.129 to 0.041)	–0.039 (–0.124 to 0.046)
Europe # day 90	–0.059 (–0.172 to 0.053)	–0.055 (–0.167 to 0.058)
Europe # day 180	–0.101 (–0.258–0.056)	–0.105 (–0.262–0.053)
Europe # day 210	–0.024 (–0.176 to 0.127)	–0.032 (–0.183 to 0.119)
Asia # day 14	Ref level	Ref level
Asia # day 30	0.007 (–0.078 to 0.091)	0.006 (–0.078 to 0.090)
Asia # day 90	–0.006 (–0.117 to 0.106)	–0.005 (–0.116 to 0.106)
Asia # day 180	–0.035 (–0.191 to 0.122)	–0.038 (–0.194 to 0.119)
Asia # day 210	0.009 (–0.142 to 0.160)	0.005 (–0.146 to 0.156)
Day 14 # baseline HbA1c (%)	Ref level	Ref level
Day 30 # baseline HbA1c (%)	0.003 (–0.023 to 0.028)	0.002 (–0.023 to 0.027)
Day 90 # baseline HbA1c (%)	0.010 (–0.023–0.044)	0.010 (–0.024–0.043)
Day 180 # baseline HbA1C (%)	0.008 (–0.038 to 0.054)	0.008 (–0.039 to 0.054)

Day 210 # baseline HbA1c (%)	0.014 (–0.031 to 0.058)	0.012 (–0.032 to 0.057)
Day 14 # baseline BMI (kg/cm^2)	Ref level	Ref level
Day 30 # baseline BMI (kg/cm^2)	–0.000 (–0.006 to 0.006)	0.000 (–0.006 to 0.006)
Day 90 # baseline BMI (kg/cm^2)	–0.001 (–0.009 to 0.006)	–0.001 (–0.009 to 0.007)
Day 180 # baseline BMI (kg/cm^2)	–0.002 (–0.012 to 0.009)	–0.002 (–0.013 to 0.009)
Day 210 # baseline BMI (kg/cm^2)	0.000 (–0.010 to 0.011)	–0.000 (–0.011 to 0.010)
ASCVD # day 14	Ref level	Ref level
ASCVD # day 30	0.027 (–0.043 to 0.096)	0.024 (–0.046 to 0.094)
ASCVD # day 90	0.048 (–0.045 to 0.141)	0.048 (–0.045 to 0.141)
ASCVD # day 180	0.004 (–0.126 to 0.134)	0.006 (–0.125 to 0.136)
ASCVD # day 210	–0.001 (–0.126 to 0.125)	0.002 (–0.123 to 0.127)
**Treatment x baseline eGFR**		
Combination # baseline eGFR values (ml/min/1.73 m^2)		Ref level
Finerenone # baseline eGFR values (ml/min/1.73 m^2)		–0.002 (–0.007 to 0.003)
Empagliflozin # baseline eGFR values (ml/min/1.73 m^2)		0.002 (–0.003 to 0.007)

**Treatment x baseline eGFR x visit**	
Day 14 # combination # baseline eGFR values (ml/min/1.73 m^2)	Ref level
Day 14 # finerenone # baseline eGFR values (ml/min/1.73 m^2)	Ref level
Day 14 # empagliflozin # baseline eGFR values (ml/min/1.73 m^2)	Ref level
Day 30 # combination # baseline eGFR values (ml/min/1.73 m^2)	Ref level
Day 30 # finerenone # baseline eGFR values (ml/min/1.73 m^2)	0.001 (–0.003 to 0.006)
Day 30 # empagliflozin # baseline eGFR values (ml/min/1.73 m^2)	–0.001 (–0.006 to 0.003)
Day 90 # combination # baseline eGFR values (ml/min/1.73 m^2)	Ref level
Day 90 # finerenone # baseline eGFR values (ml/min/1.73 m^2)	0.005 (–0.000 to 0.011)
Day 90 # empagliflozin # baseline eGFR values (ml/min/1.73 m^2)	0.001 (–0.004 to 0.007)
Day 180 # combination # baseline eGFR values (ml/min/1.73 m^2)	Ref level
Day 180 # finerenone # baseline eGFR values (ml/min/1.73 m^2)	–0.002 (–0.010 to 0.006)
Day 180 # empagliflozin # baseline eGFR values (ml/min/1.73 m^2)	0.001 (–0.008 to 0.009)
Day 210 # combination # baseline eGFR values (ml/min/1.73 m^2)	Ref level

Day 210 # finerenone # baseline eGFR values (ml/min/1.73 m^2)	–0.005 (–0.012 to 0.003)
day 210 # empagliflozin # baseline eGFR values (ml/min/1.73 m^2)	0.002 (–0.006 to 0.010)
**Treatment x baseline UACR**	
Combination # baseline Ln (UACR) mg/mg	Ref level
Finerenone # baseline Ln (UACR) mg/mg	–0.019 (–0.110 to 0.072)
Empagliflozin # baseline Ln (UACR) mg/mg	–0.018 (–0.112 to 0.076)
**Treatment x baseline UACR x visit**	
Day 14 # combination # baseline Ln (UACR) mg/mg	Ref level
Day 14 # finerenone # baseline Ln (UACR) mg/mg	Ref level
Day 14 # empagliflozin # Baseline Ln (UACR) mg/mg	Ref level
Day 30 # combination # baseline Ln (UACR) mg/mg	Ref level
Day 30 # finerenone # baseline Ln (UACR) mg/mg	0.059 (–0.019 to 0.137)
Day 30 # empagliflozin # baseline Ln (UACR) mg/mg	–0.045 (–0.125 to 0.035)
Day 90 # combination # baseline Ln (UACR) mg/mg	Ref level
Day 90 # finerenone # baseline Ln (UACR) mg/mg	–0.027 (–0.130 to 0.075)
Day 90 # empagliflozin # baseline Ln (UACR) mg/mg	–0.058 (–0.164 to 0.048)

Day 180 # combination # baseline Ln (UACR) mg/mg						Ref level
Day 180 # finerenone # baseline Ln (UACR) mg/mg						0.011 (–0.135 to 0.158)
Day 180 # empagliflozin # baseline Ln (UACR) mg/mg						0.008 (–0.145 to 0.160)
Day 210 # combination # baseline Ln (UACR) mg/mg						Ref level
Day 210 # finerenone # baseline Ln (UACR) mg/mg						–0.073 (–0.214 to 0.069)
Day 210 # empagliflozin # baseline Ln (UACR) mg/mg						–0.046 (–0.193 to 0.101)
Intercept	–0.455 (–0.518 to –0.391)	–0.260 (–0.504 to –0.015)	–0.253 (–0.385 to –0.122)	–0.010 (–0.289 to 0.268)	0.280 (–0.226 to 0.787)	0.191 (–0.467 to 0.849)

The coefficients and their 95% Cis are the log ratio of UACR at a given visit to baseline. Statistical significance of the models and the model parameters are shown in Table S2.

ASCVD, atherosclerotic cardiovascular disease; BMI, body mass index; CI, confidence interval; eGFR, estimated glomerular filtration rate; HbA1c, glycated hemoglobin; ref, reference; UACR, urine albumin-to-urine creatinine ratio.

Supplemental Table 4. Statistical significance of model parameters and comparison of the various models

	Model 1	Model 2	Model 3	Model 4	Model 5	Model 6
**Treatment**	<0.001	<0.001	<0.001	<0.001	<0.001	0.4
**Visit**	<0.001	<0.001	<0.001	0.06	0.3	0.8
**Treatment x visit**	<0.01	<0.01	<0.01	<0.01	<0.01	0.06
Baseline Ln (UACR)		0.1		0.05	<0.05	0.5
Baseline Ln (UACR) x visit		<0.01		<0.01	<0.01	0.8
**Overall UACR effect**		**<0.01**		**<0.01**	**<0.001**	0.8
**Baseline eGFR**			<0.001	<0.001	<0.001	<0.05
**Baseline eGFR x visit**			<0.05	<0.05	0.05	0.4
**Covariates and their interactions with visits**						
**Age**					<0.05	<0.05
**Age x visit**					0.08	0.08
**Sex**					0.08	0.09
**Sex x visit**					<0.01	<0.05
**Region**					0.4	0.5
**Region x visit**					0.9	0.9
**ASCVD**					<0.05	<0.05

**ASCVD x visit**					0.9	0.9
**Baseline HbA1c**					0.3	0.3
**Baseline HbA1c x visit**					1	1
**Baseline BMI**					0.5	0.5
**Baseline BMI x visit**					1	1
**Baseline eGFR x treatment**						0.4
**Baseline eGFR x treatment x visit**						0.2
**Baseline Ln UACR x treatment**						0.9
**Baseline Ln UACR x treatment x visit**						0.2
Model Chi squared	243.2	263.8	269.9	291.6	356.1	381.9
Model log likelihood	–2904.1	–2894.4	–2891.6	–2881.6	–2852.5	–2840.5
Comparison of models		2 vs 1	3 vs 1	4 vs 3 4 vs 2	5 vs 4	6 vs 5
Likelihood ratio chi 2		19.4	25	20.2 25.7	58.1	23.9
Likelihood ratio p value for comparison		0.002	<0.001	0.001 <0.001	0.008	0.25

ASCVD, atherosclerotic cardiovascular disease; BMI, body mass index; eGFR, estimated glomerular filtration rate; HbA1c, glycated hemoglobin; UACR, urine albumin-to-urine creatinine ratio.

Supplemental Table 5. Percentage change in UACR from baseline

Treatment	Day	Percent change in UACR from baseline (95% CI)
**Combination**	14	−36 (−40 to −32)
**Combination**	30	−40 (−44 to −36)
**Combination**	90	−50 (−54 to −46)
**Combination**	180	−55 (−60 to −50)
**Combination**	210	−26 (−33 to −18)
**Finerenone**	14	−18 (−23 to−13)
**Finerenone**	30	−24 (−29 to −19)
**Finerenone**	90	−33 (−38 to −27)
**Finerenone**	180	−38 (−44 to −31)
**Finerenone**	210	−17 (−25 to −8)
**Empagliflozin**	14	−22 (−26 to −17)
**Empagliflozin**	30	−22 (−27 to −17)
**Empagliflozin**	90	−29 (−35 to −24)
**Empagliflozin**	180	−35 (−41 to −28)
**Empagliflozin**	210	−18 (−25 to −9)

Marginal means derived from linear mixed model 5.
CI, confidence interval; UACR, urine albumin-to-urine creatinine ratio.

Supplemental Table 6. Advanced and serious adverse events by baseline UACR and eGFR subgroups

	eGFR ≥60 ml/min/1.73 m^2 UACR (mg/g)		eGFR <60 ml/min/1.73 m^2 UACR (mg/g)		
	<300 mg/g	≥300 (mg/g)	<300 mg/g	≥300 (mg/g)	Total
**Any adverse event — n (%)**	43 (48)	101 (54)	71 (59)	198 (50)	413 (52)
**Treatment group — n (%)**					
Combination	20 (61)	33 (55)	21 (53)	69 (52)	143 (54)
Finerenone	9 (38)	37 (51)	29 (63)	61 (50)	136 (52)
Empagliflozin	14 (44)	31 (57)	21 (60)	68 (47)	134 (51)
**Adverse event leading to treatment discontinuation — n (%)**	5 (6)	4 (2)	7 (6)	13 (3)	29 (4)
**Treatment group — n (%)**					
Combination	2 (6)	1 (2)	5 (13)	4 (3)	12 (5)
Finerenone	2 (8)	2 (3)	2 (4)	3 (3)	9 (3)
Empagliflozin	1 (3)	1 (2)	0 (0)	6 (4)	8 (3)
**Serious adverse event**	7 (8)	9 (5)	10 (8)	24 (6)	50 (6)
**Treatment group — n (%)**					
Combination	4 (12)	1 (2)	5 (13)	8 (6)	18 (7)
Finerenone	1 (4)	4 (6)	1 (2)	10 (8)	16 (6)

Empagliflozin	2 (6)	4 (7)	4 (11)	6 (4)	16 (6)
Serious adverse event leading to treatment discontinuation — n (%)	2 (2)	1 (0.5)	1 (0.8)	3 (0.8)	7 (0.9)
**Treatment group — n (%)**					
Combination	1 (3)	0 (0.0)	1 (3)	1 (0.7)	3 (1)
Finerenone	0 (0.0)	1 (1)	0 (0.0)	2 (2)	3 (1)
Empagliflozin	1 (3)	0 (0.0)	0 (0.0)	0 (0.0)	1 (0.4)
**Adverse event with death as the outcome — n (%)**	0 (0.0)	2 (1)	0 (0.0)	4 (1)	6 (0.8)
**Treatment group — n (%)**					
Combination	0 (0.0)	0 (0.0)	0 (0.0)	3 (2)	3 (1)
Finerenone	0 (0.0)	0 (0.0)	0 (0.0)	0 (0.0)	0 (0.0)
Empagliflozin	0 (0.0)	2 (4)	0 (0.0)	1 (0.7)	3 (1)

eGFR, estimated glomerular filtration rate; HbA1c, glycated hemoglobin; UACR, urine albumin-to-urine creatinine ratio.

Supplemental Figure 1. Participant disposition

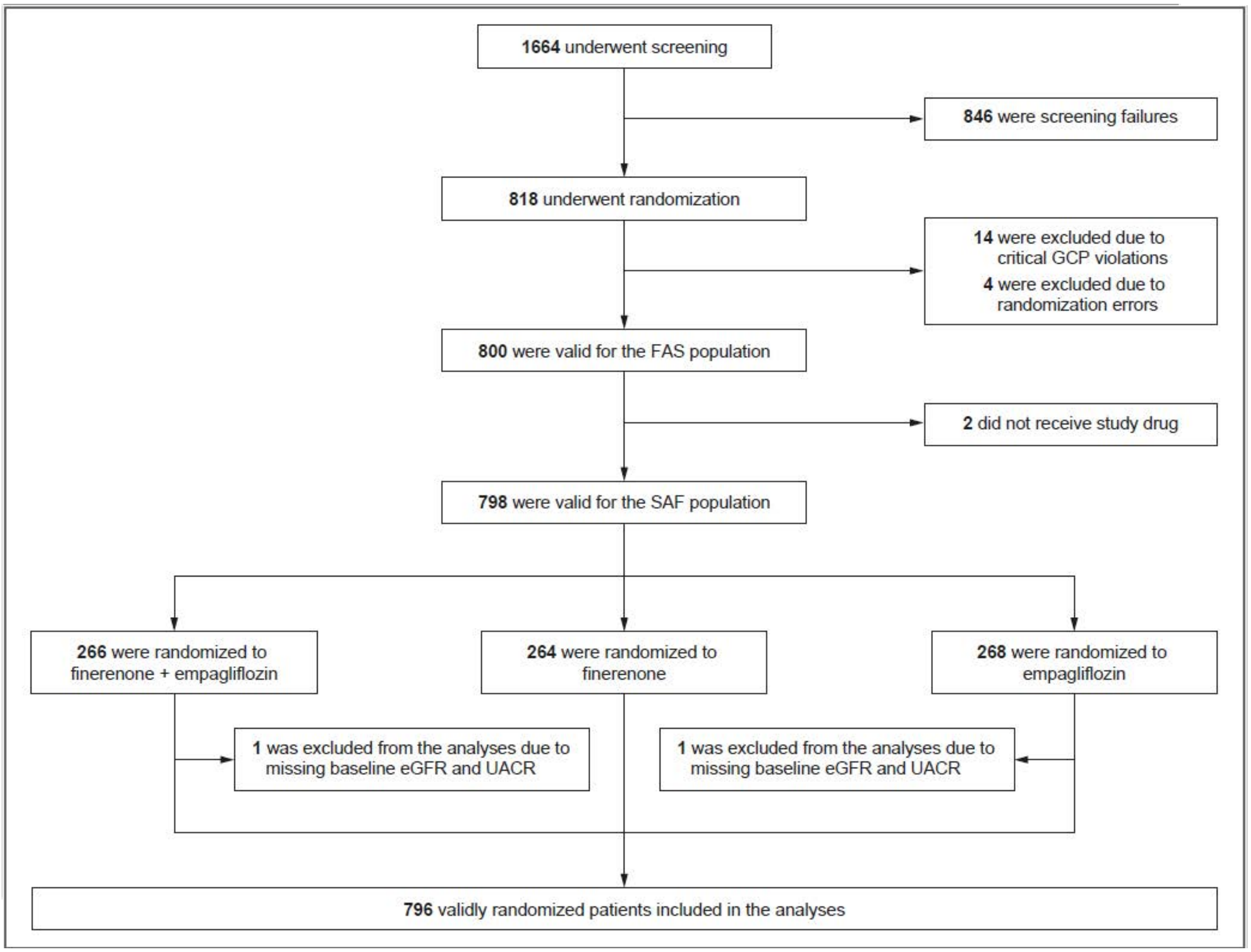

eGFR, estimated glomerular filtration rate; GCP, Good Clinical Practice; UACR, urine albumin-to-urine creatinine ratio.

Supplemental Figure 2. Plot of percent reduction from baseline UACR according to baseline eGFR versus UACR across the 3 treatment arms in the CONFIDENCE trial.

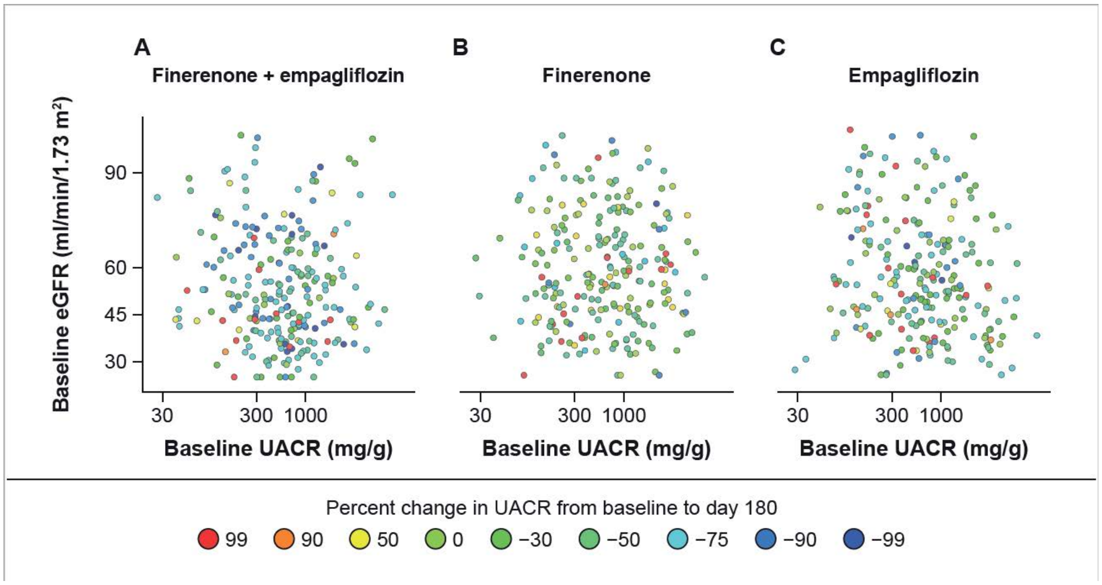

eGFR, estimated glomerular filtration rate; UACR, urine albumin-to-urine creatinine ratio.

This file contains:

Clinical study protocol Version 1.0	Page 2-100
Clinical study protocol Version 2.0	Page 101-208
Protocol Amendment Summary of Change	Page 102-105
Statistical Analysis Plan Version 1.0	Page 209-255

Title Page

Protocol Title:

A parallel-group treatment, Phase 2, double-blind, three-arm study to assess efficacy and safety of finerenone plus empagliflozin compared with either finerenone or empagliflozin in participants with chronic kidney disease and type 2 diabetes.

**Protocol Number:**	21839
**Amendment Number:**	Not applicable
**Compound Number:**	Finerenone / BAY 94-8862
**Brief Title:**	Combination of finerenone and empagliflozin in participants with CKD and T2D
**Study Phase:**	Phase 2
**Acronym:**	CONFIDENCE (**CO**mbinatio**N** effect of **FI**nerenone an**D** **E**mpagliflozi**N** in participants with CKD and T2D using an UA**C**R **E**ndpoint study)
**Sponsor Name:**	Bayer AG

Legal Registered Address:

Non-US territories: Bayer AG, 51368 Leverkusen, Germany

US territory: Bayer HealthCare Pharmaceuticals Inc., 100 Bayer Boulevard, P.O. Box 915, Whippany NJ 07981-0915, USA

Regulatory Agency Identifier Number(s):

IND: 117847

EudraCT: 2021-003037-11

Protocol Date: 15 November 2021

Medical Monitor name and contact information will be provided separately.

Name: PPD Role: Global Medical Leader

This is an electronically generated document that does not bear any sponsor signatures. The signature of the sponsor's medically responsible person is filed in the TMF and available on request.

Confidential

The information provided in this document is strictly confidential and is intended solely for the performance of the clinical investigation. Reproduction or disclosure of this document, whether in part or in full, to parties not associated with the clinical investigation or its use for any other purpose without the prior written consent of the sponsor is not permitted.

Throughout this document, symbols indicating proprietary names (®, TM) may not be displayed. Hence, the appearance of product names without these symbols does not imply that these names are not protected.

Table of Contents

Title Page 1
Table of Contents 2
Table of Tables 5
Table of Figures 5
1. Protocol Summary 7
1.1 Synopsis 7
1.2 Schema 12
1.3 Schedule of Activities 13
2. Introduction 18
2.1 Study Rationale 18
2.2 Background 19
2.3 Benefit/Risk Assessment 21
2.3.1 Risk Assessment 21
2.3.2 Benefit Assessment 22
2.3.3 Overall Benefit: Risk Conclusion 23
3. Objectives and Endpoints and Estimands 24
4. Study Design 26
4.1 Overall Design 26
4.2 Scientific Rationale for Study Design 28
4.2.1 Participant’s Input into Design 29
4.3 Justification for Dose 29
4.3.1 Finerenone 29
4.3.2 Empagliflozin 29
4.4 End of Study Definition 30
5. Study Population 30
5.1 Inclusion Criteria 30
5.2 Exclusion Criteria 31
5.3 Lifestyle Considerations 33
5.3.1 Meals and Dietary Restrictions 33
5.3.2 Other Lifestyle Considerations 33
5.4 Screen Failures 33
5.5 Criteria for Temporarily Delaying Enrollment/Randomization/Study Intervention Administration 34
6. Study Intervention(s) and Concomitant Therapy 34
6.1 Study Interventions Administered 34
6.1.1 Study Interventions 34
6.1.2 Medical Devices 37
6.2 Preparation/Handling/Storage/Accountability 37
6.3 Measures to Minimize Bias: Randomization and Blinding 37
6.4 Study Intervention Compliance 38
6.5 Dose Modification 39
6.5.1 Finerenone 39
6.5.2 Empagliflozin 42

6.6 Continued Access to Study Intervention After the End of the Study ... 42
6.7 Treatment of Overdose ... 42
6.7.1 Finerenone ... 42
6.7.2 Empagliflozin ... 42
6.7.2.1 Symptoms ... 42
6.7.2.2 Therapy ... 42
6.8 Concomitant Therapy ... 43
6.8.1 Permitted Concomitant Therapy ... 43
6.8.2 Prohibited Concomitant Therapy ... 44
6.8.3 Hyperkalemia Events ... 44
6.8.4 Unexpected Acute Declines in eGFR ... 44
6.8.5 Volume Depletion/Hypotension ... 44
6.8.6 Ketoacidosis ... 45

7. Discontinuation of Study Intervention and Participant Discontinuation/Withdrawal ... 46
7.1 Discontinuation of Study Intervention ... 46
7.1.1 Permanent Discontinuation of Study Intervention ... 46
7.1.2 Temporary Discontinuation ... 46
7.2 Participant Discontinuation/Withdrawal from the Study ... 47
7.3 Lost to Follow-Up ... 47

8. Study Assessments and Procedures ... 48
8.1 Efficacy Assessment ... 48
8.2 Safety Assessments ... 49
8.2.1 Vital Signs ... 49
8.2.1.1 Ambulatory Blood Pressure Monitoring – Only in Part A ... 49
8.2.2 Physical Examinations ... 50
8.2.3 Electrocardiograms ... 50
8.2.4 Laboratory Assessments ... 50
8.2.5 Other Safety Assessments ... 51
8.2.5.1 eGFR ... 51
8.2.5.2 Monitoring of Potassium ... 51
8.2.5.3 Acute Kidney Injury ... 52
8.2.5.4 Severe Hypoglycemia ... 52
8.2.5.5 Symptomatic Hypotension (Including Volume Depletion) ... 52
8.2.5.6 Genital Mycotic Events ... 52
8.2.5.7 Ketoacidosis Events ... 52
8.2.5.8 Necrotizing Fasciitis of the Perineum (Fournier's Gangrene) Events ... 52
8.2.5.9 Urosepsis and Pyelonephritis Events ... 52
8.2.6 Pregnancy Testing ... 53
8.2.7 Suicidal Ideation and Behavior Risk Monitoring ... 53
8.3 AEs, SAEs, and Other Safety Reporting ... 53
8.3.1 Time Period and Frequency for Collecting AE and SAE Information ... 53
8.3.2 Method of Detecting AEs and SAEs ... 53
8.3.3 Follow-Up of AEs and SAEs ... 54
8.3.4 Regulatory Reporting Requirements for SAEs ... 54
8.3.5 Pregnancy ... 54
8.3.6 Cardiovascular and Death Events ... 55

8.3.7 Disease-Related Events and/or Disease-Related Outcomes Not Qualifying for Expedited Reporting as AE or SAE 55
8.3.8 Adverse Events of Special Interest 56
8.4 Pharmacokinetics 56
8.5 Genetics and/or Pharmacogenomics 57
8.6 Biomarkers 57
8.7 Immunogenicity Assessments 58
8.8 Health Economics 58

9. Statistical Considerations 58
9.1 Statistical Hypotheses 58
9.2 Sample Size Determination 58
9.3 Analysis Sets 60
9.4 Statistical Analyses 61
9.4.1 General Considerations 61
9.4.2 Disposition, Baseline, History, Demography and Medication 62
9.4.3 Disposition 62
9.4.3.1 Population Characteristics 62
9.4.3.2 Demography and Other Baseline Characteristics 63
9.4.3.3 Medical History 63
9.4.3.4 Concomitant Medication 63
9.4.3.5 Treatment Duration, Extent of Exposure, Up-Titration Status, and Compliance 63
9.4.4 Primary Efficacy Endpoints 64
9.4.5 Secondary Efficacy Endpoints 65
9.4.5.1 Relative Change in UACR Category 65
9.4.5.2 Subgroup Analyses 65
9.4.6 Safety Analysis 66
9.4.6.1 Adverse Event 67
9.4.6.2 Laboratory Data 67
9.4.6.3 Vital Signs, Including Weight and BMI 68
9.4.6.4 Further Safety Variables 68
9.4.7 Missing Data/Dropouts 70
9.5 Interim Analysis 71

10. Supporting Documentation and Operational Considerations 72
10.1 Appendix 1: Regulatory, Ethical, and Study Oversight Considerations 72
10.1.1 Regulatory and Ethical Considerations 72
10.1.2 Financial Disclosure 72
10.1.3 Informed Consent Process 72
10.1.4 Data Protection 73
10.1.5 Committee Structure 73
10.1.5.1 Data Monitoring Committee 73
10.1.5.2 Steering Committee 73
10.1.6 Dissemination of Clinical Study Data 74
10.1.7 Data Quality Assurance 74
10.1.8 Source Documents 75
10.1.9 Study and Site Start and Closure 75
10.1.10 Publication Policy 76
10.2 Appendix 2: Clinical Laboratory Tests 76
10.2.1 Biomarkers 77

10.2.2 Pharmacokinetics ... 78
10.3 Appendix 3: Disease-Related Event Definitions ... 78
10.3.1 Myocardial Infarction ... 78
10.3.1.1 Criteria for Myocardial Infarction ... 78
10.3.2 Stroke ... 80
10.3.2.1 Ischemic Stroke ... 81
10.3.2.2 Hemorrhagic Stroke ... 81
10.3.2.3 Undetermined Stroke ... 81
10.3.3 New Onset of HF ... 81
10.3.4 Hospitalization for HF ... 81
10.3.5 Chronic Sustained Decrease in EGFR ... 82
10.4 Appendix 4: AEs and SAEs: Definitions and Procedures for Recording, Evaluating, Follow-Up, and Reporting ... 83
10.4.1 Definition of AE ... 83
10.4.2 Definition of SAE ... 84
10.4.3 Recording and Follow-Up of AE and/or SAE ... 85
10.4.4 Reporting of SAEs ... 86
10.5 Appendix 5: Death Events Definition ... 87
10.5.1 Cardiovascular Death ... 87
10.5.1.1 Death due to Acute Myocardial Infarction ... 87
10.5.1.2 Sudden Cardiac Death ... 88
10.5.1.3 Undetermined Death ... 88
10.5.1.4 Death due to Heart Failure ... 88
10.5.1.5 Death due to Stroke ... 89
10.5.1.6 Death due Cardiovascular Procedures ... 89
10.5.1.7 Death due to Other Cardiovascular Causes ... 89
10.5.2 Renal Death ... 89
10.5.3 Non-Cardiovascular and Non-Renal Deaths ... 90
10.6 Appendix 6: Contraceptive Guidance and Collection of Pregnancy Information ... 90
10.7 Appendix 7: Calculating the Child Pugh Score ... 92
10.8 Appendix 8: Guidance on Use of Common CYP Inhibitors and Inducers ... 93
10.9 Appendix 9: Abbreviations ... 94

11. References ... 95

Table of Tables

Table 1-1: Schedule of Activities ... 14
Table 6-1: Dose Adjustments of Finerenone ... 40
Table 6-2: Guidance for Finerenone Dose Adjustment at Day 30 and Subsequent Visits (Local Laboratory) Based on K+ ... 40
Table 9-1: Analysis Sets ... 60
Table 10-1: Protocol-Required Laboratory Tests ... 76
Table 10-2: Grading of Severity of Liver Disease (adapted from *Pugh et al., 1973*) ... 92
Table 10-3: Classification Using the Added Score from Table 10-2 ... 92
Table 10-4: Cytochrome P450: List of Concomitant Medication ... 93

Table of Figures

Figure 1-1: Study Design 12

1. Protocol Summary

1.1 Synopsis

Protocol Title: A parallel-group treatment, Phase 2, double-blind, three-arm study to assess efficacy and safety of finerenone plus empagliflozin compared with either finerenone or empagliflozin alone in participants with chronic kidney disease and type 2 diabetes

Brief Title: Combination of finerenone and empagliflozin in participants with CKD and T2D

Rationale:

In the FIDELIO-DKD and FIGARO-DKD phase 3 clinical trials, finerenone, a nonsteroidal, selective antagonist of the mineralocorticoid receptor (MRA), has proven its efficacy and safety to reduce risk of kidney disease progression and cardiovascular (CV) events in patients with chronic kidney disease (CKD) and type 2 diabetes (T2D).

Empagliflozin is a sodium-glucose cotransporter-2 inhibitor (SGLT2i) indicated to reduce the risk of CV death in adult patients with T2D and established CV disease. Empagliflozin has shown evidence of reduction of kidney disease progression and CV events in patients with or without T2D and CKD in CV trials and is now investigated in a dedicated CKD trial. Other SGLT2i (dapagliflozin and canagliflozin) showed renal and CV efficacy in patients with CKD with and without T2D, which led to a label change and updated guideline recommendations.

In a recent preclinical study, it has been shown that finerenone and empagliflozin have an additive effect on urinary protein-to-creatinine ratio, a predictor of CKD progression, and CV adverse outcomes in CKD patients.

In pooled analysis of FIDELIO-DKD/FIGARO-DKD (FIDELITY; data on file), it has been shown in the subgroup of patients treated with SGLT2i at baseline that there is a synergy in reduction of urinary albumin-to-creatinine ratio (UACR) and prevention of events.

This study aims to demonstrate that the initial combined use of finerenone and empagliflozin is superior to either empagliflozin alone, or finerenone alone, in reducing UACR from baseline to 180 days. UACR is a measurement of albuminuria, a predictor of long-term renal and CV adverse outcomes in T2D patients.

Objectives and Endpoints and Estimands:

Objectives	Endpoints and Estimands
**Primary**	
• To demonstrate that combination therapy using finerenone and empagliflozin is superior in reducing UACR than either empagliflozin or finerenone alone	Primary Endpoints:<br>• Relative change from baseline in UACR at 180 days in combination therapy group versus empagliflozin alone<br>or<br>• Relative change from baseline in UACR at 180 days in combination therapy group versus finerenone alone
	Intercurrent events: Participants who discontinues study medication before 180 days, dialysis or kidney transplantation, and death
	Summary Measures:<br>• Mean ratio of change from baseline to Day 180 in UACR for the combination therapy group, to empagliflozin alone<br>• Mean ratio of change from baseline to Day 180 in UACR for the combination therapy group, to finerenone alone
**Secondary**	
• To further investigate the efficacy of combination therapy using finerenone and empagliflozin versus either finerenone or empagliflozin alone	• Relative change in UACR between end of treatment visit and 30 days after end of treatment visit<br>• Relative change in UACR between 30 days after end of treatment visit and baseline<br>• Relative change in UACR category (>30%, >40%, >50%) at 180 days

Objectives	Endpoints and Estimands
• To evaluate the safety of combination therapy using finerenone and empagliflozin versus either finerenone or empagliflozin alone	• Ratio of change from baseline in eGFR at 30 days<br>• eGFR decline greater than 30% at 30 days from baseline<br>• Ratio of change in eGFR at 180 days and 210 days from Day 30<br>• Proportion of participants with of AKI events<br>• Total number of AKI events<br>• Proportion of participants with hyperkalemia events (moderate hyperkalemia [5.5 <K+ ≤6.0 mmol/L], severe hyperkalemia [K+ >6.0 mmol/L])<br>• Total number of hyperkalemia events (moderate hyperkalemia [5.5 <K+ ≤6.0 mmol/L], severe hyperkalemia [K+ >6.0 mmol/L])<br>• Change from baseline in K+<br>• Proportion of participants with severe hypoglycemia events<br>• Total number of events of severe hypoglycemia events<br>• Proportion of participants with symptomatic hypotension events<br>• Total number of symptomatic hypotension events<br>• Proportion of participants with genital mycotic events<br>• Total number of genital mycotic events<br>• Proportion of participants with ketoacidosis events<br>• Total number of ketoacidosis events<br>• Proportion of participants with necrotizing fasciitis of the perineum events<br>• Total number of necrotizing fasciitis of the perineum events<br>• Proportion of participants with urosepsis and pyelonephritis events<br>• Total number of urosepsis and pyelonephritis events
**Objectives**	**Endpoints and Estimands**
**Other exploratory**	
• To further investigate the study intervention (finerenone, empagliflozin) and similar drugs (e.g., mode-of-action-related effects, safety) and to further investigate pathomechanisms deemed relevant to CV disease, CKD, diabetes, and associated health problems	• Various biomarkers (e.g., diagnostic, safety, pharmacodynamic, monitoring, or potentially predictive biomarkers)
**Other pre-specified**	
• To characterize the PK of finerenone and empagliflozin when given in combination	• PK of finerenone and empagliflozin in plasma ($C_{max,md}$, $AUC_{\tau,md}$) (optional analysis)
Abbreviations: AKI = acute kidney injury; $AUC_{\tau,md}$ = area under the concentration vs. time curve for the expected dosing interval obtained after multiple dose administration; $C_{max,md}$ = maximum drug concentration after multiple dose administration; CV = cardiovascular; CKD = chronic kidney disease; eGFR = estimated glomerular filtration rate; K+ = serum/plasma potassium; PK = pharmacokinetics; UACR = urinary albumin-to-creatinine ratio.	

This study will include adult participants with a clinical diagnosis of T2D and CKD. There are 2 primary endpoints to address the objective of the study; relative change from baseline in UACR at 180 days in combination therapy group versus empagliflozin alone and relative change from baseline in UACR at 180 days in combination therapy group versus finerenone alone. These 2 primary endpoints are not considered as co-primary endpoints.

Overall Design:

- Phase 2, randomized, controlled, double-blind (participants and investigators), double-dummy, multicenter study in participants with CKD and T2D.
- The study will consist of 2 consecutive parts:
    - Part A: participants will be recruited if their estimated glomerular filtration rate (eGFR) is between 40 and 90 ml/min/1.73 m², and they will be equipped with an ambulatory blood pressure monitoring (ABPM) device at Visit 2 for a duration of 24 hours. An interactive web response system (IWRS) will allow capping the number of participants as follows:
        - 80% with an eGFR between ≤75 ml/min/1.73 m²
        - 20% with an eGFR between >75 ml/min/1.73 m².
    - Part B: participants will be recruited if their eGFR is between 30 and 90 ml/min/1.73 m², and they will not have an ABPM. The IWRS will allow capping the number of participants as follows:
        - 80% with an eGFR between ≤75 ml/min/1.73 m²
        - 20% with an eGFR between >75 ml/min/1.73 m².
    - The decision to move from Part A to Part B will be taken by the sponsor and the study's Steering Committee upon feedback from the Data Monitoring Committee (DMC). The safety analysis from the first 50 participants in Part A, as well as their unblinded review by the independent DMC will be used to confirm the enrollment/recruitment start for Part B. This decision shall be effective immediately or after IRB/IEC and/or local Health Authority approval, where applicable. Other inclusion/exclusion criteria or study's schedule or procedure should not be affected.
- Participants will be randomized in a 1:1:1 ratio stratified by eGFR at screening (<60, ≥60 mL/min/1.73m²) and UACR at screening (≤ 850mg/g, > 850 mg/g) in one of the 3 parallel groups:
    - Finerenone (10 or 20 mg [target dose] once daily [OD]) and empagliflozin (10 mg OD)
    - Finerenone (10 or 20 mg OD) and matching placebo to empagliflozin OD
    - Empagliflozin (10 mg OD) and matching placebo to finerenone OD.

  Starting from Day 30, finerenone or placebo doses will be adjusted based on serum/plasma potassium (K+) and eGFR values obtained from local laboratories, at each study visit.
- Participants should be treated for CKD and for T2D according to local treatment guidelines. However, participants must not be exposed to an SGLT2i and/or an MRA within at least 8 weeks prior to screening.

Brief Summary:

The purpose of this study is to assess efficacy and safety of the initial combination therapy using finerenone and empagliflozin compared with either finerenone or empagliflozin alone for reducing UACR in participants with CKD and T2D. Study details include:

Visit Frequency: Screening (up to 2 weeks before randomization), Day 1 (randomization), Days 14 (±2), 30 (±4), 90 (±5) 180 (±5) (end of study intervention), Day 210 (±5; follow-up visit/end of study).

Condition/Disease: Participants with CKD and T2D

Study Hypothesis: Assuming a cumulative effect of both study interventions we hypothesize that in the combination therapy arm, UACR will be 20% lower at 180 days versus the empagliflozin arm, or 20% lower at 180 days versus the finerenone arm.

Health Measurement/Observation: The primary endpoints will be the relative change from baseline in UACR at 180 days in the combination therapy arm versus each of the monotherapy arms.

Number of Participants: With a 50% screening failure rate and a 15% dropout rate, approximately **1,614** participants will be screened to achieve approximately **807** randomly assigned participants to study interventions, for approximately **269** participants per intervention group.

Intervention Groups and Duration:

The total study duration for each participant will be approximately 7.5 months (up to 8.5 months if the optional pre-screening is performed).

At Day 1, participants will be randomized in a 1:1:1 ratio in one of the 3 treatment groups:

- Finerenone (10 or 20 mg OD) and empagliflozin (10 mg OD)
- Finerenone (10 or 20 mg OD) and matching placebo to empagliflozin OD
- Empagliflozin (10 mg OD) and matching placebo to finerenone OD.

Finerenone, or sham placebo, up or down-titrations will be allowed during the study. Up-titration to the target dose of 20 mg will be allowed from Visit 4 onwards, based on eGFR and K+ results. Down-titration will be allowed any time during the study (e.g., between scheduled visits) for safety reasons only. Treatment duration will be approximately 180 days per participant.

Data Monitoring Committee: Yes

1.2 Schema

Figure 1-1: Study Design

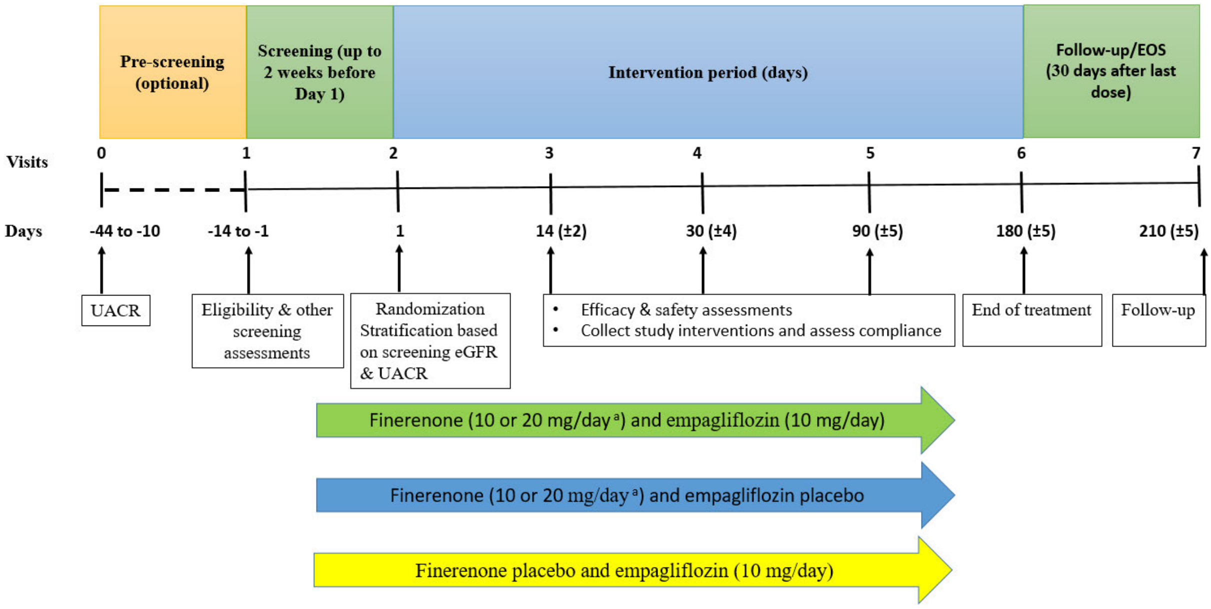

Abbreviations: eGFR = estimated glomerular filtration rate; EOS = end of study; UACR = urinary albumin-to-creatinine ratio.

[a] Up-/down-titration based on eGFR, serum/plasma potassium or potassium, safety, and tolerability.

1.3 Schedule of Activities (SoA)

Table 1-1: Schedule of Activities

Procedure	Pre-Screening[a]	Screening (up to 2 weeks before Day 1)	Intervention Period [Days]					ED	Follow-Up/EOS (30 days after last dose)	Notes
			1[b] (dose)	14±2	30±4	90±5	180±5 (EOT)		210±5	
**Visit**	**0**	**1**	**2**	**3**	**4**	**5**	**6**		**7**	
**Study Day**	**-44 to -10**	**-14 to -6**	**1**	**12-16**	**26-34**	**85-95**	**175-185**		**205-215**	
Pre-screening consent	X									
Informed consent		X								
Inclusion and exclusion criteria		X	X							Re-check clinical status before randomization
Demography		X								
Complete physical examination including height[c] and weight		X	X	X	X	X	X	X	X	
Vital signs		X	X	X	X	X	X	X	X	
Medical history		X								
Serum pregnancy test (WOCBP only)		X			X	X	X	X		To be performed by central lab
FSH test (to confirm postmenopausal status, when needed)[d]		X								
Central laboratory tests										
UACR (urinary creatinine, albumin)[e,f]		X[i]	X	X	X	X	X	X	X	
Serum K+[e]		X[i]	X	X	X	X	X	X	X	
eGFR (serum creatinine)[e]		X[i]	X	X	X	X	X	X	X	
Safety laboratory tests		X	X	X	X	X	X	X	X	Clinical chemistry, hematology, urinalysis. HDL, LDL-C, total

Table 1-1: Schedule of Activities

Procedure	Pre-Screening[a]	Screening (up to 2 weeks before Day 1)	Intervention Period [Days]					ED	Follow-Up/EOS (30 days after last dose)	Notes
			1[b] (dose)	14±2	30±4	90±5	180±5 (EOT)		210±5	
**Visit**	**0**	**1**	**2**	**3**	**4**	**5**	**6**		**7**	
**Study Day**	**-44 to -10**	**-14 to -6**	**1**	**12-16**	**26-34**	**85-95**	**175-185**		**205-215**	
										cholesterol and triglycerides will be measured at screening only
Local laboratory tests[g]										
UACR [f]	X									Urine containers to be supplied by local lab
Serum/plasma K+[h]			X[j]	X[k]	X[k]	X[k]	X[k]	X[k]	X[k]	At Visit 2 in case the participant is deemed not eligible based on K+, retest is allowed within 24 hours
eGFR (serum creatinine)[h]			X[j]	X[k]	X[k]	X[k]	X[k]	X[k]	X[k]	At Visit 2 in case the participant is deemed not eligible based on eGFR, retest is allowed within 24 hours
PK[l,m]				X	X	X	X	X		
Biomarkers (blood) [l]		X	X		X		X	X		Day 1 sample to be collected prior to start of treatment
Biomarker samples (first morning void urine)			X		X		X	X		
12-lead ECG		X	X				X			

Table 1-1: Schedule of Activities

Procedure	Pre-Screening[a]	Screening (up to 2 weeks before Day 1)	Intervention Period [Days]					ED	Follow-Up/EOS (30 days after last dose)	Notes
			1[b] (dose)	14±2	30±4	90±5	180±5 (EOT)		210±5	
**Visit**	**0**	**1**	**2**	**3**	**4**	**5**	**6**		**7**	
**Study Day**	**-44 to -10**	**-14 to -6**	**1**	**12-16**	**26-34**	**85-95**	**175-185**		**205-215**	
Randomization			X							
Dispense urine containers for UACR assessments		X	X	X	X	X	X			
Study intervention dispensation/accountability			X[n]		X	X	X[o]			
24-hour ambulatory blood pressure monitoring			X[n]							
Dose adjustment (with unscheduled visits)[p]					X	X				Up-/down-titration, restart after interruption
Adverse events	X	X	←==→							
Prior/Concomitant medication review		X	←==→							

Abbreviations: ECG = electrocardiogram; ED = early discontinuation; EOS = end of study; EOT = end of treatment; eGFR = estimated glomerular filtration rate; HDL = high density lipoprotein; LDL-C = low density lipoprotein cholesterol; K+ = serum/plasma potassium; PK = pharmacokinetics; UACR = urinary albumin-to-creatinine ratio; WOCBP = woman of childbearing potential.

a) Pre-screening visit is optional (up to 4 weeks before screening).
b) Day 1 = randomization/baseline visit.
c) Height will be measured at screening only.
d) May be used to confirm a postmenopausal state in women not using hormonal contraception or HRT. In the absence of 12 months of amenorrhea, confirmation with more than one FSH measurement is required.
e) Central laboratory values to be used for endpoint statistical analyses.
f) At the optional pre-screening and at Visit 1, first morning void urine samples to be collected on 3 consecutive days at the participant's home. At the other visits, the 2 consecutive samples can be collected ±7 days from the visit date.
g) Local laboratory values for safety purposes.
h) Blood samples (for K+ and creatinine [eGFR]) for measurement in the local laboratory may be obtained up to 72 hours before a scheduled visit.
i) One re-assessment (central lab) of eGFR, K+ and/or UACR is allowed at the screening visit.
j) K+, and eGFR values obtained from local laboratory will be used to randomize participants at Day 1.
k) K+ and eGFR values obtained from local laboratory will be used for finerenone up-/down-titration as well as monitoring after down/up-titration and restart.
l) Refer to sampling handling sheets or laboratory manual for sample handling.
m) At Visits 3 and 6, trough (i.e. pre-dose) samples for the determination of finerenone and empagliflozin plasma concentrations will be drawn before intake of study intervention. At this visit, study intervention will be administered at the study site and the exact time of study intervention intake on the day before the visit and on the day of the visit and the exact sampling time will be recorded in the electronic case report form. At Visits 4 and 5 (and ED, if applicable), post-dose blood samples for the determination of finerenone and empagliflozin plasma concentrations will be drawn during the visit, 1.5-10 hours after study intervention intake at home. Note: At all mentioned visits, samples should be taken even if the study interventions were not taken as indicated. In such cases, particular attention will be paid to properly document the actual time of study intervention intake before sampling. These samples will not be analyzed directly but may be analyzed in case of specific questions from the DMC, Steering Committee, or sponsor.
n) First study intervention at study site. The participant from Part A will remain 4 to 6 hours at the study site for office blood pressure monitoring and will then be equipped for 24 hours with an ambulatory blood pressure monitoring device.
o) Accountability only
p) Subsequent to an up-titration or restart of study drug after interruption of finerenone intake for more than 7 days, the investigator should perform an unscheduled visit, 4 weeks (±7 days) after titration or restart, in order to monitor K+ levels and eGFR.

2. Introduction

Finerenone (Kerendia®) is a novel, nonsteroidal, potent and selective mineralocorticoid receptor antagonist (MRA), recently approved by the Food and Drug Administration (FDA) to reduce the risk of sustained estimated glomerular filtration rate (eGFR) decline, end-stage kidney disease (ESKD), cardiovascular (CV) death, non-fatal myocardial infarction (MI), and hospitalization for heart failure (HF) in adult patients with chronic kidney disease (CKD) associated with type 2 diabetes (T2D).

Empagliflozin (Jardiance®) is a sodium/glucose cotransporter-2 inhibitor (SGLT2i) initially marketed to treat T2D and which has shown to prevent major adverse CV events in T2D patients. Other SGLT2i have also shown benefit in patients with CKD, while empagliflozin is still under investigation in this population.

2.1 Study Rationale

Finerenone is an MRA acting by targeting inflammatory and fibrotic pathways mediated by mineralocorticoid receptor (MR) over-activation in the heart and kidney (Kolkhof et al. 2017). In FIDELIO-DKD, finerenone protected from kidney function decline and reduced the risk of CV events in patients with CKD and T2D (Bakris et al. 2020; Filippatos et al. 2021). Finerenone also significantly reduced urinary albumin-to-creatinine ratio (UACR) compared to placebo at 4 months.

SGLT2i are indicated for the treatment of T2D and some of them are also indicated for the treatment of HF and CKD. Clinical studies in CKD patients showed that SGLT2i reduce the risk of kidney failure and CV events, regardless of the presence or absence of diabetes (Perkovic et al. 2019; Heerspink et al. 2020). Similarly, SGLT2i lower the risk of CV death or hospitalization for HF, regardless of the presence or absence of diabetes, in patients with HF (Packer et al. 2020).

Combining these 2 drugs could be interesting for the reasons explained hereafter.

Data from sub-analyses of trials in HF (McMurray et al. 2019) suggest that the usage of SGLT2i in patients already taking a steroidal MRA could reduce the incidence of hyperkalemia in this patient population. This finding has been replicated in an analysis of the DAPA-HF study (Shen et al. 2021). In a post-hoc analysis of FIDELIO-DKD, patients treated with finerenone and SGLT2i had fewer hyperkalemia events than those receiving finerenone alone (Rossing et al. 2021).

Efficacy of finerenone on CV outcomes was observed independent of the presence or absence of SGLT2i use at baseline. In a recent preclinical study on non-diabetic mice model, it has been shown that empagliflozin and finerenone have an additive effect on urinary protein-to-creatinine ratio (Kolkhof et al. 2021).

Regarding UACR, a greater improvement was observed in finerenone patients receiving SGLT2i at baseline: reduction in UACR with finerenone was seen without SGLT-2i use (ratio of LS-means 0.68, 0.65-0.71; $p<0.0001$) and on top of SGLT-2is at baseline (ratio of LS-means 0.75, 0.62-0.90; $p=0.0024$) (Rossing et al. 2021).
It is then hypothesized that the dual treatment effects of finerenone combined with an SGLT2i will have additive renoprotective benefits in CKD patients with T2D.
Elevated UACR is a key risk marker for CKD and cardiovascular disorders (Cohen et al. 2021), more particularly in patients with high albuminuria (Heerspink et al. 2019).

This study will prospectively expand on these observations by being the first parallel-group randomized controlled trial to evaluate the safety, tolerability, and additive efficacy of the combined use of finerenone and empagliflozin using the surrogate endpoint of UACR. This study will also investigate, for the first time, simultaneous initiation of finerenone and empagliflozin. The goal of this study is to show that the combined use of finerenone and empagliflozin is superior to either empagliflozin alone, or finerenone alone in reducing UACR at 180 days.

2.2 Background

CKD is defined as abnormalities of kidney structure or function, with either of the following present for at least 3 months (KDIGO 2013):

Markers of kidney damage	Albumin:creatinine ratio (ACR) ≥30 mg/g<br>Urine sediment abnormalities (e.g., hematuria, red cell casts, etc.)<br>Electrolyte and other abnormalities due to tubular disorders<br>Abnormalities detected by histology<br>Structural abnormalities detected by imaging<br>History of kidney transplantation
Decreased glomerular filtration rate	Glomerular filtration rate <60 mL/min/1.73 m^2

In 2017, 697.5 million cases of all-stage CKD were recorded accounting for a global prevalence of 9.1%. Globally, 4.6% of total mortality were caused by deaths due to CKD and CV disease attributable to impaired kidney function (GBD Chronic Kidney Disease Collaboration 2020).

CKD in patients with T2D is the most frequent cause of ESKD in western countries (Fernandez et al. 2012). In addition, the risk of CV disease and death increases in diabetic patients with CKD with decreasing glomerular filtration rate (GFR) and increasing albuminuria levels (Matsushita et al. 2010; KDIGO 2013).

As the T2D population rapidly grows throughout the world within the next years (International Diabetes Federation 2017), and with it the CKD population, there is an increasing need for new therapeutic agents that effectively target underlying disease mechanisms and slow or halt the progression of kidney disease, while also addressing the high CV morbidity and mortality in this population.

Finerenone is a nonsteroidal, selective antagonist of the MR that potently attenuates inflammation and fibrosis mediated by MR over-activation. The MR is expressed in the kidneys, heart, and blood vessels where finerenone also counteracts sodium retention and hypertrophic processes. Finerenone has a high potency and selectivity for the MR due to its nonsteroidal structure and bulky binding mode. Finerenone has no relevant affinity for androgen, progesterone, estrogen, and glucocorticoid receptors and therefore does not cause sex hormone-related adverse events (AEs; e.g., gynecomastia). Its binding to the MR leads to a specific receptor ligand complex that blocks recruitment of transcriptional coactivators implicated in the expression of pro-inflammatory and pro-fibrotic mediators (Agarwal et al. 2020; Agarwal et al. 2021).

During the phase 2 and 3 trials, it has been shown that:

- Finerenone reduced albuminuria in diabetic and non-diabetic patients with HF with reduced ejection fraction (HFrEF) and CKD and that it is at least as effective as

spironolactone in decreasing plasma N-terminal prohormone B-type natriuretic peptide (NT-proBNP) and UACR (Pitt et al. 2013).

- Among patients with T2D and UACR, ≥30 mg/g on stable therapy with an angiotensin-converting enzyme inhibitor (ACEi) or angiotensin receptor blocker (ARB), finerenone (10, 15, and 20 mg once daily [OD]) demonstrated a dose-dependent placebo-subtracted reduction in UACR of 25% to 38% over 90 days. Minimal effects on potassium (K+) and renal function and a limited effect on reducing blood pressure (BP) were observed (Bakris et al. 2015).
- 5,734 patients with CKD and T2D were further enrolled in the FIDELIO-DKD study. In this study, finerenone significantly lowered the risk of CKD progression and CV events compared to placebo. In terms of safety, the incidence of AEs was similar in both finerenone and placebo groups but higher mean K+ levels were consistently observed in the finerenone group. Finerenone also had a modest effect on BP with changes in mean systolic BP from baseline to month 1 and to month 12 of -3.0 and -2.1 mm Hg, respectively (Bakris et al. 2020).
- 7,437 patients with CKD and T2D were randomized in the FIGARO-DKD study. Among patients with T2D and stage 2 to 4 CKD with moderately elevated albuminuria or stage 1 or 2 CKD with severely elevated albuminuria, finerenone therapy improved CV outcomes as compared with placebo. The overall frequency of AEs did not differ substantially between groups. The incidence of hyperkalemia-related discontinuation of the trial regimen was higher with finerenone (1.2%) than with placebo (0.4%) (Pitt et al. 2021).

A detailed description of the chemistry, pharmacology, efficacy, and safety of finerenone is provided in the Investigator's Brochure (IB). Finerenone is currently being evaluated by several Healthcare Authorities for a marketing authorization and has been approved in the United States of America (US).

SGLT2i is highly expressed in the kidney and is responsible, as the predominant transporter, for the reabsorption of glucose from the glomerular filtrate back into the circulation. Empagliflozin acts by reducing renal glucose reabsorption. Empagliflozin is prescribed either as a monotherapy when metformin is considered inappropriate due to intolerance or as an adjunct therapy to other diabetes treatments. Clinical data from the EMPA-REG OUTCOME trial suggested that the treatment of T2D and atherosclerotic CV patients with empagliflozin reduced the risk of CV events and all-cause hospitalization (McGuire et al. 2020) and led to Jardiance® label extension. As well, in patients with T2D at higher CV risk, empagliflozin was associated with slower progression of kidney disease and lower rates of renal complications (Wanner et al. 2020). In the CVD-REAL 3, real-world clinical study, empagliflozin was used in 34% of patients and associated with a lower rate of eGFR decline and reduced risk of composite endpoint of 50% eGFR decline (Heerspink et al. 2020). These findings strongly support the potential therapeutic use of empagliflozin for the reduction of CKD in T2D patients.

A summary of the pharmacology, efficacy, and safety of empagliflozin can be found in the European Summary of Product Characteristics (EU SmPC).

2.3 Benefit/Risk Assessment

Relevant emerging safety data, e.g., serious AEs (SAEs), suspected unexpected serious adverse reactions (SUSARs), and serious safety-related protocol deviations, will be communicated as soon as possible between the sponsor, all study sites, and investigators and trial participants.

2.3.1 Risk Assessment

Finerenone has a favorable benefit-risk profile. Adverse reactions reported with finerenone include hyperkalemia, hyponatremia, hypotension (mean systolic BP [SBP] decrease of 3 mmHg and mean diastolic BP [DBP] decrease by 1 to 2 mmHg after 4 weeks of treatment) and GFR decreased (mean decrease in eGFR of 2 mL/min/1.73 m^2 within the first 4 weeks). K+, BP, and eGFR will be monitored at each study visit.

Regarding hyperkalemia, Rossing et al. (2021) found that patients treated with finerenone and SGLT2i had fewer hyperkalemia events than those receiving finerenone alone, in a post-hoc analysis of FIDELIO-DKD. However, to minimize safety risks to the participants, finerenone treatment will be initiated only if K+ is less than or equal to 4.8 mmol/L at screening and the starting dose of finerenone will be chosen according to baseline eGFR. Subsequent titration will be performed on the basis of measured K+ and eGFR values. Stopping rules for temporary and permanent discontinuation or dose reduction of finerenone based on K+ values will minimize the risk of hyperkalemia. At any time during the study, the investigator has the option to also down-titrate finerenone, depending on K+ and the progression of the underlying disease (see Section 6.5). More detailed information about finerenone may be found in the IB.

The most commonly reported AEs for empagliflozin are hypoglycemia with concomitant use with insulin and insulin secretagogues, volume depletion, genital mycotic infections, thirst, constipation, pruritus, increased urination, and serum lipids increased. Special warnings and precautions for use include ketoacidosis, volume depletion, urosepsis and pyelonephritis, and necrotizing fasciitis of the perineum (Fournier's Gangrene). Guidance on ketoacidosis management will be provided to the investigators (see Section 6.8.6). Empagliflozin is associated with an initial fall in BP and eGFR (mean eGFR decrease of 3 ml/min/1.73 m^2 [EU SmPC]). In the US, empagliflozin can be administered to patients with eGFR ≥30 ml/min/1.73 m^2 when it is not recommended for patients with an eGFR below 45 ml/min/1.73 m2 in Europe. However, in the EMPA-REG OUTCOME trial, empagliflozin has been shown to reduce CV death in patients with T2D at high risk for CV events, irrespective of the baseline eGFR (down to 30 ml/min/1.73m^2) or KDIGO risk category (Zinman et al. 2015; Levin et al 2020). In addition, empagliflozin is currently being investigated in patients with CKD, with baseline eGFR as low as 20 ml/min/1.73m^2, in the EMPA-KIDNEY study (NCT03594110). Of note, American Diabetes Association (ADA) recommends the use of a SGLT2i in patients with an eGFR ≥30 mL/min/1.73 m^2 and urinary albumin >300 mg/g creatinine for patients with CKD and T2D (ADA 2021).

As noted above, both finerenone and empaglifozin treatment are associated with an initial drop of BP and decrease in eGFR (2 mL/min/1.73 m^2 and 3 mL/min/1.73 m^2, respectively). It is unknown what the effect of concomitant treatment with both products will be on these parameters. However, a subgroup analysis was performed in the FIDELIO-DKD study, including participants concomitantly treated with SGLT2i (6.6% in the finerenone arm and 7.6% in the placebo arm; data on file). Results for this subgroup analysis were consistent with the overall analysis in terms of AEs of worsening of kidney function, associated or not with hospitalization.

CONFIDENCE will be the first controlled clinical trial where finerenone and empagliflozin are simultaneously initiated. To minimize the risk to the participants, the study will consist of 2 consecutive parts:

- Part A: participants will be recruited if their eGFR is between 40 and 90 ml/min/1.73 m^2, and they will be equipped with an ambulatory BP monitoring (ABPM) device, 1 hour before the first intake at the study site. The participant will remain 4 to 6 hours at the study site for office BP monitoring and will keep the ABPM for 24 hours.
- Part B: participants will be recruited if their eGFR is between 30 and 90 ml/min/1.73 m^2, and they will not have an ABPM.
- The decision to move from Part A to Part B will be taken by the sponsor and the study's Steering Committee (SC) upon feedback from the Data Monitoring Committee (DMC). The safety analysis from the first 50 participants in Part A, as well as their unblinded review by the independent DMC will be used to confirm the enrollment/recruitment start for Part B.

eGFR will be monitored at each study visit with a first assessment 14 days after the initial intake. In addition, before inclusion of participants with eGFR as low as 30 ml/min/1.73 m^2, data from the first 50 study participants will be carefully evaluated by the independent DMC.

There is a risk of a severe acute respiratory syndrome coronavirus 2 (SARS-CoV-2) infection for study participants as long as the coronavirus disease 2019 (COVID-19) pandemic situation is ongoing. To minimize their infection risk during study participation, the investigators/sites will follow all recommendations issued by local authorities and guidelines aiming to reduce the risk of disease spreading. Details on the measures are specified by the site and agreed with the sponsor.

As part of the study procedures, participants will be closely monitored (including for signs of COVID-19) during the entire study duration. A pre-visit call before each in-person study visit is recommended to confirm the study participant does not have any symptoms suspicious for a SARS-CoV-2 infection.

Measures which prioritize participant safety and data validity are implemented. In case these 2 objectives conflict, participant safety always prevails.

During a pandemic situation, further measures according to recommendations and requirements from local health authorities may become necessary. These will be followed within the context of this study as far as applicable.

2.3.2 Benefit Assessment

Among patients with diabetes, those with kidney disease are consistently observed to have substantially elevated mortality rates. Most of this mortality is due to CV disease (CVD) with up to a 3-fold increase in 10-year CV mortality (Afkarian et al. 2013), although non-CV mortality is also increased. Decreased eGFR is associated with increased risks of CV events, CV mortality, and all-cause mortality (Tuttle et al. 2014; Matsushita et al. 2010; Go et al. 2004). There is a high unmet medical need for treatments that can reduce the extensive burden of CV mortality and morbidity and the progression of kidney disease in diabetic patients with CKD.

CKD in patients with T2D is the most frequent cause of ESKD in western countries (Fernandez et al. 2012). It is then important to slow down CKD progression at early stages to

prevent the development of ESKD and subsequent need for renal replacement therapy (GBD Chronic Kidney Disease Collaboration 2020).

Reduction in UACR, a surrogate marker for renal outcomes, is correlated with improved renal and cardiovascular outcomes. Therefore, early and efficient intervention assessed by UACR reduction might provide long-term benefits for patients with CKD and T2D. By taking part in this study, participants treated with finerenone and empagliflozin combination could benefit from a more effective treatment to reduce CKD progression together with decreased risk of adverse drug reactions.

Participants enrolled in the monotherapy arms could benefit of either finerenone or empagliflozin and may benefit from closer follow-up compared to real-world clinical practice. More detailed information about the known and expected benefits of both study interventions may be found in the IB for finerenone and in the EU SmPC for empagliflozin. All protocol-related procedures, including vital signs measurements, 12-lead electrocardiograms (ECGs), blood and urine sampling, are non-invasive or established routine assessments in the management of patients with CKD with T2D.

2.3.3 Overall Benefit: Risk Conclusion

Finerenone successfully delayed the onset of the composite of kidney failure, sustained eGFR decrease of ≥40% from baseline for at least 4 weeks and renal death in the phase 3 FIDELIO-DKD. At 3 years a chronic eGFR slope difference of 1.37 mL/min/1.73m^2 per year was seen, reflecting the long-term preservation of renal function with finerenone. An increased risk of hyperkalemia was observed with finerenone in a population with advanced CKD and T2D; however, the majority of these events were non-serious and the number of events leading to clinical consequences was low.

The main risk of finerenone treatment, hyperkalemia, has been proven to be manageable and efficacy was also consistent in participants at higher risk of hyperkalemia (i.e., with lower eGFR, higher baseline $K+$). Mitigation measures will also be in place in CONFIDENCE.

Based on the available data and the high medical need in the studied population, inclusion of participants into this study is considered favorable.

To date, empagliflozin is approved for the treatment of T2D, and to prevent CV events in this population. It is currently explored in a clinical trial in patients with CKD (NCT03594110).

The main risk of empagliflozin treatment are ketoacidosis, volume depletion, urosepsis and pyelonephritis, hypoglycemia with concomitant use with insulin and insulin secretagogues, necrotizing fasciitis of the perineum (Fournier's Gangrene), genital mycotic infections, and hypersensitivity reactions. Empagliflozin is associated with an initial fall in BP and eGFR (mean eGFR decrease of 3 ml/min/1.73 m^2).

CONFIDENCE will be the first controlled clinical trial where finerenone and empagliflozin are simultaneously initiated. As mentioned above, initiation of each single drug can lead to a decrease in BP and eGFR. The additive effect of both drugs on BP and eGFR is not yet known. To monitor the risk of hypotension, all participants will be equipped with an ABPM device 1 hour before the first intake at the study site. The participant will remain 4 to 6 hours at the study site for office BP monitoring and will keep the ABPM for 24 hours. eGFR will be monitored at each study visit with a first assessment 14 days after the initial intake. With regards to risks specific to empagliflozin, participants with risks factors for ketoacidosis or volume depletion will be excluded from the study. Participants will also be monitored to look

for signs and symptoms of ketoacidosis. Concomitant therapy with insulin will also be carefully monitored and assessed during the medical review.

Reduction in UACR, a surrogate marker for renal outcomes, is correlated with improved renal and cardiovascular outcomes. Therefore, early and efficient intervention, resulting in slower disease progression and potentially prevention of disease progression, may provide long-term benefits for patients with CKD and T2D. By taking part in this study, participants treated with finerenone and empagliflozin combination could benefit from a more effective treatment to reduce CKD progression together with decreased risk of adverse drug reactions.

The clinical study will start after the consent of the Ethics Committees and the permission of the Competent Authorities have been obtained. All human investigations will be done in accordance to local law, Good Clinical Practice (GCP), and to the declaration of Helsinki (1964; last revised in 2013).

3. Objectives and Endpoints and Estimands

Objectives	Endpoints and Estimands
**Primary**	
• To demonstrate that combination therapy using finerenone and empagliflozin is superior in reducing UACR than either empagliflozin or finerenone alone	Primary Endpoints:<br>• Relative change from baseline in UACR at 180 days in combination therapy group versus empagliflozin alone<br>or<br>• Relative change from baseline in UACR at 180 days in combination therapy group versus finerenone alone
	Summary Measures:<br>• Mean ratio of change from baseline to Day 180 in UACR for the combination therapy group, to empagliflozin alone<br>• Mean ratio of change from baseline to Day 180 in UACR for the combination therapy group, to finerenone alone
**Secondary**	
• To further investigate the efficacy of combination therapy using finerenone and empagliflozin versus either finerenone or empagliflozin alone	• Relative change in UACR between end of treatment visit and 30 days after end of treatment visit<br>• Relative change in UACR between 30 days after end of treatment visit and baseline<br>• Relative change in UACR category (>30%, >40%, >50%) at 180 days

• To evaluate the safety of combination therapy using finerenone and empagliflozin versus either finerenone or empagliflozin alone	• Ratio of change from baseline in eGFR at 30 days<br>• eGFR decline greater than 30% at 30 days from baseline<br>• Ratio of change in eGFR at 180 days and 210 days from day 30<br>• Proportion of participants with of AKI events<br>• Total number of AKI events<br>• Proportion of participants with hyperkalemia events (moderate hyperkalemia [5.5 <K+ ≤6.0 mmol/L], severe hyperkalemia [K+ >6.0 mmol/L])<br>• Total number of hyperkalemia events (moderate hyperkalemia [5.5 <K+ ≤6.0 mmol/L], severe hyperkalemia [K+ >6.0 mmol/L])<br>• Change from baseline in K+<br>• Proportion of participants with severe hypoglycemia events<br>• Total number of events of severe hypoglycemia events<br>• Proportion of participants with symptomatic hypotension events<br>• Total number of symptomatic hypotension events<br>• Proportion of participants with genital mycotic events<br>• Total number of genital mycotic events<br>• Proportion of participants with ketoacidosis events<br>• Total number of ketoacidosis events<br>• Proportion of participants with necrotizing fasciitis of the perineum events<br>• Total number of necrotizing fasciitis of the perineum events<br>• Proportion of participants with urosepsis and pyelonephritis events<br>• Total number of urosepsis and pyelonephritis events
**Other exploratory**	
• To further investigate the study intervention (finerenone, empagliflozin) and similar drugs (e.g., mode-of-action-related effects, safety) and to further investigate pathomechanisms deemed relevant to CV disease, CKD, diabetes, and associated health problems	• Various biomarkers (e.g., diagnostic, safety, pharmacodynamic, monitoring, or potentially predictive biomarkers)
**Other pre-specified**	
• To characterize the PK of finerenone and empagliflozin when given in combination	• PK of finerenone and empagliflozin in plasma ($C_{max,md}$, $AUC_{\tau,md}$) (optional analysis)

Abbreviations: AKI = acute kidney injury; $AUC_{\tau,md}$ = area under the concentration vs. time curve for the expected dosing interval obtained after multiple dose administration; $C_{max,md}$ = maximum drug concentration after multiple dose administration; CV = cardiovascular; CKD = chronic kidney disease; eGFR = estimated glomerular filtration rate; K+ = serum/plasma potassium; PK = pharmacokinetics; UACR = urinary albumin-to-creatinine ratio.

There are 2 primary endpoints to address the objective of the study; relative change from baseline in UACR at 180 days in combination therapy group versus empagliflozin alone and

relative change from baseline in UACR at 180 days in combination therapy group versus finerenone alone. These 2 primary endpoints are not considered as co-primary endpoints.

Primary Analysis Estimand

The assessment of efficacy will be done in the estimand framework, where the primary objective of the study is to assess the effect of combination therapy on the percentage reduction in UACR from baseline to Day 180 compared to its individual components if the treatment is taken according to the instructions as given in the protocol.

The estimand for assessing this study objective is defined by the following characteristics:

- *Population*: Adult participants with a clinical diagnosis of CKD and T2D.
- *Variable*: Ratio of change from baseline to Day 180 in UACR for the combination therapy group, to empagliflozin alone or finerenone alone.
- *Treatment*: Either finerenone (10 or 20 mg OD) and empagliflozin (10 mg OD) or finerenone (10 or 20 mg OD) or empagliflozin (10 mg OD), on top of standard of care (SoC) (treatment policy).
- *Summary measure*: Mean ratio of change from baseline to Day 180 in UACR for the combination therapy group, to empagliflozin alone or finerenone alone.
- *Intercurrent events*: the 3 most important intercurrent events are

  <u>*Treatment discontinuation*</u>

  - Treatment interruption due to reasons related to the treatment, such as hyperkalemia, will be handled using *treatment policy*

  <u>*Dialysis/kidney transplantation*</u>

  - Dialysis will be handled using a *hypothetical strategy*
  - Kidney transplantation will be handled using a *hypothetical strategy*

  <u>*Death*</u>

  - Death will be handled using *a hypothetical strategy.*

Hypothetical strategy relates to envisioning a scenario where the intercurrent event did not occur. Sensitivity analyses will be performed to observe scenarios in which the intercurrent events are handled differently.

Treatment policy strategy relates to participants being followed up for the remainder of the study after discontinuing treatment and data and follow-up time after discontinuation of treatment will be included in the analysis.

4. Study Design

4.1 Overall Design

- Phase 2, randomized, controlled, double-blind (participants and investigators), double-dummy, multicenter study in participants with CKD and T2D.
- Assuming a screening failure rate of approximately 50%, **1,614** participants will have to be screened to achieve **807** randomly assigned participants to study intervention (approximately **269** participants per group).

- The study will consist of 2 consecutive parts:
  - Part A: participants will be recruited if their eGFR is between 40 and 90 ml/min/1.73 m², and they will be equipped with an ABPM at Visit 2 for a duration of 24 hours. An interactive web response system (IWRS) will allow capping the number of participants as follows:
    - 80% with an eGFR between ≤75 ml/min/1.73 m²
    - 20% with an eGFR between >75 ml/min/1.73 m².
  - Part B: participants will be recruited if their eGFR is between 30 and 90 ml/min/1.73 m², and they will not have an ABPM. The IWRS will allow capping the number of participants as follows:
    - 80% with an eGFR between ≤75 ml/min/1.73 m²
    - 20% with an eGFR between >75 ml/min/1.73 m².
  - The decision to move from Part A to Part B will be taken by the sponsor and the study's SC upon feedback from the DMC. The safety analysis from the first 50 participants in Part A, as well as their unblinded review by the independent DMC will be used to confirm the enrollment/recruitment start for Part B. This decision shall be effective immediately or after IRB/IEC and/or local Health Authority approval, where applicable.
  - Other inclusion/exclusion criteria or study's schedule or procedure should not be affected.
- Participants will be randomized in a 1:1:1 ratio stratified by eGFR at screening (<60, ≥60 mL/min/1.73m²) and UACR (≤ 850mg/g, > 850 mg/g), using the baseline median from FIDELIO-DKD study) in one of the 3 parallel groups:
  - Finerenone (10 or 20 mg once daily [OD]) and empagliflozin (10 mg OD)
  - Finerenone (10 or 20 mg OD) and matching placebo to empagliflozin (OD)
  - Empagliflozin (10 mg OD) and matching placebo to finerenone (OD).
- The starting dose of finerenone will depend on the participant's eGFR level at the screening visit: a lower dose of 10 mg OD if eGFR is between <60 mL/min/1.73m², or the higher (target) dose of 20 mg OD if eGFR is ≥60 mL/min/1.73m².
- Finerenone dose will be defined and further adjusted based on K+ and eGFR values obtained from local laboratories, at each study visit. Necessary blood samples may be obtained up to 72 hours before a scheduled visit. Finerenone dose will also be adjusted, at investigator discretion based on any safety and tolerability concern.
- Participants should be treated for CKD and T2D according to local treatment guidelines. However, participants must not be exposed to an SGLT2i and/or a MRA within at least 8 weeks prior to screening. Participants should also be treated with the clinically maximum tolerated dose, as per investigator judgment, of ACEi or ARB, but not both, for more than 1 month at screening visit.
- An DMC will review safety data during the study (see Section 10.1.5.1).
- The total study duration for each participant will be approximately 7.5 months (up to 8.5 months if the optional pre-screening is performed):
  - A screening visit will occur up to 2 weeks before randomization, during which eligibility criteria will be checked. Eligibility criteria related to laboratory evaluation (e.g., UACR) will be assessed once results are available. <u>Note</u>: Sites

will also have the option of a pre-screening visit to examine UACR. This UACR value will not be valid to determine eligibility and will only be used to assess whether the participant may progress to a formal screening visit.

- Randomization will occur at Day 1 (baseline). Following visits will occur at Days 14 (±2), 30 (±4), 90 (±5), and 180 (±5; last day of intervention period). A follow-up/end of study (EOS) visit will be scheduled, 30 days after last dose (Day 210 ±5).
- Note: Following-titration or restart of study drug after interruption of finerenone intake for more than 7 days, the investigator will have to perform an unscheduled visit, 4 weeks (±7 days) after titration or restart, in order to monitor K+ levels and eGFR.

- The period between the participant's last intake of study intervention and last visit in the study is referred to as the 'follow-up period'. If a participant withdraws from study intervention permanently but does not withdraw from the study, this would apply to the period between the early discontinuation (ED) visit, which should take place as soon as possible following permanent discontinuation of study intervention, and the follow-up/EOS visit. In this case, the follow-up period will also last approximately 30 days.
- It is planned that all randomized participants will remain in the study unless one of the following occurs: consent withdrawal, ED of the study either by the sponsor or at the recommendation of the independent DMC (see Appendix 1).

4.2 Scientific Rationale for Study Design

This is a phase 2, randomized, controlled, double-blind, double-dummy, multicenter study to investigate the efficacy and safety of the finerenone and empagliflozin combination compared to respective monotherapies in reducing UACR in participants with T2D and CKD.

Participants and investigators will be blinded to study intervention allocation ensuring a double-blind design, thus limiting bias.

The primary surrogate endpoints will be relative change from baseline at 180 days of UACR in finerenone plus empagliflozin arm versus each of the monotherapies. UACR is a measurement of albuminuria, a predictor of long-term renal and CV adverse outcomes in T2D patients (Fox 2012; Heerspink et al. 2019). Post-hoc analyses of randomized clinical trials (Heerspink et al. 2014; Levey et al. 2019) suggest that a meaningful reduction in albuminuria may translate to protection from CV events and declining renal function in patients with diabetic and non-diabetic renal disease with albuminuria. For every 30% reduction in albuminuria, one can expect the risk of ESKD to decreased by 23.7% (95% confidence interval [CI], 11.4% to 34.2%; p=0.001) (Heerspink et al. 2019). Changes in UACR can be detected early in response to treatment. Therefore, change in UACR at Day 180 is considered as an appropriate endpoint.

The study aims at showing an additive effect on UACR when finerenone and empagliflozin are taken together. The monotherapy arms will allow the comparison between the combination and each monotherapy to demonstrate this additive effect.

Given that this is the first time that the simultaneous initiation of finerenone and a SGLT2i is being investigated, the sponsor made the decision to protect the participants' safety by implementing a higher threshold for eGFR at entry in the study, combined with an ABPM. These measures are implemented to address the risks of AKI and symptomatic hypotension.

Upon unblinded data review by the DMC, these measures shall be relieved and subsequently lead to the second phase of the trial with a lower eGFR threshold. The eGFR eligibility criteria of 30 mL/min/1.73m² is based on the lowest eGFR threshold recommended by current guidelines on the introduction of empagliflozin in this patient population.

4.2.1 Participant's Input into Design

Participants were not involved in the design of this study.

4.3 Justification for Dose

4.3.1 Finerenone

Initiation of finerenone treatment is recommended when K+ ≤4.8 mmol/L (see IB). Participants with K+ above 4.8 mmol/L at screening will not be enrolled in the study (see Section 5.2).

The starting dose of finerenone will be:

- 20 mg OD if eGFR ≥60 mL/min/1.73 m^2
- 10 mg OD if eGFR <60 mL/min/1.73 m^2.

The starting dose will be established based on screening eGFR results, obtained from the central laboratory (see Section 8.2.4).

Up and down-titrations will be allowed during the study and will be based on local laboratory results (see Section 6.5).

4.3.2 Empagliflozin

In EU SmPC, the recommended dose of Jardiance is 10 mg once daily, taken with or without food. The up-titration to 25 mg is only recommended for glucose control, which is not the aim of the study.

In 2016, Cherney et al. conducted a pooled analysis on the effect of empagliflozin on UACR in T2D patients with either microalbuminuria (30-300 mg/g) or macroalbuminuria (>300 mg/g), enrolled in 1 of the 5 phase 3 trials with primary endpoints related to glucose lowering (Cherney et al. 2016). In 4 studies, patients with eGFR ≥30 ml/min/1.73 m^2 were randomized to receive empagliflozin 10 mg, empagliflozin 25 mg, or placebo. In the fifth study (EMPA-REG RENAL), which included individuals with CKD and T2D, patients with CKD stage 2 received empagliflozin 10 mg, empagliflozin 25 mg, or placebo, and patients with CKD stage 3 or stage 4 received empagliflozin 25 mg or placebo. The authors pooled a total of 388 subjects with microalbuminuria and 128 with macroalbuminuria. The authors observed a reduction of UACR at 24 weeks by 32% in the microalbuminuric subjects, and 41% in the macroalbuminuric subjects. The effect was considered the same irrespective of baseline DBP, sex, body mass index (BMI), race, weight and baseline renin-angiotensin-aldosterone system inhibitors (RAASi) use. The authors also concluded that there was no difference in the reduction of UACR between the 2 different dosages of empagliflozin.

In 2017, Cherney et al. conducted a post-hoc analysis of EMPA-REG-outcome (Cherney et al. 2017) in 7,028 patients with T2D and CVD. Subjects were randomized in each study to placebo, empagliflozin 10 mg, or empagliflozin 25 mg. In this paper, the authors present the UACR results for the pooled empagliflozin group versus placebo according to albuminuria status at baseline. A total of 1,338 subjects in the pooled empagliflozin group (10 and 25 mg) had microalbuminuria at baseline and 509 had macroalbuminuria. Results of the analysis

show a reduction of UACR by 25% in microalbuminuric subjects, and 32% in macroalbuminuric subjects. In a subsequent analysis per dosage, there were no significant differences between 10 and 25 mg.

In summary, available evidence suggests comparable UACR reduction by 10 and 25 mg of empagliflozin. Therefore, this study will use the lowest registered dose of 10 mg empagliflozin, which is also the dose selected for the ongoing event-driven EMPA-Kidney study investigating the effect of empagliflozin in patients with CKD (with and without diabetes) (Herrington et al. 2018).

4.4 End of Study Definition

The EOS is defined as the date of the last visit of the last participant in the study.

The primary completion date is defined as the date when the final participant was examined or received an intervention for the purposes of final collection of data for the primary outcome.

A participant is considered to have completed the study if he/she has completed all phases of the study including the EOS visit.

5. Study Population

Prospective approval of protocol deviations to recruitment and enrollment criteria, also known as protocol waivers or exemptions, is not permitted.

5.1 Inclusion Criteria

Participants are eligible to be included in the study only if all of the following criteria apply:

Age

1. Participant must be 18 years of age or older at screening visit. The lower age limit may be higher if legally required in the participating country.

Type of Participant and Disease Characteristics

2. Participant with a clinical diagnosis of CKD and the following:
   - In Part A: eGFR 40-90 ml/min/1.73m^2 (with no more than 20% having an eGFR >75 ml/min/1.73m^2) using Chronic Kidney Disease Epidemiology Collaboration (CKD-EPI) formula (Levey et al. 2009) at screening visit and at least one historical value of eGFR <60 mL/min/1.73 m^2 within 3 months or have a registered diagnosis of CKD.
   - In Part B: eGFR 30-90 ml/min/1.73m^2 (with no more than 20% having an eGFR >75 ml/min/1.73m^2) using CKD-EPI formula (Levey et al. 2009) at screening visit and at least one historical value of eGFR <60 mL/min/1.73 m^2 within 3 months or have a registered diagnostic of CKD (see Section 2.2).
   - 300 ≤UACR <5000 mg/g at screening visit (mean value from 3 morning void samples) and documentation of albuminuria/proteinuria (quantitative or semi-quantitative measurement) in the participant's medical records at least 3 months prior to screening

   Note: One re-assessment of eGFR and/or UACR is allowed at the screening visit (see Section 5.4). If one of the 3 UACR measurements is missing but the other 2 are valid, these values can be used to assess participant's eligibility.

3. Participant with T2D as defined by the ADA (ADA 2021), with glycated hemoglobin (HbA1c) at screening <11%. Note: Historical values for HbA1c will be acceptable for inclusion providing they have been obtained within 3 months prior to screening visit.
4. Participant treated with the clinically maximum tolerated dose, as per investigator judgment, of ACEi or ARB, but not both, for more than 1 month at screening visit (see Section 6.8.1).

Sex and Contraceptive/Barrier Requirements

5. Male or female

   Contraceptive use by women should be consistent with local regulations regarding the methods of contraception for those participating in clinical studies.

   - Female participants:
     - Women of childbearing potential (see Appendix 6) can only be included in the study if a pregnancy test is negative at the screening visit and if they agree to use adequate contraception during the study and until 8 weeks after last study interventions dose. Adequate contraception is defined as any combination of at least 2 effective methods of birth control, of which at least one is a physical barrier (e.g., condoms with hormonal contraception or implants or combined oral contraceptives, certain intrauterine devices).
     - Postmenopausal females (no menses for 12 months without an alternative medical cause; see Appendix 6) are not required to use contraception. A high follicle stimulating hormone (FSH) level in the postmenopausal range may be used to confirm a postmenopausal state in women not using hormonal contraception or hormonal replacement therapy (HRT). However, in the absence of 12 months of amenorrhea, confirmation with more than one FSH measurement is required.
     - Females on HRT and whose menopausal status is in doubt will be required to use one of the non-estrogen hormonal highly effective contraception methods if they wish to continue their HRT during the study. Otherwise, they must discontinue HRT to allow confirmation of postmenopausal status before study enrollment.
     - Females of non-childbearing potential (documented hysterectomy, documented bilateral salpingectomy, documented bilateral oophorectomy) are not required to use contraception.
     - For females with permanent infertility due to an alternate medical cause other than the above, (e.g., mullerian agenesis, androgen insensitivity), investigator discretion should be applied to determining study entry.

Informed Consent

6. Capable of giving signed informed consent as described in Appendix 1 which includes compliance with the requirements and restrictions listed in the informed consent form (ICF) and in this protocol.

5.2 Exclusion Criteria

Participants are excluded from the study if any of the following criteria apply:

Medical Conditions

1. Participants with type 1 diabetes (T1D).
2. Participant with known allergies to finerenone or any SGLT2i.
3. Participant with hepatic insufficiency classified as Child Pugh C (see Appendix 7).
4. Participant with BP at Day 1 visit higher than 160/100 or SBP lower than 90 mmHg.
5. Known bilateral clinically relevant renal artery stenosis (>75%).
6. Renal allograft in place or a scheduled kidney transplant.
7. Participant with acute kidney injury (AKI) within 6 months prior to screening.
8. Participant with ketoacidosis in the past 5 years.
9. Participant with primary adrenal insufficiency (Addison's disease).
10. Stroke, transient ischemic cerebral attack, acute coronary syndrome (MI, coronary artery bypass graft [CABG], primary percutaneous coronary intervention [PCI]), or hospitalization for worsening HF, within 90 days prior to screening visit.
11. Clinical diagnosis of chronic heart failure with reduced ejection fraction (HFrEF) and persistent symptoms (New York Heart Association class II - IV) at the screening visit (class 1A recommendation for MRAs)
12. Major surgery (major according to the investigator's assessment) performed within 90 days prior to screening visit, or scheduled major elective surgery (e.g., hip replacement) within 90 days after screening visit.
13. Gastrointestinal surgery or gastrointestinal disorder that could interfere with absorption of trial medication in the investigator's opinion.
14. Any other history, condition, therapy, or uncontrolled intercurrent illness (including AKI) which could in the opinion of the investigator affect participant safety compliance with study requirements.

Prior/Concomitant Therapy

15. Participant currently treated with a SGLT2i or combined SGLT-1 and 2 inhibitor (SGLT-1/2i) or who received a SGLT2i or SGLT-1/2i which cannot be discontinued at least 8 weeks prior to the screening visit and during study intervention treatment.
16. Participant treated with strong cytochrome P450 isoenzyme 3A4 (CYP3A4) inhibitors or inducers which cannot be discontinued 7 days before Day 1 visit (see Appendix 8).
17. Participant treated with another MRA (e.g., eplerenone, esaxerenone, spironolactone, canrenone), a renin inhibitor, K+ supplements, a K+ sparing diuretic (e.g., amiloride, triamterene), a K+ binder agent, or angiotensin receptor-

neprilysin inhibitor (ARNI) within 8 weeks prior of the screening visit and during study intervention treatment[1].

18. Participants currently treated or who were treated with finerenone (Kerendia®) within 8 weeks prior to the screening visit.

Prior/Concurrent Clinical Study Experience

19. Participation in another clinical trial with an investigational product within 1 month prior to screening visit[2].

Diagnostic Assessments

20. Participant with K+ above 4.8 mmol/L at screening visit (Note: 1 re-assessment of K+ is allowed at the screening visit [see Section 5.4] and at the baseline visit).

21. Participants with alanine aminotransferase (ALT) or aspartate aminotransferase (AST) >3x upper limit of normal (ULN) at screening visit.

Other Exclusions

22. Breastfeeding female participant.

23. Participant known for lack of compliance with clinic visits or prescribed medication.

5.3 Lifestyle Considerations

5.3.1 Meals and Dietary Restrictions

Participants must refrain from consumption of grapefruit or grapefruit juice from the start of study intervention until after the final dose as it is expected to increase plasma concentration of finerenone.

5.3.2 Other Lifestyle Considerations

There are no additional lifestyle restrictions other than the dietary restrictions mentioned in Section 5.3.1.

5.4 Screen Failures

Screen failures are defined as participants who consent to participate in the clinical study but are not subsequently randomly assigned to study intervention. A minimal set of screen failure information is required to ensure transparent reporting of screen failure participants to meet the Consolidated Standards of Reporting Trials publishing requirements and to respond to queries from regulatory authorities. Minimal information includes demography, screen failure details, eligibility criteria, and any SAE.

Individuals who do not meet the criteria for participation in this study (screen failure) may not be rescreened. However, 1 re-assessment of K+ and/or eGFR and/or UACR will be allowed at screening if the test results fall outside of the ranges defined for inclusion in the study.

[1] K+ supplements and potassium binder agents will be allowed during the study for safety reasons (see Section 6.8.1).

[2] Participants who received a COVID-19 vaccine whilst still under Emergency Use Utilization will be eligible, provided vaccination occurred at least 1 month prior to screening visit.

Sites will also have the option of 1 pre-screening visit to examine UACR. This UACR value is not valid to determine eligibility and is only to be used to assess whether the participant may progress to a formal screening visit. Participants will be required to provide informed consent for this UACR test, and very limited data will be reported. Such pre-screening failures will not be considered as part of the study population. See Section 1.3 for assessments at the pre-screening visit.

5.5 Criteria for Temporarily Delaying Enrollment/Randomization/Study Intervention Administration

Participants can be enrolled out of the screening window if central laboratory results are pending. Once central laboratory results have been reviewed by the investigator, participant eligibility will be confirmed, even if he/she is out of screening window.

Current febrile illness (temperature 38.0°C [100.4° F]) or other acute illness within 48 hours before study intervention administration may also allow a participant to be randomized once the condition has resolved and the participant is otherwise eligible.

6. Study Intervention(s) and Concomitant Therapy

Study intervention is defined as any investigational intervention(s), marketed product(s), placebo, or medical device(s) intended to be administered to a study participant according to the study protocol.

6.1 Study Interventions Administered

6.1.1 Study Interventions

Finerenone 10 and 20 mg and matching placebo, as well as over-capsulated empagliflozin tablets and matching placebo, will be supplied by the sponsor or designee.

Following a screening period of up to 2 weeks, eligible participants will be randomized in a 1:1:1 ratio to receive:

- Finerenone tablet and empagliflozin over-capsulated OD (finerenone + empagliflozin arm), OR
- Finerenone tablet and matching placebo to empagliflozin OD (finerenone arm), OR
- Empagliflozin over-capsulated and matching placebo to finerenone OD (empagliflozin arm).

The randomization will be stratified by eGFR at screening (<60, ≥60 mL/min/1.73m²) and UACR at screening (≤ 850mg/g, > 850 mg/g).

See Section 4.3.1 and Section 4.3.2 for starting dose of finerenone and empagliflozin, respectively.

Study interventions will be taken in the morning, preferably in the morning at approximately the same time each day, with or without food.

The following instructions will be given to the participant in case of missed intake:

- If >8 hours before the next scheduled dose, the participant should take study interventions as soon as possible.

- If ≤8 hours of the next scheduled dose, the participant should wait and take the next study interventions at the usual time.

Arm Name	Finerenone plus Empagliflozin		Finerenone		Empagliflozin	
**Intervention Name**	Finerenone	Empagliflozin	Finerenone	Placebo for empagliflozin	Placebo for finerenone	Empagliflozin
**Type**	Drug	Drug	Drug	Drug	Drug	Drug
**Dose Formulation**	Tablet	Over-capsulated tablet	Tablet	Over-capsulated tablet	Tablet	Over-capsulated tablet
**Unit Dose Strength(s)**	10 and 20 mg	10 mg	10 and 20 mg	N/A	N/A	10 mg
**Dosage Level(s)**	One tablet daily	One capsule daily	One tablet daily	One capsule daily	One tablet daily	One capsule daily
**Route of Administration**	Oral	Oral	Oral	Oral	Oral	Oral
**Use**	Experimental	Experimental	Experimental	Placebo	Placebo	Experimental
**Packaging and Labeling**	Study Intervention will be provided in bottles. Each bottle will be labeled as required per country requirement	Study Intervention will be provided in bottles. Each bottle will be labeled as required per country requirement	Study Intervention will be provided in bottles. Each bottle will be labeled as required per country requirement	Study Intervention will be provided in bottles. Each bottle will be labeled as required per country requirement	Study Intervention will be provided in bottles. Each bottle will be labeled as required per country requirement	Study Intervention will be provided in bottles. Each bottle will be labeled as required per country requirement
**Current/Former Name(s) or Alias(es)**	BAY 94–8862-Kerendia	BI 10773-Jardiance	BAY 94–8862-Kerendia	N/A	N/A	BI 10773 -Jardiance

6.1.2 Medical Devices

No sponsor manufactured devices or devices manufactured for the sponsor are used in this study. Other medical device (not manufactured by or for sponsor) provided for use in this study is the ABPM.

Instructions for ABPM use are provided in the corresponding manual. All device deficiencies (including malfunction, use error and inadequate labeling) that caused or could have caused a SAE to a study participant shall be documented and reported by the investigator throughout the study and appropriately managed by the sponsor.

A device deficiency is an inadequacy of a medical device with respect to its identity, quality, durability, reliability, safety, or performance. Device deficiencies include malfunctions, use errors, and inadequate labeling.

For any device deficiencies related to a study participant AE or SAE: the investigator should complete the AE case report form (CRF) and safety reports (complementary pages) in addition to the Medical Device Incident CRF.

6.2 Preparation/Handling/Storage/Accountability

1. The investigator or designee must confirm appropriate temperature conditions have been maintained during transit for all study interventions received and any discrepancies are reported and resolved before use of the study interventions.
2. Only participants randomized in the study may receive study interventions and only authorized site staff may supply or administer study intervention. All study interventions must be stored in a secure, environmentally controlled, and monitored (manual or automated) area in accordance with the labeled storage conditions with access limited to the investigator and authorized site staff.
3. The investigator or the head of the institution (where applicable) is responsible for study intervention accountability, reconciliation, and record maintenance (i.e., receipt, reconciliation, and final disposition records). Drug return, reconciliation and destruction information will be captured in the IWRS.
4. Returned study intervention should not be re-dispensed to the participants.
5. In the event of a significant trial-continuity issue (e.g., caused by a pandemic), site-to-patient distribution of study interventions might occur at selected sites and visits. The investigator will maintain responsibility and control for dispensing via IxRS. The sponsor will contract a distribution provider to conduct site-to-patient distribution of study interventions, including the delegation of storage and handling as needed until handover to the participant.
6. Further guidance and information for the final disposition of unused study interventions are provided in a separate document.

6.3 Measures to Minimize Bias: Randomization and Blinding

All participants will be centrally assigned to randomized study interventions at Day 1, using an IWRS. Before the study is initiated, the telephone number and call-in directions for the IRT and/or the log in information and directions for the IWRS will be provided to each site. Once a randomization number has been assigned it must not be re-assigned.

Following enrollment of the participant, the site will contact the IWRS prior to the start of study intervention administration for each participant.

The IWRS will determine the bottle number for the study site investigator or designee to select for the participant. The investigator is to instruct participants to take 1 capsule/tablet of each study intervention, OD, preferably in the morning. The first dose of study interventions should be taken at the site on Day 1.

Study interventions will be assigned by IWRS and dispensed at the study visits summarized in the Schedule of Activities (SoA; see Section 1.3) and after the eGFR and K+ results have been made available to the investigator.

Participants will be randomly assigned in a 1:1:1 ratio to receive study interventions. The randomization will be stratified by eGFR at screening (<60, ≥60 mL/min/1.73m²) and UACR at screening (≤ 850mg/g, > 850 mg/g). The IWRS will allow to cap the number of participants with an eGFR >75 mL/min/1.73m² to 20%. Investigators and participants will remain blinded to each participant's assigned study intervention throughout the course of the study. Each study intervention and its matching placebo will be identical in appearance (size, shape, color). The packaging and labeling will be designed to maintain the blinding of the investigator's team and the participants. The study data will remain blinded until database lock and authorization of data release according to standard operating procedures.

Appropriate measures will be taken to maintain blinding while bioanalysis is ongoing.

The IWRS will be programmed with blind-breaking instructions. In case of an emergency, the investigator has the responsibility for determining if unblinding of a participant's intervention assignment is warranted. If the investigator is unavailable, and a treating physician not associated with the study requests emergency unblinding, the emergency unblinding requests are forwarded to the emergency medical advice 24-hours/7-day service. Participant safety must always be the first consideration in making such a determination. If the investigator decides that unblinding is warranted, the investigator should make every effort to contact the sponsor prior to unblinding a participant's intervention assignment unless this could delay emergency treatment of the participant. If a participant's intervention assignment is unblinded, the sponsor must be notified within 24 hours after breaking the blind. The date and reason that the blind was broken must be recorded in the source documentation and CRF, as applicable.

Sponsor safety staff may unblind the intervention assignment for any participant with an SAE. If the SAE requires that an expedited regulatory report be sent to one or more regulatory agencies, a copy of the report, identifying the participant's intervention assignment, may be sent to investigators in accordance with local regulations and/or sponsor policy.

6.4 Study Intervention Compliance

Compliance with study interventions will be assessed at each visit. Compliance will be assessed by direct questioning and counting returned capsules/tablets during the site visits and documented in the source documents and relevant form. Deviations from the prescribed dosage regimen should be recorded.

A record of the quantity of each study intervention dispensed to and administered by each participant must be maintained and reconciled with study intervention and compliance records. Intervention start and stop dates, including dates for intervention delays and/or dose reductions/increases will also be recorded.

6.5 Dose Modification

6.5.1 Finerenone

The investigator is encouraged to up-titrate the dose of study drug, at any time once the participant has been on a stable dose for 4 weeks (±4 days, e.g., from Day 30 onwards for participant starting study drug on the lower dose) and at any visit. Up-titration shall be performed provided:

- The serum/plasma K+ concentration (local laboratory value) is ≤4.8 mmol/L,

AND

- eGFR decrease (local laboratory value) is less than 30% below the value measured at the last regular visit.

K+ and eGFR values obtained from local laboratory will be used for finerenone up-/down-titration as well as monitoring after down/up-titration and restart. Potassium and eGFR values used for up-/down-titration must be documented in the electronic CRF (eCRF).

Dose adjustments of finerenone are described in Table 6-1.

Table 6-1: Dose Adjustments of Finerenone

**eGFR value at the screening visit, based on central laboratory results:**	**<60 mL/min/1.73m²**		**≥60 mL/min/1.73m²**	
**Participant randomized to group receives**	**Finerenone**<br>10 mg finerenone OD	**Placebo**<br>Placebo OD	**Finerenone**<br>20 mg finerenone OD	**Placebo**<br>Placebo OD
**Up-titration of dose** allowed from Visit 4 (Day 30) onwards provided that:<br>• K+ is ≤4.8 mmol/L[a]<br>• eGFR decrease is less than 30% below the value measured at the last scheduled visit[a]<br>• Must be documented in eCRF	20 mg finerenone OD	Sham-titrate	Not applicable	Not applicable
**Down-titration of dose**:<br>• Only for safety reasons (for guidance, see Table 6-2 and Section 8.2.5.2)<br>• Allowed any time during the study (e.g., between scheduled visits)<br>• Must be documented in eCRF<br>• An unscheduled safety visit is performed within an adequate timeframe proposed by the investigator	• If at higher dose of finerenone, down-titrate to lower dose of finerenone<br>• If at lower dose of finerenone, interrupt finerenone			

Abbreviations: eCRF = electronic case report form; eGFR = estimated glomerular filtration rate; K+ = serum/plasma potassium; OD = once daily.
NOTE: lower dose = 10 mg once daily; higher dose = 20 mg once daily.
[a] K+ and eGFR according to local laboratory values

Adjustment of dose after start of study drug intake based on blood serum K+ levels are provided in Table 6-2.

Table 6-2: Guidance for Finerenone Dose Adjustment at Day 30 and Subsequent Visits (Local Laboratory) Based on K+

**K+ (mmol/L)**	**Action**

Table 6-2: Guidance for Finerenone Dose Adjustment at Day 30 and Subsequent Visits (Local Laboratory) Based on K+

K+ (mmol/L)	Action
**First sample:**	
≤4.8	If on lower dose of study drug, up-titrate to higher dose.<br>If on higher dose of study drug, continue on the same dose.
4.9 to 5.5	Continue on the same dose.
>5.5	Withhold study drug and re-check K+ within 72 hours.
**Second and subsequent samples:**	
≤5.0	Restart study drug at lower dose.
>5.0	Continue to withhold study drug; continue to monitor K+ and restart study drug at the lower dose only if K+ is ≤5.0.

Abbreviations: K+ = serum/plasma potassium.
NOTE: lower dose = 10 mg once daily; higher dose = 20 mg once daily.

The following aspects have also to be taken into consideration:

- K+ should be measured 4 weeks (±7 days) after restarting treatment or dose adjustment, especially after up-titration
- If central laboratory results for K+ differ from local laboratory results to such an extent that the investigator is uncomfortable with the dose adjustment guidance shown in Table 6-2, blood sampling may be repeated at the investigator's discretion
- If the participant is already on the lower dose of study drug but hyperkalemia recurs soon after a previous event of hyperkalemia after restarting study intervention following interruption, and there is no explanation for the recurring hyperkalemia event other than intake of study intervention, permanent discontinuation of study intervention is recommended
- If K+ is >6.5 mmol/L, an ECG should be obtained
- Participants will maintain their normal diet throughout the study and will not be given any specific advice on dietary K+ restrictions.

Participants who started with or were up-titrated to the target dose (20 mg OD) of finerenone but who do not tolerate this dose may be down-titrated at any point during the study if required for safety reasons. These participants may be up-titrated again based on the rules provided above. If the participant is already at the lower dose, finerenone can be interrupted at the investigator's discretion (refer to Table 6-1 for guidance). If finerenone is interrupted for more than 7 days, it should be restarted at the lower dose (10 mg OD).

Subsequent to an up-titration or restart of study drug after interruption of finerenone intake for more than 7 days, the investigator should perform an unscheduled visit, 4 weeks (±7 days) after titration or restart, in order to monitor K+ levels and eGFR. If a regular study visit is scheduled to take place 4 weeks (±7 days) after up-titration, the monitoring of K+ and eGFR is assured and no unscheduled visit must be performed in addition. With down-titrations, the unscheduled safety visit may need a smaller time window and should be performed at the investigator's discretion.

At any point during study drug treatment, if K+ is found to be elevated, finerenone treatment should be adjusted according to guidance provided in Table 6-2. Both up-/down-titrations will

be at the investigator's discretion and will depend on the general clinical condition of the individual participant. The investigator should attempt to reach the maximum target dose of study drug if safety is not compromised.

All titrations, including the reasons for down-titration or for not up-titrating to the 20-mg dose, must be documented in the eCRF.

6.5.2 Empagliflozin

If additional glycemic control is required, other anti-diabetic agents may be added or doses adjusted at the discretion of the principal investigator according to local guidelines (see Section 6.8.1).

6.6 Continued Access to Study Intervention After the End of the Study

At study completion, the investigator will decide in consultation with the individual participant if additional treatment is required and choose from existing treatment options. The investigator must provide follow-up medical care for all participants who complete the study or who are prematurely withdrawn from the study or must refer them for appropriate ongoing care as required.

6.7 Treatment of Overdose

6.7.1 Finerenone

No cases of AEs associated with finerenone overdose in humans have been reported during its development. The most likely manifestation of overdose is anticipated to be hyperkalemia. If hyperkalemia develops, standard treatment will be initiated, as per local guidelines, and action with regards to study drug should be taken as mentioned in Section 6.5.1.

6.7.2 Empagliflozin

6.7.2.1 Symptoms

In controlled clinical studies, single doses of up to 800 mg empagliflozin in healthy volunteers and multiple daily doses of up to 100 mg empagliflozin in patients with T2D did not show any toxicity. Empagliflozin increased urine glucose excretion leading to an increase in urine volume. The observed increase in urine volume was not dose dependent and is not clinically meaningful. There is no experience with doses above 800 mg in humans (EU SmPC).

6.7.2.2 Therapy

In the event of an overdose, treatment should be initiated as appropriate to the participant clinical status.

The removal of empagliflozin by hemodialysis has not been studied (EU SmPC).

In the event of an overdose, the investigator should:

- Contact the medical monitor immediately.
- Evaluate the participant to determine, in consultation with the medical monitor, whether study intervention should be interrupted.
- Closely monitor the participant for any AE/SAE (at least 48 hours).
- Document the quantity of the excess dose as well as the duration of the overdose in the eCRF.

Decisions regarding dose interruptions or modifications will be made by the investigator in consultation with the medical monitor based on the clinical evaluation of the participant.

6.8 Concomitant Therapy

All treatment that the investigator considers necessary for the participant's welfare may be administered at the discretion of the investigator in keeping with the standards of medical care.

ACEis or ARBs are considered as SoC therapy in patients with CKD and T2D, and often prescribed to patients with CKD at early stages (Molitch et al. 2014).

It is advisable to follow the recommendations of local guidelines for the management of CVD and CKD, the use of statins, anti-platelets, and beta-blockers, and for glycemic control.

The medical monitor should be contacted if there are any questions regarding concomitant or prior therapy.

Any medication or vaccine (including over the counter or prescription medicines, recreational drugs, vitamins, traditional Chinese medicines, and/or herbal supplements) that the participant is receiving at the time of screening or receives during the study must be recorded along with:

- Reason for use
- Dates of administration including start and end dates
- Dosage information including dose and frequency.

6.8.1 Permitted Concomitant Therapy

The following concomitant therapies will be permitted during the study:

- Insulin, insulin secretagogues, and thiazolidinediones (TZD). For participants treated with a sulfonylurea or with insulin, a lower dose of the sulfonylurea or insulin will have to be considered by the investigator to reduce the risk of hypoglycemia.
- ACEis or ARBs at stable doses; change in ACEis or ARBs doses will be allowed for safety purposes, only. The maximum clinically tolerated dose for ACEis or ARBs should be the highest dose, according to local labels, which the participant can safely tolerate. This dose should not be below the minimum labeled dose to maximize therapeutic benefit of background SoC.
- Antihypertensive therapy will be administered according to local guidelines. Depending on BP, serum creatinine, or eGFR, participants may need to have their study intervention dose, or the dose of another concomitant medication reduced or discontinued. The dosage of SoC therapies should not be reduced to solely facilitate maintenance of study intervention.
- Loop diuretics and thiazides should be carefully evaluated before inclusion; if participant is taking loop diuretic at study inclusion, diuretic dose should be adjusted appropriately, according to PI discretion. Loop diuretic and thiazides dosages should be stable during the study. Changes in dosage will be allowed for safety purposes (see Section 6.8.5).
- Treatments of hyperkalemia as per local guideline recommendations.

  - K+ lowering agents (e.g., sodium polystyrene sulfonate, calcium polystyrene sulfonate, patiromer, sodium zirconium cyclosilicate) are allowed to be started in case of a hyperkalemia event.
- Treatments of hypoglycemia as per local guideline recommendations.
- Treatments of hypotension as per local guideline recommendations (see Section 6.8.5).
- Glucagon-like peptide-1 receptor agonist treatments are allowed if dose was stable for at least 4 weeks prior to the screening visit. Dose reductions (down-titration) will be allowed during the study. Dose increases (up-titrations) will not be allowed. If needed, the investigators should privilege the use of insulin or TZD to control HbA1c.

Trimethoprim and trimethoprim-sulfamethoxazole can be used with caution. Increased K+ monitoring and potential temporary discontinuation of finerenone may be advised.

6.8.2 Prohibited Concomitant Therapy

The following concomitant therapies will be prohibited during the study. Participants should be discontinued from the study if receiving prohibited concomitant therapy.

- K+ sparing diuretics (e.g., amiloride, triamterene)
- K+ supplements
- Marketed finerenone (Kerendia®)
- Other MRAs (e.g., eplerenone, esaxerenone, spironolactone, canrenone)
- Any renin inhibitor (e.g., aliskiren)
- ARNI
- Concomitant therapy with both an ACEi and an ARB
- Strong CYP3A4 inhibitors (see Appendix 8)
- Any other SGLT-2i or SGLT-1/2i during as long as the participant is taking study interventions and up to 7 days after last study intervention intake.

6.8.3 Hyperkalemia Events

See Section 6.5.1.

6.8.4 Unexpected Acute Declines in eGFR

If an unexpected, acute decline in kidney function is observed, the participant should be evaluated. Volume depletion, hypotension, intercurrent medical problems and concomitant drugs may cause increases in serum creatinine. Urinary tract infection and urinary obstruction should be considered (the latter especially in men). Several drugs may cause a decline in kidney function, especially nonsteroidal anti-inflammatory drugs and certain antibiotics such as trimethoprim. If any drug is suspected of causing or contributing to worsening kidney function, their use should be re-considered.

6.8.5 Volume Depletion/Hypotension

Participants with clinically relevant symptoms/signs of suspected volume depletion and/or hypotension, should have their regular medication reviewed, and consideration given to reducing the dose of, or stopping concomitant non-essential medications, as assessed on an individual basis, including diuretics and drugs that lower BP (except ACEi, ARB, or beta-blockers prescribed for HF). The need for conventional diuretics (or the dose of diuretic used) should be reevaluated considering the participant’s symptoms and signs. In participants

with HF, discontinuation of diuretic should only be undertaken cautiously. Hypotension may also occur with other BP lowering drugs and once again the need for (and dose of) non-essential agents of this type (e.g., calcium channel blockers, alpha adrenoceptor antagonists, and nitrates) should also be re-considered.

6.8.6 Ketoacidosis

Reports of ketoacidosis, a serious life-threatening condition requiring urgent hospitalization have been identified in clinical trials and post marketing surveillance in patients with T1D and T2D mellitus receiving SGLT2i, including empagliflozin. Fatal cases of ketoacidosis have been reported in patients taking empagliflozin.

Participants treated with study interventions who present with signs and symptoms consistent with severe metabolic acidosis should be assessed for ketoacidosis regardless of presenting blood glucose levels, as ketoacidosis associated with empagliflozin may be present even if blood glucose levels are less than 250 mg/dL. If ketoacidosis is suspected, study interventions should be discontinued, participant should be evaluated, and prompt treatment should be instituted. Treatment of ketoacidosis may require insulin, fluid, and carbohydrate replacement. In case of ketoacidosis, the investigator will investigate whether the participant is not suffering from T1D instead of T2D.

In many of the post-marketing reports, and particularly in patients with T1D, the presence of ketoacidosis was not immediately recognized and institution of treatment was delayed because presenting blood glucose levels were below those typically expected for diabetic ketoacidosis (often less than 250 mg/dL). Signs and symptoms at presentation were consistent with dehydration and severe metabolic acidosis and included nausea, vomiting, abdominal pain, generalized malaise, and shortness of breath. In some but not all cases, factors predisposing to ketoacidosis such as insulin dose reduction, acute febrile illness, reduced caloric intake, surgery, pancreatic disorders suggesting insulin deficiency (e.g., T1D, history of pancreatitis, or pancreatic surgery), and alcohol abuse were identified.

Before initiating study interventions, factors in the participant history that may predispose to ketoacidosis including pancreatic insulin deficiency from any cause, caloric restriction, and alcohol abuse will be considered.

For participants who undergo scheduled surgery, temporary discontinuation of study drug for at least 3 days prior to surgery will be considered.

Monitoring for ketoacidosis and temporary discontinuation of study interventions in other clinical situations known to predispose to ketoacidosis (e.g., prolonged fasting due to acute illness or post-surgery) will be considered. Risk factors for ketoacidosis will be resolved prior to restarting study intervention.

Participants will be educated on the signs and symptoms of ketoacidosis and instructed to discontinue study interventions and seek medical attention immediately if signs and symptoms occur.

7. Discontinuation of Study Intervention and Participant Discontinuation/Withdrawal

7.1 Discontinuation of Study Intervention

7.1.1 Permanent Discontinuation of Study Intervention

In rare instances, it may be necessary for a participant to permanently discontinue study intervention.

Participants must be withdrawn from the study intervention for the following reasons:

- If the participant experiences unacceptable toxicities to any study intervention:
  - Ketoacidosis (see Section 6.8.6)
  - Necrotizing fasciitis of the perineum (Fournier's gangrene).
- At the specific request of the sponsor and in liaison with the investigator (e.g., obvious noncompliance, safety concerns).

For one single participant, permanent discontinuation of study intervention means that the 2 study treatments are discontinued (finerenone tablet and empagliflozin, or finerenone tablet and empagliflozin placebo, or empagliflozin and finerenone placebo).

Any participant deciding to discontinue study interventions will always be asked about the reason(s) and the presence of any AEs. The reason for premature discontinuation of the study interventions should be documented in the source documentation and captured in the eCRF.

Even participants who have stopped taking study interventions are expected to attend all the protocol specified study visits and will be encouraged to perform all assessments as stipulated in the visit schedule.

If it is not possible for a participant who has permanently discontinued study intervention to attend any visit(s) in person, the site staff will keep in touch with him/her by means of phone or virtual contact to the participant himself/herself, or to a person pre-designated by the participant, in accordance with the participant's study visit schedule. Data will continue to be collected about his/her health status, including information on adverse events. This information may be provided either by the participant himself/herself, his/her general practitioner, or a family relative (if allowed in the respective country). Data, such as information on survival and potential protocol specified endpoints, might be also collected from a healthcare provider, from public or medical records, or other sources as available according to local guidelines and as allowed by local regulations. These data will be collected until the study is concluded, even if the participant no longer attends study visits in person, unless he/she withdrew consent and did not agree to release further information.

See the SoA (Section 1.3) for data to be collected at the time of discontinuation of study intervention and follow-up and for any further evaluations that need to be completed.

7.1.2 Temporary Discontinuation

Interruption of study intervention is permitted at any time during the study for the following reasons:

- Temporary interruption of study interventions may be considered in participants thought to be at risk of volume depletion/hypotension, such as participants with an acute medical illness potentially causing volume depletion because of inadequate fluid intake or

fluid/blood loss (e.g., gastroenteritis, gastrointestinal hemorrhage), or those undergoing major surgery.

- Other safety reasons (see Section 6.5 for guidance).
- Temporary discontinuation of study intervention for an incurrent illness, at the discretion of the investigator
- In the event of a trial-continuity issue (e.g., caused by a pandemic), the sponsor may provide additional guidance in study-specific communication.

Upon temporary interruption of the study intervention due to hyperkalemia, eGFR decrease, (S)AE, intolerability, or any other reason, intake should be resumed as soon as medically acceptable at the discretion of the investigator. There is no defined maximum time limit for temporary interruption. In all cases, the reason for study intervention interruption must be recorded in the eCRF and the participant's medical records.

If the study intervention is interrupted for more than 7 days, the re-start should be performed at the 10-mg dose and the investigator should schedule a 4-week safety visit (±7 days) in order to monitor K+ levels and renal function (see Table 6-1). If a regular visit will be scheduled to take place 4 weeks ±7 days after up-titration or re-start, the monitoring of K+ and renal function is assured, and no 4-week safety visit has to be performed in addition.

7.2 Participant Discontinuation/Withdrawal from the Study

- A participant may withdraw from the study at any time at his/her own request or may be withdrawn at any time at the discretion of the investigator for safety, behavioral, or compliance reasons. This is expected to be uncommon.
- At the time of discontinuing from the study, if possible, an ED visit should be conducted, as shown in the SoA (Section 1.3). See SoA for data to be collected at the time of study discontinuation and follow-up and for any further evaluations that need to be completed.
- The participant will be permanently discontinued both from the study interventions and from the study at that time.
- If the participant withdraws consent for disclosure of future information, the sponsor may retain and continue to use any data collected before such a withdrawal of consent.

7.3 Lost to Follow-Up

A participant will be considered lost to follow-up if he/she repeatedly fails to return for scheduled visits and is unable to be contacted by the study site.

The following actions must be taken if a participant fails to return to the clinic for a required study visit:

- The site must attempt to contact the participant and reschedule the missed visit as soon as possible and counsel the participant on the importance of maintaining the assigned visit schedule and ascertain whether or not the participant wishes to and/or should continue in the study.
- Before a participant is deemed lost to follow-up, the investigator or designee must make every effort to regain contact with the participant (where possible, 3 telephone calls and, if necessary, a certified letter to the participant's last known mailing address or local

equivalent methods). These contact attempts should be documented in the participant's medical record.

- Should the participant continue to be unreachable, he/she will be considered to have withdrawn from the study.
- Site personnel, or an independent third party, will attempt to collect the vital status of the participant within legal and ethical boundaries for all participants randomized, including those who did not get study intervention. Public sources may be searched for vital status information. If vital status is determined as deceased, this will be documented, and the participant will not be considered lost to follow-up. Sponsor personnel will not be involved in any attempts to collect vital status information.

Discontinuation of specific sites or of the study as a whole are handled as part of Appendix 1.

8. Study Assessments and Procedures

- Study procedures and their timing are summarized in the SoA (Section 1.3). Protocol waivers or exemptions are not allowed.
- Immediate safety concerns should be discussed with the sponsor immediately, upon occurrence or awareness, to determine if the participant should continue or discontinue study intervention.
- Adherence to the study design requirements, including those specified in the SoA, is essential and required for study conduct.
- All screening evaluations must be completed and reviewed to confirm that potential participants meet all eligibility criteria. The investigator will maintain a screening log to record details of all participants screened and to confirm eligibility or record reasons for screening failure, as applicable.
- Procedures conducted as part of the participant's routine clinical management (e.g., blood count) and obtained before signing of the ICF may be utilized for screening or baseline purposes provided the procedures met the protocol specified criteria and were performed within the time frame defined in the SoA.
- Laboratory results that could unblind the study will not be reported to investigative sites or other blinded personnel until the study has been unblinded.
- Repeat or unscheduled samples may be taken for safety reasons or for technical issues with the samples.
- In the event of a significant trial-continuity issue (e.g., caused by a pandemic), alternate strategies for participant visits, assessments, medication distribution and monitoring may be implemented by the sponsor or the investigator, as per local health authority/ethics requirements.

8.1 Efficacy Assessment

Planned time points for efficacy assessment are provided in the SoA (Section 1.3).

UACR

The UACR (mg of albumin per gram of creatinine) will be measured in the first morning void urine samples collected at the participant's home. For the optional pre-screening and the screening eligibility, samples will be collected on 3 consecutive days, while only 2 samples

will be collected for the following visits. Only pre-screening UACR will be assessed at local laboratory, when a central laboratory will be used for all other time points.

The participant will be provided with the necessary urine sample collection kit. Collected samples should not be refrigerated but stored in a provided cooling bag until they can be brought to the study site for further processing in accordance with the laboratory manual. The last of the collection days will be the day of the study visit, for the optional pre-screening and screening visits, only. At the other visits, the 2 consecutive samples can be collected ±7 days from the visit date.

If one of the collection samples is missing, the UACR for a time point will be calculated based on the available results.

Samples collected for UACR determination should not be used for assessment of safety parameters and pregnancy test.

UACR will be assessed at all study visits.

8.2 Safety Assessments

Safety will be assessed by monitoring and recording all AEs and SAEs, cardiac, hematologic, and blood chemistry parameters, vital signs, and any abnormal findings observed during the performance of physical examinations.

Planned time points for all safety assessments are provided in the SoA (see Section 1.3).

8.2.1 Vital Signs

Vital signs will be performed at the study site, at the times indicated in SoA (see Section 1.3):

- Pulse rate and BP will be assessed at all visits.
- BP and pulse measurements will be assessed in a sitting position, with a completely automated device. Manual techniques will be used only if an automated device is not available.
- BP and pulse measurements should be preceded by at least 5 minutes of rest for the participant in a quiet setting without distractions (e.g., television, cell phones).
- Vital signs (to be taken before blood collection for laboratory tests) will consist of 1 pulse and 3 BP measurements (3 consecutive BP readings will be recorded at intervals of at least 1 minute). The average of the 3 BP readings will be recorded.

8.2.1.1 Ambulatory Blood Pressure Monitoring – Only in Part A

In addition, participants enrolled in Part A will be equipped for 24 hours with an ABPM device 1 hour before the first study intervention intake, at Day 1. The name and address for the ABPM service provider can be found in the documentation supplied by the vendor.

ABPM starts during the visit and finishes approximately 24 hours later on the following day.

The 24-hour profiles will be recorded at the following intervals:

- At 30-min intervals from 06:00 to <22:00 (daytime).
- At 60-min intervals from 22:00 to <06:00 (night time).

During the recording of 24-hour ABPM, participants should refrain from unusual physical exercise.

ABPM is to be performed with a non-invasive oscillometric ambulatory BP monitor used for multiple measurements of brachial BP over an extended period of time. Once the monitor is returned to the site after the 24-hour recording, ABPM data will be uploaded to the ABPM supplier platform by the study sites. For a detailed description of the ABPM measurement schedule and procedures refer to the Manual of Procedures.

8.2.2 Physical Examinations

Physical examinations will be performed at the times indicated in SoA (see Section 1.3):

- A complete physical examination will be included, at a minimum, assessments of the CV, respiratory, gastrointestinal, and neurological systems. Weight (to the nearest 0.1 kg in indoor clothing without shoes) will also be measured and recorded. Height will only be measured and recorded at the screening visit.
- Investigators should pay special attention to clinical signs related to previous serious illnesses.

8.2.3 Electrocardiograms

12-lead ECGs will be obtained as outlined in the SoA (see Section 1.3):

- Single 12-lead ECG will be performed locally using an ECG machine that automatically calculates the heart rate and measures PR, QRS, QT, and QT corrected intervals.
- If there is any clinical indication, unscheduled 12-lead ECGs can be performed at any point during the study.
- In addition, an ECG should be obtained within 72 hours (see Section 6.5.1) if a participant has a K+ >6.5 mmol/L.
- The interpretation of the tracing must be made locally by a qualified physician.
- The date of the recording must be documented on the ECG section of the eCRF. Each ECG tracing should be labeled with the study number, participant number, and date and kept in the source documents at the study site.
- Clinically significant abnormalities should be reported as AEs or outcome event as appropriate (e.g., new onset of atrial fibrillation) when not already reported in the medical history or in one of the previous recordings and in the event of worsening of previous findings.

8.2.4 Laboratory Assessments

- See Appendix 2 for the list of clinical laboratory tests to be performed and see the SoA (Section 1.3) for the timing and frequency.
- Laboratory assessments will be performed for both efficacy and safety purposes. Per the SoA (see Section 1.3), UACR, eGFR, K+ as well as clinical chemistry, hematology, and urine analysis, will be assessed by the central laboratory (except optional pre-screening UACR which will be assessed at a local laboratory). K+ and eGFR will also be analyzed by a local laboratory and these assessments will be used for randomization, up-/down-titration and monitoring further to dose modification of finerenone as per SoA (see Section 1.3). **These samples may be obtained up to 72 hours before a scheduled visit in order to be available for the visit.**
- The investigator must review the laboratory report, document this review, and record any clinically significant changes occurring during the study as an AE. The laboratory reports must be filed with the source documents.

- Abnormal laboratory findings associated with the underlying disease are not considered clinically significant unless judged by the investigator to be more severe than expected for the participant's condition.
- All laboratory tests with values considered clinically significantly abnormal during participation in the study or within 72 hours after the last dose of study intervention should be repeated until the values return to normal or baseline or are no longer considered clinically significant by the investigator or the medical monitor.
    - If clinically significant values do not return to normal/baseline within a period judged reasonable by the investigator, the etiology should be identified, and the sponsor notified.
    - All protocol-required laboratory tests, as defined in Appendix 2, must be conducted in accordance with the laboratory manual and the SoA.
    - If laboratory values from non-protocol specified laboratory tests performed at the institution's local laboratory require a change in participant management or are considered clinically significant by the investigator (e.g., SAE or AE or dose modification), then the results must be recorded in the eCRF.
- The steps for the review and processing of the local laboratory data will be documented within the Local Laboratory Data Handling Plan. This plan will define the processes for obtaining and using laboratory data within the clinical database to ensure the delivery of accurate laboratory data.

8.2.5 Other Safety Assessments

8.2.5.1 eGFR

eGFR will be automatically calculated by the central laboratory by using the CKD-EPI creatinine equation (Levey et al. 2009). Estimate GFR values calculated with this formula (eGFRcr) will be used for screening/eligibility and primary data analysis.

A new CKD-EPI creatinine- and cystatin C-based equation will also be used to calculate eGFR without the race factor (Inker et al. 2021). Estimate GFR values calculated with this formula (eGFRcr-cys) will be used for sensitivity analysis.

For local laboratory values, eGFR will be calculated in the eCRF by using the corresponding CKD-EPI formula.

eGFR will be assessed at all study visits.

8.2.5.2 Monitoring of Potassium

Hyperkalemia is a frequent event in participants with CKD progressing to ESKD and one of the main risks identified from previous studies with MRAs including finerenone. K^+ will be monitored closely during this study, with assessments of K^+ scheduled at all visits (see Section 1.3).

During treatment with study interventions, all assessments of K^+, including re-tests, will be performed at both local and central laboratories. Due to sample handling and extended transportation time to the central laboratory, falsely elevated K^+ values in centrally (but not locally) analyzed samples have been observed in previous studies with finerenone. Since the value of K^+ in the local sample is usually not affected by this and results from the local

laboratory are available earlier, local samples will be taken not only for safety purposes but also for dose adjustments.

Re-checking of K+ values must be done at the latest within 72 hours of the investigator being aware of results that need confirmation. The investigator can perform a retest at any time if he/she considers it necessary to confirm the K+ concentration of the local or the central sample.

K+ values should be recorded using a single decimal point (e.g., 4.5 mmol/L).

8.2.5.2.1 Moderate or Severe Hyperkalemia

Moderate hyperkalemia will be defined as K+ ≥5.5 to ≤6.0 mmol/L and severe hyperkalemia will be defined as K+ >6.0 mmol/L).

8.2.5.3 Acute Kidney Injury

AKI is defined as any of the following:

- An increase in serum creatinine by greater than or equal to 0.3 mg/dL within 48 hours; or
- An increase in serum creatinine by greater than or equal to 1.5 times baseline, which is known or presumed to have occurred within the prior 7 days; or
- A urine volume less than 0.5 ml/kg/h for 6 hours (KDIGO 2012).

8.2.5.4 Severe Hypoglycemia

Severe hypoglycemia will be defined as glucose level of <3.0 mmol/L (<54 mg/dL) is sufficiently low to indicate serious, clinically important hypoglycemia. In addition, severe hypoglycemia, as defined by the ADA denotes severe cognitive impairment requiring external assistance for recovery (Agiostratidou et al. 2017). It should be documented in the eCRF as an AE.

8.2.5.5 Symptomatic Hypotension (Including Volume Depletion)

Occurrence of symptomatic hypotensive and syncope events will be assessed at all study visits and should be documented as AEs.

8.2.5.6 Genital Mycotic Events

Occurrence of urinary tract infection events will be assessed at all study visits and should be documented as AEs.

8.2.5.7 Ketoacidosis Events

Occurrence of ketoacidosis events will be assessed at all study visits and should be documented as AEs.

8.2.5.8 Necrotizing Fasciitis of the Perineum (Fournier's Gangrene) Events

Occurrence of necrotizing fasciitis of the perineum events will be assessed at all study visits and should be documented as AEs.

8.2.5.9 Urosepsis and Pyelonephritis Events

Occurrence of urosepsis and pyelonephritis events will be assessed at all study visits and should be documented as AEs.

8.2.6 Pregnancy Testing

Serum pregnancy test will be performed for all female participants of childbearing potential at timepoints indicated in the SoA (see Section 1.3).

8.2.7 Suicidal Ideation and Behavior Risk Monitoring

Not applicable for this study.

8.3 Adverse Events (AEs), Serious Adverse Events (SAEs) and Other Safety Reporting

The definitions of AEs and SAEs can be found in Appendix 4.

AEs will be reported by the participant.

The investigator and any qualified designees are responsible for detecting, documenting, and recording events that meet the definition of an AE or SAE and remain responsible for following up all AEs that are serious, considered related to the study intervention, or that caused the participant to discontinue the study intervention or the study (see Section 7).

The method of recording, evaluating, and assessing causality of AEs and SAEs and the procedures for completing and transmitting SAE reports are provided in Appendix 4.

8.3.1 Time Period and Frequency for Collecting AE and SAE Information

AEs and SAEs will be collected from the start of study intervention until the last follow-up visit at the time points specified in the SoA (see Section 1.3). AEs and SAEs which are related to protocol-required study procedures (e.g., adjustment of therapy to comply with inclusion or exclusion criteria of the study) will be recorded as AEs or SAEs from the signing of the ICF.

Any medical occurrences/conditions that begin in the period between signing ICF and the start of study intervention, and which are not related to a protocol-required study procedure, will be recorded on the medical history/current medical conditions, not as AEs.

All SAEs will be recorded and reported to the sponsor or designee immediately and under no circumstance should this exceed 24 hours of learning of the event, as indicated in Appendix 4. The investigator will submit any updated SAE data to the sponsor immediately and not longer than 24 hours of it being available.

Investigators are not obligated to actively seek information on AEs or SAEs after conclusion of the study participation. However, if the investigator learns of any SAE, including a death, at any time after a participant has been discharged from the study, and he/she considers the event to be reasonably related to the study intervention or study participation, the investigator must promptly notify the sponsor.

8.3.2 Method of Detecting AEs and SAEs

Care will be taken not to introduce bias when detecting AEs and/or SAEs. Open-ended and non-leading verbal questioning of the participant is the preferred method to inquire about AE occurrences.

8.3.3 Follow-Up of AEs and SAEs

After the initial AE/SAE report, the investigator is required to proactively follow each participant at subsequent visits/contacts. All SAEs, and AEs of special interest (AESIs; as defined in Section 8.3.8), will be followed until resolution, stabilization, the event is otherwise explained, or the participant is lost to follow-up (as defined in Section 7.3). Further information on follow-up procedures is provided in Appendix 4.

8.3.4 Regulatory Reporting Requirements for SAEs

- Prompt notification by the investigator to the sponsor of an SAE is essential so that legal obligations and ethical responsibilities toward the safety of participants and the safety of a study intervention under clinical investigation are met.
- The sponsor has a legal responsibility to notify both the local regulatory authority and other regulatory agencies about the safety of a study intervention under clinical investigation. The sponsor will comply with country-specific regulatory requirements relating to safety reporting to the regulatory authority, Institutional Review Boards (IRB)/Independent Ethics Committees (IEC), and investigators.
- An investigator who receives an investigator safety report describing an SAE or other specific safety information (e.g., summary or listing of SAEs) from the sponsor will review and then file it along with the IB and package insert] and will notify the IRB/IEC, if appropriate according to local requirements.
- Investigator safety reports will be prepared for SUSAR according to local regulatory requirements and sponsor policy and forwarded to investigators as necessary.

For disease-related events (DREs) excluded from SAE reporting, see Section 8.3.7.

8.3.5 Pregnancy

- Details of all pregnancies in female participants will be collected after the start of study intervention and until 8 weeks after last study intervention dose.
- If a pregnancy is reported, the investigator will record pregnancy information on the appropriate form and submit it to the sponsor immediately and not longer than 24 hours of learning of the female participant pregnancy.
- While pregnancy itself is not considered to be an AE or SAE, any pregnancy complication or elective termination of a pregnancy for medical reasons will be reported as an AE or SAE.
- Abnormal pregnancy outcomes (e.g., spontaneous abortion, fetal death, stillbirth, congenital anomalies, ectopic pregnancy) are considered SAEs, and will be reported as such.
- The participant will be followed to determine the outcome of the pregnancy. The investigator will collect follow-up information on the participant and the neonate, and the information will be forwarded to the sponsor.
- Any post-study pregnancy-related SAE considered reasonably related to the study intervention by the investigator will be reported to the sponsor as described in Section 8.3.4. While the investigator is not obligated to actively seek this information in former study participants, he or she may learn of an SAE through spontaneous reporting.
- Any female participant who becomes pregnant while participating in the study will discontinue study intervention and be withdrawn from the study.

8.3.6 Cardiovascular and Death Events

Death could be classified into the following categories:

- CV death
- Non-CV death.

Classification of deaths will be based on investigator's judgment.

All deaths will be considered as CV deaths unless otherwise classified by the MedDRA preferred term. All unwitnessed deaths will be classified as CV death.

See Appendix 5 for definitions of each category and Section 8.3.7 for DRE.

8.3.7 Disease-Related Events and/or Disease-Related Outcomes Not Qualifying for Expedited Reporting as AE or SAE

The following DREs are common in participants with CKD in T2D and can be serious/life-threatening:

- Kidney failure defined as:
    - ESRD: Initiation of chronic dialysis (hemo- or peritoneal dialysis) for at least 30 days or renal transplantation.
    - Decrease of eGFR to less than 15 mL/min/1.73m², confirmed by at least one additional standardized measurement at least 4 weeks after the initial measurement.
- Renal death (see Appendix 5 for definition)
- Chronic sustained decrease in eGFR (see Appendix 3 for definition)
- CV death (see Appendix 5 for definition)
- Non-fatal stroke (see Appendix 3 for definition)
- Non-fatal MI (see Appendix 3 for definition)
- Hospitalization for HF (see Appendix 3 for definition)
- New onset of HF (see Appendix 3 for definition).

Because these events are typically associated with the disease under study, they will not be reported according to the standard process for expedited reporting of SAEs even though the event may meet the definition of an SAE. These events will be recorded immediately of investigator's awareness. These DREs will be monitored by the DMC on a regular basis (see Appendix 1).

However, if either of the following conditions applies, then the event must be recorded and reported as an AE/SAE (instead of a DRE):

- The event is, in the investigator's opinion, of greater intensity, frequency, or duration than expected for the individual participant.

OR

- The investigator considers that there is a reasonable possibility that the event was related to study intervention.

Note: These rules will not entirely apply to participants enrolled in Japan where the only events recorded as DREs will be kidney failure, renal death, and chronic sustained decrease in

eGFR. CV death, non-fatal stroke, non-fatal MI, hospitalization for HF, and new onset of HF have to be reported as SAE for Japanese participants.

8.3.8 Adverse Events of Special Interest

To further characterize the safety of finerenone in combination with empagliflozin, symptomatic hypotension as well as AKI will be collected as AESIs and must be reported to the sponsor.

There is no need for expedited reporting unless it meets the criteria for an SAE as defined in Appendix 4; however, these events will need to be monitored and reviewed, and documented timely and accurately both in the source data and on the eCRF.

8.4 Pharmacokinetics

Collection of blood samples for pharmacokinetics of finerenone and empagliflozin will be performed for all participants at the time points indicated in the SoA (see Section 1.3). These samples will not be analyzed directly but may be analyzed in case of specific questions from the DMC, SC, or sponsor.

For the investigation of systemic exposure to finerenone and empagliflozin and their relationship with treatment effects, the plasma concentrations of finerenone and empagliflozin will be determined at different time points using a sparse sampling approach in all participants. The plasma concentration versus time data collected will be evaluated descriptively, separated by dose and visit. Plots will be prepared of all individual plasma concentrations versus actual relative study times (time of sample collection after time of study intervention administration).

At Visits 3 and 6, trough (i.e. pre-dose) samples for the determination of finerenone and empagliflozin plasma concentrations will be drawn before intake of study interventions. At these visits, study intervention will be administered at the study site by study personnel and the exact time of study intervention intake on the day before the visit and on the day of the visit and the exact sampling time will be recorded in the eCRF. The study personnel should contact the participant prior to Visits 3 and 6 to remind her/him not to take the study interventions as usual in the morning at home and to remind her/him to properly document time of intake.

At Visits 4, 5, and ED (if applicable), blood samples for the determination of finerenone and empagliflozin plasma concentrations will be drawn during the visit 1.5 to 10 hours after study intervention intake at home. The participants should be advised to take their study interventions as usual in the morning at home and recall the time of study intervention intake or note the time of study intervention intake on the contact card. The exact time of study intervention intake and the exact sampling times will be recorded in the eCRF.

Note: At all visits mentioned above, samples should be taken even if the study interventions were not taken as indicated. In such cases, particular attention will be paid to properly document the actual time of study intervention intake before sampling.

Pharmacokinetics and exposure-response analysis may be performed using population approaches (popPK and popPK/PD, e.g., by non-linear mixed effect modeling). Such evaluations will be described in a separate analysis plan and will be reported separately. Such evaluations may be started prior to database lock. If this is applicable, appropriate measures will be taken to maintain blinding of the study team, e.g., data will be stored separately, and

members of the study team will neither have access to the randomization list nor to individual data.

Details about the collection, processing, storage and shipment of samples will be provided separately (e.g., sample handling sheets or laboratory manual).

8.5 Genetics and/or Pharmacogenomics

Genetics and pharmacogenomics are not evaluated in this study.

8.6 Biomarkers

Biomarkers will be evaluated in samples collected before, during and after treatment in order to determine the impact of study intervention. These biomarkers fall into the following categories:

- Biomarkers related to the mode-of-action of the study intervention and/or functional markers of the cardiovascular system: vasoactive agents (aldosterone, plasma renin activity etc.), NT-proBNP/B-type natriuretic peptide (BNP), and sodium.
- OMICS/Multiplex Analysis: semi-targeted/untargeted omics analysis (e.g., metabolomics by mass spectrometry), OLINK Proximity Extension Assays (e.g., “Explore” Panels) may identify ‘*de novo*’ PD biomarkers, biomarkers which support the understanding of the mode-of-action and/or biomarkers which may indicate disease progression.
- Further biomarkers related to the mode-of-action or the safety of study intervention and similar drugs may be examined. The same applies to further biomarkers deemed relevant to kidney and (cardio)vascular diseases and associated health problems. These investigations may include e.g., diagnostic, safety, PD, monitoring, or potentially predictive biomarkers.

Timing of Collection

The planned time points of sample collection are provided in the SoA in Section 1.3. If deemed necessary, the sampling time points or frequency according to the SoA may be adjusted.

Specimen Types

The following sample types will be collected for biomarker analysis:

- Blood (plasma/serum)
- Urine

The exact specimen type for a particular biomarker analysis (e.g., serum or plasma) will be provided in separate documents (e.g., sample handling sheets or lab manual).

Sample Handling and Storage

Details on the collection, processing, shipment, and storage of samples will also be provided in separate documents (e.g., sample handling sheets or lab manual). Samples may be stored for a maximum of 15 years (or according to local regulations) following the end of the study at a facility selected by the sponsor to enable further analyses.

Reporting

Biomarker investigations may be reported separately (e.g., in a biomarker evaluation report) except for plasma renin activity, aldosterone, urinary sodium, and biomarkers described in Section 9 or the statistical analysis plan (SAP).

8.7 Immunogenicity Assessments

Not applicable for this study.

8.8 Health Economics

Health Economics/Medical Resource Utilization and Health Economics parameters are not evaluated in this study.

9. Statistical Considerations

9.1 Statistical Hypotheses

The primary analysis population for analyses of the primary endpoints will be the full analysis set (FAS). Define μ_i as the relative change (ratio) from baseline to 180 days in UACR for treatment group i, where I = Combi (finerenone and empagliflozin), Emp (empagliflozin), Fin (finerenone).

The multiple primary endpoints are:

- Relative change in UACR from baseline at 180 days in combination (finerenone and empagliflozin) versus empagliflozin alone
- Relative change in UACR from baseline at 180 days in combination (finerenone and empagliflozin) versus finerenone alone.

In order to evaluate whether the combination of finerenone and empagliflozin is superior in reducing UACR than either empagliflozin or finerenone alone, we will analyze this using a repeated measures mixed model. However, to be conservative in our estimations we will apply a 2-sided 2-sample t-test of equal variance at an overall two-sided significance level of alpha=0.05. To adjust for the multiple testing of two hypotheses, the Bonferoni-Holm method will be applied. The 2 two-sided p-values are first ordered increasingly. If the lower p-value $P_i<0.025$, the corresponding null hypothesis can be rejected. The p-value Pj of the other test can then be compared with 0.05. If Pj<0.05, the second null hypothesis can also be rejected.

The primary hypotheses to be tested for the primary endpoints are provided below:

- $H0_{P1}$: $\mu_{Comb} - \mu_{Emp} = 0$
- Ha_{P1}: $\mu_{Comb} - \mu_{Emp} \neq 0$
- $H0_{P2}$: $\mu_{Comb} - \mu_{Fin} = 0$
- Ha_{P2}: $\mu_{Comb} - \mu_{Fin} \neq 0$

With two-sided overall significance level = 0.05 (0.025 for the initial hypothesis).

9.2 Sample Size Determination

Study Design and Objective

The objective of the study will be to investigate the efficacy and safety of combination of finerenone and SGLT2i versus the individual components. This will be a 3-arm study

comparing combination therapy and finerenone alone and combination therapy and SGLT2i alone. The primary analysis method will be a repeated measures mixed model. However, the following calculation is based on using a two-sample t-test. Justification for this can be found in the fact that the t-test offers conservative estimates compared to the mixed model, as it only describes the final visit and adding in the information from all the visits in the study should increase the power.

The primary summary variable will be the ratio to baseline of UACR after 6 months. The objective of the study is to test the superiority of combination compared to SGLT2i and/or finerenone by applying a 2-sided two-sample t-test of equal variance at an overall two-sided significance level of alpha=0.05. To adjust for the multiple testing of two hypotheses, the Bonferoni-Holm method will be applied. The 2 two-sided p-values are first ordered increasingly. If the lower p-value P1<0.025, the corresponding null hypothesis can be rejected. The p-value P2 of the other test can then be compared with 0.05. If P2<0.05, the second null hypothesis can also be rejected.

All participants are planned to be treated for 6 months with either combination therapy or finerenone alone or SGLT2i alone.

Assumptions about UACR

UACR will be analyzed assuming a lognormal distribution. Assumptions for the UACR ratios to baseline after 6 months for finerenone are based on the data of the FIDELIO-DKD study. Assumptions for SGLT2i are based on the EMPA-REG outcome trial.

Effect of SGLT2i

It is estimated that the effect of the SGLT2i on UACR at 6 months will be approximately 30% (Wang et al. 2016; Bae et al. 2019; Cherney et al. 2016; Cherney et al. 2017; DeFronzo et al. 2015; Ridderstrale et al. 2014; Softeland et al. 2017), therefore, a mean ratio to baseline of 0.7 of UACR at 180 days is reasonable.

Effect of Finerenone

In the FIDELIO-DKD study the UACR ratio to baseline after 4 months was 0.646 and 0.573 after 12 months in the finerenone group. Using linear interpolation we were able to estimate of the value at 6 months: (0.573/-0.646)/(12-4)*(6-4)+0.646 = 0.628. The value of 0.628 for the effect of finerenone on UACR at 6 months will be investigated for the power calculations.

Effect of Combination Therapy

The combination therapy aims to be powered to detect a 20% further reduction in UACR to the monotherapy (see Section 2.1). As such, when testing the combination versus SGLT2i alone, it is intended to detect a mean ratio to baseline of 0.56 (further 20% reduction from 0.7). For combination versus finerenone alone, it is intended to detect a mean ratio to baseline of 0.5024 (further 20% reduction from 0.628).

Standard Deviation

In the FIDELIO-DKD study the standard deviation of the difference between the log-transformed UACR values after 4 months and at baseline was 0.77 in the finerenone. The data from another finerenone trial (ARTS-DN) showed a SD of 0.7 for the effect of finerenone on UACR after 3 months. Therefore, based on the FIDELIO-DKD data, a standard deviation of 0.77 is reasonable.

Sample Size

When testing the combination therapy versus finerenone alone, group sample sizes of 226 and 226 achieve 80% power to reject the null hypothesis of equal means when the log-transformed population mean difference is ln(μ1) – ln(μ2) = ln (0.502 / 0.628) = -0.224, with a standard deviation for both groups of 0.77 and with a significance level (alpha) of 0.025 using a 2-sided 2-sample -equal variance t-test.
When testing the combination therapy versus SGLT2i alone group sample sizes of 228 and 228 achieve 80% power to reject the null hypothesis of equal means when the log-transformed population mean difference is ln(μ1) – ln(μ2) = ln(0.56 / 0.7) = -0.223, with a standard deviation for both groups of 0.77 and with a significance level (alpha) of 0.025 using a 2-sided 2-sample -equal variance t-test.
There will be the same number of participants in each group, so for the combi therapy versus finerenone alone the power will be boosted to approximately 81% due to the extra participants included. Therefore, group sample sizes of 228, 228, and 228 will be sufficient.
Assuming a 15% drop out rate an extra 123 participants are required takes the total sample size to 807. Therefore, group sample sizes of 269, 269, and 269 will be sufficient to detect a 20% further reduction in UACR in the combination arm versus empagliflozin or finerenone.

9.3 Analysis Sets

For the purposes of analysis, the following analysis sets are defined:

Table 9-1: Analysis Sets

Participant Analysis Set	Description

Table 9-1: Analysis Sets

Participant Analysis Set	Description
Listing Only Set (LOS)	All other participants screened who did not receive any dose of study intervention or for whom no data after beginning of treatment are available will be classified as LOS.<br>Their data will be presented in the individual participant data listings but will not be included in any statistical analysis.
Safety Analysis Set (SAF)	All randomized participants who have taken at least 1 dose of study intervention.<br>All participants will be analyzed according to the actual study intervention received. For the non-combination therapy, i.e., finerenone, or empagliflozin alone, if a participant receives both study interventions due to a bottle error, the study intervention actually received for the majority of the time in the study will be used.
Full Analysis Set (FAS)	All randomized participants.<br>All participants will be analyzed according to the planned study intervention (the intent-to-treat principle).
Per-Protocol Analysis Set (PPS)	All participants of the FAS without any important protocol deviations which would interfere with the evaluation of the efficacy data.<br>All participants will be analyzed according to the actual study intervention(s) received. For the non-combination therapy i.e., finerenone, or empagliflozin alone, if a participant receives both treatments due to a bottle error, the treatment received for the majority of the time in the study will be used.
Pharmacokinetic Analysis Set	All randomized participants receiving at least 1 dose of study intervention and have 1) at least 1 quantifiable concentration and 2) no protocol deviation that would interfere with PK data evaluation.

The stratum variable eGFR category used in the statistical analysis will be derived based on the screening eGFR assessment. This assessment will use the 2009 CKD-EPI formula (Levey et al. 2009). All participants will be analyzed according to their correct stratification category. In the event of stratification errors, the primary analysis will also be repeated based on the stratification category used in the randomization as a sensitivity analysis.

9.4 Statistical Analyses

The SAP will be finalized prior to database lock and will include a more technical and detailed description of the statistical analyses described in this section. This section is a summary of the planned statistical analyses of the most important endpoints including primary and exploratory endpoints.

The primary and exploratory endpoints are defined in Section 3.

9.4.1 General Considerations

Statistical analysis will be performed using SAS; the version used will be specified in the SAP.

A lognormal distribution is assumed for serum creatinine and UACR. For all other metric variables, a normal distribution is assumed. The distributional assumptions will be investigated and if necessary, nonparametric methods or transformation of the data will be considered.

All variables will be analyzed by descriptive statistical methods. The number of data available, mean, standard deviation (SDv), minimum, median, and maximum will be calculated for metric data. The geometric mean and SDv will be provided instead of the arithmetic mean and SDv for the variables where lognormal distributions are assumed.

Frequency tables will be generated for categorical data.

Baseline values will be defined as the last non-missing measurement before first study intervention intake (Day 1). If the last observation available prior to randomization is the measurement from the screening visit, this would be used as the baseline value. This also includes assessments from a local laboratory, in case that prior to randomization, no assessment from the central laboratory is available. Otherwise baseline will be missing. If more than one measurement was planned for a scheduled time point, for example BP measurements, the mean value of the last set of measurements per time point prior to randomization will be used as the baseline value.

Only the data provided by the central laboratory will be used for analysis, values from local laboratories will not be used in the statistical analysis and listed only.

In the event of repeated measurements for screening and the randomization visit (Day 1, baseline), the closest measurement prior to randomization will be used for analysis instead of the scheduled measurements. At all visits post-randomization, unless stated otherwise, only the values at scheduled measurements will be used for analysis.

The derived visit ‘any time post baseline’ (applicable for efficacy) will include any measurement after randomization, including unscheduled assessments. For the derived visit ‘any time on-treatment’, only assessments up to 30 days after last study intervention administration, including unscheduled assessments, will be considered (applicable for efficacy; for safety assessments within 3 days after last study intervention administration will be considered).

Comparisons will only be drawn between the combination therapy and each monotherapy. No statistical comparison between the monotherapies will be performed.

Further details on the statistical analyses will be provided in the SAP.

9.4.2 Disposition, Baseline, History, Demography and Medication

The analyses of disposition, baseline, history, and demography are described below.

9.4.3 Disposition

The number of participants screened, screen failed, enrolled, randomized, and valid for the safety analysis set (SAF), FAS, and per-protocol analysis set (PPS) will be summarized overall and by treatment group, country, and investigator. The number of participants discontinuing the treatment and follow-up epochs, together with the primary reason for discontinuation, will be presented by treatment group and overall, in separate tables. In addition, the number of participants with important protocol deviations and validity findings will be presented overall, by investigator and country for each treatment group, and in total. The frequencies of each important protocol deviation and validity finding will be presented by treatment group and in total.

9.4.3.1 Population Characteristics

Population characteristics analyses, except for participant disposition, will be performed for the FAS, if not stated otherwise.

9.4.3.2 Demography and Other Baseline Characteristics

Demography includes age, sex, race, ethnicity, region (North America, Europe, and Asia), body weight, body height, BMI, smoking history (never, former, current smoker), drugs, caffeine, and alcohol consumption. Other baseline characteristics include baseline UACR, K+, categories for K+ (≤4.5 mmol and >4.5 mmol), eGFR (calculated by CKD-EPI [Levey et al. 2009] formula), serum creatinine, HbA1c, and values for vital signs parameters (i.e., systolic BP, diastolic BP, and pulse rate).

All demographic data and baseline characteristics will be tabulated by treatment group and overall. The demographic and other baseline characteristics table will also be presented, separated by each level of the stratification factors, UACR category and eGFR category.

The non-stratified demographic and other baseline characteristics table will be repeated for all other analysis sets if they differ in sample size from the FAS.

Demographics and other baseline characteristics will be presented for the FAS separately for the participants belonging to PPS or not (only overall, not by treatment group).

9.4.3.3 Medical History

Medical history will be coded using the Medical Dictionary for Regulatory Activities (MedDRA) dictionary. Medical history will be presented for each MedDRA primary System Organ Class (SOC) and Preferred Term (PT) by treatment group and overall, in a summary table. Additional medical history terms by Standardized MedDRA Queries (SMQ) will also be presented.

9.4.3.4 Concomitant Medication

Concomitant medication will be coded using the WHO Drug Dictionary (WHO-DD). The number of participants who took at least 1 concomitant medication, the number of participants who took at least 1 medication that started and ended before administration of study intervention, and the number of participants who took at least 1 concomitant medication that started after start of study intervention will be presented by treatment group and overall using Anatomical Therapeutic Chemical (ATC) classes and subclasses. These tables will be repeated, summarizing the number of participants with medication in the Bayer drug groups of interest (including ACEi, ARBs, insulin, insulin secretagogues, metformin, beta-blocker, diuretics, K+ sparing diuretics, K+ supplements, K+ lowering agents, alpha blocking agents, calcium channel blockers, centrally acting antihypertensives and strong, unclassified, moderate, or weak CYP3A4 inhibitors, trimethoprim, trimethoprim-sulfamethoxazole, any other SGLT-2i or combined SGLT-1 and 2i). A participant will be counted only once within each ATC class/subclass or sponsor drug group, respectively.

A listing will be provided including all medication classified as a weak, moderate, or strong CYP3A4 inhibitor according to the sponsor drug groupings together with the respective classification information.

9.4.3.5 Treatment Duration, Extent of Exposure, Up-Titration Status, and Compliance

The analyses described in this section will be repeated for the SAF and PPS if they differ in sample size from the FAS.

Treatment duration (number of months with study intervention intake) will be summarized using descriptive statistics by treatment group and overall. In addition, treatment duration will

be categorized and presented with the corresponding number and percentage of participants by treatment group and overall. Further specification of the categories will be provided in the SAP.

A table will be presented with the absolute and relative frequencies of participants still in the study at each visit. Kaplan-Meier plots for '*Time to end of study intervention*' will be provided. The extent of exposure to study intervention (total amount of intake in grams) will be summarized using descriptive statistics by treatment group.

The up-titration status (yes/no), regardless of actual or sham up-titration, will be summarized with absolute and relative frequencies per treatment group for each visit as well as participants never up-titrated, up-titrated once, and up-titrated more than once. Compliance (as a percentage) will be calculated as follows:

- 100 * Number of taken tablets or capsules/Number of planned tablets or capsules.

The number of planned tablets or capsules will be calculated as follows:

- (Days from randomization to last intake of study intervention + 1) * Number of planned tablets or capsules per day.

All tablets and capsules, including the dummy placebo tablets and capsules, will be counted. For participants who withdraw prematurely from the study intervention, compliance will be calculated up to the time of last dose.

The compliance will be summarized descriptively by treatment group and overall. In addition, percent of compliance will be categorized into 3 groups, <80%, ≥80 to ≤120%, and >120%, and the categories will be summarized by treatment group and overall.

9.4.4 Primary Efficacy Endpoints

The primary efficacy endpoints are:

- Relative change from baseline in UACR at 180 days in combination (finerenone and empagliflozin) versus empagliflozin alone
- Relative change from baseline in UACR at 180 days in combination (finerenone and empagliflozin) versus finerenone alone.

In order to evaluate whether finerenone and empagliflozin is superior in reducing UACR than either empagliflozin or finerenone alone, a two-sided two-group t-test of equal means at a multiplicity adjusted (Bonferroni-Holm) significance level of 0.05/2 = 0.025 will be applied for the initial hypothesis test.

UACR during the study will be summarized descriptively by treatment group and visit including ratios to baseline. These analyses will be performed overall and separated by the stratification factors (UACR category and eGFR category).

The log-transformed ratio of UACR to baseline at each visit up to 180 days will be analyzed by a mixed model with the factors treatment group, visit, treatment by visit interaction, factors for the 2 stratification levels, UACR category and eGFR category, log-transformed baseline value as covariate nested within type of albuminuria, and log-transformed baseline value by visit interaction. Pairwise ratios between the finerenone plus empagliflozin, and the empagliflozin treatment group will be calculated and corresponding two-sided 95% CIs will be computed.

The same analysis will be performed between the finerenone plus empagliflozin, and the finerenone treatment group.

Intercurrent events shall be handled using the policies outlined in Section 3. Patients that experience kidney transplant/dialysis/death will be included in the analysis with all measures up to the date of the event. Patients that discontinue treatment will have all measures available included in the analysis.

The primary analysis of the primary efficacy variable will be repeated in the PPS as a supportive analysis. An 'on-treatment' analysis will be performed, including only events occurring while taking study drug or until 30 days after stop of study drug. This analysis will be performed in the FAS. Sensitivity analyses for the primary endpoint will be performed taking into consideration different strategies for handling the intercurrent events of kidney transplant/dialysis/death. Further details for all analyses will be provided in the statistical analysis plan.

9.4.5 Secondary Efficacy Endpoints

The secondary efficacy endpoints will be analyzed in the FAS population unless otherwise specified in SAP. Time-to-event endpoints will be analyzed using stratified log–rank test with randomization stratification factors. Hazard ratio and 95% CI will be provided using the Cox model stratified by the same factors as stated above. Detailed analysis methods and the plan for type 1 error control for exploratory endpoints will be specified in SAP.

The secondary analysis endpoints are:

- Relative change in UACR between end of treatment visit and at 30 days after end of treatment visit
- Relative change in UACR between 30 days after end of treatment visit and baseline
- Relative change in UACR category (>30%, >40%, and >50%) at 180 days.

9.4.5.1 Relative Change in UACR Category

Frequency tables will be generated for the number of participants with a relative decrease and increase in UACR of >30%, >40%, and >50% from baseline UACR. The analysis will be performed for each visit and for any time post baseline. The analysis will also be performed stratified for each level of the stratification factors (UACR category and eGFR category).

A shift table will be provided displaying the number of participants who changed from baseline to each visit. Change in albuminuria category will only be considered as shifts if they are accompanied by a UACR decrease of at least 30% from baseline to each visit.

The additional categorical UACR efficacy variables listed in Section 3 will be summarized for presence or absence of the event using logistic regression with the factor's treatment group and stratification levels (UACR category and eGFR category). Pairwise differences between the combination versus empagliflozin or combination versus finerenone group will be calculated and corresponding two-sided 95% CIs will be computed.

9.4.5.2 Subgroup Analyses

Exploratory subgroup analyses are planned for the primary efficacy variable.

This will include descriptive statistics and a statistical test for interaction.

The following subgroups will be considered for exploratory subgroup analyses:

- Region (North America, Europe, and Asia)
- eGFR category at screening and baseline (eGFR 40 to <60, 60 to 75 mL/min/1.73m^2)

- History of CVD (present, absent)
- Baseline K+ (≤4.5 versus >4.5 mmol/L)
- Systolic BP at baseline (>90 to <130, 130 to <160 mmHg).

It is anticipated that in these proposed subgroups for analysis, differences in treatment effects may be observed according to the screening or baseline characteristics defined, due in part to the differences in the risk of clinical events expected in the different subgroups.

Furthermore, subgroup analysis usually required will be performed, including the following subgroups:

- Race
- Sex
- Age group.

9.4.6 Safety Analysis

All analyses on safety data will be performed in the SAF.

The following safety variables will be assessed during the study:

- AEs
- Laboratory data
- ECG data
- Vital signs, including weight and BMI
- Further safety variables:
  - Change from baseline in eGFR at 30 days
  - eGFR decline greater than 30% at Day 30 from baseline
  - Change from baseline in eGFR at 180 days
  - Change from baseline in eGFR at 210 days
  - Proportion of participants with AKI events
  - Total number of AKI events
  - Change from baseline in K+
  - Proportion of participants with hyperkalemia events
  - Total number of hyperkalemia events
  - Proportion of participants with moderate hyperkalemia events (K+ >5.5 to ≤6.0 mmol/L)
  - Total number of moderate hyperkalemia events (K+ >5.5 to ≤6.0 mmol/L)
  - Proportion of participants with severe hyperkalemia events (K+ >6.0 mmol/L)
  - Total number of severe hyperkalemia events (K+ >6.0 mmol/L)
  - Proportion of participants with severe hypoglycemia events
  - Total number of severe hypoglycemia events
  - Proportion of participants with symptomatic hypotension events
  - Total number of symptomatic hypotension events
  - Proportion of participants with genital mycotic events
  - Total number of genital mycotic events

- Proportion of participants with ketoacidosis
- Total number of ketoacidosis events
- Proportion of participants with necrotizing fasciitis of the perineum events
- Total number of necrotizing fasciitis of the perineum events.
- Proportion of participants with urosepsis and pyelonephritis events
- Total number of urosepsis and pyelonephritis events.

9.4.6.1 Adverse Event

AEs will be coded using the MedDRA (latest version available prior to data base freeze). A listing will be provided linking the original investigator terms and the coded terms. AEs will also be presented grouped by SMQs.

AEs that started or worsened after the first dose of study intervention up to 3 days after any temporary or permanent interruption of study intervention will be considered as treatment-emergent AEs (TEAEs).

An overall summary of all AEs and TEAEs will be generated by treatment group. The number of participants with TEAEs, post-treatment AEs occurring more than 3 days after stop of study intervention, treatment-emergent SAEs, treatment-emergent study intervention-related AEs, treatment-emergent study intervention-related SAEs, TEAEs causing permanent discontinuation of study intervention, treatment-emergent non-serious AEs, non-serious AEs, TEAEs by maximum intensity, treatment-emergent SAEs by maximum intensity, study intervention-related TEAEs by maximum intensity, TEAEs by worst outcome, and treatment-emergent SAEs by worst outcome will be summarized by treatment group using MedDRA terms grouped by primary SOC and PT.

In case of events with different intensity within a participant, the maximum reported intensity will be used. If intensity is missing, the event will be considered as severe. If the same event is reported as both unrelated and related to the study intervention within a participant, the event will be reported as related to study intervention. If the drug relationship is missing, the event will be considered as being related to the study intervention.

Separate tables summarizing TEAEs, treatment-emergent study intervention-related AEs, and SAEs that occurred in more than 5% of the participants will be provided.

Deaths, SAEs, and AEs leading to permanent study intervention discontinuation will be listed separately.

9.4.6.2 Laboratory Data

The number of participants with treatment-emergent (until 3 days after any temporary or permanent interruption of study intervention) abnormal laboratory values above or below the normal range will be tabulated by the laboratory parameter and treatment group.

Summary statistics including changes to baseline will be calculated by treatment group and visit for all quantitative laboratory parameters, e.g., for hematology, HbA1c, clinical chemistry, and urinalysis. Geometric statistics and ratios to baseline will be presented for creatinine instead of arithmetic statistics with changes from baseline. For eGFR, the relative change will be displayed in addition to the absolute change from baseline.

Summary statistics for K+, eGFR, and serum creatinine will also be repeated by treatment group and visit separately for each level of the stratification factors (UACR category and

eGFR category). As a sensitivity analysis, the analysis on eGFR will be repeated using the updated CKD-EPI formula published in 2021 (Inker et al. 2021).

9.4.6.3 Vital Signs, Including Weight and BMI

At the corresponding visits, 3 measurements of BP will be taken in sitting position with at least a 1-minute interval between each reading. Averages of non-missing values of these 3 measurements will be calculated and used for the statistical analysis. If only 1 of the planned measurements is available, this value will be used.

Vital signs values will be summarized by treatment group and visit using descriptive statistics including absolute changes from baseline. The analysis will be repeated for systolic BP stratified by baseline systolic BP >90 to <130 mmHg, 130 to <160 mmHg, and ≥160 mmHg.

The values and the changes from baseline will be summarized by treatment group and visit using descriptive statistics for weight and BMI.

9.4.6.4 Further Safety Variables

All safety variables are listed at the start of this section. Not covered in the above sections are detailed in the below sections.

For each analysis, the percentage of participants with the respective events (non-stratified) at any time post-baseline (including unscheduled assessments) will be compared between the finerenone and empagliflozin, and the empagliflozin treatment group by applying separate explorative χ^2 tests with continuity correction. If the expected number of participants in at least 1 cell of the 2x2 contingency table is <5, Fisher's exact test will be applied instead of the χ^2 test. Estimates and 2-sided 95% CIs will be provided for each treatment group and the treatment differences. Clopper Pearson CIs will be calculated for each treatment group, while for treatment differences the exact unconditional confidence limits will be calculated. Similarly, this will be repeated for finerenone and empagliflozin, and the finerenone treatment group.

The eGFR endpoints will be analyzed in the FAS population unless otherwise specified in SAP. As a sensitivity analysis, the analysis on eGFR endpoints will be repeated using the updated CKD-EPI formula published in 2021 (Inker et al. 2021).

Time-to-event endpoints will be analyzed using stratified log-rank test with randomization stratification factors. Hazard ratio and 95% CI will be provided using the stratified Cox proportional hazards model. Detailed analysis methods and the plan for type 1 error control for exploratory endpoints will be specified in the SAP.

9.4.6.4.1 Ratio of Change from Baseline in eGFR at 30 days

The endpoint will be analyzed as described for the primary endpoint. As a sensitivity analysis, this will be repeated using the updated CKD-EPI formula published in 2021 (Inker et al. 2021).

9.4.6.4.2 eGFR Decline Greater than 30% at 30 days from Baseline

The percentage of participants with eGFR decline greater than 30% at Day 30 from baseline will be summarized by treatment group. As a sensitivity analysis, this will be repeated using the updated CKD-EPI formula published in 2021 (Inker et al. 2021).

9.4.6.4.3 Ratio of change in eGFR at 180 days and 210 days from Day 30

The endpoint will be analyzed as described for the primary endpoint. As a sensitivity analysis, this will be repeated using the updated CKD-EPI formula published in 2021 (Inker et al. 2021).

9.4.6.4.4 Proportion of Participants with Acute Kidney Injury and Number of AKI Events

The percentage of participants with and total number of events of AKI will be summarized by treatment group.

9.4.6.4.5 Proportion of Participants with Hyperkalemia and Number of Hyperkalemia Events

The percentage of participants with and total number of events of hyperkalemia (K+ >5.5 mmol/L) will be summarized by treatment group.

9.4.6.4.6 Proportion of Participants with Moderate Hyperkalemia and Number of Moderate Hyperkalemia Events

The percentage of participants with and total number of events of moderate hyperkalemia (K+ >5.5 mmol/L to ≤6.0 mmol/L) will be summarized by treatment group.

9.4.6.4.7 Proportion of Participants with Severe Hyperkalemia and Number of Severe Hyperkalemia Events

The percentage of participants with and total number of events of severe hyperkalemia (K+ >6.0 mmol/L) will be summarized by treatment group.

9.4.6.4.8 Proportion of Participants with Severe Hypoglycemia Events and Number of Severe Hypoglycemia Events

The percentage of participants with and total number of events of severe hypoglycemia events will be summarized by treatment group.

9.4.6.4.9 Proportion of Participants with Symptomatic Hypotension and Number of Symptomatic Hypotension Events

The percentage of participants reporting symptomatic hypotension and syncope events and total number of symptomatic events will be summarized by treatment group.

9.4.6.4.10 Proportion of Participants with Genital Mycotic Events and Number of Genital Mycotic Events

The percentage of participants with and total number of events of genital mycotic events will be summarized by treatment group.

9.4.6.4.11 Proportion of Participants with Ketoacidosis Events and Number of Ketoacidosis Events

The percentage of participants with and total number of events of ketoacidosis will be summarized by treatment group.

9.4.6.4.12 Proportion of Participants with Necrotizing Fasciitis of the Perineum and Number of Necrotizing Fasciitis of the Perineum Events

The percentage of participants with and total number of events of necrotizing fasciitis of the perineum will be summarized by treatment group.

9.4.6.4.13 Proportion of Participants with Urosepsis and Pyelonephritis and Number of Urosepsis and Pyelonephritis Events

The percentage of participants with and total number of events of urosepsis and pyelonephritis events will be summarized by treatment group.

9.4.7 Missing Data/Dropouts

A participant who has been randomized and discontinues study participation prematurely for any reason, either from study intervention or from follow-up, is defined as a '*dropout*', even if no study intervention has been taken. Dropouts will not be replaced.

Data from participants who prematurely terminated the study will be used to the maximum extent possible.

All efforts will be made to collect complete data for all participants randomized in this study. Participants will be followed until the end of the study, and all required data will be collected, regardless of their compliance with study intervention use or visit schedule.

After randomization, study intervention discontinuation for any reason does not constitute withdrawal from the study and should not lead to the participant being withdrawn from the study. On the contrary, even participants who have stopped taking study intervention are expected to attend all the protocol specified study visits and will be encouraged to perform all assessments as stipulated in the visit schedule.

If it is not possible for a participant who has withdrawn from study intervention to attend any visit(s) in person, the site staff will keep in touch with the participant by means of phone or virtual contact to the participant himself/herself, or to a person pre-designated by the participant, in accordance with the participant's study visit schedule.

Data will continue to be collected about his/her health status, including information on adverse events. This information may be provided either by the participant himself/herself, his/her general practitioner, or a family relative (if allowed in the respective country). Data, such as information on survival and potential protocol specified endpoints, might be also collected from a healthcare provider, from public or medical records, or other sources as available according to local guidelines and as allowed by local regulations. These data will be collected until the study is concluded, even if the participant no longer attends study visits in person, unless he/she withdrew consent and did not agree to release further information.

When an event date is not known, the site investigator will be asked to provide a best estimate as to when the event occurred. Even though the exact date of an event is unknown, the investigator often has some information that would give an approximate date (e.g., the first week of a month, the fall of a year, or the middle of a particular year) or at least the date when the participant was last seen or contacted. This information can be meaningfully incorporated into the estimated date recorded, as this is likely to be closer to the true date than any produced by an uninformed computer algorithm. This estimated date should be the middle date within the period that the event is known to have occurred. If the event is known

to have occurred in the first week of a month, then the date in the middle of that week should be recorded as the estimate. If it occurred in the fall of a year, then the middle date in the fall is the appropriate estimate. If no information is known, then the date in the middle of the plausible period should be given, based on the last contact with the participant prior to the event and the date of contact when information about the event was known.

Data from participants who prematurely terminate the study will be used to the maximum extent possible. All missing or partial data will be presented in the participant data listing as they are recorded on the eCRF. Data are collected primarily through an electronic data capture system, which allows ongoing data entry and monitoring.

For those participants who withdraw consent, sensitivity analyses will be performed to assess the impact of potential informative censoring of such participants. These will include the use of different imputation rules for considering participants without an event of the primary composite endpoint as having an event or being censored.

9.5 Interim Analysis

No interim analysis is planned for this study.

10. Supporting Documentation and Operational Considerations

10.1 Appendix 1: Regulatory, Ethical, and Study Oversight Considerations

10.1.1 Regulatory and Ethical Considerations

- This study will be conducted in accordance with the protocol and with the following:
    - Consensus ethical principles derived from international guidelines including the Declaration of Helsinki and Council for International Organizations of Medical Sciences International Ethical Guidelines
    - Applicable International Council for Harmonization (ICH) GCP Guidelines
    - Applicable laws and regulations.
- The protocol, protocol amendments, ICF, IB, and other relevant documents (e.g., advertisements) must be submitted to an IRB/IEC by the investigator and reviewed and approved by the IRB/IEC before the study is initiated.
- Any amendments to the protocol will require IRB/IEC approval before implementation of changes made to the study design, except for changes necessary to eliminate an immediate hazard to study participants. Any substantial modification of the protocol will be submitted to the competent authorities as substantial amendments for approval, in accordance with ICH GCP and national and international regulations.
- Protocols and any substantial amendments to the protocol will require health authority approval prior to initiation except for changes necessary to eliminate an immediate hazard to study participants.
- The investigator will be responsible for the following:
    - Providing written summaries of the status of the study to the IRB/IEC annually or more frequently in accordance with the requirements, policies, and procedures established by the IRB/IEC
    - Notifying the IRB/IEC of SAEs or other significant safety findings as required by IRB/IEC procedures
    - Providing oversight of the conduct of the study at the site and adherence to requirements of ICH guidelines, the IRB/IEC, and all other applicable local regulations.

10.1.2 Financial Disclosure

Investigators and sub-investigators will provide the sponsor with sufficient, accurate financial information as requested to allow the sponsor to submit complete and accurate financial certification, or disclosure statements to the appropriate regulatory authorities. Investigators are responsible for providing information on financial interests during the study and for 1 year after completion of the study.

10.1.3 Informed Consent Process

- The investigator or his/her representative will explain the nature of the study to the participants or their legally authorized representative and answer all questions regarding the study.
- Participants must be informed that their participation is voluntary. Participants or their legally authorized representative will be required to sign a statement of informed consent that meets the requirements of 21 Code of Federal Reguation 50, local regulations, ICH

guidelines, Health Insurance Portability and Accountability Act requirements, where applicable, and the IRB/IEC or study center.

- The medical record must include a statement that written informed consent was obtained before the participant was enrolled in the study and the date the written consent was obtained. The authorized person obtaining the informed consent must also sign the ICF.
- Participants must be re-consented to the most current version of the ICF(s) during their participation in the study.
- A copy of the ICF(s) must be provided to the participants or their legally authorized representative.

10.1.4 Data Protection

- Participants will be assigned a unique identifier by the sponsor. Any participant records, datasets or biological samples that are transferred to the sponsor will contain the identifier only; participant names or any information which would make the participant identifiable will not be transferred.
- The participant must be informed that his/her personal study-related data will be used by the sponsor in accordance with local data protection law. The level of disclosure must also be explained to the participant who will be required to give consent for their data to be used as described in the informed consent
- The participant must be informed that his/her medical records may be examined by Clinical Quality Assurance auditors or other authorized personnel appointed by the sponsor, by appropriate IRB/IEC members, and by inspectors from regulatory authorities.

10.1.5 Committee Structure

10.1.5.1 Data Monitoring Committee

Ongoing safety monitoring during the conduct of the study will be performed by an external and independent DMC. An independent statistical analysis center (SAC) will be involved in processing unblinded safety data for the DMC. Analysis periods and procedures will be defined in an operational charter (DMC charter) filed in the study file.

The independent DMC will periodically review and assess safety data from the study for imbalances in safety outcomes in an unblinded manner. It is believed that in this way, participant safety can continue to be monitored throughout the duration of the trial, and the integrity of the study maintained. If unexpected safety issues are identified, specific amendments will be implemented based on the recommendation of the DMC.

Following data review, the DMC will provide written recommendations that will be transferred to the chairmen of the SC and the sponsor. DMC opinions and recommendations will be notified by the sponsor as soon as possible to the competent authorities and the IRBs/IECs where they qualify for expedited reporting.

10.1.5.2 Steering Committee

The SC will consist of external experts in the area of nephrology and diabetology. The SC will be blinded to the study data while the trial is ongoing. Their main responsibilities are as follows:

- Provide input to protocol-related issues and protocol amendments that may arise during the study.

- Oversee study progress and provide recommendations to the sponsor in regard to any necessary modifications that may be required in study conduct or study monitoring.
- Transmission of information to individual investigators.
- Serve as resource for scientific review of sub-studies, publications, presentations, and/or educational material as applicable.

10.1.6 Dissemination of Clinical Study Data

Result Summaries of Bayer's sponsored clinical trials in drug development phases 2, 3, and 4, and phase 1 trials in participants are provided in the Bayer Trial Finder application after marketing authorization approval in line with the position of the global pharmaceutical industry associations laid down in the "Joint Position on the Disclosure of Clinical Trial Information via Clinical Trial Registries and Databases". In addition, results of clinical drug trials will be provided on the publicly funded website www.ClinicalTrials.gov and European Union (EU) Clinical Trials Register in line with the applicable regulations.

Bayer commits to sharing upon request from qualified scientific and medical researchers, participant-level clinical trial data, study-level clinical trial data, and protocols from clinical trials in participants for medicines and indications approved in the US and EU on or after 01 JAN 2014 as necessary for conducting legitimate research.

All Bayer-sponsored clinical trials are considered for publication in the scientific literature irrespective of whether the results of the clinical trials are positive or negative.

10.1.7 Data Quality Assurance

- All participant data relating to the study will be recorded on printed or eCRF unless transmitted to the sponsor or designee electronically (e.g., laboratory data). The investigator is responsible for verifying that data entries are accurate and correct by physically or electronically signing the eCRF.
- Guidance on completion of eCRFs will be provided in the eCRF guidelines.
- The investigator must permit study-related monitoring, audits, IRB/IEC review, and regulatory agency inspections and provide direct access to source data documents.
- Quality tolerance limits (QTLs) will be predefined in the Integrated Quality Risk Management Plan to identify systematic issues that can impact participant safety and/or reliability of study results. These predefined parameters will be monitored during the study and important deviations from the QTLs and remedial actions taken will be summarized in the clinical study report.
- Monitoring details describing strategy (e.g., risk-based initiatives in operations and quality such as Risk Management and Mitigation Strategies and Analytical Risk-Based Monitoring), methods, responsibilities and requirements, including handling of noncompliance issues and monitoring techniques (central, remote, or on-site monitoring) are provided in the Monitoring Plan.
- The sponsor or designee is responsible for the data management of this study including quality checking of the data.
- The sponsor assumes accountability for actions delegated to other individuals (e.g., contract research organizations).

- Records and documents, including signed ICFs, pertaining to the conduct of this study must be retained by the investigator for 25 years after study completion unless local regulations or institutional policies require a longer retention period. No records may be destroyed during the retention period without the written approval of the sponsor. No records may be transferred to another location or party without written notification to the sponsor.

10.1.8 Source Documents

- Source documents provide evidence for the existence of the participant and substantiate the integrity of the data collected. Source documents are filed at the investigator's site.
- Data reported on the eCRF or entered in the eCRF that are transcribed from source documents must be consistent with the source documents or the discrepancies must be explained. The investigator may need to request previous medical records or transfer records, depending on the study. Also, current medical records must be available.
- Definition of what constitutes source data can be found in the Source Data Identification Form.
- The investigator must maintain accurate documentation (source data) that supports the information entered in the eCRF.
- Study monitors will perform ongoing source data verification to confirm that data entered into the eCRF by authorized site personnel are accurate, complete, and verifiable from source documents; that the safety and rights of participants are being protected; and that the study is being conducted in accordance with the currently approved protocol and any other study agreements, ICH GCP, and all applicable regulatory requirements.

10.1.9 Study and Site Start and Closure

First Act of Recruitment

The study start date is the date on which the clinical study will be open for recruitment of participants.

The first act of recruitment is the first signed ICF and will be the study start date.

Study/Site Termination

The sponsor or designee reserves the right to close the study site or terminate the study at any time for any reason at the sole discretion of the sponsor. Study sites will be closed upon study completion. A study site is considered closed when all required documents and study supplies have been collected and a study site closure visit has been performed.

The investigator may initiate study site closure at any time, provided there is reasonable cause and sufficient notice is given in advance of the intended termination.

Reasons for the early closure of a study site by the sponsor or investigator may include but are not limited to:

For study termination:

- Discontinuation of further study intervention development.

For site termination:

- Failure of the investigator to comply with the protocol, the requirements of the IRB/IEC or local health authorities, the sponsor's procedures, or GCP guidelines
- Inadequate or no recruitment (evaluated after a reasonable amount of time) of participants by the investigator
- Total number of participants included earlier than expected.

If the study is prematurely terminated or suspended, the sponsor shall promptly inform the investigators, the IECs/IRBs, the regulatory authorities, and any contract research organization(s) used in the study of the reason for termination or suspension, as specified by the applicable regulatory requirements. The investigator shall promptly inform the participant and should assure appropriate participant therapy and/or follow-up.

10.1.10 Publication Policy

- The results of this study may be published or presented at scientific meetings. If this is foreseen, the investigator agrees to submit all manuscripts or abstracts to the sponsor before submission. This allows the sponsor to protect proprietary information and to provide comments.
- The sponsor will comply with the requirements for publication of study results. In accordance with standard editorial and ethical practice, the sponsor will generally support publication of multicenter studies only in their entirety and not as individual site data. In this case, a coordinating investigator will be designated by mutual agreement.
- Authorship will be determined by mutual agreement and in line with International Committee of Medical Journal Editors authorship requirements.

10.2 Appendix 2: Clinical Laboratory Tests

- The test detailed in Table 10-1 will be performed at the times indicated in SoA (see Section 1.3).
- Both local and central laboratories will be used.
- It is important that the samples for central analysis are obtained at the same time than the samples for the local laboratory.
- Additionally, local laboratory results used to make either a study intervention decision or response evaluation must be recorded.
- Protocol-specific requirements for inclusion or exclusion of participants are detailed in Section 5 of the protocol.
- Additional tests may be performed at any time during the study as determined necessary by the investigator or required by local regulations.

Table 10-1: Protocol-Required Laboratory Tests

Laboratory Tests	Parameters

Table 10-1: Protocol-Required Laboratory Tests

Laboratory Tests	Parameters			
Hematology	Platelet count	Red blood cell (RBC) indices:<br>MCV<br>MCH<br>MCHC		White blood cell (WBC) count
	RBC count			
	Hemoglobin			
	Hematocrit			
Clinical chemistry	Blood urea nitrogen (BUN)			Total and direct bilirubin
	Aspartate aminotransferase (AST)	Alanine aminotransferase (ALT)	Alkaline phosphatase (AP)	Gamma glutamyl transpeptidase (GGT)
	Lactate dehydrogenase (LDH)	Creatine kinase (CK)	Albumin	Total protein
	Creatinine	Cystatin C	eGFRcr (CKD-EPI creatinine, Levey et al. 2009) and eGFRcr-cys (CKD-EPI creatinine-cystatin C, Inker et al. 2021)	
	High density lipoprotein (HDL)	Low density lipoprotein cholesterol (LDL-C)	Total cholesterol	Triglycerides
	Potassium (K+)	Bicarbonates	Sodium	
	Glucose (fasting)	Glycated hemoglobin (HbA1c)[1]		
Urinalysis	• Urinary albumin-to-creatinine ratio (UACR)<br>• pH, glucose, protein, blood, ketones, by dipstick<br>• Microscopic examination (if blood or protein is abnormal)			
Pregnancy testing	• Highly sensitive serum human chorionic gonadotropin (hCG) pregnancy test (as needed for women of childbearing potential)			
Postmenopausal status confirmation[2]	• Follicle stimulating hormone (FSH)			
	All study-required laboratory assessments will be performed by a central laboratory, with the exception of:<br>• optional pre-screening UACR (local laboratory)<br>• K+ (both at central and local laboratories)<br>• estimated glomerular filtration rate (both at central and local laboratories)			

NOTES:
[1] At Day 1.
[2] A high FSH level in the postmenopausal range may be used to confirm a postmenopausal state in women not using hormonal contraception or HRT. However, in the absence of 12 months of amenorrhea, confirmation with more than one FSH measurement is required.

Investigators must document their review of each laboratory safety report.

10.2.1 Biomarkers

Biomarkers associated to the mode-of action of the study intervention and/or functional markers relevant to CKD and CV disease will be evaluated. Biomarkers include:

- Vasoactive agents (aldosterone, plasma renin activity, etc.), NT-proBNP/BNP, and sodium.
- 'De novo' PD biomarkers identified by OMICS/Multiplex Analysis which may indicate disease progression.
- Other biomarkers deemed relevant to kidney and CV diseases.

The correlation between the measurements of the above indicators and clinical outcomes will be described.

10.2.2 Pharmacokinetics

Finerenone and empagliflozin plasma concentrations might be analyzed in case of specific questions from the DMC, Steering Committee, or sponsor.

10.3 Appendix 3: Disease-Related Event Definitions

10.3.1 Myocardial Infarction

The term MI should be used when there is evidence of myocardial necrosis in a clinical setting consistent with myocardial ischemia.

In general, the diagnosis of MI requires the combination of (Thygesen et al. 2019):

1. Evidence of myocardial necrosis (either changes in cardiac biomarkers or postmortem pathological findings); and
2. Supporting information derived from the clinical presentation, electrocardiographic changes, or the results of myocardial or coronary artery imaging.

The totality of the clinical, electrocardiographic, and cardiac biomarker information should be considered to determine whether a MI has occurred. Specifically, timing and trends in cardiac biomarkers and electrocardiographic information require careful analysis. The diagnosis of MI should also consider the clinical setting in which the event occurs. MI may be diagnosed for an event that has characteristics of a MI, but which does not meet the strict definition because biomarker or electrocardiographic results are not available.

10.3.1.1 Criteria for Myocardial Infarction

Clinical Presentation

The clinical presentation should be consistent with diagnosis of myocardial ischemia and infarction. Other findings that might support the diagnosis of MI should be taken into account because a number of conditions are associated with elevations in cardiac biomarkers (e.g., trauma, surgery, pacing, ablation, congestive HF, hypertrophic cardiomyopathy, pulmonary embolism, severe pulmonary hypertension, stroke or subarachnoid hemorrhage, infiltrative and inflammatory disorders of cardiac muscle, drug toxicity, burns, critical illness, extreme exertion, and chronic kidney disease). Supporting information can also be considered from myocardial imaging and coronary imaging. The totality of the data may help differentiate acute MI (AMI) from the background disease process.

10.3.1.1.1 Criteria for Acute Myocardial Infarction

- Detection of rise and/or fall of cardiac biomarkers (preferably cardiac troponin [cTn]) with at least one value above the 99th percentile of the upper reference limit (URL) or

at least 1 value exceeding the local reference limit for non-highly sensitive methods together with evidence of myocardial ischemia with at least one of the following:

- Symptoms of ischemia
- ECG changes indicative of new ischemia (new ST-T changes or new left bundle branch block [LBBB])
- Development of pathological Q waves in the ECG
- Imaging evidence of new loss of viable myocardium or new regional wall motion abnormality
- Identification of an intracoronary thrombus by angiography.

- PCI related MI is arbitrarily defined by elevation of cTn values (>5 x 99th percentile URL) in participants with normal baseline values (≤99th percentile URL) or a rise of cTn values >20% if the baseline values are elevated and are stable or falling. In addition, either (i) symptoms suggestive of myocardial ischemia, or (ii) new ischemic ECG changes, or (iii) angiographic findings consistent with a procedural complication, or (iv) imaging demonstration of new loss of viable myocardium or new regional wall motion abnormality are required.
- Stent thrombosis associated with MI when detected by coronary angiography or autopsy in the setting of myocardial ischemia and with a rise and/or fall of cardiac biomarker values with at least one value above the 99th percentile URL.
- CABG related MI is arbitrarily defined by elevation of cardiac biomarker values (>10 x 99th percentile URL) in participants with normal baseline cTn values (≤99th percentile URL). In addition, either (i) new pathological Q waves or new LBBB, or (ii) angiographic documented new graft or new native coronary artery occlusion, or (iii) imaging evidence of new loss of viable myocardium or new regional wall motion abnormality.

10.3.1.1.2 Criteria for Prior Myocardial Infarction

- Pathological Q waves with or without symptoms in the absence of non-ischemic causes
- Imaging evidence of a region of loss of viable myocardium that is thinned and fails to contract in the absence of a non-ischemic cause
- Pathological findings of a healed or healing myocardium infarction.

10.3.1.1.3 Ischemic Symptoms

Ischemic symptoms are considered to be present if there is discomfort in the chest, upper extremity, mandibular or epigastric region lasting greater than 20 minutes. The discomfort is usually diffuse and may be accompanied by diaphoresis, nausea, or syncope. Atypical symptoms may also occur with an MI such as palpitations or cardiac arrest.

10.3.1.1.4 Cardiac Markers

All pertinent enzyme results (cTn T and I, creatine kinase (CK), CK-MB (CK-myocardial band), lactate dehydrogenase, and others) available will be reviewed.

The preferred biomarker for myocardial necrosis is cTn (I or T), which has nearly absolute myocardial tissue specificity as well as high clinical sensitivity.

An increased value for cTn is defined as a measurement exceeding the 99th percentile of a normal reference population (URL). If troponin assays are not available, the best alternative is CK-MB (measured by mass assay). As with troponin, an increased CK-MB value is defined as a measurement above the 99th percentile URL.

These markers must be evaluated in the absence of known non-ischemic cause.

Enzymes are classified as "incomplete" if no enzyme data (or not enough data) are available to allow classification.

10.3.1.1.5 ECG Changes

ECG changes can be used to support or confirm a MI. Supporting evidence may be ischemic changes and confirmatory information may be new Q waves.

ECG Manifestations of Acute Myocardial Ischemia (in Absence of Left Ventricular Hypertrophy and LBBB)

ST elevation

- New ST elevation at the J point in 2 anatomically contiguous leads with the cut-points: ≥0.2 mV in men ≥40 years (≥0.25 mV in men <40 years) or ≥0.15 mV in women in leads V2-V3 and/or ≥0.1 mV in other leads.

ST depression and T-wave changes

- New horizontal or down-sloping ST depression ≥0.05 mV in 2 contiguous leads and/or new T inversion ≥0.1 mV in 2 contiguous leads with prominent R-wave or R/S ratio >1.

The above ECG criteria illustrate patterns consistent with myocardial ischemia. In patients with abnormal biomarkers, it is recognized that lesser ECG abnormalities may represent an ischemic response and may be accepted under the category of abnormal ECG findings.

ECG Changes Associated with Prior MI

- Any Q-wave in leads V2-V3 ≥0.02 seconds or QS complex in leads V2 and V3
- Q-wave ≥0.03 seconds and ≥0.1 mV deep or QS complex in leads I, II, aVL, aVF, or V4-V6 in any 2 leads of a contiguous lead grouping (I, aVL, V1-V6; II, III, and aVF)
- The same criteria are used for supplemental leads V7-V9, and for the Cabrera frontal plane lead grouping
- R-wave ≥0.04 seconds in V1-V2 and R/S ≥1 with a concordant positive T-wave in the absence of a conduction defect.

10.3.2 Stroke

Stroke is defined as an acute episode of focal or global neurological dysfunction caused by brain, spinal cord, or retinal vascular injury as a result of hemorrhage or infarction, with symptom duration of 24 hours or more. Episodes lasting less than 24 hours can be considered a stroke if there is an intervention to abort the stroke (e.g., thrombolytic therapy), diagnostic confirmation of the stroke, or participant death prior to reaching the 24-hour duration.

Subdural hematomas are intracranial hemorrhagic events and not strokes.

10.3.2.1 Ischemic Stroke

Ischemic stroke is defined as an acute episode of focal cerebral, spinal, or retinal dysfunction caused by an infarction of central nervous system tissue.

Hemorrhage may be a consequence of ischemic stroke. In this situation, the stroke is an ischemic stroke with hemorrhagic transformation and not a hemorrhagic stroke.

10.3.2.2 Hemorrhagic Stroke

Hemorrhagic stroke is defined as an acute episode of focal or global cerebral or spinal dysfunction caused by an intraparenchymal, intraventricular, or subarachnoid hemorrhage.

10.3.2.3 Undetermined Stroke

Undetermined stroke is defined as an acute episode of focal or global neurological dysfunction caused by presumed brain, spinal cord, or retinal vascular injury as a result of hemorrhage or infarctions but with insufficient information to allow categorization as Ischemic or Hemorrhagic.

Note: Given the scope of this study, disability will not be measured.

10.3.3 New Onset of HF

This definition will be used to determine if cases referred to by investigators as HF meet criteria for new onset of HF.

New onset of HF is defined as an event that meets ALL the following criteria:

1. The participant does NOT have a prior history of HF documented.
2. The participant must have all criteria #2 to #4 as noted below in Section 10.3.4.
3. The participant receives initiation of treatment specifically for HF; criteria #5, as indicated in Section 10.3.4 with the difference that the route of administration could be oral and/or intravenous.

10.3.4 Hospitalization for HF

HF requiring hospitalization is defined as an event that meets ALL the following criteria:

1. The participant is admitted to the hospital with a primary diagnosis of HF
2. The participant's length of stay in hospital extends for at least 24 hours (or a change in calendar date if the hospital admissions and discharge times are unavailable)
3. The participant exhibits documented new symptoms or worsening symptoms due to HF on presentation, including at least ONE of the following:
    a. Dyspnea (dyspnea with exertion, dyspnea at rest, orthopnea, paroxysmal nocturnal dyspnea)
    b. Decreased exercise tolerance
    c. Fatigue
    d. Other symptoms of worsened end-organ perfusion or volume overload end-organ perfusion and volume overload will be clinically determined by the DMC members since these definitions are not protocol defined
4. The participant has objective evidence of worsening HF, consisting of at least TWO physical examination findings OR ONE physical examination finding and at least ONE laboratory criterion, including:

   a. Physical examination findings considered to be due to HF, including new or worsened:
      i. Peripheral edema
      ii. Increasing abdominal distention or ascites (in the absence of hepatic disease)
      iii. Pulmonary rales/crackles/crepitations
      iv. Increased jugular venous pressure and/or hepatojugular reflux
      v. S3 gallop
      vi. Clinically significant or rapid weight gain thought to be related to fluid retention
   b. Laboratory evidence of new or worsening HF, if obtained within 24 hours of presentation, including:
      i. Increased BNP/N-terminal pro-BNP (NT-pro BNP) or mid-regional pro-atrial natriuretic peptide concentrations consistent with decompensation of HF. In participants with chronically elevated natriuretic peptides, a significant increase should be noted above baseline
      ii. Radiological evidence of pulmonary congestion
      iii. Non-invasive or invasive diagnostic evidence of clinically significant elevated left or right sided ventricular filling pressure or low cardiac output. For example, echocardiographic criteria could include E/e >15 or D-dominant pulmonary venous inflow pattern, plethoric inferior vena cava with minimal collapse on inspiration, or decreased left ventricular outflow tract minute stroke distance (time velocity integral) OR right heart catheterization showing a pulmonary capillary wedge pressure (pulmonary artery occlusion pressure) >18 mmHg, central venous pressure >12 mmHg, or a cardiac index <2.2 L/min/m^2
5. The participant receives initiation or intensification of treatment specifically for HF, including **at least ONE** of the following:
   a. Intravenous diuretic, inotrope, or vasodilator therapy
   b. Mechanical or surgical intervention, including:
      i. Mechanical circulatory support (e.g., intra-aortic balloon pump, ventricular assist device)
      ii. Mechanical fluid removal (e.g., ultrafiltration, hemofiltration, dialysis).

10.3.5 Chronic Sustained Decrease in EGFR

Central laboratory values will be used for the definitions described below. Local laboratory values will not be considered. The eGFR from Day 1 will be considered as the baseline value. In cases where the eGFR from Day 1 is missing, the last value measured prior to randomization will be considered as the baseline value.

A chronic sustained decrease in eGFR is defined as a decrease in eGFR (≥40% or ≥57% compared to baseline [Day 1], or to <15mL/min/1.73m^2), observed over at least 4 weeks with at least 2 consecutive central laboratory assessments confirming the decrease.

The confirmatory sample is expected to be collected at least 4 weeks after the initial assessment showing decrease of x% or more. The date of onset of sustained decrease in eGFR ≥x% compared to baseline is the date of the initial sample exceeding the threshold.
If the participant has an initial decrease in eGFR at the EOS visit or before the EOS which has not been confirmed yet at the time of EOS visit, it should be confirmed after 4 weeks from the initial decrease.

10.4 Appendix 4: AEs and SAEs: Definitions and Procedures for Recording, Evaluating, Follow-Up, and Reporting

10.4.1 Definition of AE

AE Definition
• An AE is any untoward medical occurrence in a clinical study participant, associated with the use of study intervention, whether or not considered related to the study intervention.<br>• NOTE: An AE can therefore be any unfavorable and unintended sign (including an abnormal laboratory finding), symptom, or disease (new or exacerbated) associated with the use of study intervention.

Events Meeting the AE Definition
• Any abnormal laboratory test results (hematology, clinical chemistry, or urinalysis) or other safety assessments (e.g., ECG, radiological scans, vital signs measurements), including those that worsen from baseline, considered clinically significant in the medical and scientific judgment of the investigator (i.e., not related to progression of underlying disease).<br>• Exacerbation of a chronic or intermittent pre-existing condition including either an increase in frequency and/or intensity of the condition.<br>• New conditions detected or diagnosed after study intervention administration even though it may have been present before the start of the study.<br>• Signs, symptoms, or the clinical sequelae of a suspected intervention-intervention interaction.<br>o Signs, symptoms, or the clinical sequelae of a suspected overdose of either study intervention or a concomitant medication. Overdose per se will not be reported as an AE/SAE unless it is an intentional overdose taken with possible suicidal/self-harming intent. Such overdoses should be reported regardless of sequelae.<br>o Lack of efficacy or failure of expected pharmacological action per se will not be reported as an AE or SAE. Such instances will be captured in the efficacy assessments. However, the signs, symptoms, and/or clinical sequelae resulting from lack of efficacy will be reported as AE or SAE if they fulfill the definition of an AE or SAE.

Events NOT Meeting the AE Definition

- Any clinically significant abnormal laboratory findings or other abnormal safety assessments which are associated with the underlying disease, unless judged by the investigator to be more severe than expected for the participant's condition.
- The disease/disorder being studied or expected progression, signs, or symptoms of the disease/disorder being studied, unless more severe than expected for the participant's condition.
- Medical or surgical procedure (e.g., endoscopy, appendectomy): the condition that leads to the procedure is the AE.
- Situations in which an untoward medical occurrence did not occur (social and/or convenience admission to a hospital).
- Anticipated day-to-day fluctuations of pre-existing disease(s) or condition(s) present or detected at the start of the study that do not worsen.

10.4.2 Definition of SAE

An SAE is defined as any AE that, at any dose:

a. Results in death

b. Is life-threatening

- The term 'life-threatening' in the definition of 'serious' refers to an event in which the participant was at risk of death at the time of the event. It does not refer to an event, which hypothetically might have caused death, if it were more severe.

c. Requires inpatient hospitalization or prolongation of existing hospitalization

- In general, hospitalization signifies that the participant has been admitted (usually involving at least an overnight stay) at the hospital or emergency ward for observation and/or treatment that would not have been appropriate in the physician's office or outpatient setting. Complications that occur during hospitalization are AEs. If a complication prolongs hospitalization or fulfills any other serious criteria, the event is serious. When in doubt as to whether "hospitalization" occurred or was necessary, the AE should be considered serious.
- Hospitalization for elective treatment of a pre-existing condition that did not worsen from baseline is not considered an AE.

d. Results in persistent or significant disability/incapacity

- The term disability means a substantial disruption of a person's ability to conduct normal life functions.
- This definition is not intended to include experiences of relatively minor medical significance such as uncomplicated headache, nausea, vomiting, diarrhea, influenza, and accidental trauma (e.g., sprained ankle) which may interfere with or prevent everyday life functions but do not constitute a substantial disruption.

e. Is a congenital anomaly/birth defect

f. Other situations:

- Medical or scientific judgment should be exercised by the investigator in deciding whether SAE reporting is appropriate in other situations such as significant medical events that may jeopardize the participant or may require medical or surgical intervention to prevent one of the other outcomes listed in the above definition. These events should usually be considered serious.
- Examples of such events include invasive or malignant cancers, intensive treatment for allergic bronchospasm, blood dyscrasias, convulsions, or development of intervention dependency or intervention abuse.

10.4.3 Recording and Follow-Up of AE and/or SAE

AE and SAE Recording

- When an AE/SAE occurs, it is the responsibility of the investigator to review all documentation (e.g., hospital progress notes, laboratory reports, and diagnostics reports) related to the event.
- The investigator will then record all relevant AE/SAE information.
- It is not acceptable for the investigator to send photocopies of the participant's medical records to the medical monitor in lieu of completion of the AE/SAE CRF required form.
    - There may be instances when copies of medical records for certain cases are requested by the sponsor. In this case, all participant identifiers, with the exception of the participant number, will be redacted on the copies of the medical records before submission to the sponsor.
    - The investigator will attempt to establish a diagnosis of the event based on signs, symptoms, and/or other clinical information. Whenever possible, the diagnosis (not the individual signs/symptoms) will be documented as the AE/SAE.

Assessment of Intensity

- The investigator will make an assessment of intensity for each AE and SAE reported during the study and assign it to 1 of the following categories:
    - Mild: An event that is easily tolerated by the participant, causing minimal discomfort and not interfering with everyday activities.
    - Moderate: An event that causes sufficient discomfort to interfere with normal everyday activities.
    - Severe: An event that prevents normal everyday activities. An AE that is assessed as severe should not be confused with an SAE. Severe is a category utilized for rating the intensity of an event; and both AEs and SAEs can be assessed as severe.
    - An event is defined as 'serious' when it meets at least 1 of the predefined outcomes as described in the definition of an SAE, NOT when it is rated as severe.

Assessment of Causality

- The investigator is obligated to assess the relationship between study intervention and each occurrence of each AE/SAE.
- A "reasonable possibility" of a relationship conveys that there are facts, evidence, and/or arguments to suggest a causal relationship, rather than a relationship cannot be ruled out.
- The investigator will use clinical judgment to determine the relationship.
- Alternative causes, such as underlying disease(s), concomitant therapy, and other risk factors, as well as the temporal relationship of the event to study intervention administration will be considered and investigated.
- The investigator will also consult the IB and/or Product Information, for marketed products, in his/her assessment.
- For each AE/SAE, the investigator **must** document in the medical notes that he/she has reviewed the AE/SAE and has provided an assessment of causality.
- There may be situations in which an SAE has occurred, and the investigator has minimal information to include in the initial report to the sponsor. However, **it is very important that the investigator always make an assessment of causality for every event before the initial transmission** of the SAE data to the sponsor.
- The investigator may change his/her opinion of causality in light of follow-up information and send an SAE follow-up report with the updated causality assessment.
- The causality assessment is one of the criteria used when determining regulatory reporting requirements.

Follow-up of AEs and SAEs

- The investigator is obligated to perform or arrange for the conduct of supplemental measurements and/or evaluations as medically indicated or as requested by the sponsor to elucidate the nature and/or causality of the AE or SAE as fully as possible. This may include additional laboratory tests or investigations, histopathological examinations, or consultation with other health care professionals.
- If a participant dies during participation in the study or during a recognized follow-up period, the investigator will provide the sponsor with a copy of any post-mortem findings including histopathology.
- New or updated information will be recorded in the originally submitted documents.
- The investigator will submit any updated SAE data to the sponsor immediately and no later than 24 hours of receipt of the information.

10.4.4 Reporting of SAEs

SAE Reporting to the sponsor via an Electronic Data Collection Tool

- The primary mechanism for reporting an SAE to the sponsor will be the electronic data collection tool.
- If the electronic system is unavailable, then the site will use the paper SAE data

transmission (see next section) to report the event within 24 hours.

- The site will enter the SAE data into the electronic system as soon as it becomes available.
- After the study is completed at a given site, the electronic data collection tool will be taken off-line to prevent the entry of new data or changes to existing data.
- If a site receives a report of a new SAE from a study participant or receives updated data on a previously reported SAE after the electronic data collection tool has been taken off-line, then the site can report this information on a paper SAE form (see next section) or to the sponsor medical monitor by telephone.
- Contacts for SAE reporting can be found in the investigator site file.

**SAE Reporting to the sponsor via Paper Data Collection Tool**
• Email transmission of the SAE paper data collection tool is the preferred method to transmit this information to the sponsor medical monitor.<br>• In rare circumstances and if email transmission is not feasible, notification by telephone is acceptable with a copy of the SAE data collection tool sent by overnight mail or courier service.<br>• Initial notification via telephone does not replace the need for the investigator to complete and sign the SAE data collection tool within the designated reporting time frames.<br>• Contacts for SAE reporting can be found in the investigator site file.

10.5 Appendix 5: Death Events Definition

10.5.1 Cardiovascular Death

CV death includes death resulting from an AMI, sudden cardiac death, undetermined death, death due to HF, death due to stroke, death due to CV procedures, and death due to other CV causes, as follows:

10.5.1.1 Death due to Acute Myocardial Infarction

Death due to AMI refers to a death by any CV mechanism (e.g., arrhythmia, sudden death, HF, stroke, pulmonary embolus, peripheral artery disease) within 30 days after an MI and related to the immediate consequences of the MI, such as progressive HF or recalcitrant arrhythmia. AMI should be verified to the extent possible by the diagnostic criteria outlined for AMI or by autopsy findings showing recent MI or recent coronary thrombosis.

Death resulting from a procedure to treat a MI (PCI, CABG), or to treat a complication resulting from MI should also be considered death due to AMI.

Death resulting from an elective coronary procedure to treat myocardial ischemia (i.e., chronic stable angina) or death due to an MI that occurs as a direct consequence of a CV investigation/procedure/operation should be considered as a death due to a CV procedure.

Note: If within the 30 days following a MI event, the participant does not die, or dies from a cause that is not directly related to the MI event, then the MI will be classified as non-fatal.

10.5.1.2 Sudden Cardiac Death

Sudden Cardiac Death refers to a death that occurs unexpectedly, not following an AMI, (i.e., not within 30 days of an AMI) and includes the following deaths:

- Death witnessed and instantaneous without new or worsening symptoms
- Death witnessed within 1 hour of the onset of new or worsening cardiac symptoms, unless the symptoms suggest AMI
- Death witnessed and attributed to an identified arrhythmia (e.g., captured on an electrocardiographic [ECG] recording, witnessed on a monitor, or unwitnessed but found on implantable cardioverter-defibrillator review)
- Death after unsuccessful resuscitation from cardiac arrest
- Death after successful resuscitation from cardiac arrest and without identification of a specific etiology
- Unwitnessed death in a participant seen alive and clinically stable ≤24 hours prior to being found dead without any evidence supporting a specific non-CV cause of death (information regarding the participant's clinical status preceding death should be provided, if available).

10.5.1.3 Undetermined Death

- For participants who were NOT observed alive within 24 hours of death and without any other likely cause of death, undetermined cause of death should be recorded (e.g., a participant found dead in bed but who had not been seen by family members for >24 hours).
- Considering the targeted participant population and the competing causes of death, undetermined cause of death will by default to CV death.

10.5.1.4 Death due to Heart Failure

Death due to HF or cardiogenic shock refers to a death in association with clinically worsening symptoms and/or signs of HF without evidence of another cause and NOT following an AMI. Deaths due to HF can have various etiologies, including single or recurrent MIs (late effect, i.e., >30 days), ischemic or non-ischemic cardiomyopathy, hypertension, or valvular disease. There is no other identified cause of death other than HF.

Death due to HF or cardiogenic shock should include sudden death occurring during an admission for worsening HF as well as death from progressive HF or cardiogenic shock following implantation of a mechanical assist device.

No evidence of AMI or stroke in the previous 30 days.

New or worsening signs and/or symptoms of HF include any of the following:

- New or increasing symptoms and/or signs of HF requiring the initiation of, or an increase in, treatment directed at HF or occurring in a participant already receiving maximal therapy for HF
- HF symptoms or signs requiring intravenous therapy or chronic oxygen administration for hypoxia due to pulmonary edema
- Confinement to bed predominantly due to HF symptoms

- Pulmonary edema sufficient to cause tachypnea and distress not occurring in the context of an AMI, worsening renal function, or as the consequence of an arrhythmia
- Cardiogenic shock not occurring in the context of an AMI or as the consequence of an arrhythmia.

Cardiogenic shock is defined as SBP <90 mm Hg for greater than 1 hour, not responsive to fluid resuscitation and/or heart rate correction, and felt to be secondary to cardiac dysfunction and associated with at least one of the following signs of hypoperfusion:

- Cool, clammy skin or
- Oliguria (urine output <30 mL/hour) or
- Altered sensorium or
- Cardiac index <2.2 L/min/m^2.

Cardiogenic shock can also be defined if SBP <90 mm Hg and increases to ≥ 90 mm Hg in less than 1 hour with positive inotropic or vasopressor agents alone and/or with mechanical support.

For diagnosis of death due to HF, it will be evaluated whether new onset of HF criteria were also met, as indicated in Section 10.3.3.

10.5.1.5 Death due to Stroke

Death due to stroke refers to death within 30 days after a stroke that is either a direct consequence of the stroke or a complication of the stroke. Acute stroke should be verified to the extent possible by the diagnostic criteria outlined for stroke.

Note: If within the 30 days following a stroke event, the participant does not die, or dies from a cause that is not directly related to the stroke event, then the stroke will be classified as non-fatal.

10.5.1.6 Death due Cardiovascular Procedures

Death due to CV procedures refers to death within 30 days caused by the immediate complications of a CV procedure.

10.5.1.7 Death due to Other Cardiovascular Causes

Death due to other CV causes refers to a CV death not included in the above categories (e.g., CV hemorrhage, pulmonary embolism, or peripheral arterial disease). Non-stroke intracranial hemorrhage, non-procedural or non-traumatic vascular rupture (e.g., aortic rupture) or hemorrhage causing cardiac tamponade are considered CV hemorrhages. Any other bleeding should be considered as non-CV.

Note: Death due to CV hemorrhage refers to death related to hemorrhage such as a non-stroke intracranial hemorrhage, non-procedural or non-traumatic vascular rupture (e.g., aortic aneurysm), or hemorrhage causing cardiac tamponade. Bleeding that does not fall under the mentioned conditions will be considered as non-CV event.

10.5.2 Renal Death

The following events will be classified as renal death when they satisfy the following criteria:

1. The participant dies

AND

2. Renal replacement therapy (RRT) has not been initiated (although clinically indicated, e.g., death due to progressive kidney failure occurs before RRT can be introduced)
   a. If a participant has advanced kidney failure and is denied dialysis or refuses dialysis and dies, then death is eligible to be called renal death
   b. If there is a reason that the participant was denied RRT in the first place, then another more proximal cause of death will be identified. As examples:
      i. Cancer death if the participant refused dialysis due to metastatic cancer
      ii. CV death, because the participant was in shock and the participant did not want dialysis to be done would not be called renal death
      iii. Infection death (e.g., septic shock)

AND

3. There is no likely other cause of death.

Note: In a participant who is already on dialysis and decides to withdraw from dialysis, death is due to withdrawal of dialysis. Such a participant did not die because lack of initiation of dialysis therefore this is not called "renal death" in this study.

General Considerations

The RRT, that is not initiated, must be directly related to the kidney failure to be considered for renal death definition. RRT, that is not initiated, related to another disease condition (e.g., volume overload) and unrelated to kidney failure should NOT be considered in renal death definition. The proximal cause of death must be directly related to kidney failure.

10.5.3 Non-Cardiovascular and Non-Renal Deaths

Non-CV and non-renal death are defined as any death that is not thought to be due to a CV cause or renal cause. Non-CV and non-renal causes of death will be categorized as follows:

- Infection
- Malignancy
- Other (specify).

10.6 Appendix 6: Contraceptive Guidance and Collection of Pregnancy Information

Definitions:

Woman of Childbearing Potential (WOCBP)

A woman is considered fertile following menarche and until becoming postmenopausal unless permanently sterile (see below).

If fertility is unclear (e.g., amenorrhea in adolescents or athletes) and a menstrual cycle cannot be confirmed before first dose of study intervention, additional evaluation should be considered.

Women in the following categories are not considered WOCBP

1. Premenarchal
2. Premenopausal female with 1 of the following:
   - Documented hysterectomy
   - Documented bilateral salpingectomy
   - Documented bilateral oophorectomy.

   For individuals with permanent infertility due to an alternate medical cause other than the above, (e.g., Mullerian agenesis, androgen insensitivity), investigator discretion should be applied to determining study entry.

   Note: Documentation can come from the site personnel's: review of the participant's medical records, medical examination, or medical history interview.
3. Postmenopausal female
   - A postmenopausal state is defined as no menses for 12 months without an alternative medical cause.
     - A high FSH level in the postmenopausal range may be used to confirm a postmenopausal state in women not using hormonal contraception or HRT. However, in the absence of 12 months of amenorrhea, confirmation with more than 1 FSH measurement is required.
   - Females on HRT and whose menopausal status is in doubt will be required to use one of the non-estrogen hormonal highly effective contraception methods if they wish to continue their HRT during the study. Otherwise, they must discontinue HRT to allow confirmation of postmenopausal status before study enrollment.

Contraception Guidance:

WOCBP can only be included in the study if a pregnancy test is negative at the screening visit and if they agree to use adequate contraception during the study and until 8 weeks after last study interventions dose. Adequate contraception is defined as any combination of at least 2 effective methods of birth control, of which at least one is a physical barrier (e.g., condoms with hormonal contraception or implants or combined oral contraceptives, certain intrauterine devices).

Collection of Pregnancy Information for Female Participants who Become Pregnant:

- The investigator will collect pregnancy information on any female participant who becomes pregnant while participating in this study. The initial information will be recorded on the appropriate form and submitted to the sponsor within 24 hours of learning of a participant pregnancy.
- The participant will be followed to determine the outcome of the pregnancy. The investigator will collect follow-up information on the participant and the neonate, and the information will be forwarded to the sponsor. Generally, follow-up will not be required for longer than 6 to 8 weeks beyond the estimated delivery date. Any termination of pregnancy will be reported, regardless of fetal status (presence or absence of anomalies) or indication for the procedure.

- While pregnancy itself is not considered to be an AE or SAE, any pregnancy complication or elective termination of a pregnancy for medical reasons will be reported as an AE or SAE.
- A spontaneous abortion (occurring at <22 weeks' gestational age) or still birth (occurring at >22 weeks' gestational age) is always considered to be an SAE and will be reported as such.
  - Any post-study pregnancy-related SAE considered reasonably related to the study intervention by the investigator will be reported to the sponsor as described in Section 8.3.4. While the investigator is not obligated to actively seek this information in former study participants, he or she may learn of an SAE through spontaneous reporting.
  - Any female participant who becomes pregnant while participating in the study will discontinue study intervention and be withdrawn from the study after completing ED visit.

10.7 Appendix 7: Calculating the Child Pugh Score

The severity of liver disease (Table 10-2) will determine the Child Pugh score (Table 10-3).

Table 10-2: Grading of Severity of Liver Disease (adapted from *Pugh et al., 1973*)

Factor	+1	+2	+3
**Bilirubin (mg/dL)**	<2	2 – 3	>3
**Albumin (g/dL)**	>3.5	2.8 – 3.5	<2.8
**International Normalized Ratio**	<1.7	1.7 – 2.3	>2.3
**Ascites**	None	Mild	Moderate/Severe
**Encephalopathy**	None	Grade I - II	Grade III - IV

Table 10-3: Classification Using the Added Score from Table 10-2

Child-Pugh Class	A	B	C
**Points**	5 – 6	7 - 9	10 - 15

10.8 Appendix 8: Guidance on Use of Common CYP Inhibitors and Inducers

Table 10-4 lists the most common medication regarded as potent CYP3A4 inhibitors or inducers.

Table 10-4: Cytochrome P450: List of Concomitant Medication

Excluded Cytochrome P450 Isoenzyme 3A4 (CYP3A4) inducers	Excluded CYP3A4 inhibitors	Allowed CYP3A4 inhibitors
apalutamide<br>asunaprevir/beclabuvir/daclatasvir<br>avasimibe<br>bosentan<br>carbamazepine<br>cenobamate<br>dabrafenib<br>efavirenz<br>elagolix<br>enzalutamide<br>etravirine<br>fosphenytoin<br>hypericum perforatum / St John's wort<br>ivosidenib<br>lersivirine<br>lesinurad<br>lorlatinib<br>lumacaftor<br>mephenytoin<br>metamizole<br>methylphenobarbital<br>mitotane<br>modafinil<br>nafcillin<br>nevirapine<br>oxcarbazepine<br>pexidartinib<br>phenobarbital<br>phenytoin<br>primidone<br>rifabutin<br>rifapentin<br>rifampicin<br>semagacestat<br>sotorasib<br>talviraline<br>telotristat ethyl<br>thioridazine<br>troglitazone<br>vemurafenib	amprenavir<br>atazanavir and other inhibitors of human HIV protease<br>boceprevir<br>ceritinib<br>clarithromycin<br>cobicistat<br>danoprevir<br>darunavir<br>dasabuvir<br>elvitegravir<br>fosamprenavir<br>grapefruit juice<br>idelalisib<br>indinavir<br>itraconazole<br>jasomycin<br>ketoconazole<br>lonafarnib<br>lopinavir<br>nefazodone<br>nelfinavir<br>ombitasvir<br>paritaprevir<br>posaconazole<br>ribociclib<br>ritonavir<br>saquinavir<br>telaprevir<br>telithromycin<br>tipranavir<br>troleandomycin<br>tucatinib<br>voriconazole	amlodipine<br>amiodarone<br>aprepitant<br>berotralstat<br>bicalutamide<br>chloramphenicol<br>cilostazol<br>cimetidine<br>ciprofloxacin<br>conivaptan<br>crizotinib<br>cyclosporine<br>diltiazem<br>dronedarone<br>duvelisib<br>erythromycin<br>fluconazole<br>fluvoxamine<br>fosaprepitant<br>imatinib<br>isavuconazole<br>istradefylline<br>ivacaftor<br>letermovir<br>lomitapide<br>mifepristone<br>norfloxacine<br>ranitidine<br>ranolazine<br>rimegepant<br>tacrolimus<br>ticagrelor<br>tofisopam<br>verapamil<br>lapatinib<br>dasatinib<br>nilotinib<br>voxelotor

HIV = human immunodeficiency virus

10.9 Appendix 9: Abbreviations

ABPM	Ambulatory blood pressure monitoring
ACEi	Angiotensin-converting enzyme inhibitor
ADA	American Diabetes Association
AE	Adverse event
AKI	Acute kidney injury
ALT	Alanine aminotransferase
AMI	Acute myocardial infarction
ARB	Angiotensin receptor blocker
ARNI	Angiotensin receptor-neprilysin inhibitor
AST	Aspartate aminotransferase
ATC	Anatomical therapeutic chemical
$AUC_{\tau,md}$	Area under the concentration vs. time curve for the expected dosing interval obtained after multiple dose administration
BMI	Body mass index
BNP	B-type natriuretic peptide
BP	Blood pressure
$C_{max,md}$	Maximum drug concentration after multiple dose administration
CABG	Coronary artery bypass graft
CI	Confidence interval
CK	Creatinine kinase
CKD	Chronic kidney disease
CKD-EPI	Chronic Kidney Disease Epidemiology Collaboration
CK-MB	Creatinine kinase-myocardial band
COVID-19	Coronavirus disease 2019
CRF	Case report form
cTn	Cardiac troponin
CV	Cardiovascular
CVD	Cardiovascular disease
CYP3A4	Cytochrome P450 isoenzyme 3A4
DBP	Diastolic blood pressure
DMC	Data Monitoring Committee
DRE	Disease-related event
ECG	Electrocardiogram
eCRF	Electronic Case Report Form
ED	Early discontinuation
e.g.	Exempli gratia, for example
eGFR	Estimated glomerular filtration rate
eGFRcr	Estimated glomerular filtration rate based on creatinine
eGFRcr-cys	Estimated glomerular filtration rate based on creatinine and cystatin C
EOS	End of study
ESKD	End-stage kidney disease
EU	European Union
FAS	Full analysis set
FDA	Food and Drug Administration
FSH	Follicle stimulating hormone
GCP	Good Clinical Practice
GFR	Glomerular filtration rate
HbA1c	Glycated hemoglobin
HF	Heart failure
HFrEF	Heart failure with reduced ejection fraction
HRT	Hormonal replacement therapy
IB	Investigator’s Brochure
ICF	Informed consent form
ICH	International Council for Harmonization
i.e.	Id est, that is
IEC	Independent Ethics Committees

IRB	Institutional Review Board
IWRS	Interactive web response system
K+	Serum/plasma potassium or potassium
LBBB	Left bundle branch block
MedDRA	Medical Dictionary for Regulatory Activities
MI	Myocardial infarction
MR	Mineralocorticoid receptor
MRA	Mineralocorticoid receptor antagonist
NT-proBNP	N-terminal prohormone B-type natriuretic peptide
OD	Once daily
PCI	Percutaneous coronary intervention
PD	Pharmacodynamic(s)
PPS	Per-protocol analysis set
PT	Preferred Term
QTL	Quality tolerance limit
RAASi	Renin-angiotensin-aldosterone system inhibitors
RRT	Renal replacement therapy
SAC	Statistical analysis center
SAE	Serious adverse event
SAF	Safety analysis set
SAP	Statistical analysis plan
SARS-CoV-2	Severe acute respiratory syndrome coronavirus 2
SBP	Systolic blood pressure
SC	Steering Committee
SDv	Standard deviation
SGLT2i	Sodium/glucose cotransporter-2 inhibitor(s)
SGLT-1/2i	Combined SGLT-1 and 2 inhibitor(s)
SMQ	Standardized MedDRA queries
SoA	Schedule of activities
SoC	Standard of care
SOC	System Organ Class
SUSAR	Suspected unexpected serious adverse reaction
T1D	Type 1 diabetes
T2D	Type 2 diabetes
TEAE	Treatment-emergent adverse event
TZD	Thiazolidinediones
UACR	Urinary albumin-to-creatinine ratio
ULN	Upper limit of normal
URL	Upper reference limit
US	United States (of America)
WHO-DD	World Health Organization Drug Dictionary

11. References

Afkarian M, Sachs MC, Kestenbaum B, et al. Kidney disease and increased mortality risk in type 2 diabetes. J Am Soc Nephrol 2013; 24: 302–308.

Agarwal R, Anker SD, Bakris G, et al. Investigating new treatment opportunities for patients with chronic kidney disease in type 2 diabetes: the role of finerenone. Nephrol Dial Transplant 2020 Dec 6:gfaa294. doi: 10.1093/ndt/gfaa294. Epub ahead of print. PMID: 33280027.

Agarwal R, Kolkhof P, Bakris G, et al. Steroidal and non-steroidal mineralocorticoid receptor antagonists in cardiorenal medicine. European Heart Journal 2021; Jan 7; 42(2): 152-161.

Agiostratidou G, Anhalt H, Ball D, et al. Standardizing clinically meaningful outcome measures beyond HbA1c for type 1 diabetes: a consensus report of

the American Association of Clinical Endocrinologists, the American Association of Diabetes Educators, the American Diabetes Association, the Endocrine Society, JDRF International, The Leona M. and Harry B. Helmsley Charitable Trust, the Pediatric Endocrine Society, and the T1D Exchange. Diabetes Care 2017; 40: 1622–1630.

American Diabetes Association. 2. Classification and Diagnosis of Diabetes: Standards of Medical Care in Diabetes - 2021. Diabetes Care 2021;44(Supplement 1): S15-S33.

American Diabetes Association. Microvascular complications and foot care: Standards of Medical Care in Diabetes - 2021. Diabetes Care 2021;44(Supplement 1): S151-S167.

Bae JH, Park EG, Kim S, et al. Effects of sodium-glucose cotransporter 2 inhibitors on renal outcomes in patients with type 2 diabetes: A systematic review and meta-analysis of randomised controlled trials. Sci Rep 2019;9, 13009.

Bakris GL, Agarwal R, Anker SD, et al. Effect of finerenone on chronic kidney disease outcomes in type 2 diabetes. N Engl J Med 2020; 383: 2219-2229.

Bakris GL, Agarwal R, Chan JC, et al. Effect of finerenone on albuminuria in patients with diabetic nephropathy: a randomised clinical trial. JAMA 2015; 314 (9): 884-894.

Cherney D, Lund SS, Perkins BA, et al. The effect of sodium glucose cotransporter 2 inhibition with empagliflozin on microalbuminuria and macroalbuminuria in patients with type 2 diabetes. Diabetologia 2016; 59 (9): 1860-1870.

Cherney D, Zinman B, Inzucchi SE, et al. Effects of empagliflozin on the urinary albumin-to-creatinine ratio in patients with type 2 diabetes and established cardiovascular disease: an exploratory analysis from the EMPA-REG OUTCOME randomised, placebo-controlled trial. The Lancet Diabetes & Endocrinology 2017; 5(8): 610-621.

Cohen JB, Yang W, Li L, et al. Time-Updated Changes in Estimated GFR and Proteinuria and Major Adverse Cardiac Events: Findings from the Chronic Renal Insufficiency Cohort (CRIC) Study. Am J Kidney Dis. 2021; S0272-6386(21)00629-6.

DeFronzo, RA, Lewin A, Patel S, et al. Combination of empagliflozin and linagliptin as second-line therapy in participants with type 2 diabetes inadequately controlled on metformin. Diabetes Care 2015; 38(3): 384.

Fernandez B, Elewa U, Sanchez-Nino MD, et al. 2012 update on diabetic kidney disease: the expanding spectrum, novel pathogenic insights and recent clinical trials. Minerva Med 2012; 103 (4): 219-34.

Filippatos G, Anker SD, Agarwall R, et al. Finerenone and Cardiovascular Outcomes in Patients with Chronic Kidney Disease and Type 2 Diabetes. Circulation. 2021; 143: 540-552.

Fox CS. Associations of kidney disease measures with mortality and end-stage renal disease in individuals with and without diabetes: a meta-analysis. Lancet 2012; 380 (9854): 1662-1673

GBD Chronic Kidney Disease Collaboration. Global, regional, and national burden of chronic kidney disease, 1990-2017: a systematic analysis for the Global Burden of Disease Study 2017. Lancet 2020; 395 (10225): 709-733.

Go AS, Chertow GM, Fan D, et al. Chronic kidney disease and the risks of death, cardiovascular events, and hospitalization. N Engl J Med 2004; 351 (13): 1296-1305.

Heerspink HJL, de Zeeuw D. Are post-trial observational studies useful? J Am Soc Nephrol. 2014; 25 (10): 2148-50.

Heerspink HJL, Stefánsson BV, Correa-Rotter R, et al. Dapagliflozin in patients with chronic kidney disease. N Engl J Med 2020; 383: 1436-1446.

Heerspink HJL, Greene T, Tighiouart H, et al. Change in albuminuria as a surrogate endpoint for progression of kidney disease: a meta-analysis of treatment effects in randomised clinical trials. Lancet Diabetes Endocrinol 2019; 7(2): 128-139.

Heerspink HJL, Avraham K, Thuresson M, et al. Kidney outcomes associated with use of SGLT2 inhibitors in real-world clinical practice (CVD-REAL 3): a multinational observational cohort study. Lancet Diabetes Endocrinol. 2020; 8: 27-35.

Herrington WG, Preiss D, Haynes R, et al. The potential for improving cardio-renal outcomes by sodium-glucose co-transporter-2 inhibition in people with chronic kidney disease: a rationale for the EMPA-KIDNEY study.CKJ, 2018; 749-761.

Inker LA, Eneanya ND, Coresh J, et al. New Creatinine- and Cystatin C–Based Equations to Estimate GFR without Race. N Engl J Med 2021 Sep 23. doi: 10.1056/NEJMoa2102953. Online ahead of print.

International Diabetes Federation. IDF Diabetes Atlas. 8th edn. Brussels, Belgium: International Diabetes Federation, 2017.

Jardiance® EU SmPC - European Medicines Agency web site: http://www.ema.europa.eu.

Kidney Disease: Improving Global Outcomes (KDIGO) Blood Pressure Work Group. KDIGO Clinical Practice Guideline for the Management of Blood Pressure in Chronic Kidney Disease. Kidney Int Suppl 2012;2:337–414.

Kidney Disease: Improving Global Outcomes (KDIGO) CKD Work Group. KDIGO 2012 clinical practice guideline for the evaluation and management of chronic kidney disease. Kidney Int Suppl 2013; 3: 1–150.

Kidney Disease: Improving Global Outcomes (KDIGO) CKD Work Group. KDIGO clinical practice guideline for acute kidney injury. Kidney Int Suppl 2012; 2: 19–36.

Kolkhof P, Jaisser F, Kim SY, et al. Steroidal and Novel Non-steroidal Mineralocorticoid Receptor Antagonists in Heart Failure and Cardiorenal Diseases: Comparison at Bench and Bedside. Handb Exp Pharmacol. 2017;243:271-305. doi: 10.1007/164_2016_76. PMID: 27830348.

Kolkhof P, Pavkovic M, Hartmann E, et al. Combined efficacy of the novel nonsteroidal and selective mineralocorticoid receptor antagonist finerenone and the SGLT2 inhibitor empagliflozin in a non-diabetic cardiorenal rat model. J Am Soc Nephrol 2021; 31 (243).

Levin A, Perkovic V, Wheeler DC, et al. Empagliflozin and Cardiovascular and Kidney Outcomes across KDIGO Risk Categories. Post Hoc Analysis of a Randomised, Double-Blind, Placebo-Controlled, Multinational Trial. Clin J Am Soc Nephrol 2020; 15 (10): 1433-1444.

Levey AS, Stevens LA, Schmid CH, et al. A new equation to estimate glomerular filtration rate. Ann Intern Med. 2009; 150 (9): 604-12.

Levey AS, Gansevoort RT, Coresh J, et al. Change in albuminuria and GFR as end points for clinical trials in early stages of CKD: A scientific workshop sponsored by the National Kidney Foundation in collaboration with the US Food and Drug Administration and European Medicines Agency. Am J Kidney Dis. 2019; 75(1): 84-104.

Matsushita K, van der Velde M, Astor BC, et al. Association of estimated glomerular filtration rate and albuminuria with all-cause and cardiovascular mortality in general population cohorts: a collaborative meta-analysis. Lancet 2010; 375 (9731): 2073-81.

McGuire DK, Zinman B, Elnzucchi S, et al. Effects of empagliflozin on first and recurrent clinical events in patients with type 2 diabetes and atherosclerotic cardiovascular disease: a secondary analysis of the EMPA-REG OUTCOME trial. Lancet Diabetes Endocrinol. 2020; 8: 959-59.

McMurray JJV, Solomon SD, Inzucchi SE, et al. Dapagliflozin in patients with heart failure and reduced ejection fraction. N Engl J Med 2019; 381(21): 1995-2008.

Molitch ME, Adler AI, Flyvbjerg A, et al. Diabetic kidney disease: a clinical update from Kidney Disease: Improving Global Outcomes. Kidney Int. 2014.

Packer M, Anker SD, Butler J, et al. Cardiovascular and renal outcomes with empagliflozin in heart failure. N Engl J Med 2020; 383(15): 1413-1424.

Perkovic V, Jardine MJ, Neal B, et al. Canagliflozin and renal outcomes in type 2 diabetes and nephropathy. N Engl J Med 2019; 380 (24): 2295-2306.

Pitt B, Kober L, Ponikowski P, et al. Safety and tolerability of the novel non-steroidal mineralocorticoid receptor antagonist BAY 94-8862 in patients with chronic heart failure and mild or moderate chronic kidney disease: a randomised, double-blind trial. European Heart Journal 2013; Aug 14;34(31):2453-2563.

Pitt B, Filippatos G, Agarwal R, et al. Cardiovascular Events with Finerenone in Kidney Disease and Type 2 Diabetes. N Engl J Med 2021; Aug 28. doi: 10.1056/NEJMoa2110956. Online ahead of print.

Pugh RN, Murray-Lyon IM, Dawson JL, et al. Transection of the oesophagus for bleeding oesophageal varices. Br J Surg 1973; 60 (8): 646–649.

Ridderstråle M, Andersen KR, Zeller C, et al. Comparison of empagliflozin and glimepiride as add-on to metformin in patients with type 2 diabetes: a 104-week randomised, active-controlled, double-blind, phase 3 trial. The Lancet Diabetes & Endocrinology 2014; 2(9): 691-700.

Rossing P, Agarwal R, Anker S, et al. Finerenone in Patients with CKD and T2D by SGLT2i Treatment: An Analysis of the FIDELIO-DKD Study (poster abstract). Diabetes 2021; 70(supplement 1): 14-LB.

Shen L, Kristensen SL, Bengtsson O, et al. Dapagliflozin in HFrEF patients treated with mineralocorticoid receptor antagonists: an analysis of DAPA-HF. JACC: Heart Failure 2021; 9 (4): 254-264.

Søfteland E, Meier JJ, Vangen B, et al. Empagliflozin as add-on therapy in patients with type 2 diabetes inadequately controlled with linagliptin and metformin: a 24-week randomized, double-blind, parallel-group trial. Diabetes Care 2017; 40(2): 201-209.

Thygesen K, Alpert JS, Jaffe AS, et al. Fourth universal definition of myocardial infarction. European Heart Journal 2019; 40(3): 237-269.

Tuttle KR, Bakris GL, Bilous RW, et al. Diabetic kidney disease: a report from an ADA Consensus Conference. Diabetes Care 2014; 37 (10): 2864-83.

Wang Y, Hu X, Liu X, Wang Z. An overview of the effect of sodium glucose cotransporter 2 inhibitor monotherapy on glycemic and other clinical laboratory parameters in type 2 diabetes patients. Ther Clin Risk Manag. 2016; 12: 1113-1131.

Wanner C, Inzucchi S, Zinman B. Consistent effects of empagliflozin on cardiovascular and kidney outcomes irrespective of diabetic kidney disease catategories: Insights from the EMPA-REG OUTCOME trial. Diabetes Obes Metab. 2020; 22: 2335-2347.

Zinman B, Wanner C, Lachin JM, et al. Empagliflozin, Cardiovascular Outcomes, and Mortality in Type 2 Diabetes. N Engl J Med 2015; 373: 2117-28.

Title Page

Protocol Title:

A parallel-group treatment, Phase 2, double-blind, three-arm study to assess efficacy and safety of finerenone plus empagliflozin compared with either finerenone or empagliflozin in participants with chronic kidney disease and type 2 diabetes.

Protocol Number: 21839

Protocol Version: 2.0

Amendment Number: 1

Compound Number: Finerenone / BAY 94-8862

Brief Title: Combination of finerenone and empagliflozin in participants with CKD and T2D

Study Phase: Phase 2

Acronym: CONFIDENCE (**CO**mbinatio**N** effect of **FI**nerenone an**D** **E**mpaglifloz**N** in participants with CKD and T2D using an UA**CR** **E**ndpoint study)

Sponsor Name: Bayer AG

Legal Registered Address:

Non-US territories: Bayer AG, 51368 Leverkusen, Germany

US territory: Bayer HealthCare Pharmaceuticals Inc., 100 Bayer Boulevard, P.O. Box 915, Whippany NJ 07981-0915, USA

Regulatory Agency Identifier Number(s):

IND: 117847

EudraCT: 2021-003037-11

EU CT (CTIS): 2023-506981-30-00

Protocol Date: 24 OCT 2023

Medical Monitor name and contact information will be provided separately.

Name: PPD Role: Global Medical Leader

This is an electronically generated document that does not bear any sponsor signatures. The signature of the sponsor’s medically responsible person is filed in the TMF and available on request.

Confidential

The information provided in this document is strictly confidential and is intended solely for the performance of the clinical investigation. Reproduction or disclosure of this document, whether in part or in full, to parties not associated with the clinical investigation or its use for any other purpose without the prior written consent of the sponsor is not permitted.

Throughout this document, symbols indicating proprietary names (®, TM) may not be displayed. Hence, the appearance of product names without these symbols does not imply that these names are not protected.

Document History Table

DOCUMENT HISTORY			
**Document name**	**Version**	**Date**	**Comments**
Amendment 1	2.0	24 OCT 2023	Consolidated version created to include the already approved country-specific protocol version (technical amendment). Change in one inclusion criterion and other minor changes (please see full list in the table below).
JPN-1	1	17 DEC 2021	Local amendment for Japan
Original Protocol	1.0	15 NOV 2021	

Note: A new naming and versioning approach was adopted after the local amendment for Japan (JPN-1, date 17 DEC 2021) was prepared. According to the current naming and versioning approach, all amendments (both global and local) are sequentially numbered and versioned.

Amendment 1 (24 OCT 2023)

Overall Rationale for the Amendment:

1. Align with EU CTR requirement:

- local versions of the global protocol are no longer allowed in the EU countries (i.e., no local stand-alone protocol amendments or local integrated protocol amendments). Due to concurrent reviews of IRB/IEC or local Health Authorities, some changes were previously implemented in a local amendment for Japan. This local amendment, called JPN-1 (dated 17 DEC 2021), is now included in Section 10.10 Appendix 10 of this Clinical Study Protocol.

2. Change in one main inclusion criterion.

Section # and Name	Description of Change	Brief Rationale
Section 1.1 Synopsis<br>Section 1.3 Schedule of Activities (SoA)<br>Section 4.1 Overall Design<br>Section 4.3.1 Finerenone<br>Section 6.5.1 Finerenone<br>Section 8.2.4 Laboratory Assessments<br>Section 8.2.5.2 Monitoring of Potassium	K+ and eGFR values obtained from local laboratory will be used for finerenone up-/down-titration as well as monitoring after down/up-titration and restart.<br>It is also specified that in exceptional cases where local laboratory results are not available, central laboratory values can be used.	Clarification edit.
Section 1.1 Synopsis<br>Section 4.3.1 Finerenone	It is specified that up-titration will be allowed from Visit 4 onward, only.	Clarification edit.

Section # and Name	Description of Change	Brief Rationale
Section 6.5.1 Finerenone		
Section 1.3 Schedule of Activities (SoA)	Footnote added for screening.	To clarify that screening assessments will be performed over several days due to 3-day urine collection.
Section 1.3 Schedule of Activities (SoA)	Notes added for prescreening activities and related footnote amended.	To clarify that prescreening UACR can be analyzed based on urine from 1 day using local laboratory.
Section 1.3 Schedule of Activities (SoA)<br>Section 8.6 Biomarkers	SoA notes for biomarkers as well as Section 8.6 are amended.	To clarify data collection for biomarkers.
Section 1.3 Schedule of Activities (SoA)	Footnote h is amended.	To allow plasma, instead of serum, creatinine assessments according to some site common practices.
Section 5.4 Screen Failures	Rescreening is now allowed once.	To allow for reassessment of eligibility for participants who were screen failed.
Section 1.3 Schedule of Activities (SoA)	Footnote j is amended.	To clarify that eligibility is based on screening laboratory results.
Section 4.3.1 Finerenone	The sentences “Initiation of finerenone treatment is recommended when K+ ≤4.8 mmol/L at the screening visit (see IB). Participants with K+ above 4.8 mmol/L at screening will not be enrolled in the study (see Section 5.2)” are deleted.	Clarification edit.
Section 5.1 Inclusion Criteria	Change from “300 ≤UACR<5000 mg/g at screening visit” to “100 ≤UACR<5000 mg/g at screening visit”.	To allow for better representation of study population and to take into account recent changes in standard of care.
Section 5.2 Exclusion Criteria	Exclusion criterion #20: the note linked to exclusion criterion #20 is changed from “1 re-assessment of K+ is allowed at the screening visit [see Section 5.4] and at the baseline visit)” to “1 re-assessment of K+ is allowed at the screening visit”.	To be coherent with the fact that participant eligibility will be based on screening K+ values.
Section 5.2 Exclusion Criteria	Exclusion criterion #5: other non-diabetic renal disease is added to the criterion.	To avoid including patients with CKD due to other reasons other than diabetes.

Section # and Name	Description of Change	Brief Rationale
Section 6.5.1 Finerenone	It is specified that dosing and titration follow the guidance in place for finerenone.	Clarification edit.
Section 6.5.1 Finerenone	Table 6-2: Guidance for Finerenone Dose Adjustment Based on K+ is edited.	Clarification edits.
Section 6.5.1 Finerenone	It is specified that whenever K+ or eGFR are checked locally during an unscheduled visit, these results should also be checked centrally.	Clarification edit.
Section 6.5.2 Empagliflozin	It is specified that there are no specific dosing requirements for empagliflozin.	Clarification edit.
Section 6.8.2 Prohibited Concomitant Therapy	K+ supplements are removed from the list of prohibited concomitant medications.	To clarify that protocol allows for treatment of hypokalemia using K+ supplements.
Section 7.1.1 Permanent Discontinuation of Study Intervention	It is specified that performing all assessments as stipulated in the visit schedule is of particular importance at Visit 6 for participants who have stopped taking study interventions	Clarification edit.
Section 7.1.2 Temporary Discontinuation	It is specified that for one single participant, interruption of study intervention means that the 2 study interventions are interrupted.	Clarification edit.
Section 8.1 Efficacy Assessment	Storage conditions for urine samples are edited.<br><br>Prescreening UACR allowed to be analyzed from urine from 1 day (instead of following 3 consecutive days)<br><br>The goal of the pre-screening UACR result is further explained.	To ensure consistency with storage conditions described in the Laboratory Manual.<br><br>To increase prescreening and to reduce patient burden.<br><br>Clarification edit.
Section 8.2.4 Laboratory Assessments	The aim of local laboratory assessments of K+ and eGFR is edited.	To be consistent with the fact that eligibility will be performed based on screening central laboratory values of K+ and eGFR.
Section 8.3.7 Disease-Related Events and/or Disease-Related Outcomes Not Qualifying for	Japan specificities are removed and reference to a new appendix is added.	These specificities are now described in a newly created appendix (Appendix 10: Country/Region-specific requirements; see below).

Section # and Name	Description of Change	Brief Rationale
Expedited Reporting as AE or SAE		
Section 10.2 Appendix 2: Clinical Laboratory Tests	Fasting conditions for glucose testing are changed from “Fasting” to “Fasting or not fasting”	Fasting state is not required as glucose is not an endpoint.
Section 10.8 Appendix 8: Guidance on Use of Common CYP Inhibitors and Inducers	Edits were made to the paragraph describing table 10-4 content and two excluded CYP3A4 inhibitors are added to the list.	New drugs on the market.
Section 10.10 Appendix 10: Country/Region-specific requirements	Local protocol amendment (JPN-1) is added in the newly created Section 10.10 Appendix 10.<br>Cross-reference to this appendix is made in Section 8.3.4 Regulatory Reporting Requirements for SAEs.	To consolidate local amendment in one global amendment to comply with the EU CTR requirement.
Section 10.10 Appendix 10: Country/Region-specific requirements	India specific requirements for safety reporting are added in Section 10.10 Appendix 10.<br>Cross-reference to this appendix is made in Section 8.3.4 Regulatory Reporting Requirements for SAEs.	To comply with regulatory requirements in India.
Throughout	Editorial updates.	-

Table of Contents

Title Page **1**

Document History Table **2**

Table of Contents **6**

Table of Tables **9**

Table of Figures **10**

1. Protocol Summary **11**
1.1 Synopsis 11
1.2 Schema 16
1.3 Schedule of Activities (SoA) 17

2. Introduction **23**
2.1 Study Rationale 23
2.2 Background 24
2.3 Benefit/Risk Assessment 26
2.3.1 Risk Assessment 26
2.3.2 Benefit Assessment 27
2.3.3 Overall Benefit: Risk Conclusion 28

3. Objectives and Endpoints and Estimands **29**

4. Study Design **31**
4.1 Overall Design 31
4.2 Scientific Rationale for Study Design 33
4.2.1 Participant’s Input into Design 34
4.3 Justification for Dose 34
4.3.1 Finerenone 34
4.3.2 Empagliflozin 34
4.4 End of Study Definition 35

5. Study Population **35**
5.1 Inclusion Criteria 35
5.2 Exclusion Criteria 37
5.3 Lifestyle Considerations 38
5.3.1 Meals and Dietary Restrictions 38
5.3.2 Other Lifestyle Considerations 38
5.4 Screen Failures 38
5.5 Criteria for Temporarily Delaying Enrollment/Randomization/Study Intervention Administration 39

6. Study Intervention(s) and Concomitant Therapy **39**
6.1 Study Interventions Administered 39
6.1.1 Study Interventions 39
6.1.2 Medical Devices 42
6.2 Preparation/Handling/Storage/Accountability 42
6.3 Measures to Minimize Bias: Randomization and Blinding 42
6.4 Study Intervention Compliance 43
6.5 Dose Modification 44
6.5.1 Finerenone 44

6.5.2 Empagliflozin 47
6.6 Continued Access to Study Intervention After the End of the Study 47
6.7 Treatment of Overdose 47
6.7.1 Finerenone 47
6.7.2 Empagliflozin 47
6.7.2.1 Symptoms 47
6.7.2.2 Therapy 47
6.8 Concomitant Therapy 48
6.8.1 Permitted Concomitant Therapy 48
6.8.2 Prohibited Concomitant Therapy 49
6.8.3 Hyperkalemia Events 49
6.8.4 Unexpected Acute Declines in eGFR 49
6.8.5 Volume Depletion/Hypotension 50
6.8.6 Ketoacidosis 50

7. Discontinuation of Study Intervention and Participant Discontinuation/Withdrawal 51
7.1 Discontinuation of Study Intervention 51
7.1.1 Permanent Discontinuation of Study Intervention 51
7.1.2 Temporary Discontinuation 51
7.2 Participant Discontinuation/Withdrawal from the Study 52
7.3 Lost to Follow-Up 52

8. Study Assessments and Procedures 53
8.1 Efficacy Assessment 53
8.2 Safety Assessments 54
8.2.1 Vital Signs 54
8.2.1.1 Ambulatory Blood Pressure Monitoring – Only in Part A 54
8.2.2 Physical Examinations 55
8.2.3 Electrocardiograms 55
8.2.4 Laboratory Assessments 55
8.2.5 Other Safety Assessments 56
8.2.5.1 eGFR 56
8.2.5.2 Monitoring of Potassium 57
8.2.5.3 Acute Kidney Injury 57
8.2.5.4 Severe Hypoglycemia 57
8.2.5.5 Symptomatic Hypotension (Including Volume Depletion) 57
8.2.5.6 Genital Mycotic Events 57
8.2.5.7 Ketoacidosis Events 58
8.2.5.8 Necrotizing Fasciitis of the Perineum (Fournier’s Gangrene) Events 58
8.2.5.9 Urosepsis and Pyelonephritis Events 58
8.2.6 Pregnancy Testing 58
8.2.7 Suicidal Ideation and Behavior Risk Monitoring 58
8.3 Adverse Events (AEs), Serious Adverse Events (SAEs) and Other Safety Reporting 58
8.3.1 Time Period and Frequency for Collecting AE and SAE Information 58
8.3.2 Method of Detecting AEs and SAEs 59
8.3.3 Follow-Up of AEs and SAEs 59
8.3.4 Regulatory Reporting Requirements for SAEs 59
8.3.5 Pregnancy 59

8.3.6 Cardiovascular and Death Events 60
8.3.7 Disease-Related Events and/or Disease-Related Outcomes Not Qualifying for Expedited Reporting as AE or SAE 60
8.3.8 Adverse Events of Special Interest 61
8.4 Pharmacokinetics 61
8.5 Genetics and/or Pharmacogenomics 62
8.6 Biomarkers 62
8.7 Immunogenicity Assessments 63
8.8 Health Economics 63

9. Statistical Considerations 63
9.1 Statistical Hypotheses 63
9.2 Sample Size Determination 64
9.3 Analysis Sets 65
9.4 Statistical Analyses 66
9.4.1 General Considerations 66
9.4.2 Disposition, Baseline, History, Demography and Medication 67
9.4.3 Disposition 67
9.4.3.1 Population Characteristics 67
9.4.3.2 Demography and Other Baseline Characteristics 68
9.4.3.3 Medical History 68
9.4.3.4 Concomitant Medication 68
9.4.3.5 Treatment Duration, Extent of Exposure, Up-Titration Status, and Compliance 68
9.4.4 Primary Efficacy Endpoints 69
9.4.5 Secondary Efficacy Endpoints 70
9.4.5.1 Relative Change in UACR Category 70
9.4.5.2 Subgroup Analyses 70
9.4.6 Safety Analysis 71
9.4.6.1 Adverse Event 72
9.4.6.2 Laboratory Data 72
9.4.6.3 Vital Signs, Including Weight and BMI 73
9.4.6.4 Further Safety Variables 73
9.4.7 Missing Data/Dropouts 75
9.5 Interim Analysis 76

10. Supporting Documentation and Operational Considerations 77
10.1 Appendix 1: Regulatory, Ethical, and Study Oversight Considerations 77
10.1.1 Regulatory and Ethical Considerations 77
10.1.2 Financial Disclosure 77
10.1.3 Informed Consent Process 77
10.1.4 Data Protection 78
10.1.5 Committee Structure 78
10.1.5.1 Data Monitoring Committee 78
10.1.5.2 Steering Committee 78
10.1.6 Dissemination of Clinical Study Data 79
10.1.7 Data Quality Assurance 79
10.1.8 Source Documents 80
10.1.9 Study and Site Start and Closure 80
10.1.10 Publication Policy 81

10.2 Appendix 2: Clinical Laboratory Tests 81
10.2.1 Biomarkers 82
10.2.2 Pharmacokinetics 83
10.3 Appendix 3: Disease-Related Event Definitions 83
10.3.1 Myocardial Infarction 83
10.3.1.1 Criteria for Myocardial Infarction 83
10.3.2 Stroke 85
10.3.2.1 Ischemic Stroke 86
10.3.2.2 Hemorrhagic Stroke 86
10.3.2.3 Undetermined Stroke 86
10.3.3 New Onset of HF 86
10.3.4 Hospitalization for HF 86
10.3.5 Chronic Sustained Decrease in EGFR 87
10.4 Appendix 4: AEs and SAEs: Definitions and Procedures for Recording, Evaluating, Follow-Up, and Reporting 88
10.4.1 Definition of AE 88
10.4.2 Definition of SAE 89
10.4.3 Recording and Follow-Up of AE and/or SAE 90
10.4.4 Reporting of SAEs 92
10.5 Appendix 5: Death Events Definition 92
10.5.1 Cardiovascular Death 92
10.5.1.1 Death due to Acute Myocardial Infarction 92
10.5.1.2 Sudden Cardiac Death 93
10.5.1.3 Undetermined Death 93
10.5.1.4 Death due to Heart Failure 93
10.5.1.5 Death due to Stroke 94
10.5.1.6 Death due Cardiovascular Procedures 94
10.5.1.7 Death due to Other Cardiovascular Causes 94
10.5.2 Renal Death 95
10.5.3 Non-Cardiovascular and Non-Renal Deaths 95
10.6 Appendix 6: Contraceptive Guidance and Collection of Pregnancy Information 95
10.7 Appendix 7: Calculating the Child Pugh Score 97
10.8 Appendix 8: Guidance on Use of Common CYP Inhibitors and Inducers 98
10.9 Appendix 9: Abbreviations 99
10.10 Appendix 10: Country/Region-specific Requirements 101
10.10.1 Japan 101
10.10.1.1 JPN-1: Country-specific requirements valid for Japan only 101
10.10.2 India 103

11. References 105

Table of Tables

Table 1-1: Schedule of Activities 18
Table 6-1: Dose Adjustments of Finerenone 45
Table 6-2: Guidance for Finerenone Dose Adjustment Based on K+ 46
Table 9-1: Analysis Sets 66
Table 10-1: Protocol-Required Laboratory Tests 82
Table 10-2: Grading of Severity of Liver Disease (adapted from *Pugh et al., 1973*) 97

Table 10-3: Classification Using the Added Score from Table 10-2 97
Table 10-4: Cytochrome P450: List of Concomitant Medication.. 98

Table of Figures

Figure 1-1: Study Design 16

1. Protocol Summary

1.1 Synopsis

Protocol Title: A parallel-group treatment, Phase 2, double-blind, three-arm study to assess efficacy and safety of finerenone plus empagliflozin compared with either finerenone or empagliflozin alone in participants with chronic kidney disease and type 2 diabetes

Brief Title: Combination of finerenone and empagliflozin in participants with CKD and T2D

Rationale:

In the FIDELIO-DKD and FIGARO-DKD phase 3 clinical trials, finerenone, a nonsteroidal, selective antagonist of the mineralocorticoid receptor (MRA), has proven its efficacy and safety to reduce risk of kidney disease progression and cardiovascular (CV) events in patients with chronic kidney disease (CKD) and type 2 diabetes (T2D).

Empagliflozin is a sodium-glucose cotransporter-2 inhibitor (SGLT2i) indicated to reduce the risk of CV death in adult patients with T2D and established CV disease. Empagliflozin has shown evidence of reduction of kidney disease progression and CV events in patients with or without T2D and CKD in CV trials and is now investigated in a dedicated CKD trial. Other SGLT2i (dapagliflozin and canagliflozin) showed renal and CV efficacy in patients with CKD with and without T2D, which led to a label change and updated guideline recommendations.

In a recent preclinical study, it has been shown that finerenone and empagliflozin have an additive effect on urinary protein-to-creatinine ratio, a predictor of CKD progression, and CV adverse outcomes in CKD patients.

In pooled analysis of FIDELIO-DKD/FIGARO-DKD (FIDELITY; data on file), it has been shown in the subgroup of patients treated with SGLT2i at baseline that there is a synergy in reduction of urinary albumin-to-creatinine ratio (UACR) and prevention of events.

This study aims to demonstrate that the initial combined use of finerenone and empagliflozin is superior to either empagliflozin alone, or finerenone alone, in reducing UACR from baseline to 180 days. UACR is a measurement of albuminuria, a predictor of long-term renal and CV adverse outcomes in T2D patients.

Objectives and Endpoints and Estimands:

Objectives	Endpoints and Estimands
**Primary**	
• To demonstrate that combination therapy using finerenone and empagliflozin is superior in reducing UACR than either empagliflozin or finerenone alone	Primary Endpoints:<br>• Relative change from baseline in UACR at 180 days in combination therapy group versus empagliflozin alone<br>or<br>• Relative change from baseline in UACR at 180 days in combination therapy group versus finerenone alone
	Intercurrent events: Participants who discontinues study medication before 180 days, dialysis or kidney transplantation, and death
	Summary Measures:<br>• Mean ratio of change from baseline to Day 180 in UACR for the combination therapy group, to empagliflozin alone<br>• Mean ratio of change from baseline to Day 180 in UACR for the combination therapy group, to finerenone alone
**Secondary**	
• To further investigate the efficacy of combination therapy using finerenone and empagliflozin versus either finerenone or empagliflozin alone	• Relative change in UACR between end of treatment visit and 30 days after end of treatment visit<br>• Relative change in UACR between 30 days after end of treatment visit and baseline<br>• Relative change in UACR category (>30%, >40%, >50%) at 180 days

Objectives	Endpoints and Estimands
• To evaluate the safety of combination therapy using finerenone and empagliflozin versus either finerenone or empagliflozin alone	• Ratio of change from baseline in eGFR at 30 days<br>• eGFR decline greater than 30% at 30 days from baseline<br>• Ratio of change in eGFR at 180 days and 210 days from Day 30<br>• Proportion of participants with of AKI events<br>• Total number of AKI events<br>• Proportion of participants with hyperkalemia events (moderate hyperkalemia [5.5 <K+ ≤6.0 mmol/L], severe hyperkalemia [K+ >6.0 mmol/L])<br>• Total number of hyperkalemia events (moderate hyperkalemia [5.5 <K+ ≤6.0 mmol/L], severe hyperkalemia [K+ >6.0 mmol/L])<br>• Change from baseline in K+<br>• Proportion of participants with severe hypoglycemia events<br>• Total number of events of severe hypoglycemia events<br>• Proportion of participants with symptomatic hypotension events<br>• Total number of symptomatic hypotension events<br>• Proportion of participants with genital mycotic events<br>• Total number of genital mycotic events<br>• Proportion of participants with ketoacidosis events<br>• Total number of ketoacidosis events<br>• Proportion of participants with necrotizing fasciitis of the perineum events<br>• Total number of necrotizing fasciitis of the perineum events<br>• Proportion of participants with urosepsis and pyelonephritis events<br>• Total number of urosepsis and pyelonephritis events

Objectives	Endpoints and Estimands
**Other exploratory**	
• To further investigate the study intervention (finerenone, empagliflozin) and similar drugs (e.g., mode-of-action-related effects, safety) and to further investigate pathomechanisms deemed relevant to CV disease, CKD, diabetes, and associated health problems	• Various biomarkers (e.g., diagnostic, safety, pharmacodynamic, monitoring, or potentially predictive biomarkers)
**Other pre-specified**	
• To characterize the PK of finerenone and empagliflozin when given in combination	• PK of finerenone and empagliflozin in plasma ($C_{max,md}$, $AUC_{\tau,md}$) (optional analysis)
Abbreviations: AKI = acute kidney injury; $AUC_{\tau,md}$ = area under the concentration vs. time curve for the expected dosing interval obtained after multiple dose administration; $C_{max,md}$ = maximum drug concentration after multiple dose administration; CV = cardiovascular; CKD = chronic kidney disease; eGFR = estimated glomerular filtration rate; K+ = serum/plasma potassium; PK = pharmacokinetics; UACR = urinary albumin-to-creatinine ratio.	

This study will include adult participants with a clinical diagnosis of T2D and CKD. There are 2 primary endpoints to address the objective of the study; relative change from baseline in UACR at 180 days in combination therapy group versus empagliflozin alone and relative change from baseline in UACR at 180 days in combination therapy group versus finerenone alone. These 2 primary endpoints are not considered as co-primary endpoints.

Overall Design:

- Phase 2, randomized, controlled, double-blind (participants and investigators), double-dummy, multicenter study in participants with CKD and T2D.
- The study will consist of 2 consecutive parts:
    - Part A: participants will be recruited if their estimated glomerular filtration rate (eGFR) is between 40 and 90 ml/min/1.73 m², and they will be equipped with an ambulatory blood pressure monitoring (ABPM) device at Visit 2 for a duration of 24 hours. An interactive web response system (IWRS) will allow capping the number of participants as follows:
        - 80% with an eGFR between ≤75 ml/min/1.73 m²
        - 20% with an eGFR between >75 ml/min/1.73 m².
    - Part B: participants will be recruited if their eGFR is between 30 and 90 ml/min/1.73 m², and they will not have an ABPM. The IWRS will allow capping the number of participants as follows:
        - 80% with an eGFR between ≤75 ml/min/1.73 m²
        - 20% with an eGFR between >75 ml/min/1.73 m².
    - The decision to move from Part A to Part B will be taken by the sponsor and the study's Steering Committee upon feedback from the Data Monitoring Committee (DMC). The safety analysis from the first 50 participants in Part A, as well as their unblinded review by the independent DMC will be used to confirm the enrollment/recruitment start for Part B. This decision shall be effective immediately or after IRB/IEC and/or local Health Authority approval,

where applicable. Other inclusion/exclusion criteria or study's schedule or procedure should not be affected.

- Participants will be randomized in a 1:1:1 ratio stratified by eGFR at screening (<60, ≥60 mL/min/1.73m²) and UACR at screening (≤ 850mg/g, > 850 mg/g) in one of the 3 parallel groups:
    - Finerenone (10 or 20 mg [target dose] once daily [OD]) and empagliflozin (10 mg OD)
    - Finerenone (10 or 20 mg OD) and matching placebo to empagliflozin OD
    - Empagliflozin (10 mg OD) and matching placebo to finerenone OD.

  Starting from Visit 4, finerenone or placebo doses will be adjusted based on serum/plasma potassium (K+) and eGFR values obtained from local laboratories (in exceptional cases where local laboratory results are not available, central laboratory values can be used), at each study visit.
- Participants should be treated for CKD and for T2D according to local treatment guidelines. However, participants must not be exposed to an SGLT2i and/or an MRA within at least 8 weeks prior to screening.

Brief Summary:

The purpose of this study is to assess efficacy and safety of the initial combination therapy using finerenone and empagliflozin compared with either finerenone or empagliflozin alone for reducing UACR in participants with CKD and T2D. Study details include:

Visit Frequency: Screening (up to 2 weeks before randomization), Day 1 (randomization), Days 14 (±2), 30 (±4), 90 (±5) 180 (±5) (end of study intervention), Day 210 (±5; follow-up visit/end of study).

Condition/Disease: Participants with CKD and T2D

Study Hypothesis: Assuming a cumulative effect of both study interventions we hypothesize that in the combination therapy arm, UACR will be 20% lower at 180 days versus the empagliflozin arm, or 20% lower at 180 days versus the finerenone arm.

Health Measurement/Observation: The primary endpoints will be the relative change from baseline in UACR at 180 days in the combination therapy arm versus each of the monotherapy arms.

Number of Participants: With a 50% screening failure rate and a 15% dropout rate, approximately **1,614** participants will be screened to achieve approximately **807** randomly assigned participants to study interventions, for approximately **269** participants per intervention group.

Intervention Groups and Duration:

The total study duration for each participant will be approximately 7.5 months (up to 8.5 months if the optional pre-screening is performed).

At Day 1, participants will be randomized in a 1:1:1 ratio in one of the 3 treatment groups:

- Finerenone (10 or 20 mg OD) and empagliflozin (10 mg OD)
- Finerenone (10 or 20 mg OD) and matching placebo to empagliflozin OD
- Empagliflozin (10 mg OD) and matching placebo to finerenone OD.

Finerenone, or sham placebo, up or down-titrations will be allowed during the study. Up-titration to the target dose of 20 mg will be allowed from Visit 4 onwards, based on eGFR and K+ results. Down-titration will be allowed any time during the study (e.g., between scheduled visits) for safety reasons only. Treatment duration will be approximately 180 days per participant.

Data Monitoring Committee: Yes

1.2 Schema

Figure 1-1: Study Design

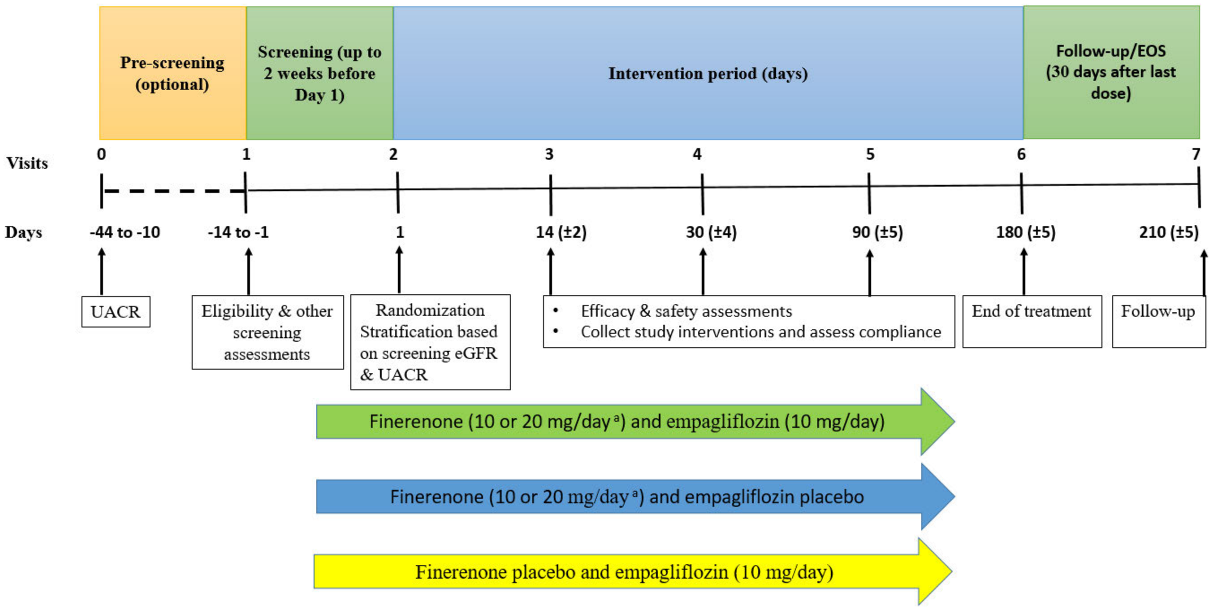

Abbreviations: eGFR = estimated glomerular filtration rate; EOS = end of study; UACR = urinary albumin-to-creatinine ratio.

[a] Up-/down-titration based on eGFR, serum/plasma potassium or potassium, safety, and tolerability.

1.3 Schedule of Activities (SoA)

Table 1-1: Schedule of Activities

Procedure	Pre-Screening[a]	Screening (up to 2 weeks before Day 1)[q]	Intervention Period [Days]					ED	Follow-Up/EOS (30 days after last dose)	Notes
			1[b] (dose)	14±2	30±4	90±5	180±5 (EOT)		210±5	
**Visit**	**0**	**1**	**2**	**3[r]**	**4**	**5**	**6**		**7**	
**Study Day**	**-44 to -10**	**-14 to -6**	**1**	**12-16**	**26-34**	**85-95**	**175-185**		**205-215**	
Pre-screening consent	X									
Informed consent		X								
Inclusion and exclusion criteria		X	X							Re-check clinical status before randomization
Demography		X								
Complete physical examination including height[c] and weight		X	X	X	X	X	X	X	X	
Vital signs		X	X	X	X	X	X	X	X	
Medical history		X								
Serum pregnancy test (WOCBP only)		X			X	X	X	X		To be performed by central lab
FSH test (to confirm postmenopausal status, when needed)[d]		X								
Central laboratory tests										
UACR (urinary creatinine, albumin)[e,f]		X[i]	X	X	X	X	X	X	X	
Serum K+[e]		X[i]	X	X	X	X	X	X	X	
eGFR (serum creatinine)[e]		X[i]	X	X	X	X	X	X	X	

Table 1-1: Schedule of Activities

Procedure	Pre-Screening[a]	Screening (up to 2 weeks before Day 1)[q]	Intervention Period [Days]					ED	Follow-Up/EOS (30 days after last dose)	Notes
			1[b] (dose)	14±2	30±4	90±5	180±5 (EOT)		210±5	
**Visit**	**0**	**1**	**2**	**3[r]**	**4**	**5**	**6**		**7**	
**Study Day**	**-44 to -10**	**-14 to -6**	**1**	**12-16**	**26-34**	**85-95**	**175-185**		**205-215**	
Safety laboratory tests		X	X	X	X	X	X	X	X	Clinical chemistry, hematology, urinalysis. Lipids (HDL, LDL-C, total cholesterol and triglycerides) will be measured at screening only
Local laboratory tests[g]										
UACR	X									One sample, preferably first morning void
Serum/plasma K+[h]			X[j]	X[k]	X[k]	X[k]	X[k]	X[k]	X[k]	In exceptional cases where local laboratory results are not available, central laboratory values can be used.
eGFR (serum creatinine)[h]			X[j]	X[k]	X[k]	X[k]	X[k]	X[k]	X[k]	In exceptional cases where local laboratory results are not available, central laboratory values can be used.
PK[l,m]				X	X	X	X	X		

Table 1-1: Schedule of Activities

Procedure	Pre-Screening[a]	Screening (up to 2 weeks before Day 1)[q]	Intervention Period [Days]					ED	Follow-Up/EOS (30 days after last dose)	Notes
			1[b] (dose)	14±2	30±4	90±5	180±5 (EOT)		210±5	
**Visit**	**0**	**1**	**2**	**3[r]**	**4**	**5**	**6**		**7**	
**Study Day**	**-44 to -10**	**-14 to -6**	**1**	**12-16**	**26-34**	**85-95**	**175-185**		**205-215**	
Biomarkers (blood)[l]		X	X		X		X	X		Day 1 sample to be collected prior to start of treatment. Visit 6 sample to be collected after treatment administration at site. For Visit 4 and ED, timing of at-home treatment should be recorded on contact card.
Biomarker samples (first morning void urine)			X		X		X	X		
12-lead ECG		X	X				X			
Randomization			X							
Dispense urine containers for UACR assessments		X	X	X	X	X	X			
Study intervention dispensation/accountability			X[n]		X	X	X[o]			
24-hour ABPM			X[n]							
Dose adjustment (with unscheduled visits)[p]					X	X				Up-/down-titration, restart after interruption
Adverse events	X	X	←==→							

Table 1-1: Schedule of Activities

Procedure	Pre-Screening[a]	Screening (up to 2 weeks before Day 1)[q]	Intervention Period [Days]					ED	Follow-Up/EOS (30 days after last dose)	Notes
			1[b] (dose)	14±2	30±4	90±5	180±5 (EOT)		210±5	
**Visit**	**0**	**1**	**2**	**3[r]**	**4**	**5**	**6**		**7**	
**Study Day**	**-44 to -10**	**-14 to -6**	**1**	**12-16**	**26-34**	**85-95**	**175-185**		**205-215**	
Prior/Concomitant medication review		X	←==→							

Abbreviations: ABPM = ambulatory blood pressure monitoring; ECG = electrocardiogram; ED = early discontinuation; EOS = end of study; EOT = end of treatment; eGFR = estimated glomerular filtration rate; HDL = high density lipoprotein; LDL-C = low density lipoprotein cholesterol; K+ = serum/plasma potassium; PK = pharmacokinetics; UACR = urinary albumin-to-creatinine ratio; WOCBP = woman of childbearing potential.

a) Pre-screening visit is optional (up to 4 weeks before screening).
b) Day 1 = randomization/baseline visit.
c) Height will be measured at screening only.
d) May be used to confirm a postmenopausal state in women not using hormonal contraception or HRT. In the absence of 12 months of amenorrhea, confirmation with more than one FSH measurement is required.
e) Central laboratory values to be used for endpoint statistical analyses.
f) At Visit 1, first morning void urine samples to be collected on 3 consecutive days at the participant's home. At the other visits, the 2 consecutive samples can be collected ±7 days from the visit date.
g) Local laboratory values for safety purposes.
h) Blood samples (for K+ and creatinine [eGFR]) for measurement in the local laboratory may be obtained up to 72 hours before a scheduled visit. If serum creatinine is not available, plasma may be used.
i) One re-assessment (central lab) of eGFR, K+ and/or UACR is allowed at the screening visit.
j) K+ and eGFR values obtained from screening will be used for participant eligibility at Day 1.
k) K+ and eGFR values obtained from local laboratory will be used for finerenone up-/down-titration as well as monitoring after down/up-titration and restart.
l) Refer to sampling handling sheets or laboratory manual for sample handling.
m) At Visits 3 and 6, trough (i.e. pre-dose) samples for the determination of finerenone and empagliflozin plasma concentrations will be drawn before intake of study intervention. At this visit, study intervention will be administered at the study site and the exact time of study intervention intake on the day before the visit and on the day of the visit and the exact sampling time will be recorded in the electronic case report form. At Visits 4 and 5 (and ED, if applicable), post-dose blood samples for the determination of finerenone and empagliflozin plasma concentrations will be drawn during the visit, 1.5-10 hours after study intervention intake at home. Note: At all mentioned visits, samples should be taken even if the study interventions were not taken as indicated. In such cases, particular attention will be paid to properly document the actual time of study intervention intake before sampling. These samples will not be analyzed directly but may be analyzed in case of specific questions from the DMC, Steering Committee, or sponsor.
n) First study intervention at study site. The participant from Part A will remain 4 to 6 hours at the study site for office blood pressure monitoring and will then be equipped for 24 hours with an ABPM device.
o) Accountability only
p) Subsequent to an up-titration or restart of study drug after interruption of finerenone intake for more than 7 days, the investigator should perform an unscheduled visit, 4 weeks (±7 days) after titration or restart, in order to monitor K+ levels and eGFR.
q) Screening assessments will be done over several days due to urine collection during 3 consecutive days.
r) At Visit 3, no up-titration is allowed (for other actions, see Section 6.5.1).

2. Introduction

Finerenone (Kerendia®) is a novel, nonsteroidal, potent and selective mineralocorticoid receptor antagonist (MRA), recently approved by the Food and Drug Administration (FDA) to reduce the risk of sustained estimated glomerular filtration rate (eGFR) decline, end-stage kidney disease (ESKD), cardiovascular (CV) death, non-fatal myocardial infarction (MI), and hospitalization for heart failure (HF) in adult patients with chronic kidney disease (CKD) associated with type 2 diabetes (T2D).

Empagliflozin (Jardiance®) is a sodium/glucose cotransporter-2 inhibitor (SGLT2i) initially marketed to treat T2D and which has shown to prevent major adverse CV events in T2D patients. Other SGLT2i have also shown benefit in patients with CKD, while empagliflozin is still under investigation in this population.

2.1 Study Rationale

Finerenone is an MRA acting by targeting inflammatory and fibrotic pathways mediated by mineralocorticoid receptor (MR) over-activation in the heart and kidney (Kolkhof et al. 2017). In FIDELIO-DKD, finerenone protected from kidney function decline and reduced the risk of CV events in patients with CKD and T2D (Bakris et al. 2020; Filippatos et al. 2021). Finerenone also significantly reduced urinary albumin-to-creatinine ratio (UACR) compared to placebo at 4 months.

SGLT2i are indicated for the treatment of T2D and some of them are also indicated for the treatment of HF and CKD. Clinical studies in CKD patients showed that SGLT2i reduce the risk of kidney failure and CV events, regardless of the presence or absence of diabetes (Perkovic et al. 2019; Heerspink et al. 2020). Similarly, SGLT2i lower the risk of CV death or hospitalization for HF, regardless of the presence or absence of diabetes, in patients with HF (Packer et al. 2020).

Combining these 2 drugs could be interesting for the reasons explained hereafter.

Data from sub-analyses of trials in HF (McMurray et al. 2019) suggest that the usage of SGLT2i in patients already taking a steroidal MRA could reduce the incidence of hyperkalemia in this patient population. This finding has been replicated in an analysis of the DAPA-HF study (Shen et al. 2021). In a post-hoc analysis of FIDELIO-DKD, patients treated with finerenone and SGLT2i had fewer hyperkalemia events than those receiving finerenone alone (Rossing et al. 2021).

Efficacy of finerenone on CV outcomes was observed independent of the presence or absence of SGLT2i use at baseline. In a recent preclinical study on non-diabetic mice model, it has been shown that empagliflozin and finerenone have an additive effect on urinary protein-to-creatinine ratio (Kolkhof et al. 2021).

Regarding UACR, a greater improvement was observed in finerenone patients receiving SGLT2i at baseline: reduction in UACR with finerenone was seen without SGLT-2i use (ratio of LS-means 0.68, 0.65-0.71; $p<0.0001$) and on top of SGLT-2is at baseline (ratio of LS-means 0.75, 0.62-0.90; $p=0.0024$) (Rossing et al. 2021).
It is then hypothesized that the dual treatment effects of finerenone combined with an SGLT2i will have additive renoprotective benefits in CKD patients with T2D.
Elevated UACR is a key risk marker for CKD and cardiovascular disorders (Cohen et al. 2021), more particularly in patients with high albuminuria (Heerspink et al. 2019).

This study will prospectively expand on these observations by being the first parallel-group randomized controlled trial to evaluate the safety, tolerability, and additive efficacy of the combined use of finerenone and empagliflozin using the surrogate endpoint of UACR. This study will also investigate, for the first time, simultaneous initiation of finerenone and empagliflozin. The goal of this study is to show that the combined use of finerenone and empagliflozin is superior to either empagliflozin alone, or finerenone alone in reducing UACR at 180 days.

2.2 Background

CKD is defined as abnormalities of kidney structure or function, with either of the following present for at least 3 months (KDIGO 2013):

Markers of kidney damage	Albumin:creatinine ratio (ACR) ≥30 mg/g<br>Urine sediment abnormalities (e.g., hematuria, red cell casts, etc.)<br>Electrolyte and other abnormalities due to tubular disorders<br>Abnormalities detected by histology<br>Structural abnormalities detected by imaging<br>History of kidney transplantation
Decreased glomerular filtration rate	Glomerular filtration rate <60 mL/min/1.73 m^2

In 2017, 697.5 million cases of all-stage CKD were recorded accounting for a global prevalence of 9.1%. Globally, 4.6% of total mortality were caused by deaths due to CKD and CV disease attributable to impaired kidney function (GBD Chronic Kidney Disease Collaboration 2020).

CKD in patients with T2D is the most frequent cause of ESKD in western countries (Fernandez et al. 2012). In addition, the risk of CV disease and death increases in diabetic patients with CKD with decreasing glomerular filtration rate (GFR) and increasing albuminuria levels (Matsushita et al. 2010; KDIGO 2013).

As the T2D population rapidly grows throughout the world within the next years (International Diabetes Federation 2017), and with it the CKD population, there is an increasing need for new therapeutic agents that effectively target underlying disease mechanisms and slow or halt the progression of kidney disease, while also addressing the high CV morbidity and mortality in this population.

Finerenone is a nonsteroidal, selective antagonist of the MR that potently attenuates inflammation and fibrosis mediated by MR over-activation. The MR is expressed in the kidneys, heart, and blood vessels where finerenone also counteracts sodium retention and hypertrophic processes. Finerenone has a high potency and selectivity for the MR due to its nonsteroidal structure and bulky binding mode. Finerenone has no relevant affinity for androgen, progesterone, estrogen, and glucocorticoid receptors and therefore does not cause sex hormone-related adverse events (AEs; e.g., gynecomastia). Its binding to the MR leads to a specific receptor ligand complex that blocks recruitment of transcriptional coactivators implicated in the expression of pro-inflammatory and pro-fibrotic mediators (Agarwal et al. 2020; Agarwal et al. 2021).

During the phase 2 and 3 trials, it has been shown that:

- Finerenone reduced albuminuria in diabetic and non-diabetic patients with HF with reduced ejection fraction (HFrEF) and CKD and that it is at least as effective as

spironolactone in decreasing plasma N-terminal prohormone B-type natriuretic peptide (NT-proBNP) and UACR (Pitt et al. 2013).

- Among patients with T2D and UACR, ≥30 mg/g on stable therapy with an angiotensin-converting enzyme inhibitor (ACEi) or angiotensin receptor blocker (ARB), finerenone (10, 15, and 20 mg once daily [OD]) demonstrated a dose-dependent placebo-subtracted reduction in UACR of 25% to 38% over 90 days. Minimal effects on potassium (K+) and renal function and a limited effect on reducing blood pressure (BP) were observed (Bakris et al. 2015).
- 5,734 patients with CKD and T2D were further enrolled in the FIDELIO-DKD study. In this study, finerenone significantly lowered the risk of CKD progression and CV events compared to placebo. In terms of safety, the incidence of AEs was similar in both finerenone and placebo groups but higher mean K+ levels were consistently observed in the finerenone group. Finerenone also had a modest effect on BP with changes in mean systolic BP from baseline to month 1 and to month 12 of -3.0 and -2.1 mm Hg, respectively (Bakris et al. 2020).
- 7,437 patients with CKD and T2D were randomized in the FIGARO-DKD study. Among patients with T2D and stage 2 to 4 CKD with moderately elevated albuminuria or stage 1 or 2 CKD with severely elevated albuminuria, finerenone therapy improved CV outcomes as compared with placebo. The overall frequency of AEs did not differ substantially between groups. The incidence of hyperkalemia-related discontinuation of the trial regimen was higher with finerenone (1.2%) than with placebo (0.4%) (Pitt et al. 2021).

A detailed description of the chemistry, pharmacology, efficacy, and safety of finerenone is provided in the Investigator's Brochure (IB). Finerenone is currently being evaluated by several Healthcare Authorities for a marketing authorization and has been approved in the United States of America (US).

SGLT2i is highly expressed in the kidney and is responsible, as the predominant transporter, for the reabsorption of glucose from the glomerular filtrate back into the circulation. Empagliflozin acts by reducing renal glucose reabsorption. Empagliflozin is prescribed either as a monotherapy when metformin is considered inappropriate due to intolerance or as an adjunct therapy to other diabetes treatments. Clinical data from the EMPA-REG OUTCOME trial suggested that the treatment of T2D and atherosclerotic CV patients with empagliflozin reduced the risk of CV events and all-cause hospitalization (McGuire et al. 2020) and led to Jardiance® label extension. As well, in patients with T2D at higher CV risk, empagliflozin was associated with slower progression of kidney disease and lower rates of renal complications (Wanner et al. 2020). In the CVD-REAL 3, real-world clinical study, empagliflozin was used in 34% of patients and associated with a lower rate of eGFR decline and reduced risk of composite endpoint of 50% eGFR decline (Heerspink et al. 2020). These findings strongly support the potential therapeutic use of empagliflozin for the reduction of CKD in T2D patients.

A summary of the pharmacology, efficacy, and safety of empagliflozin can be found in the European Summary of Product Characteristics (EU SmPC).

2.3 Benefit/Risk Assessment

Relevant emerging safety data, e.g., serious AEs (SAEs), suspected unexpected serious adverse reactions (SUSARs), and serious safety-related protocol deviations, will be communicated as soon as possible between the sponsor, all study sites, and investigators and trial participants.

2.3.1 Risk Assessment

Finerenone has a favorable benefit-risk profile. Adverse reactions reported with finerenone include hyperkalemia, hyponatremia, hypotension (mean systolic BP [SBP] decrease of 3 mmHg and mean diastolic BP [DBP] decrease by 1 to 2 mmHg after 4 weeks of treatment) and GFR decreased (mean decrease in eGFR of 2 mL/min/1.73 m^2 within the first 4 weeks). K+, BP, and eGFR will be monitored at each study visit.

Regarding hyperkalemia, Rossing et al. (2021) found that patients treated with finerenone and SGLT2i had fewer hyperkalemia events than those receiving finerenone alone, in a post-hoc analysis of FIDELIO-DKD. However, to minimize safety risks to the participants, finerenone treatment will be initiated only if K+ is less than or equal to 4.8 mmol/L at screening and the starting dose of finerenone will be chosen according to baseline eGFR. Subsequent titration will be performed on the basis of measured K+ and eGFR values. Stopping rules for temporary and permanent discontinuation or dose reduction of finerenone based on K+ values will minimize the risk of hyperkalemia. At any time during the study, the investigator has the option to also down-titrate finerenone, depending on K+ and the progression of the underlying disease (see Section 6.5). More detailed information about finerenone may be found in the IB.

The most commonly reported AEs for empagliflozin are hypoglycemia with concomitant use with insulin and insulin secretagogues, volume depletion, genital mycotic infections, thirst, constipation, pruritus, increased urination, and serum lipids increased. Special warnings and precautions for use include ketoacidosis, volume depletion, urosepsis and pyelonephritis, and necrotizing fasciitis of the perineum (Fournier's Gangrene). Guidance on ketoacidosis management will be provided to the investigators (see Section 6.8.6). Empagliflozin is associated with an initial fall in BP and eGFR (mean eGFR decrease of 3 ml/min/1.73 m^2 [EU SmPC]). In the US, empagliflozin can be administered to patients with eGFR ≥30 ml/min/1.73 m^2 when it is not recommended for patients with an eGFR below 45 ml/min/1.73 m2 in Europe. However, in the EMPA-REG OUTCOME trial, empagliflozin has been shown to reduce CV death in patients with T2D at high risk for CV events, irrespective of the baseline eGFR (down to 30 ml/min/1.73m^2) or KDIGO risk category (Zinman et al. 2015; Levin et al 2020). In addition, empagliflozin is currently being investigated in patients with CKD, with baseline eGFR as low as 20 ml/min/1.73m^2, in the EMPA-KIDNEY study (NCT03594110). Of note, American Diabetes Association (ADA) recommends the use of a SGLT2i in patients with an eGFR ≥30 mL/min/1.73 m^2 and urinary albumin >300 mg/g creatinine for patients with CKD and T2D (ADA 2021).

As noted above, both finerenone and empaglifozin treatment are associated with an initial drop of BP and decrease in eGFR (2 mL/min/1.73 m^2 and 3 mL/min/1.73 m^2, respectively). It is unknown what the effect of concomitant treatment with both products will be on these parameters. However, a subgroup analysis was performed in the FIDELIO-DKD study, including participants concomitantly treated with SGLT2i (6.6% in the finerenone arm and 7.6% in the placebo arm; data on file). Results for this subgroup analysis were consistent with the overall analysis in terms of AEs of worsening of kidney function, associated or not with hospitalization.

CONFIDENCE will be the first controlled clinical trial where finerenone and empagliflozin are simultaneously initiated. To minimize the risk to the participants, the study will consist of 2 consecutive parts:

- Part A: participants will be recruited if their eGFR is between 40 and 90 ml/min/1.73 m^2, and they will be equipped with an ambulatory BP monitoring (ABPM) device, 1 hour before the first intake at the study site. The participant will remain 4 to 6 hours at the study site for office BP monitoring and will keep the ABPM for 24 hours.
- Part B: participants will be recruited if their eGFR is between 30 and 90 ml/min/1.73 m^2, and they will not have an ABPM.
- The decision to move from Part A to Part B will be taken by the sponsor and the study's Steering Committee (SC) upon feedback from the Data Monitoring Committee (DMC). The safety analysis from the first 50 participants in Part A, as well as their unblinded review by the independent DMC will be used to confirm the enrollment/recruitment start for Part B.

eGFR will be monitored at each study visit with a first assessment 14 days after the initial intake. In addition, before inclusion of participants with eGFR as low as 30 ml/min/1.73 m^2, data from the first 50 study participants will be carefully evaluated by the independent DMC.

There is a risk of a severe acute respiratory syndrome coronavirus 2 (SARS-CoV-2) infection for study participants as long as the coronavirus disease 2019 (COVID-19) pandemic situation is ongoing. To minimize their infection risk during study participation, the investigators/sites will follow all recommendations issued by local authorities and guidelines aiming to reduce the risk of disease spreading. Details on the measures are specified by the site and agreed with the sponsor.

As part of the study procedures, participants will be closely monitored (including for signs of COVID-19) during the entire study duration. A pre-visit call before each in-person study visit is recommended to confirm the study participant does not have any symptoms suspicious for a SARS-CoV-2 infection.

Measures which prioritize participant safety and data validity are implemented. In case these 2 objectives conflict, participant safety always prevails.

During a pandemic situation, further measures according to recommendations and requirements from local health authorities may become necessary. These will be followed within the context of this study as far as applicable.

2.3.2 Benefit Assessment

Among patients with diabetes, those with kidney disease are consistently observed to have substantially elevated mortality rates. Most of this mortality is due to CV disease (CVD) with up to a 3-fold increase in 10-year CV mortality (Afkarian et al. 2013), although non-CV mortality is also increased. Decreased eGFR is associated with increased risks of CV events, CV mortality, and all-cause mortality (Tuttle et al. 2014; Matsushita et al. 2010; Go et al. 2004). There is a high unmet medical need for treatments that can reduce the extensive burden of CV mortality and morbidity and the progression of kidney disease in diabetic patients with CKD.

CKD in patients with T2D is the most frequent cause of ESKD in western countries (Fernandez et al. 2012). It is then important to slow down CKD progression at early stages to

prevent the development of ESKD and subsequent need for renal replacement therapy (GBD Chronic Kidney Disease Collaboration 2020).

Reduction in UACR, a surrogate marker for renal outcomes, is correlated with improved renal and cardiovascular outcomes. Therefore, early and efficient intervention assessed by UACR reduction might provide long-term benefits for patients with CKD and T2D. By taking part in this study, participants treated with finerenone and empagliflozin combination could benefit from a more effective treatment to reduce CKD progression together with decreased risk of adverse drug reactions.

Participants enrolled in the monotherapy arms could benefit of either finerenone or empagliflozin and may benefit from closer follow-up compared to real-world clinical practice. More detailed information about the known and expected benefits of both study interventions may be found in the IB for finerenone and in the EU SmPC for empagliflozin. All protocol-related procedures, including vital signs measurements, 12-lead electrocardiograms (ECGs), blood and urine sampling, are non-invasive or established routine assessments in the management of patients with CKD with T2D.

2.3.3 Overall Benefit: Risk Conclusion

Finerenone successfully delayed the onset of the composite of kidney failure, sustained eGFR decrease of ≥40% from baseline for at least 4 weeks and renal death in the phase 3 FIDELIO-DKD. At 3 years a chronic eGFR slope difference of 1.37 mL/min/1.73m^2 per year was seen, reflecting the long-term preservation of renal function with finerenone. An increased risk of hyperkalemia was observed with finerenone in a population with advanced CKD and T2D; however, the majority of these events were non-serious and the number of events leading to clinical consequences was low.

The main risk of finerenone treatment, hyperkalemia, has been proven to be manageable and efficacy was also consistent in participants at higher risk of hyperkalemia (i.e., with lower eGFR, higher baseline K+). Mitigation measures will also be in place in CONFIDENCE.

Based on the available data and the high medical need in the studied population, inclusion of participants into this study is considered favorable.

To date, empagliflozin is approved for the treatment of T2D, and to prevent CV events in this population. It is currently explored in a clinical trial in patients with CKD (NCT03594110).

The main risk of empagliflozin treatment are ketoacidosis, volume depletion, urosepsis and pyelonephritis, hypoglycemia with concomitant use with insulin and insulin secretagogues, necrotizing fasciitis of the perineum (Fournier’s Gangrene), genital mycotic infections, and hypersensitivity reactions. Empagliflozin is associated with an initial fall in BP and eGFR (mean eGFR decrease of 3 ml/min/1.73 m^2).

CONFIDENCE will be the first controlled clinical trial where finerenone and empagliflozin are simultaneously initiated. As mentioned above, initiation of each single drug can lead to a decrease in BP and eGFR. The additive effect of both drugs on BP and eGFR is not yet known. To monitor the risk of hypotension, all participants will be equipped with an ABPM device 1 hour before the first intake at the study site. The participant will remain 4 to 6 hours at the study site for office BP monitoring and will keep the ABPM for 24 hours. eGFR will be monitored at each study visit with a first assessment 14 days after the initial intake. With regards to risks specific to empagliflozin, participants with risks factors for ketoacidosis or volume depletion will be excluded from the study. Participants will also be monitored to look

for signs and symptoms of ketoacidosis. Concomitant therapy with insulin will also be carefully monitored and assessed during the medical review.

Reduction in UACR, a surrogate marker for renal outcomes, is correlated with improved renal and cardiovascular outcomes. Therefore, early and efficient intervention, resulting in slower disease progression and potentially prevention of disease progression, may provide long-term benefits for patients with CKD and T2D. By taking part in this study, participants treated with finerenone and empagliflozin combination could benefit from a more effective treatment to reduce CKD progression together with decreased risk of adverse drug reactions.

The clinical study will start after the consent of the Ethics Committees and the permission of the Competent Authorities have been obtained. All human investigations will be done in accordance to local law, Good Clinical Practice (GCP), and to the declaration of Helsinki (1964; last revised in 2013).

3. Objectives and Endpoints and Estimands

Objectives	Endpoints and Estimands
**Primary**	
• To demonstrate that combination therapy using finerenone and empagliflozin is superior in reducing UACR than either empagliflozin or finerenone alone	Primary Endpoints:<br>• Relative change from baseline in UACR at 180 days in combination therapy group versus empagliflozin alone<br>or<br>• Relative change from baseline in UACR at 180 days in combination therapy group versus finerenone alone
	Summary Measures:<br>• Mean ratio of change from baseline to Day 180 in UACR for the combination therapy group, to empagliflozin alone<br>• Mean ratio of change from baseline to Day 180 in UACR for the combination therapy group, to finerenone alone
**Secondary**	
• To further investigate the efficacy of combination therapy using finerenone and empagliflozin versus either finerenone or empagliflozin alone	• Relative change in UACR between end of treatment visit and 30 days after end of treatment visit<br>• Relative change in UACR between 30 days after end of treatment visit and baseline<br>• Relative change in UACR category (>30%, >40%, >50%) at 180 days

• To evaluate the safety of combination therapy using finerenone and empagliflozin versus either finerenone or empagliflozin alone	• Ratio of change from baseline in eGFR at 30 days<br>• eGFR decline greater than 30% at 30 days from baseline<br>• Ratio of change in eGFR at 180 days and 210 days from day 30<br>• Proportion of participants with of AKI events<br>• Total number of AKI events<br>• Proportion of participants with hyperkalemia events (moderate hyperkalemia [5.5 <K+ ≤6.0 mmol/L], severe hyperkalemia [K+ >6.0 mmol/L])<br>• Total number of hyperkalemia events (moderate hyperkalemia [5.5 <K+ ≤6.0 mmol/L], severe hyperkalemia [K+ >6.0 mmol/L])<br>• Change from baseline in K+<br>• Proportion of participants with severe hypoglycemia events<br>• Total number of events of severe hypoglycemia events<br>• Proportion of participants with symptomatic hypotension events<br>• Total number of symptomatic hypotension events<br>• Proportion of participants with genital mycotic events<br>• Total number of genital mycotic events<br>• Proportion of participants with ketoacidosis events<br>• Total number of ketoacidosis events<br>• Proportion of participants with necrotizing fasciitis of the perineum events<br>• Total number of necrotizing fasciitis of the perineum events<br>• Proportion of participants with urosepsis and pyelonephritis events<br>• Total number of urosepsis and pyelonephritis events
**Other exploratory**	
• To further investigate the study intervention (finerenone, empagliflozin) and similar drugs (e.g., mode-of-action-related effects, safety) and to further investigate pathomechanisms deemed relevant to CV disease, CKD, diabetes, and associated health problems	• Various biomarkers (e.g., diagnostic, safety, pharmacodynamic, monitoring, or potentially predictive biomarkers)
**Other pre-specified**	
• To characterize the PK of finerenone and empagliflozin when given in combination	• PK of finerenone and empagliflozin in plasma ($C_{max,md}$, $AUC_{\tau,md}$) (optional analysis)
Abbreviations: AKI = acute kidney injury; $AUC_{\tau,md}$ = area under the concentration vs. time curve for the expected dosing interval obtained after multiple dose administration; $C_{max,md}$ = maximum drug concentration after multiple dose administration; CV = cardiovascular; CKD = chronic kidney disease; eGFR = estimated glomerular filtration rate; K+ = serum/plasma potassium; PK = pharmacokinetics; UACR = urinary albumin-to-creatinine ratio.	

There are 2 primary endpoints to address the objective of the study: relative change from baseline in UACR at 180 days in combination therapy group versus empagliflozin alone and

relative change from baseline in UACR at 180 days in combination therapy group versus finerenone alone. These 2 primary endpoints are not considered as co-primary endpoints.

Primary Analysis Estimand

The assessment of efficacy will be done in the estimand framework, where the primary objective of the study is to assess the effect of combination therapy on the percentage reduction in UACR from baseline to Day 180 compared to its individual components if the treatment is taken according to the instructions as given in the protocol.

The estimand for assessing this study objective is defined by the following characteristics:

- *Population*: Adult participants with a clinical diagnosis of CKD and T2D.
- *Variable*: Ratio of change from baseline to Day 180 in UACR for the combination therapy group, to empagliflozin alone or finerenone alone.
- *Treatment*: Either finerenone (10 or 20 mg OD) and empagliflozin (10 mg OD) or finerenone (10 or 20 mg OD) or empagliflozin (10 mg OD), on top of standard of care (SoC) (treatment policy).
- *Summary measure*: Mean ratio of change from baseline to Day 180 in UACR for the combination therapy group, to empagliflozin alone or finerenone alone.
- *Intercurrent events*: the 3 most important intercurrent events are

  Treatment discontinuation

  - Treatment interruption due to reasons related to the treatment, such as hyperkalemia, will be handled using *treatment policy*

  Dialysis/kidney transplantation

  - Dialysis will be handled using a *hypothetical strategy*
  - Kidney transplantation will be handled using a *hypothetical strategy*

  Death

  - Death will be handled using *a hypothetical strategy*.

Hypothetical strategy relates to envisioning a scenario where the intercurrent event did not occur. Sensitivity analyses will be performed to observe scenarios in which the intercurrent events are handled differently.

Treatment policy strategy relates to participants being followed up for the remainder of the study after discontinuing treatment and data and follow-up time after discontinuation of treatment will be included in the analysis.

4. Study Design

4.1 Overall Design

- Phase 2, randomized, controlled, double-blind (participants and investigators), double-dummy, multicenter study in participants with CKD and T2D.
- Assuming a screening failure rate of approximately 50%, **1,614** participants will have to be screened to achieve **807** randomly assigned participants to study intervention (approximately **269** participants per group).

- The study will consist of 2 consecutive parts:
    - Part A: participants will be recruited if their eGFR is between 40 and 90 ml/min/1.73 m², and they will be equipped with an ABPM at Visit 2 for a duration of 24 hours. An interactive web response system (IWRS) will allow capping the number of participants as follows:
        - 80% with an eGFR between ≤75 ml/min/1.73 m²
        - 20% with an eGFR between >75 ml/min/1.73 m².
    - Part B: participants will be recruited if their eGFR is between 30 and 90 ml/min/1.73 m², and they will not have an ABPM. The IWRS will allow capping the number of participants as follows:
        - 80% with an eGFR between ≤75 ml/min/1.73 m²
        - 20% with an eGFR between >75 ml/min/1.73 m².
    - The decision to move from Part A to Part B will be taken by the sponsor and the study's SC upon feedback from the DMC. The safety analysis from the first 50 participants in Part A, as well as their unblinded review by the independent DMC will be used to confirm the enrollment/recruitment start for Part B. This decision shall be effective immediately or after IRB/IEC and/or local Health Authority approval, where applicable.
    - Other inclusion/exclusion criteria or study's schedule or procedure should not be affected.
- Participants will be randomized in a 1:1:1 ratio stratified by eGFR at screening (<60, ≥60 mL/min/1.73m²) and UACR (≤ 850mg/g, > 850 mg/g), using the baseline median from FIDELIO-DKD study) in one of the 3 parallel groups:
    - Finerenone (10 or 20 mg once daily [OD]) and empagliflozin (10 mg OD)
    - Finerenone (10 or 20 mg OD) and matching placebo to empagliflozin (OD)
    - Empagliflozin (10 mg OD) and matching placebo to finerenone (OD).
- The starting dose of finerenone will depend on the participant's eGFR level at the screening visit: a lower dose of 10 mg OD if eGFR is between <60 mL/min/1.73m², or the higher (target) dose of 20 mg OD if eGFR is ≥60 mL/min/1.73m².
- Finerenone dose will be defined and further adjusted based on K+ and eGFR values obtained from local laboratories (in exceptional cases where local laboratory results are not available, central laboratory values can be used), at each study visit. Necessary blood samples may be obtained up to 72 hours before a scheduled visit. Finerenone dose will also be adjusted, at investigator discretion based on any safety and tolerability concern.
- Participants should be treated for CKD and T2D according to local treatment guidelines. However, participants must not be exposed to an SGLT2i and/or a MRA within at least 8 weeks prior to screening. Participants should also be treated with the clinically maximum tolerated dose, as per investigator judgment, of ACEi or ARB, but not both, for more than 1 month at screening visit.
- A DMC will review safety data during the study (see Section 10.1.5.1).
- The total study duration for each participant will be approximately 7.5 months (up to 8.5 months if the optional pre-screening is performed):

  - A screening visit will occur up to 2 weeks before randomization, during which eligibility criteria will be checked. Eligibility criteria related to laboratory evaluation (e.g., UACR) will be assessed once results are available. Note: Sites will also have the option of a pre-screening visit to examine UACR. This UACR value will not be valid to determine eligibility and will only be used to assess whether the participant may progress to a formal screening visit.
  - Randomization will occur at Day 1 (baseline). Following visits will occur at Days 14 (±2), 30 (±4), 90 (±5), and 180 (±5; last day of intervention period). A follow-up/end of study (EOS) visit will be scheduled, 30 days after last dose (Day 210 ±5).
  - Note: Following up-titration or restart of study drug after interruption of finerenone intake for more than 7 days, the investigator will have to perform an unscheduled visit, 4 weeks (±7 days) after titration or restart, in order to monitor K+ levels and eGFR.
- The period between the participant's last intake of study intervention and last visit in the study is referred to as the 'follow-up period'. If a participant withdraws from study intervention permanently but does not withdraw from the study, this would apply to the period between the early discontinuation (ED) visit, which should take place as soon as possible following permanent discontinuation of study intervention, and the follow-up/EOS visit. In this case, the follow-up period will also last approximately 30 days.
- It is planned that all randomized participants will remain in the study unless one of the following occurs: consent withdrawal, ED of the study either by the sponsor or at the recommendation of the independent DMC (see Appendix 1).

4.2 Scientific Rationale for Study Design

This is a phase 2, randomized, controlled, double-blind, double-dummy, multicenter study to investigate the efficacy and safety of the finerenone and empagliflozin combination compared to respective monotherapies in reducing UACR in participants with T2D and CKD.

Participants and investigators will be blinded to study intervention allocation ensuring a double-blind design, thus limiting bias.

The primary surrogate endpoints will be relative change from baseline at 180 days of UACR in finerenone plus empagliflozin arm versus each of the monotherapies. UACR is a measurement of albuminuria, a predictor of long-term renal and CV adverse outcomes in T2D patients (Fox 2012; Heerspink et al. 2019). Post-hoc analyses of randomized clinical trials (Heerspink et al. 2014; Levey et al. 2019) suggest that a meaningful reduction in albuminuria may translate to protection from CV events and declining renal function in patients with diabetic and non-diabetic renal disease with albuminuria. For every 30% reduction in albuminuria, one can expect the risk of ESKD to decreased by 23.7% (95% confidence interval [CI], 11.4% to 34.2%; p=0.001) (Heerspink et al. 2019). Changes in UACR can be detected early in response to treatment. Therefore, change in UACR at Day 180 is considered as an appropriate endpoint.

The study aims at showing an additive effect on UACR when finerenone and empagliflozin are taken together. The monotherapy arms will allow the comparison between the combination and each monotherapy to demonstrate this additive effect.

Given that this is the first time that the simultaneous initiation of finerenone and a SGLT2i is being investigated, the sponsor made the decision to protect the participants' safety by implementing a higher threshold for eGFR at entry in the study, combined with an ABPM. These measures are implemented to address the risks of AKI and symptomatic hypotension. Upon unblinded data review by the DMC, these measures shall be relieved and subsequently lead to the second phase of the trial with a lower eGFR threshold. The eGFR eligibility criteria of 30 mL/min/1.73m² is based on the lowest eGFR threshold recommended by current guidelines on the introduction of empagliflozin in this patient population.

4.2.1 Participant's Input into Design

Participants were not involved in the design of this study.

4.3 Justification for Dose

4.3.1 Finerenone

The starting dose of finerenone will be:

- 20 mg OD if eGFR ≥60 mL/min/1.73 m^2
- 10 mg OD if eGFR <60 mL/min/1.73 m^2.

The starting dose will be established based on screening eGFR results, obtained from the central laboratory (see Section 8.2.4).

Up and down-titrations will be allowed during the study and will be based on local laboratory results (see Section 6.5). In exceptional cases where local laboratory results are not available, central laboratory values can be used. Note: up-titration will be allowed from Visit 4 onward, only.

4.3.2 Empagliflozin

In EU SmPC, the recommended dose of Jardiance is 10 mg once daily, taken with or without food. The up-titration to 25 mg is only recommended for glucose control, which is not the aim of the study.

In 2016, Cherney et al. conducted a pooled analysis on the effect of empagliflozin on UACR in T2D patients with either microalbuminuria (30-300 mg/g) or macroalbuminuria (>300 mg/g), enrolled in 1 of the 5 phase 3 trials with primary endpoints related to glucose lowering (Cherney et al. 2016). In 4 studies, patients with eGFR ≥30 ml/min/1.73 m^2 were randomized to receive empagliflozin 10 mg, empagliflozin 25 mg, or placebo. In the fifth study (EMPA-REG RENAL), which included individuals with CKD and T2D, patients with CKD stage 2 received empagliflozin 10 mg, empagliflozin 25 mg, or placebo, and patients with CKD stage 3 or stage 4 received empagliflozin 25 mg or placebo. The authors pooled a total of 388 subjects with microalbuminuria and 128 with macroalbuminuria. The authors observed a reduction of UACR at 24 weeks by 32% in the microalbuminuric subjects, and 41% in the macroalbuminuric subjects. The effect was considered the same irrespective of baseline DBP, sex, body mass index (BMI), race, weight and baseline renin-angiotensin-aldosterone system inhibitors (RAASi) use. The authors also concluded that there was no difference in the reduction of UACR between the 2 different dosages of empagliflozin.

In 2017, Cherney et al. conducted a post-hoc analysis of EMPA-REG-outcome (Cherney et al. 2017) in 7,028 patients with T2D and CVD. Subjects were randomized in each study to placebo, empagliflozin 10 mg, or empagliflozin 25 mg. In this paper, the authors present the

UACR results for the pooled empagliflozin group versus placebo according to albuminuria status at baseline. A total of 1,338 subjects in the pooled empagliflozin group (10 and 25 mg) had microalbuminuria at baseline and 509 had macroalbuminuria. Results of the analysis show a reduction of UACR by 25% in microalbuminuric subjects, and 32% in macroalbuminuric subjects. In a subsequent analysis per dosage, there were no significant differences between 10 and 25 mg.

In summary, available evidence suggests comparable UACR reduction by 10 and 25 mg of empagliflozin. Therefore, this study will use the lowest registered dose of 10 mg empagliflozin, which is also the dose selected for the ongoing event-driven EMPA-Kidney study investigating the effect of empagliflozin in patients with CKD (with and without diabetes) (Herrington et al. 2018).

4.4 End of Study Definition

The EOS is defined as the date of the last visit of the last participant in the study.

The primary completion date is defined as the date when the final participant was examined or received an intervention for the purposes of final collection of data for the primary outcome.

A participant is considered to have completed the study if he/she has completed all phases of the study including the EOS visit.

5. Study Population

Prospective approval of protocol deviations to recruitment and enrollment criteria, also known as protocol waivers or exemptions, is not permitted.

5.1 Inclusion Criteria

Participants are eligible to be included in the study only if all of the following criteria apply:

Age

1. Participant must be 18 years of age or older at screening visit. The lower age limit may be higher if legally required in the participating country.

Type of Participant and Disease Characteristics

2. Participant with a clinical diagnosis of CKD and the following:

   2a:

   - In Part A: eGFR 40-90 ml/min/1.73m^2 (with no more than 20% having an eGFR >75 ml/min/1.73m^2) using Chronic Kidney Disease Epidemiology Collaboration (CKD-EPI) formula (Levey et al. 2009) at screening visit and at least one historical value of eGFR <60 mL/min/1.73 m^2 within 3 months or have a registered diagnosis of CKD.
   - In Part B: eGFR 30-90 ml/min/1.73m^2 (with no more than 20% having an eGFR >75 ml/min/1.73m^2) using CKD-EPI formula (Levey et al. 2009) at screening visit and at least one historical value of eGFR <60 mL/min/1.73 m^2 within 3 months or have a registered diagnostic of CKD (see Section 2.2).

   2b:

   - 100 ≤UACR <5000 mg/g at screening visit (mean value from 3 morning void samples) and documentation of albuminuria/proteinuria (quantitative or

semi-quantitative measurement) in the participant’s medical records at least 3 months prior to screening

Note: One re-assessment of eGFR and/or UACR is allowed at the screening visit (see Section 5.4). If one of the 3 UACR measurements is missing but the other 2 are valid, these values can be used to assess participant’s eligibility.

3. Participant with T2D as defined by the ADA (ADA 2021), with glycated hemoglobin (HbA1c) at screening <11%. Note: Historical values for HbA1c will be acceptable for inclusion providing they have been obtained within 3 months prior to screening visit.
4. Participant treated with the clinically maximum tolerated dose, as per investigator judgment, of ACEi or ARB, but not both, for more than 1 month at screening visit (see Section 6.8.1).

Sex and Contraceptive/Barrier Requirements

5. Male or female

   Contraceptive use by women should be consistent with local regulations regarding the methods of contraception for those participating in clinical studies.

   - Female participants:
     - Women of childbearing potential (see Appendix 6) can only be included in the study if a pregnancy test is negative at the screening visit and if they agree to use adequate contraception during the study and until 8 weeks after last study interventions dose. Adequate contraception is defined as any combination of at least 2 effective methods of birth control, of which at least one is a physical barrier (e.g., condoms with hormonal contraception or implants or combined oral contraceptives, certain intrauterine devices).
     - Postmenopausal females (no menses for 12 months without an alternative medical cause; see Appendix 6) are not required to use contraception. A high follicle stimulating hormone (FSH) level in the postmenopausal range may be used to confirm a postmenopausal state in women not using hormonal contraception or hormonal replacement therapy (HRT). However, in the absence of 12 months of amenorrhea, confirmation with more than one FSH measurement is required.
     - Females on HRT and whose menopausal status is in doubt will be required to use one of the non-estrogen hormonal highly effective contraception methods if they wish to continue their HRT during the study. Otherwise, they must discontinue HRT to allow confirmation of postmenopausal status before study enrollment.
     - Females of non-childbearing potential (documented hysterectomy, documented bilateral salpingectomy, documented bilateral oophorectomy) are not required to use contraception.
     - For females with permanent infertility due to an alternate medical cause other than the above, (e.g., mullerian agenesis, androgen insensitivity), investigator discretion should be applied to determining study entry.

Informed Consent

6. Capable of giving signed informed consent as described in Appendix 1 which includes compliance with the requirements and restrictions listed in the informed consent form (ICF) and in this protocol.

5.2 Exclusion Criteria

Participants are excluded from the study if any of the following criteria apply:

Medical Conditions

1. Participants with type 1 diabetes (T1D).
2. Participant with known allergies to finerenone or any SGLT2i.
3. Participant with hepatic insufficiency classified as Child Pugh C (see Appendix 7).
4. Participant with BP at Day 1 visit (Visit 2) higher than 160 SBP or 100 DBP or SBP lower than 90 mmHg.
5. Known bilateral clinically relevant renal artery stenosis (>75%) or other non-diabetic renal disease.
6. Renal allograft in place or a scheduled kidney transplant.
7. Participant with acute kidney injury (AKI) within 6 months prior to screening.
8. Participant with ketoacidosis in the past 5 years.
9. Participant with primary adrenal insufficiency (Addison’s disease).
10. Stroke, transient ischemic cerebral attack, acute coronary syndrome (MI, coronary artery bypass graft [CABG], primary percutaneous coronary intervention [PCI]), or hospitalization for worsening HF, within 90 days prior to screening visit.
11. Clinical diagnosis of chronic heart failure with reduced ejection fraction (HFrEF) and persistent symptoms (New York Heart Association class II - IV) at the screening visit (class 1A recommendation for MRAs)
12. Major surgery (major according to the investigator’s assessment) performed within 90 days prior to screening visit, or scheduled major elective surgery (e.g., hip replacement) within 90 days after screening visit.
13. Gastrointestinal surgery or gastrointestinal disorder that could interfere with absorption of trial medication in the investigator’s opinion.
14. Any other history, condition, therapy, or uncontrolled intercurrent illness (including AKI) which could in the opinion of the investigator affect participant safety compliance with study requirements.

Prior/Concomitant Therapy

15. Participant currently treated with a SGLT2i or combined SGLT-1 and 2 inhibitor (SGLT-1/2i) or who received a SGLT2i or SGLT-1/2i which cannot be discontinued at least 8 weeks prior to the screening visit and during study intervention treatment.

16. Participant treated with strong cytochrome P450 isoenzyme 3A4 (CYP3A4) inhibitors or inducers which cannot be discontinued 7 days before Day 1 visit (see Appendix 8).
17. Participant treated with another MRA (e.g., eplerenone, esaxerenone, spironolactone, canrenone), a renin inhibitor, K+ supplements, a K+ sparing diuretic (e.g., amiloride, triamterene), a K+ binder agent, or angiotensin receptor-neprilysin inhibitor (ARNI) within 8 weeks prior of the screening visit and during study intervention treatment[1].
18. Participants currently treated or who were treated with finerenone (Kerendia®) within 8 weeks prior to the screening visit.

Prior/Concurrent Clinical Study Experience

19. Participation in another clinical trial with an investigational product within 1 month prior to screening visit[2].

Diagnostic Assessments

20. Participant with K+ above 4.8 mmol/L at screening visit (central laboratory value). Note: 1 re-assessment of K+ is allowed at the screening visit.
21. Participants with alanine aminotransferase (ALT) or aspartate aminotransferase (AST) >3x upper limit of normal (ULN) at screening visit.

Other Exclusions

22. Breastfeeding female participant.
23. Participant known for lack of compliance with clinic visits or prescribed medication.

5.3 Lifestyle Considerations

5.3.1 Meals and Dietary Restrictions

Participants must refrain from consumption of grapefruit or grapefruit juice from the start of study intervention until after the final dose as it is expected to increase plasma concentration of finerenone.

5.3.2 Other Lifestyle Considerations

There are no additional lifestyle restrictions other than the dietary restrictions mentioned in Section 5.3.1.

5.4 Screen Failures

Screen failures are defined as participants who consent to participate in the clinical study but are not subsequently randomly assigned to study intervention. A minimal set of screen failure information is required to ensure transparent reporting of screen failure participants to meet the Consolidated Standards of Reporting Trials publishing requirements and to respond to

[1] K+ supplements and potassium binder agents will be allowed during the study for safety reasons (see Section 6.8.1).
[2] Participants who received a COVID-19 vaccine whilst still under Emergency Use Utilization will be eligible, provided vaccination occurred at least 1 month prior to screening visit.

queries from regulatory authorities. Minimal information includes demography, screen failure details, eligibility criteria, and any SAE.

Individuals who do not meet the criteria for participation in this study (screen failure) may be rescreened once. A minimum of 1 month between the initial screening and re-screening is required. In addition, 1 re-assessment of K+ and/or eGFR and/or UACR will be allowed at screening if the test results fall outside of the ranges defined for inclusion in the study.

Sites will also have the option of 1 pre-screening visit to examine UACR. This UACR value is not valid to determine eligibility and is only to be used to assess whether the participant may progress to a formal screening visit. Participants will be required to provide informed consent for this UACR test, and very limited data will be reported. Such pre-screening failures will not be considered as part of the study population. See Section 1.3 for assessments at the pre-screening visit.

5.5 Criteria for Temporarily Delaying Enrollment/Randomization/Study Intervention Administration

Participants can be enrolled out of the screening window if central laboratory results are pending. Once central laboratory results have been reviewed by the investigator, participant eligibility will be confirmed, even if he/she is out of screening window.

Current febrile illness (temperature 38.0°C [100.4° F]) or other acute illness within 48 hours before study intervention administration may also allow a participant to be randomized once the condition has resolved and the participant is otherwise eligible.

6. Study Intervention(s) and Concomitant Therapy

Study intervention is defined as any investigational intervention(s), marketed product(s), placebo, or medical device(s) intended to be administered to a study participant according to the study protocol.

6.1 Study Interventions Administered

6.1.1 Study Interventions

Finerenone 10 and 20 mg and matching placebo, as well as over-capsulated empagliflozin tablets and matching placebo, will be supplied by the sponsor or designee.

Following a screening period of up to 2 weeks, eligible participants will be randomized in a 1:1:1 ratio to receive:

- Finerenone tablet and empagliflozin over-capsulated OD (finerenone + empagliflozin arm), OR
- Finerenone tablet and matching placebo to empagliflozin OD (finerenone arm), OR
- Empagliflozin over-capsulated and matching placebo to finerenone OD (empagliflozin arm).

The randomization will be stratified by eGFR at screening (<60, ≥60 mL/min/1.73m²) and UACR at screening (≤ 850mg/g, > 850 mg/g).

See Section 4.3.1 and Section 4.3.2 for starting dose of finerenone and empagliflozin, respectively.

Study interventions will be taken in the morning, preferably in the morning at approximately the same time each day, with or without food.

The following instructions will be given to the participant in case of missed intake:

- If >8 hours before the next scheduled dose, the participant should take study interventions as soon as possible.
- If ≤8 hours of the next scheduled dose, the participant should wait and take the next study interventions at the usual time.

Arm Name	Finerenone plus Empagliflozin		Finerenone		Empagliflozin	
**Intervention Name**	Finerenone	Empagliflozin	Finerenone	Placebo for empagliflozin	Placebo for finerenone	Empagliflozin
**Type**	Drug	Drug	Drug	Drug	Drug	Drug
**Dose Formulation**	Tablet	Over-capsulated tablet	Tablet	Over-capsulated tablet	Tablet	Over-capsulated tablet
**Unit Dose Strength(s)**	10 and 20 mg	10 mg	10 and 20 mg	N/A	N/A	10 mg
**Dosage Level(s)**	One tablet daily	One capsule daily	One tablet daily	One capsule daily	One tablet daily	One capsule daily
**Route of Administration**	Oral	Oral	Oral	Oral	Oral	Oral
**Use**	Experimental	Experimental	Experimental	Placebo	Placebo	Experimental
**Packaging and Labeling**	Study Intervention will be provided in bottles. Each bottle will be labeled as required per country requirement	Study Intervention will be provided in bottles. Each bottle will be labeled as required per country requirement	Study Intervention will be provided in bottles. Each bottle will be labeled as required per country requirement	Study Intervention will be provided in bottles. Each bottle will be labeled as required per country requirement	Study Intervention will be provided in bottles. Each bottle will be labeled as required per country requirement	Study Intervention will be provided in bottles. Each bottle will be labeled as required per country requirement
**Current/Former Name(s) or Alias(es)**	BAY 94–8862- Kerendia	BI 10773- Jardiance	BAY 94–8862- Kerendia	N/A	N/A	BI 10773 - Jardiance

6.1.2 Medical Devices

No sponsor manufactured devices or devices manufactured for the sponsor are used in this study. Other medical device (not manufactured by or for sponsor) provided for use in this study is the ABPM.

Instructions for ABPM use are provided in the corresponding manual. All device deficiencies (including malfunction, use error and inadequate labeling) that caused or could have caused a SAE to a study participant shall be documented and reported by the investigator throughout the study and appropriately managed by the sponsor.

A device deficiency is an inadequacy of a medical device with respect to its identity, quality, durability, reliability, safety, or performance. Device deficiencies include malfunctions, use errors, and inadequate labeling.

For any device deficiencies related to a study participant AE or SAE: the investigator should complete the AE case report form (CRF) and safety reports (complementary pages) in addition to the Medical Device Incident CRF.

6.2 Preparation/Handling/Storage/Accountability

1. The investigator or designee must confirm appropriate temperature conditions have been maintained during transit for all study interventions received and any discrepancies are reported and resolved before use of the study interventions.
2. Only participants randomized in the study may receive study interventions and only authorized site staff may supply or administer study intervention. All study interventions must be stored in a secure, environmentally controlled, and monitored (manual or automated) area in accordance with the labeled storage conditions with access limited to the investigator and authorized site staff.
3. The investigator or the head of the institution (where applicable) is responsible for study intervention accountability, reconciliation, and record maintenance (i.e., receipt, reconciliation, and final disposition records). Drug return, reconciliation and destruction information will be captured in the IWRS.
4. Returned study intervention should not be re-dispensed to the participants.
5. In the event of a significant trial-continuity issue (e.g., caused by a pandemic), site-to-patient distribution of study interventions might occur at selected sites and visits. The investigator will maintain responsibility and control for dispensing via IxRS. The sponsor will contract a distribution provider to conduct site-to-patient distribution of study interventions, including the delegation of storage and handling as needed until handover to the participant.
6. Further guidance and information for the final disposition of unused study interventions are provided in a separate document.

6.3 Measures to Minimize Bias: Randomization and Blinding

All participants will be centrally assigned to randomized study interventions at Day 1, using an IWRS. Before the study is initiated, the telephone number and call-in directions for the IRT and/or the log in information and directions for the IWRS will be provided to each site. Once a randomization number has been assigned it must not be re-assigned.

Following enrollment of the participant, the site will contact the IWRS prior to the start of study intervention administration for each participant.

The IWRS will determine the bottle number for the study site investigator or designee to select for the participant. The investigator is to instruct participants to take 1 capsule/tablet of each study intervention, OD, preferably in the morning. The first dose of study interventions should be taken at the site on Day 1.

Study interventions will be assigned by IWRS and dispensed at the study visits summarized in the Schedule of Activities (SoA; see Section 1.3) and after the eGFR and K+ results have been made available to the investigator.

Participants will be randomly assigned in a 1:1:1 ratio to receive study interventions. The randomization will be stratified by eGFR at screening (<60, ≥60 mL/min/1.73m²) and UACR at screening (≤ 850mg/g, > 850 mg/g). The IWRS will allow to cap the number of participants with an eGFR >75 mL/min/1.73m² to 20%. Investigators and participants will remain blinded to each participant's assigned study intervention throughout the course of the study. Each study intervention and its matching placebo will be identical in appearance (size, shape, color). The packaging and labeling will be designed to maintain the blinding of the investigator's team and the participants. The study data will remain blinded until database lock and authorization of data release according to standard operating procedures.

Appropriate measures will be taken to maintain blinding while bioanalysis is ongoing.

The IWRS will be programmed with blind-breaking instructions. In case of an emergency, the investigator has the responsibility for determining if unblinding of a participant's intervention assignment is warranted. If the investigator is unavailable, and a treating physician not associated with the study requests emergency unblinding, the emergency unblinding requests are forwarded to the emergency medical advice 24-hours/7-day service. Participant safety must always be the first consideration in making such a determination. If the investigator decides that unblinding is warranted, the investigator should make every effort to contact the sponsor prior to unblinding a participant's intervention assignment unless this could delay emergency treatment of the participant. If a participant's intervention assignment is unblinded, the sponsor must be notified within 24 hours after breaking the blind. The date and reason that the blind was broken must be recorded in the source documentation and CRF, as applicable.

Sponsor safety staff may unblind the intervention assignment for any participant with an SAE. If the SAE requires that an expedited regulatory report be sent to one or more regulatory agencies, a copy of the report, identifying the participant's intervention assignment, may be sent to investigators in accordance with local regulations and/or sponsor policy.

6.4 Study Intervention Compliance

Compliance with study interventions will be assessed at each visit. Compliance will be assessed by direct questioning and counting returned capsules/tablets during the site visits and documented in the source documents and relevant form. Deviations from the prescribed dosage regimen should be recorded.

A record of the quantity of each study intervention dispensed to and administered by each participant must be maintained and reconciled with study intervention and compliance records. Intervention start and stop dates, including dates for intervention delays and/or dose reductions/increases will also be recorded.

6.5 Dose Modification

6.5.1 Finerenone

Dosing and titration follow the guidance in place for finerenone.

The investigator is encouraged to up-titrate the dose of study drug, at any time once the participant has been on a stable dose for 4 weeks (±4 days, e.g., from Visit 4 onwards for participant starting study drug on the lower dose) and at any visit. Up-titration shall be performed provided:

- The serum/plasma K^+ concentration (local laboratory value) is ≤4.8 mmol/L,

AND

- eGFR decrease (local laboratory value) is less than 30% below the value measured at the last regular visit.

K^+ and eGFR values obtained from local laboratory will be used for finerenone up-/down-titration as well as monitoring after down/up-titration and restart. In exceptional cases where local laboratory results are not available, central laboratory values can be used. Potassium and eGFR values used for up-/down-titration must be documented in the electronic CRF (eCRF).

Dose adjustments of finerenone are described in Table 6-1.

Table 6-1: Dose Adjustments of Finerenone

**eGFR value at the screening visit, based on central laboratory results:**	**<60 mL/min/1.73m^2**		**≥60 mL/min/1.73m^2**	
**Participant randomized to group receives**	**Finerenone**<br>10 mg finerenone OD	**Placebo**<br>Placebo OD	**Finerenone**<br>20 mg finerenone OD	**Placebo**<br>Placebo OD
**Up-titration of dose** allowed from Visit 4 onwards provided that:<br>• K+ is ≤4.8 mmol/L[a]<br>• eGFR decrease is less than 30% below the value measured at the last scheduled visit[a]<br>• Must be documented in eCRF	20 mg finerenone OD	Sham-titrate	Not applicable	Not applicable
**Down-titration of dose**:<br>• Only for safety reasons (for guidance, see Table 6-2 and Section 8.2.5.2)<br>• Allowed any time during the study (e.g., between scheduled visits)<br>• Must be documented in eCRF<br>• An unscheduled safety visit is performed within an adequate timeframe proposed by the investigator	• If at higher dose of finerenone, down-titrate to lower dose of finerenone<br>• If at lower dose of finerenone, interrupt finerenone			

Abbreviations: eCRF = electronic case report form; eGFR = estimated glomerular filtration rate; K+ = serum/plasma potassium; OD = once daily.
NOTE: lower dose = 10 mg once daily; higher dose = 20 mg once daily.
[a] K+ and eGFR according to local laboratory values (in exceptional cases where local laboratory results are not available, central laboratory values can be used).

Adjustment of dose after start of study drug intake based on blood serum K+ levels are provided in Table 6-2.

Table 6-2: Guidance for Finerenone Dose Adjustment Based on K+

K+ (mmol/L)	Action
**First sample:**	
≤4.8	**Before Visit 4:** continue on the same dose.<br><br>**From Visit 4 and subsequent visits**:<br>If on lower dose of study drug, up-titrate to higher dose.<br>If on higher dose of study drug, continue on the same dose.
4.9 to 5.5	Continue on the same dose.
>5.5	Withhold study drug and re-check K+ within 72 hours.
**Second and subsequent samples:**	
≤5.0	Restart study drug at lower dose.
>5.0	Continue to withhold study drug; continue to monitor K+ and restart study drug at the lower dose only if K+ is ≤5.0.

Abbreviations: K+ = serum/plasma potassium.
NOTE 1: lower dose = 10 mg once daily; higher dose = 20 mg once daily.
NOTE 2: in exceptional cases where local laboratory results are not available, central laboratory values can be used.

The following aspects have also to be taken into consideration:

- K+ should be measured 4 weeks (±7 days) after restarting treatment or dose adjustment, especially after up-titration.
- Whenever K+ or eGFR are checked locally during an unscheduled visit, these results should also be checked centrally (see Section 8.2.5.2).
- If central laboratory results for K+ differ from local laboratory results to such an extent that the investigator is uncomfortable with the dose adjustment guidance shown in Table 6-2, blood sampling may be repeated at the investigator's discretion.
- If the participant is already on the lower dose of study drug but hyperkalemia recurs soon after a previous event of hyperkalemia after restarting study intervention following interruption, and there is no explanation for the recurring hyperkalemia event other than intake of study intervention, permanent discontinuation of study intervention is recommended.
- If K+ is >6.5 mmol/L, an ECG should be obtained.
- Participants will maintain their normal diet throughout the study and will not be given any specific advice on dietary K+ restrictions.

Participants who started with or were up-titrated to the target dose (20 mg OD) of finerenone but who do not tolerate this dose may be down-titrated at any point during the study if required for safety reasons. These participants may be up-titrated again based on the rules provided above. If the participant is already at the lower dose, finerenone can be interrupted at the investigator's discretion (refer to Table 6-1 for guidance). If finerenone is interrupted for more than 7 days, it should be restarted at the lower dose (10 mg OD).

Subsequent to an up-titration or restart of study drug after interruption of finerenone intake for more than 7 days, the investigator should perform an unscheduled visit, 4 weeks (±7 days) after titration or restart, in order to monitor K+ levels and eGFR. If a regular study visit is scheduled to take place 4 weeks (±7 days) after up-titration, the monitoring of K+ and eGFR is assured and no unscheduled visit must be performed in addition. With down-titrations, the unscheduled safety visit may need a smaller time window and should be performed at the investigator's discretion.

At any point during study drug treatment, if K+ is found to be elevated, finerenone treatment should be adjusted according to guidance provided in Table 6-2. Both up-/down-titrations will be at the investigator's discretion and will depend on the general clinical condition of the individual participant. The investigator should attempt to reach the maximum target dose of study drug if safety is not compromised.

All titrations, including the reasons for down-titration or for not up-titrating to the 20-mg dose, must be documented in the eCRF.

6.5.2 Empagliflozin

There are no specific dosing requirements for empagliflozin.

If additional glycemic control is required, other anti-diabetic agents may be added or doses adjusted at the discretion of the principal investigator according to local guidelines (see Section 6.8.1).

6.6 Continued Access to Study Intervention After the End of the Study

At study completion, the investigator will decide in consultation with the individual participant if additional treatment is required and choose from existing treatment options. The investigator must provide follow-up medical care for all participants who complete the study or who are prematurely withdrawn from the study or must refer them for appropriate ongoing care as required.

6.7 Treatment of Overdose

6.7.1 Finerenone

No cases of AEs associated with finerenone overdose in humans have been reported during its development. The most likely manifestation of overdose is anticipated to be hyperkalemia. If hyperkalemia develops, standard treatment will be initiated, as per local guidelines, and action with regards to study drug should be taken as mentioned in Section 6.5.1.

6.7.2 Empagliflozin

6.7.2.1 Symptoms

In controlled clinical studies, single doses of up to 800 mg empagliflozin in healthy volunteers and multiple daily doses of up to 100 mg empagliflozin in patients with T2D did not show any toxicity. Empagliflozin increased urine glucose excretion leading to an increase in urine volume. The observed increase in urine volume was not dose dependent and is not clinically meaningful. There is no experience with doses above 800 mg in humans (EU SmPC).

6.7.2.2 Therapy

In the event of an overdose, treatment should be initiated as appropriate to the participant clinical status.

The removal of empagliflozin by hemodialysis has not been studied (EU SmPC).

In the event of an overdose, the investigator should:

- Contact the medical monitor immediately.
- Evaluate the participant to determine, in consultation with the medical monitor, whether study intervention should be interrupted.
- Closely monitor the participant for any AE/SAE (at least 48 hours).
- Document the quantity of the excess dose as well as the duration of the overdose in the eCRF.

Decisions regarding dose interruptions or modifications will be made by the investigator in consultation with the medical monitor based on the clinical evaluation of the participant.

6.8 Concomitant Therapy

All treatment that the investigator considers necessary for the participant’s welfare may be administered at the discretion of the investigator in keeping with the standards of medical care.

ACEis or ARBs are considered as SoC therapy in patients with CKD and T2D, and often prescribed to patients with CKD at early stages (Molitch et al. 2014).

It is advisable to follow the recommendations of local guidelines for the management of CVD and CKD, the use of statins, anti-platelets, and beta-blockers, and for glycemic control.

The medical monitor should be contacted if there are any questions regarding concomitant or prior therapy.

Any medication or vaccine (including over the counter or prescription medicines, recreational drugs, vitamins, traditional Chinese medicines, and/or herbal supplements) that the participant is receiving at the time of screening or receives during the study must be recorded along with:

- Reason for use
- Dates of administration including start and end dates
- Dosage information including dose and frequency.

6.8.1 Permitted Concomitant Therapy

The following concomitant therapies will be permitted during the study:

- Insulin, insulin secretagogues, and thiazolidinediones (TZD). For participants treated with a sulfonylurea or with insulin, a lower dose of the sulfonylurea or insulin will have to be considered by the investigator to reduce the risk of hypoglycemia.
- ACEis or ARBs at stable doses; change in ACEis or ARBs doses will be allowed for safety purposes, only. The maximum clinically tolerated dose for ACEis or ARBs should be the highest dose, according to local labels, which the participant can safely tolerate. This dose should not be below the minimum labeled dose to maximize therapeutic benefit of background SoC.
- Antihypertensive therapy will be administered according to local guidelines. Depending on BP, serum creatinine, or eGFR, participants may need to have their study intervention dose, or the dose of another concomitant medication reduced or

discontinued. The dosage of SoC therapies should not be reduced to solely facilitate maintenance of study intervention.

- Loop diuretics and thiazides should be carefully evaluated before inclusion; if participant is taking loop diuretic at study inclusion, diuretic dose should be adjusted appropriately, according to PI discretion. Loop diuretic and thiazides dosages should be stable during the study. Changes in dosage will be allowed for safety purposes (see Section 6.8.5).
- Treatments of hyperkalemia as per local guideline recommendations.
  - K+ lowering agents (e.g., sodium polystyrene sulfonate, calcium polystyrene sulfonate, patiromer, sodium zirconium cyclosilicate) are allowed to be started.
- Treatments of hypoglycemia as per local guideline recommendations.
- Treatments of hypotension as per local guideline recommendations (see Section 6.8.5).
- Glucagon-like peptide-1 receptor agonist treatments are allowed if dose was stable for at least 4 weeks prior to the screening visit. Dose reductions (down-titration) will be allowed during the study. Dose increases (up-titrations) will not be allowed. If needed, the investigators should privilege the use of insulin or TZD to control HbA1c.

Trimethoprim and trimethoprim-sulfamethoxazole can be used with caution. Increased K+ monitoring and potential temporary discontinuation of finerenone may be advised.

6.8.2 Prohibited Concomitant Therapy

The following concomitant therapies will be prohibited during the study. Participants should be discontinued from the study if receiving prohibited concomitant therapy.

- K+ sparing diuretics (e.g., amiloride, triamterene)
- Marketed finerenone (Kerendia®)
- Other MRAs (e.g., eplerenone, esaxerenone, spironolactone, canrenone)
- Any renin inhibitor (e.g., aliskiren)
- ARNI
- Concomitant therapy with both an ACEi and an ARB
- Strong CYP3A4 inhibitors (see Appendix 8)
- Any other SGLT-2i or SGLT-1/2i during as long as the participant is taking study interventions and up to 7 days after last study intervention intake.

6.8.3 Hyperkalemia Events

See Section 6.5.1.

6.8.4 Unexpected Acute Declines in eGFR

If an unexpected, acute decline in kidney function is observed, the participant should be evaluated. Volume depletion, hypotension, intercurrent medical problems and concomitant drugs may cause increases in serum creatinine. Urinary tract infection and urinary obstruction should be considered (the latter especially in men). Several drugs may cause a decline in kidney function, especially nonsteroidal anti-inflammatory drugs and certain antibiotics such as trimethoprim. If any drug is suspected of causing or contributing to worsening kidney function, their use should be re-considered.

6.8.5 Volume Depletion/Hypotension

Participants with clinically relevant symptoms/signs of suspected volume depletion and/or hypotension, should have their regular medication reviewed, and consideration given to reducing the dose of, or stopping concomitant non-essential medications, as assessed on an individual basis, including diuretics and drugs that lower BP (except ACEi, ARB, or beta-blockers prescribed for HF). The need for conventional diuretics (or the dose of diuretic used) should be reevaluated considering the participant's symptoms and signs. In participants with HF, discontinuation of diuretic should only be undertaken cautiously. Hypotension may also occur with other BP lowering drugs and once again the need for (and dose of) non-essential agents of this type (e.g., calcium channel blockers, alpha adrenoceptor antagonists, and nitrates) should also be re-considered.

6.8.6 Ketoacidosis

Reports of ketoacidosis, a serious life-threatening condition requiring urgent hospitalization have been identified in clinical trials and post marketing surveillance in patients with T1D and T2D mellitus receiving SGLT2i, including empagliflozin. Fatal cases of ketoacidosis have been reported in patients taking empagliflozin.

Participants treated with study interventions who present with signs and symptoms consistent with severe metabolic acidosis should be assessed for ketoacidosis regardless of presenting blood glucose levels, as ketoacidosis associated with empagliflozin may be present even if blood glucose levels are less than 250 mg/dL. If ketoacidosis is suspected, study interventions should be discontinued, participant should be evaluated, and prompt treatment should be instituted. Treatment of ketoacidosis may require insulin, fluid, and carbohydrate replacement. In case of ketoacidosis, the investigator will investigate whether the participant is not suffering from T1D instead of T2D.

In many of the post-marketing reports, and particularly in patients with T1D, the presence of ketoacidosis was not immediately recognized and institution of treatment was delayed because presenting blood glucose levels were below those typically expected for diabetic ketoacidosis (often less than 250 mg/dL). Signs and symptoms at presentation were consistent with dehydration and severe metabolic acidosis and included nausea, vomiting, abdominal pain, generalized malaise, and shortness of breath. In some but not all cases, factors predisposing to ketoacidosis such as insulin dose reduction, acute febrile illness, reduced caloric intake, surgery, pancreatic disorders suggesting insulin deficiency (e.g., T1D, history of pancreatitis, or pancreatic surgery), and alcohol abuse were identified.

Before initiating study interventions, factors in the participant history that may predispose to ketoacidosis including pancreatic insulin deficiency from any cause, caloric restriction, and alcohol abuse will be considered.

For participants who undergo scheduled surgery, temporary discontinuation of study drug for at least 3 days prior to surgery will be considered.

Monitoring for ketoacidosis and temporary discontinuation of study interventions in other clinical situations known to predispose to ketoacidosis (e.g., prolonged fasting due to acute illness or post-surgery) will be considered. Risk factors for ketoacidosis will be resolved prior to restarting study intervention.

Participants will be educated on the signs and symptoms of ketoacidosis and instructed to discontinue study interventions and seek medical attention immediately if signs and symptoms occur.

7. Discontinuation of Study Intervention and Participant Discontinuation/Withdrawal

7.1 Discontinuation of Study Intervention

7.1.1 Permanent Discontinuation of Study Intervention

In rare instances, it may be necessary for a participant to permanently discontinue study intervention.

Participants must be withdrawn from the study intervention for the following reasons:

- If the participant experiences unacceptable toxicities to any study intervention:
  - Ketoacidosis (see Section 6.8.6)
  - Necrotizing fasciitis of the perineum (Fournier’s gangrene).
- At the specific request of the sponsor and in liaison with the investigator (e.g., obvious noncompliance, safety concerns).

For one single participant, permanent discontinuation of study intervention means that the 2 study interventions are discontinued (finerenone tablet and empagliflozin, or finerenone tablet and empagliflozin placebo, or empagliflozin and finerenone placebo).

Any participant deciding to discontinue study interventions will always be asked about the reason(s) and the presence of any AEs. The reason for premature discontinuation of the study interventions should be documented in the source documentation and captured in the eCRF.

Even participants who have stopped taking study interventions are expected to attend all the protocol specified study visits and will be encouraged to perform all assessments as stipulated in the visit schedule (especially Visit 6).

If it is not possible for a participant who has permanently discontinued study intervention to attend any visit(s) in person, the site staff will keep in touch with him/her by means of phone or virtual contact to the participant himself/herself, or to a person pre-designated by the participant, in accordance with the participant’s study visit schedule. Data will continue to be collected about his/her health status, including information on adverse events. This information may be provided either by the participant himself/herself, his/her general practitioner, or a family relative (if allowed in the respective country). Data, such as information on survival and potential protocol specified endpoints, might be also collected from a healthcare provider, from public or medical records, or other sources as available according to local guidelines and as allowed by local regulations. These data will be collected until the study is concluded, even if the participant no longer attends study visits in person, unless he/she withdrew consent and did not agree to release further information.

See the SoA (Section 1.3) for data to be collected at the time of discontinuation of study intervention and follow-up and for any further evaluations that need to be completed.

7.1.2 Temporary Discontinuation

Interruption of study intervention is permitted at any time during the study for the following reasons:

- Temporary interruption of study interventions may be considered in participants thought to be at risk of volume depletion/hypotension, such as participants with an acute medical illness potentially causing volume depletion because of inadequate fluid intake or

fluid/blood loss (e.g., gastroenteritis, gastrointestinal hemorrhage), or those undergoing major surgery.

- Other safety reasons (see Section 6.5 for guidance).
- Temporary discontinuation of study intervention for an incurrent illness, at the discretion of the investigator
- In the event of a trial-continuity issue (e.g., caused by a pandemic), the sponsor may provide additional guidance in study-specific communication.

For one single participant, interruption of study intervention means that the 2 study interventions are interrupted (finerenone tablet and empagliflozin, or finerenone tablet and empagliflozin placebo, or empagliflozin and finerenone placebo).

Upon temporary interruption of the study intervention due to hyperkalemia, eGFR decrease, (S)AE, intolerability, or any other reason, intake should be resumed as soon as medically acceptable at the discretion of the investigator. There is no defined maximum time limit for temporary interruption. In all cases, the reason for study intervention interruption must be recorded in the eCRF and the participant's medical records.

If the study intervention is interrupted for more than 7 days, the re-start should be performed at the 10-mg dose and the investigator should schedule a 4-week safety visit (±7 days) in order to monitor K+ levels and renal function (see Table 6-1). If a regular visit will be scheduled to take place 4 weeks ±7 days after up-titration or re-start, the monitoring of K+ and renal function is assured, and no 4-week safety visit has to be performed in addition.

7.2 Participant Discontinuation/Withdrawal from the Study

- A participant may withdraw from the study at any time at his/her own request or may be withdrawn at any time at the discretion of the investigator for safety, behavioral, or compliance reasons. This is expected to be uncommon.
- At the time of discontinuing from the study, if possible, an ED visit should be conducted, as shown in the SoA (Section 1.3). See SoA for data to be collected at the time of study discontinuation and follow-up and for any further evaluations that need to be completed.
- The participant will be permanently discontinued both from the study interventions and from the study at that time.
- If the participant withdraws consent for disclosure of future information, the sponsor may retain and continue to use any data collected before such a withdrawal of consent.

7.3 Lost to Follow-Up

A participant will be considered lost to follow-up if he/she repeatedly fails to return for scheduled visits and is unable to be contacted by the study site.

The following actions must be taken if a participant fails to return to the clinic for a required study visit:

- The site must attempt to contact the participant and reschedule the missed visit as soon as possible and counsel the participant on the importance of maintaining the assigned visit schedule and ascertain whether or not the participant wishes to and/or should continue in the study.

- Before a participant is deemed lost to follow-up, the investigator or designee must make every effort to regain contact with the participant (where possible, 3 telephone calls and, if necessary, a certified letter to the participant's last known mailing address or local equivalent methods). These contact attempts should be documented in the participant's medical record.
- Should the participant continue to be unreachable, he/she will be considered to have withdrawn from the study.
- Site personnel, or an independent third party, will attempt to collect the vital status of the participant within legal and ethical boundaries for all participants randomized, including those who did not get study intervention. Public sources may be searched for vital status information. If vital status is determined as deceased, this will be documented, and the participant will not be considered lost to follow-up. Sponsor personnel will not be involved in any attempts to collect vital status information.

Discontinuation of specific sites or of the study as a whole are handled as part of Appendix 1.

8. Study Assessments and Procedures

- Study procedures and their timing are summarized in the SoA (Section 1.3). Protocol waivers or exemptions are not allowed.
- Immediate safety concerns should be discussed with the sponsor immediately, upon occurrence or awareness, to determine if the participant should continue or discontinue study intervention.
- Adherence to the study design requirements, including those specified in the SoA, is essential and required for study conduct.
- All screening evaluations must be completed and reviewed to confirm that potential participants meet all eligibility criteria. The investigator will maintain a screening log to record details of all participants screened and to confirm eligibility or record reasons for screening failure, as applicable.
- Procedures conducted as part of the participant's routine clinical management (e.g., blood count) and obtained before signing of the ICF may be utilized for screening or baseline purposes provided the procedures met the protocol specified criteria and were performed within the time frame defined in the SoA.
- Laboratory results that could unblind the study will not be reported to investigative sites or other blinded personnel until the study has been unblinded.
- Repeat or unscheduled samples may be taken for safety reasons or for technical issues with the samples.
- In the event of a significant trial-continuity issue (e.g., caused by a pandemic), alternate strategies for participant visits, assessments, medication distribution and monitoring may be implemented by the sponsor or the investigator, as per local health authority/ethics requirements.

8.1 Efficacy Assessment

Planned time points for efficacy assessment are provided in the SoA (Section 1.3).

UACR

The UACR (mg of albumin per gram of creatinine) will be measured in the first morning void urine samples collected at the participant's home. For the screening eligibility, samples will be collected on 3 consecutive days, while only 2 samples will be collected for the following visits. Central laboratory will be used for all time points.

The participant will be provided with the necessary urine sample collection kit after signing the ICF. For collection details, please refer to the Central Laboratory Manual. At visits other than screeningvisit, the 2 consecutive samples can be collected ±7 days from the visit date.

If one of the collection samples is missing, the UACR for a time point will be calculated based on the available results.

Samples collected for UACR determination should not be used for assessment of safety parameters and pregnancy test.

UACR will be assessed at all study visits.

Sites will also have the option of a pre-screening visit to examine UACR using local laboratory (one sample, preferably morning void). This UACR value is not valid to determine eligibility and is only to be used to assess whether the participant may progress to a formal screening visit. Participants will be required to provide informed consent for this UACR test. In the exceptional case where no previous evidence of albuminuria is documented in the participant's medical records, the pre-screening value can be used instead. In such situations, the screening visit can only take place at least 3 months after pre-screening.

8.2 Safety Assessments

Safety will be assessed by monitoring and recording all AEs and SAEs, cardiac, hematologic, and blood chemistry parameters, vital signs, and any abnormal findings observed during the performance of physical examinations.

Planned time points for all safety assessments are provided in the SoA (see Section 1.3).

8.2.1 Vital Signs

Vital signs will be performed at the study site, at the times indicated in SoA (see Section 1.3):

- Pulse rate and BP will be assessed at all visits.
- BP and pulse measurements will be assessed in a sitting position, with a completely automated device. Manual techniques will be used only if an automated device is not available.
- BP and pulse measurements should be preceded by at least 5 minutes of rest for the participant in a quiet setting without distractions (e.g., television, cell phones).
- Vital signs (to be taken before blood collection for laboratory tests) will consist of 1 pulse and 3 BP measurements (3 consecutive BP readings will be recorded at intervals of at least 1 minute). The average of the 3 BP readings will be recorded.

8.2.1.1 Ambulatory Blood Pressure Monitoring – Only in Part A

In addition, participants enrolled in Part A will be equipped for 24 hours with an ABPM device 1 hour before the first study intervention intake, at Day 1. The name and address for the ABPM service provider can be found in the documentation supplied by the vendor.

ABPM starts during the visit and finishes approximately 24 hours later on the following day.

The 24-hour profiles will be recorded at the following intervals:

- At 30-min intervals from 06:00 to <22:00 (daytime).
- At 60-min intervals from 22:00 to <06:00 (night time).

During the recording of 24-hour ABPM, participants should refrain from unusual physical exercise.

ABPM is to be performed with a non-invasive oscillometric ambulatory BP monitor used for multiple measurements of brachial BP over an extended period of time. Once the monitor is returned to the site after the 24-hour recording, ABPM data will be uploaded to the ABPM supplier platform by the study sites. For a detailed description of the ABPM measurement schedule and procedures refer to the Manual of Procedures.

8.2.2 Physical Examinations

Physical examinations will be performed at the times indicated in SoA (see Section 1.3):

- A complete physical examination will be included, at a minimum, assessments of the CV, respiratory, gastrointestinal, and neurological systems. Weight (to the nearest 0.1 kg in indoor clothing without shoes) will also be measured and recorded. Height will only be measured and recorded at the screening visit.
- Investigators should pay special attention to clinical signs related to previous serious illnesses.

8.2.3 Electrocardiograms

12-lead ECGs will be obtained as outlined in the SoA (see Section 1.3):

- Single 12-lead ECG will be performed locally using an ECG machine that automatically calculates the heart rate and measures PR, QRS, QT, and QT corrected intervals.
- If there is any clinical indication, unscheduled 12-lead ECGs can be performed at any point during the study.
- In addition, an ECG should be obtained within 72 hours (see Section 6.5.1) if a participant has a K+ >6.5 mmol/L.
- The interpretation of the tracing must be made locally by a qualified physician.
- The date of the recording must be documented on the ECG section of the eCRF. Each ECG tracing should be labeled with the study number, participant number, and date and kept in the source documents at the study site.
- Clinically significant abnormalities should be reported as AEs or outcome event as appropriate (e.g., new onset of atrial fibrillation) when not already reported in the medical history or in one of the previous recordings and in the event of worsening of previous findings.

8.2.4 Laboratory Assessments

- See Appendix 2 for the list of clinical laboratory tests to be performed and see the SoA (Section 1.3) for the timing and frequency.
- Laboratory assessments will be performed for both efficacy and safety purposes. Per the SoA (see Section 1.3), UACR, eGFR, K+ as well as clinical chemistry, hematology, and urine analysis, will be assessed by the central laboratory (except optional pre-screening

UACR which will be assessed at a local laboratory). K+ and eGFR will also be analyzed by a local laboratory and these assessments will be used for up-/down-titration and monitoring further to dose modification of finerenone as per SoA (see Section 1.3); in exceptional cases where local laboratory K+ and eGFR results are not available, central laboratory values can be used. **These samples may be obtained up to 72 hours before a scheduled visit in order to be available for the visit.**

- The investigator must review the laboratory report, document this review, and record any clinically significant changes occurring during the study as an AE. The laboratory reports must be filed with the source documents.
- Abnormal laboratory findings associated with the underlying disease are not considered clinically significant unless judged by the investigator to be more severe than expected for the participant's condition.
- All laboratory tests with values considered clinically significantly abnormal during participation in the study or within 72 hours after the last dose of study intervention should be repeated until the values return to normal or baseline or are no longer considered clinically significant by the investigator or the medical monitor.
  - If clinically significant values do not return to normal/baseline within a period judged reasonable by the investigator, the etiology should be identified, and the sponsor notified.
  - All protocol-required laboratory tests, as defined in Appendix 2, must be conducted in accordance with the laboratory manual and the SoA.
  - If laboratory values from non-protocol specified laboratory tests performed at the institution's local laboratory require a change in participant management or are considered clinically significant by the investigator (e.g., SAE or AE or dose modification), then the results must be recorded in the eCRF.
- The steps for the review and processing of the local laboratory data will be documented within the Local Laboratory Data Handling Plan. This plan will define the processes for obtaining and using laboratory data within the clinical database to ensure the delivery of accurate laboratory data.

8.2.5 Other Safety Assessments

8.2.5.1 eGFR

eGFR will be automatically calculated by the central laboratory by using the CKD-EPI creatinine equation (Levey et al. 2009). Estimate GFR values calculated with this formula (eGFRcr) will be used for screening/eligibility and primary data analysis.

A new CKD-EPI creatinine- and cystatin C-based equation will also be used to calculate eGFR without the race factor (Inker et al. 2021). Estimate GFR values calculated with this formula (eGFRcr-cys) will be used for sensitivity analysis.

For local laboratory values, eGFR will be calculated in the eCRF by using the corresponding CKD-EPI formula.

eGFR will be assessed at all study visits.

8.2.5.2 Monitoring of Potassium

Hyperkalemia is a frequent event in participants with CKD progressing to ESKD and one of the main risks identified from previous studies with MRAs including finerenone. K+ will be monitored closely during this study, with assessments of K+ scheduled at all visits (see Section 1.3).

During treatment with study interventions, all assessments of K+, including re-tests, will be performed at both local and central laboratories. Due to sample handling and extended transportation time to the central laboratory, falsely elevated K+ values in centrally (but not locally) analyzed samples have been observed in previous studies with finerenone. Since the value of K+ in the local sample is usually not affected by this and results from the local laboratory are available earlier, local samples will be taken not only for safety purposes but also for dose adjustments. In exceptional cases where local laboratory results are not available, central laboratory values can be used.

Re-checking of K+ values must be done at the latest within 72 hours of the investigator being aware of results that need confirmation. The investigator can perform a retest at any time if he/she considers it necessary to confirm the K+ concentration of the local or the central sample.

K+ values should be recorded using a single decimal point (e.g., 4.5 mmol/L).

8.2.5.2.1 Moderate or Severe Hyperkalemia

Moderate hyperkalemia will be defined as K+ ≥5.5 to ≤6.0 mmol/L and severe hyperkalemia will be defined as K+ >6.0 mmol/L).

8.2.5.3 Acute Kidney Injury

AKI is defined as any of the following:

- An increase in serum creatinine by greater than or equal to 0.3 mg/dL within 48 hours; or
- An increase in serum creatinine by greater than or equal to 1.5 times baseline, which is known or presumed to have occurred within the prior 7 days; or
- A urine volume less than 0.5 ml/kg/h for 6 hours (KDIGO 2012).

8.2.5.4 Severe Hypoglycemia

Severe hypoglycemia will be defined as glucose level of <3.0 mmol/L (<54 mg/dL) is sufficiently low to indicate serious, clinically important hypoglycemia. In addition, severe hypoglycemia, as defined by the ADA denotes severe cognitive impairment requiring external assistance for recovery (Agiostratidou et al. 2017). It should be documented in the eCRF as an AE.

8.2.5.5 Symptomatic Hypotension (Including Volume Depletion)

Occurrence of symptomatic hypotensive and syncope events will be assessed at all study visits and should be documented as AEs.

8.2.5.6 Genital Mycotic Events

Occurrence of urinary tract infection events will be assessed at all study visits and should be documented as AEs.

8.2.5.7 Ketoacidosis Events
Occurrence of ketoacidosis events will be assessed at all study visits and should be documented as AEs.

8.2.5.8 Necrotizing Fasciitis of the Perineum (Fournier's Gangrene) Events
Occurrence of necrotizing fasciitis of the perineum events will be assessed at all study visits and should be documented as AEs.

8.2.5.9 Urosepsis and Pyelonephritis Events
Occurrence of urosepsis and pyelonephritis events will be assessed at all study visits and should be documented as AEs.

8.2.6 Pregnancy Testing

Serum pregnancy test will be performed for all female participants of childbearing potential at timepoints indicated in the SoA (see Section 1.3).

8.2.7 Suicidal Ideation and Behavior Risk Monitoring

Not applicable for this study.

8.3 Adverse Events (AEs), Serious Adverse Events (SAEs) and Other Safety Reporting

The definitions of AEs and SAEs can be found in Appendix 4.

AEs will be reported by the participant.

The investigator and any qualified designees are responsible for detecting, documenting, and recording events that meet the definition of an AE or SAE and remain responsible for following up all AEs that are serious, considered related to the study intervention, or that caused the participant to discontinue the study intervention or the study (see Section 7).

The method of recording, evaluating, and assessing causality of AEs and SAEs and the procedures for completing and transmitting SAE reports are provided in Appendix 4.

8.3.1 Time Period and Frequency for Collecting AE and SAE Information

AEs and SAEs will be collected from the start of study intervention until the last follow-up visit at the time points specified in the SoA (see Section 1.3). AEs and SAEs which are related to protocol-required study procedures (e.g., adjustment of therapy to comply with inclusion or exclusion criteria of the study) will be recorded as AEs or SAEs from the signing of the ICF.

Any medical occurrences/conditions that begin in the period between signing ICF and the start of study intervention, and which are not related to a protocol-required study procedure, will be recorded on the medical history/current medical conditions, not as AEs.

All SAEs will be recorded and reported to the sponsor or designee immediately and under no circumstance should this exceed 24 hours of learning of the event, as indicated in Appendix 4. The investigator will submit any updated SAE data to the sponsor immediately and not longer than 24 hours of it being available.

Investigators are not obligated to actively seek information on AEs or SAEs after conclusion of the study participation. However, if the investigator learns of any SAE, including a death, at any time after a participant has been discharged from the study, and he/she considers the

event to be reasonably related to the study intervention or study participation, the investigator must promptly notify the sponsor.

8.3.2 Method of Detecting AEs and SAEs

Care will be taken not to introduce bias when detecting AEs and/or SAEs. Open-ended and non-leading verbal questioning of the participant is the preferred method to inquire about AE occurrences.

8.3.3 Follow-Up of AEs and SAEs

After the initial AE/SAE report, the investigator is required to proactively follow each participant at subsequent visits/contacts. All SAEs, and AEs of special interest (AESIs; as defined in Section 8.3.8), will be followed until resolution, stabilization, the event is otherwise explained, or the participant is lost to follow-up (as defined in Section 7.3). Further information on follow-up procedures is provided in Appendix 4.

8.3.4 Regulatory Reporting Requirements for SAEs

- Prompt notification by the investigator to the sponsor of an SAE is essential so that legal obligations and ethical responsibilities toward the safety of participants and the safety of a study intervention under clinical investigation are met.
- The sponsor has a legal responsibility to notify both the local regulatory authority and other regulatory agencies about the safety of a study intervention under clinical investigation. The sponsor will comply with country-specific regulatory requirements relating to safety reporting to the regulatory authority, Institutional Review Boards (IRB)/Independent Ethics Committees (IEC), and investigators. For details on relevant country-specific requirements, please refer to Sections 10.10.1.1 for Japan and 10.10.2 for India.
- An investigator who receives an investigator safety report describing an SAE or other specific safety information (e.g., summary or listing of SAEs) from the sponsor will review and then file it along with the IB and package insert] and will notify the IRB/IEC, if appropriate according to local requirements.
- Investigator safety reports will be prepared for SUSAR according to local regulatory requirements and sponsor policy and forwarded to investigators as necessary.

For disease-related events (DREs) excluded from SAE reporting, see Section 8.3.7.

8.3.5 Pregnancy

- Details of all pregnancies in female participants will be collected after the start of study intervention and until 8 weeks after last study intervention dose.
- If a pregnancy is reported, the investigator will record pregnancy information on the appropriate form and submit it to the sponsor immediately and not longer than 24 hours of learning of the female participant pregnancy.
- While pregnancy itself is not considered to be an AE or SAE, any pregnancy complication or elective termination of a pregnancy for medical reasons will be reported as an AE or SAE.
- Abnormal pregnancy outcomes (e.g., spontaneous abortion, fetal death, stillbirth, congenital anomalies, ectopic pregnancy) are considered SAEs, and will be reported as such.

- The participant will be followed to determine the outcome of the pregnancy. The investigator will collect follow-up information on the participant and the neonate, and the information will be forwarded to the sponsor.
- Any post-study pregnancy-related SAE considered reasonably related to the study intervention by the investigator will be reported to the sponsor as described in Section 8.3.4. While the investigator is not obligated to actively seek this information in former study participants, he or she may learn of an SAE through spontaneous reporting.
- Any female participant who becomes pregnant while participating in the study will discontinue study intervention and be withdrawn from the study.

8.3.6 Cardiovascular and Death Events

Death could be classified into the following categories:

- CV death
- Non-CV death.

Classification of deaths will be based on investigator’s judgment.

All deaths will be considered as CV deaths unless otherwise classified by the MedDRA preferred term. All unwitnessed deaths will be classified as CV death.

See Appendix 5 for definitions of each category and Section 8.3.7 for DRE.

8.3.7 Disease-Related Events and/or Disease-Related Outcomes Not Qualifying for Expedited Reporting as AE or SAE

The following DREs are common in participants with CKD in T2D and can be serious/life-threatening:

- Kidney failure defined as:
  - ESRD: Initiation of chronic dialysis (hemo- or peritoneal dialysis) for at least 30 days or renal transplantation.
  - Decrease of eGFR to less than 15 mL/min/1.73m², confirmed by at least one additional standardized measurement at least 4 weeks after the initial measurement.
- Renal death (see Appendix 5 for definition)
- Chronic sustained decrease in eGFR (see Appendix 3 for definition)
- CV death (see Appendix 5 for definition)
- Non-fatal stroke (see Appendix 3 for definition)
- Non-fatal MI (see Appendix 3 for definition)
- Hospitalization for HF (see Appendix 3 for definition)
- New onset of HF (see Appendix 3 for definition).

Because these events are typically associated with the disease under study, they will not be reported according to the standard process for expedited reporting of SAEs even though the event may meet the definition of an SAE. These events will be recorded immediately of investigator’s awareness. These DREs will be monitored by the DMC on a regular basis (see Appendix 1). Please refer to Appendix 10.10 for country specificities.

However, if either of the following conditions applies, then the event must be recorded and reported as an AE/SAE (instead of a DRE):

- The event is, in the investigator's opinion, of greater intensity, frequency, or duration than expected for the individual participant.

OR

- The investigator considers that there is a reasonable possibility that the event was related to study intervention.

8.3.8 Adverse Events of Special Interest

To further characterize the safety of finerenone in combination with empagliflozin, symptomatic hypotension as well as AKI will be collected as AESIs and must be reported to the sponsor.

There is no need for expedited reporting unless it meets the criteria for an SAE as defined in Appendix 4; however, these events will need to be monitored and reviewed, and documented timely and accurately both in the source data and on the eCRF.

8.4 Pharmacokinetics

Collection of blood samples for pharmacokinetics of finerenone and empagliflozin will be performed for all participants at the time points indicated in the SoA (see Section 1.3). These samples will not be analyzed directly but may be analyzed in case of specific questions from the DMC, SC, or sponsor.

For the investigation of systemic exposure to finerenone and empagliflozin and their relationship with treatment effects, the plasma concentrations of finerenone and empagliflozin will be determined at different time points using a sparse sampling approach in all participants. The plasma concentration versus time data collected will be evaluated descriptively, separated by dose and visit. Plots will be prepared of all individual plasma concentrations versus actual relative study times (time of sample collection after time of study intervention administration).

At Visits 3 and 6, trough (i.e. pre-dose) samples for the determination of finerenone and empagliflozin plasma concentrations will be drawn before intake of study interventions. At these visits, study intervention will be administered at the study site by study personnel and the exact time of study intervention intake on the day before the visit and on the day of the visit and the exact sampling time will be recorded in the eCRF. The study personnel should contact the participant prior to Visits 3 and 6 to remind her/him not to take the study interventions as usual in the morning at home and to remind her/him to properly document time of intake.

At Visits 4, 5, and ED (if applicable), blood samples for the determination of finerenone and empagliflozin plasma concentrations will be drawn during the visit 1.5 to 10 hours after study intervention intake at home. The participants should be advised to take their study interventions as usual in the morning at home and recall the time of study intervention intake or note the time of study intervention intake on the contact card. The exact time of study intervention intake and the exact sampling times will be recorded in the eCRF.

Note: At all visits mentioned above, samples should be taken even if the study interventions were not taken as indicated. In such cases, particular attention will be paid to properly document the actual time of study intervention intake before sampling.

Pharmacokinetics and exposure-response analysis may be performed using population approaches (popPK and popPK/PD, e.g., by non-linear mixed effect modeling). Such evaluations will be described in a separate analysis plan and will be reported separately. Such evaluations may be started prior to database lock. If this is applicable, appropriate measures will be taken to maintain blinding of the study team, e.g., data will be stored separately, and members of the study team will neither have access to the randomization list nor to individual data.

Details about the collection, processing, storage and shipment of samples will be provided separately (e.g., sample handling sheets or laboratory manual).

8.5 Genetics and/or Pharmacogenomics

Genetics and pharmacogenomics are not evaluated in this study.

8.6 Biomarkers

Biomarkers may be evaluated in samples collected before, during and after treatment in order to determine the impact of study intervention. These biomarkers may fall into the following categories:

- Biomarkers related to the mode-of-action of the study intervention and/or functional markers of the cardiovascular system: e.g., vasoactive agents (aldosterone, plasma renin activity etc.), NT-proBNP/B-type natriuretic peptide (BNP), and sodium.
- OMICS/Multiplex Analysis: semi-targeted/untargeted omics analysis (e.g., metabolomics by mass spectrometry), OLINK Proximity Extension Assays (e.g., “Explore” Panels) may identify ‘*de novo*’ PD biomarkers, biomarkers which support the understanding of the mode-of-action and/or biomarkers which may indicate disease progression.
- Further biomarkers related to the mode-of-action or the safety of study intervention and similar drugs may be examined. The same applies to further biomarkers deemed relevant to kidney and (cardio)vascular diseases and associated health problems. These investigations may include e.g., diagnostic, safety, PD, monitoring, or potentially predictive biomarkers.

Timing of Collection

The planned time points of sample collection are provided in the SoA in Section 1.3. If deemed necessary, the sampling time points or frequency according to the SoA may be adjusted. Biomarker samples should be collected after the participant has been in the supine position for at least 30 minutes.

Specimen Types

The following sample types will be collected for biomarker analysis:

- Blood (plasma/serum)
- Urine

The exact specimen type for a particular biomarker analysis (e.g., serum or plasma) will be provided in separate documents (e.g., sample handling sheets or lab manual).

Sample Handling and Storage

Details on the collection, processing, shipment, and storage of samples will also be provided in separate documents (e.g., sample handling sheets or lab manual). Samples may be stored for a maximum of 15 years (or according to local regulations) following the end of the study at a facility selected by the sponsor to enable further analyses.

Reporting

Biomarker investigations may be reported separately (e.g., in a biomarker evaluation report) except, for example, plasma renin activity, aldosterone, urinary sodium, and biomarkers described in Section 9 or the statistical analysis plan (SAP).

8.7 Immunogenicity Assessments

Not applicable for this study.

8.8 Health Economics

Health Economics/Medical Resource Utilization and Health Economics parameters are not evaluated in this study.

9. Statistical Considerations

9.1 Statistical Hypotheses

The primary analysis population for analyses of the primary endpoints will be the full analysis set (FAS). Define μ_i as the relative change (ratio) from baseline to 180 days in UACR for treatment group i, where I = Combi (finerenone and empagliflozin), Emp (empagliflozin), Fin (finerenone).

The multiple primary endpoints are:

- Relative change in UACR from baseline at 180 days in combination (finerenone and empagliflozin) versus empagliflozin alone
- Relative change in UACR from baseline at 180 days in combination (finerenone and empagliflozin) versus finerenone alone.

In order to evaluate whether the combination of finerenone and empagliflozin is superior in reducing UACR than either empagliflozin or finerenone alone, we will analyze this using a repeated measures mixed model. However, to be conservative in our estimations we will apply a 2-sided 2-sample t-test of equal variance at an overall two-sided significance level of alpha=0.05. To adjust for the multiple testing of two hypotheses, the Bonferoni-Holm method will be applied. The 2 two-sided p-values are first ordered increasingly. If the lower p-value $P_i<0.025$, the corresponding null hypothesis can be rejected. The p-value Pj of the other test can then be compared with 0.05. If Pj<0.05, the second null hypothesis can also be rejected.

The primary hypotheses to be tested for the primary endpoints are provided below:

- $H0_{P1}$: $\mu_{Comb} - \mu_{Emp} = 0$
- Ha_{P1}: $\mu_{Comb} - \mu_{Emp} \neq 0$
- $H0_{P2}$: $\mu_{Comb} - \mu_{Fin} = 0$
- Ha_{P2}: $\mu_{Comb} - \mu_{Fin} \neq 0$

With two-sided overall significance level = 0.05 (0.025 for the initial hypothesis).

9.2 Sample Size Determination

Study Design and Objective

The objective of the study will be to investigate the efficacy and safety of combination of finerenone and SGLT2i versus the individual components. This will be a 3-arm study comparing combination therapy and finerenone alone and combination therapy and SGLT2i alone. The primary analysis method will be a repeated measures mixed model. However, the following calculation is based on using a two-sample t-test. Justification for this can be found in the fact that the t-test offers conservative estimates compared to the mixed model, as it only describes the final visit and adding in the information from all the visits in the study should increase the power.

The primary summary variable will be the ratio to baseline of UACR after 6 months. The objective of the study is to test the superiority of combination compared to SGLT2i and/or finerenone by applying a 2-sided two-sample t-test of equal variance at an overall two-sided significance level of alpha=0.05. To adjust for the multiple testing of two hypotheses, the Bonferoni-Holm method will be applied. The 2 two-sided p-values are first ordered increasingly. If the lower p-value P1<0.025, the corresponding null hypothesis can be rejected. The p-value P2 of the other test can then be compared with 0.05. If P2<0.05, the second null hypothesis can also be rejected.

All participants are planned to be treated for 6 months with either combination therapy or finerenone alone or SGLT2i alone.

Assumptions about UACR

UACR will be analyzed assuming a lognormal distribution. Assumptions for the UACR ratios to baseline after 6 months for finerenone are based on the data of the FIDELIO-DKD study. Assumptions for SGLT2i are based on the EMPA-REG outcome trial.

Effect of SGLT2i

It is estimated that the effect of the SGLT2i on UACR at 6 months will be approximately 30% (Wang et al. 2016; Bae et al. 2019; Cherney et al. 2016; Cherney et al. 2017; DeFronzo et al. 2015; Ridderstrale et al. 2014; Softeland et al. 2017), therefore, a mean ratio to baseline of 0.7 of UACR at 180 days is reasonable.

Effect of Finerenone

In the FIDELIO-DKD study the UACR ratio to baseline after 4 months was 0.646 and 0.573 after 12 months in the finerenone group. Using linear interpolation we were able to estimate of the value at 6 months: (0.573/-0.646)/(12-4)*(6-4)+0.646 = 0.628. The value of 0.628 for the effect of finerenone on UACR at 6 months will be investigated for the power calculations.

Effect of Combination Therapy

The combination therapy aims to be powered to detect a 20% further reduction in UACR to the monotherapy (see Section 2.1). As such, when testing the combination versus SGLT2i alone, it is intended to detect a mean ratio to baseline of 0.56 (further 20% reduction from 0.7). For combination versus finerenone alone, it is intended to detect a mean ratio to baseline of 0.5024 (further 20% reduction from 0.628).

Standard Deviation

In the FIDELIO-DKD study the standard deviation of the difference between the log-transformed UACR values after 4 months and at baseline was 0.77 in the finerenone. The data from another finerenone trial (ARTS-DN) showed a SD of 0.7 for the effect of finerenone on UACR after 3 months. Therefore, based on the FIDELIO-DKD data, a standard deviation of 0.77 is reasonable.

Sample Size

When testing the combination therapy versus finerenone alone, group sample sizes of 226 and 226 achieve 80% power to reject the null hypothesis of equal means when the log-transformed population mean difference is $\ln(\mu 1) - \ln(\mu 2) = \ln (0.502 / 0.628) = -0.224$, with a standard deviation for both groups of 0.77 and with a significance level (alpha) of 0.025 using a 2-sided 2-sample -equal variance t-test.

When testing the combination therapy versus SGLT2i alone group sample sizes of 228 and 228 achieve 80% power to reject the null hypothesis of equal means when the log-transformed population mean difference is $\ln(\mu 1) - \ln(\mu 2) = \ln(0.56 / 0.7) = -0.223$, with a standard deviation for both groups of 0.77 and with a significance level (alpha) of 0.025 using a 2-sided 2-sample -equal variance t-test.

There will be the same number of participants in each group, so for the combi therapy versus finerenone alone the power will be boosted to approximately 81% due to the extra participants included. Therefore, group sample sizes of 228, 228, and 228 will be sufficient.

Assuming a 15% drop out rate an extra 123 participants are required takes the total sample size to 807. Therefore, group sample sizes of 269, 269, and 269 will be sufficient to detect a 20% further reduction in UACR in the combination arm versus empagliflozin or finerenone.

9.3 Analysis Sets

For the purposes of analysis, the following analysis sets are defined:

Table 9-1: Analysis Sets

Participant Analysis Set	Description
Listing Only Set (LOS)	All other participants screened who did not receive any dose of study intervention or for whom no data after beginning of treatment are available will be classified as LOS.<br>Their data will be presented in the individual participant data listings but will not be included in any statistical analysis.
Safety Analysis Set (SAF)	All randomized participants who have taken at least 1 dose of study intervention.<br>All participants will be analyzed according to the actual study intervention received. For the non-combination therapy, i.e., finerenone, or empagliflozin alone, if a participant receives both study interventions due to a bottle error, the study intervention actually received for the majority of the time in the study will be used.
Full Analysis Set (FAS)	All randomized participants.<br>All participants will be analyzed according to the planned study intervention (the intent-to-treat principle).
Per-Protocol Analysis Set (PPS)	All participants of the FAS without any important protocol deviations which would interfere with the evaluation of the efficacy data.<br>All participants will be analyzed according to the actual study intervention(s) received. For the non-combination therapy i.e., finerenone, or empagliflozin alone, if a participant receives both treatments due to a bottle error, the treatment received for the majority of the time in the study will be used.
Pharmacokinetic Analysis Set	All randomized participants receiving at least 1 dose of study intervention and have 1) at least 1 quantifiable concentration and 2) no protocol deviation that would interfere with PK data evaluation.

The stratum variable eGFR category used in the statistical analysis will be derived based on the screening eGFR assessment. This assessment will use the 2009 CKD-EPI formula (Levey et al. 2009). All participants will be analyzed according to their correct stratification category. In the event of stratification errors, the primary analysis will also be repeated based on the stratification category used in the randomization as a sensitivity analysis.

9.4 Statistical Analyses

The SAP will be finalized prior to database lock and will include a more technical and detailed description of the statistical analyses described in this section. This section is a summary of the planned statistical analyses of the most important endpoints including primary and exploratory endpoints.

The primary and exploratory endpoints are defined in Section 3.

9.4.1 General Considerations

Statistical analysis will be performed using SAS; the version used will be specified in the SAP.

A lognormal distribution is assumed for serum creatinine and UACR. For all other metric variables, a normal distribution is assumed. The distributional assumptions will be investigated and if necessary, nonparametric methods or transformation of the data will be considered.

All variables will be analyzed by descriptive statistical methods. The number of data available, mean, standard deviation (SDv), minimum, median, and maximum will be calculated for metric data. The geometric mean and SDv will be provided instead of the arithmetic mean and SDv for the variables where lognormal distributions are assumed.

Frequency tables will be generated for categorical data.

Baseline values will be defined as the last non-missing measurement before first study intervention intake (Day 1). If the last observation available prior to randomization is the measurement from the screening visit, this would be used as the baseline value. This also includes assessments from a local laboratory, in case that prior to randomization, no assessment from the central laboratory is available. Otherwise baseline will be missing. If more than one measurement was planned for a scheduled time point, for example BP measurements, the mean value of the last set of measurements per time point prior to randomization will be used as the baseline value.

Only the data provided by the central laboratory will be used for analysis, values from local laboratories will not be used in the statistical analysis and listed only.

In the event of repeated measurements for screening and the randomization visit (Day 1, baseline), the closest measurement prior to randomization will be used for analysis instead of the scheduled measurements. At all visits post-randomization, unless stated otherwise, only the values at scheduled measurements will be used for analysis.

The derived visit ‘any time post baseline’ (applicable for efficacy) will include any measurement after randomization, including unscheduled assessments. For the derived visit ‘any time on-treatment’, only assessments up to 30 days after last study intervention administration, including unscheduled assessments, will be considered (applicable for efficacy; for safety assessments within 3 days after last study intervention administration will be considered).

Comparisons will only be drawn between the combination therapy and each monotherapy. No statistical comparison between the monotherapies will be performed.

Further details on the statistical analyses will be provided in the SAP.

9.4.2 Disposition, Baseline, History, Demography and Medication

The analyses of disposition, baseline, history, and demography are described below.

9.4.3 Disposition

The number of participants screened, screen failed, enrolled, randomized, and valid for the safety analysis set (SAF), FAS, and per-protocol analysis set (PPS) will be summarized overall and by treatment group, country, and investigator. The number of participants discontinuing the treatment and follow-up epochs, together with the primary reason for discontinuation, will be presented by treatment group and overall, in separate tables. In addition, the number of participants with important protocol deviations and validity findings will be presented overall, by investigator and country for each treatment group, and in total. The frequencies of each important protocol deviation and validity finding will be presented by treatment group and in total.

9.4.3.1 Population Characteristics

Population characteristics analyses, except for participant disposition, will be performed for the FAS, if not stated otherwise.

9.4.3.2 Demography and Other Baseline Characteristics

Demography includes age, sex, race, ethnicity, region (North America, Europe, and Asia), body weight, body height, BMI, smoking history (never, former, current smoker), drugs, caffeine, and alcohol consumption. Other baseline characteristics include baseline UACR, K+, categories for K+ (≤4.5 mmol and >4.5 mmol), eGFR (calculated by CKD-EPI [Levey et al. 2009] formula), serum creatinine, HbA1c, and values for vital signs parameters (i.e., systolic BP, diastolic BP, and pulse rate).

All demographic data and baseline characteristics will be tabulated by treatment group and overall. The demographic and other baseline characteristics table will also be presented, separated by each level of the stratification factors, UACR category and eGFR category.

The non-stratified demographic and other baseline characteristics table will be repeated for all other analysis sets if they differ in sample size from the FAS.

Demographics and other baseline characteristics will be presented for the FAS separately for the participants belonging to PPS or not (only overall, not by treatment group).

9.4.3.3 Medical History

Medical history will be coded using the Medical Dictionary for Regulatory Activities (MedDRA) dictionary. Medical history will be presented for each MedDRA primary System Organ Class (SOC) and Preferred Term (PT) by treatment group and overall, in a summary table. Additional medical history terms by Standardized MedDRA Queries (SMQ) will also be presented.

9.4.3.4 Concomitant Medication

Concomitant medication will be coded using the WHO Drug Dictionary (WHO-DD). The number of participants who took at least 1 concomitant medication, the number of participants who took at least 1 medication that started and ended before administration of study intervention, and the number of participants who took at least 1 concomitant medication that started after start of study intervention will be presented by treatment group and overall using Anatomical Therapeutic Chemical (ATC) classes and subclasses. These tables will be repeated, summarizing the number of participants with medication in the Bayer drug groups of interest (including ACEi, ARBs, insulin, insulin secretagogues, metformin, beta-blocker, diuretics, K+ sparing diuretics, K+ supplements, K+ lowering agents, alpha blocking agents, calcium channel blockers, centrally acting antihypertensives and strong, unclassified, moderate, or weak CYP3A4 inhibitors, trimethoprim, trimethoprim-sulfamethoxazole, any other SGLT-2i or combined SGLT-1 and 2i). A participant will be counted only once within each ATC class/subclass or sponsor drug group, respectively.

A listing will be provided including all medication classified as a weak, moderate, or strong CYP3A4 inhibitor according to the sponsor drug groupings together with the respective classification information.

9.4.3.5 Treatment Duration, Extent of Exposure, Up-Titration Status, and Compliance

The analyses described in this section will be repeated for the SAF and PPS if they differ in sample size from the FAS.

Treatment duration (number of months with study intervention intake) will be summarized using descriptive statistics by treatment group and overall. In addition, treatment duration will

be categorized and presented with the corresponding number and percentage of participants by treatment group and overall. Further specification of the categories will be provided in the SAP.

A table will be presented with the absolute and relative frequencies of participants still in the study at each visit. Kaplan-Meier plots for '*Time to end of study intervention*' will be provided. The extent of exposure to study intervention (total amount of intake in grams) will be summarized using descriptive statistics by treatment group.

The up-titration status (yes/no), regardless of actual or sham up-titration, will be summarized with absolute and relative frequencies per treatment group for each visit as well as participants never up-titrated, up-titrated once, and up-titrated more than once. Compliance (as a percentage) will be calculated as follows:

- 100 * Number of taken tablets or capsules/Number of planned tablets or capsules.

The number of planned tablets or capsules will be calculated as follows:

- (Days from randomization to last intake of study intervention + 1) * Number of planned tablets or capsules per day.

All tablets and capsules, including the dummy placebo tablets and capsules, will be counted. For participants who withdraw prematurely from the study intervention, compliance will be calculated up to the time of last dose.

The compliance will be summarized descriptively by treatment group and overall. In addition, percent of compliance will be categorized into 3 groups, <80%, ≥80 to ≤120%, and >120%, and the categories will be summarized by treatment group and overall.

9.4.4 Primary Efficacy Endpoints

The primary efficacy endpoints are:

- Relative change from baseline in UACR at 180 days in combination (finerenone and empagliflozin) versus empagliflozin alone
- Relative change from baseline in UACR at 180 days in combination (finerenone and empagliflozin) versus finerenone alone.

In order to evaluate whether finerenone and empagliflozin is superior in reducing UACR than either empagliflozin or finerenone alone, a two-sided two-group t-test of equal means at a multiplicity adjusted (Bonferroni-Holm) significance level of 0.05/2 = 0.025 will be applied for the initial hypothesis test.

UACR during the study will be summarized descriptively by treatment group and visit including ratios to baseline. These analyses will be performed overall and separated by the stratification factors (UACR category and eGFR category).

The log-transformed ratio of UACR to baseline at each visit up to 180 days will be analyzed by a mixed model with the factors treatment group, visit, treatment by visit interaction, factors for the 2 stratification levels, UACR category and eGFR category, log-transformed baseline value as covariate nested within type of albuminuria, and log-transformed baseline value by visit interaction. Pairwise ratios between the finerenone plus empagliflozin, and the empagliflozin treatment group will be calculated and corresponding two-sided 95% CIs will be computed.

The same analysis will be performed between the finerenone plus empagliflozin, and the finerenone treatment group.

Intercurrent events shall be handled using the policies outlined in Section 3. Patients that experience kidney transplant/dialysis/death will be included in the analysis with all measures up to the date of the event. Patients that discontinue treatment will have all measures available included in the analysis.

The primary analysis of the primary efficacy variable will be repeated in the PPS as a supportive analysis. An 'on-treatment' analysis will be performed, including only events occurring while taking study drug or until 30 days after stop of study drug. This analysis will be performed in the FAS. Sensitivity analyses for the primary endpoint will be performed taking into consideration different strategies for handling the intercurrent events of kidney transplant/dialysis/death. Further details for all analyses will be provided in the statistical analysis plan.

9.4.5 Secondary Efficacy Endpoints

The secondary efficacy endpoints will be analyzed in the FAS population unless otherwise specified in SAP. Time-to-event endpoints will be analyzed using stratified log–rank test with randomization stratification factors. Hazard ratio and 95% CI will be provided using the Cox model stratified by the same factors as stated above. Detailed analysis methods and the plan for type 1 error control for exploratory endpoints will be specified in SAP.

The secondary analysis endpoints are:

- Relative change in UACR between end of treatment visit and at 30 days after end of treatment visit
- Relative change in UACR between 30 days after end of treatment visit and baseline
- Relative change in UACR category (>30%, >40%, and >50%) at 180 days.

9.4.5.1 Relative Change in UACR Category

Frequency tables will be generated for the number of participants with a relative decrease and increase in UACR of >30%, >40%, and >50% from baseline UACR. The analysis will be performed for each visit and for any time post baseline. The analysis will also be performed stratified for each level of the stratification factors (UACR category and eGFR category).

A shift table will be provided displaying the number of participants who changed from baseline to each visit. Change in albuminuria category will only be considered as shifts if they are accompanied by a UACR decrease of at least 30% from baseline to each visit.

The additional categorical UACR efficacy variables listed in Section 3 will be summarized for presence or absence of the event using logistic regression with the factor's treatment group and stratification levels (UACR category and eGFR category). Pairwise differences between the combination versus empagliflozin or combination versus finerenone group will be calculated and corresponding two-sided 95% CIs will be computed.

9.4.5.2 Subgroup Analyses

Exploratory subgroup analyses are planned for the primary efficacy variable.

This will include descriptive statistics and a statistical test for interaction.

The following subgroups will be considered for exploratory subgroup analyses:

- Region (North America, Europe, and Asia)
- eGFR category at screening and baseline (eGFR 40 to <60, 60 to 75 mL/min/1.73m^2)

- History of CVD (present, absent)
- Baseline K+ (≤4.5 versus >4.5 mmol/L)
- Systolic BP at baseline (>90 to <130, 130 to <160 mmHg).

It is anticipated that in these proposed subgroups for analysis, differences in treatment effects may be observed according to the screening or baseline characteristics defined, due in part to the differences in the risk of clinical events expected in the different subgroups.

Furthermore, subgroup analysis usually required will be performed, including the following subgroups:

- Race
- Sex
- Age group.

9.4.6 Safety Analysis

All analyses on safety data will be performed in the SAF.

The following safety variables will be assessed during the study:

- AEs
- Laboratory data
- ECG data
- Vital signs, including weight and BMI
- Further safety variables:
    - Change from baseline in eGFR at 30 days
    - eGFR decline greater than 30% at Day 30 from baseline
    - Change from baseline in eGFR at 180 days
    - Change from baseline in eGFR at 210 days
    - Proportion of participants with AKI events
    - Total number of AKI events
    - Change from baseline in K+
    - Proportion of participants with hyperkalemia events
    - Total number of hyperkalemia events
    - Proportion of participants with moderate hyperkalemia events (K+ >5.5 to ≤6.0 mmol/L)
    - Total number of moderate hyperkalemia events (K+ >5.5 to ≤6.0 mmol/L)
    - Proportion of participants with severe hyperkalemia events (K+ >6.0 mmol/L)
    - Total number of severe hyperkalemia events (K+ >6.0 mmol/L)
    - Proportion of participants with severe hypoglycemia events
    - Total number of severe hypoglycemia events
    - Proportion of participants with symptomatic hypotension events
    - Total number of symptomatic hypotension events
    - Proportion of participants with genital mycotic events
    - Total number of genital mycotic events

- Proportion of participants with ketoacidosis
- Total number of ketoacidosis events
- Proportion of participants with necrotizing fasciitis of the perineum events
- Total number of necrotizing fasciitis of the perineum events.
- Proportion of participants with urosepsis and pyelonephritis events
- Total number of urosepsis and pyelonephritis events.

9.4.6.1 Adverse Event

AEs will be coded using the MedDRA (latest version available prior to data base freeze). A listing will be provided linking the original investigator terms and the coded terms. AEs will also be presented grouped by SMQs.

AEs that started or worsened after the first dose of study intervention up to 3 days after any temporary or permanent interruption of study intervention will be considered as treatment-emergent AEs (TEAEs).

An overall summary of all AEs and TEAEs will be generated by treatment group. The number of participants with TEAEs, post-treatment AEs occurring more than 3 days after stop of study intervention, treatment-emergent SAEs, treatment-emergent study intervention-related AEs, treatment-emergent study intervention-related SAEs, TEAEs causing permanent discontinuation of study intervention, treatment-emergent non-serious AEs, non-serious AEs, TEAEs by maximum intensity, treatment-emergent SAEs by maximum intensity, study intervention-related TEAEs by maximum intensity, TEAEs by worst outcome, and treatment-emergent SAEs by worst outcome will be summarized by treatment group using MedDRA terms grouped by primary SOC and PT.

In case of events with different intensity within a participant, the maximum reported intensity will be used. If intensity is missing, the event will be considered as severe. If the same event is reported as both unrelated and related to the study intervention within a participant, the event will be reported as related to study intervention. If the drug relationship is missing, the event will be considered as being related to the study intervention.

Separate tables summarizing TEAEs, treatment-emergent study intervention-related AEs, and SAEs that occurred in more than 5% of the participants will be provided.

Deaths, SAEs, and AEs leading to permanent study intervention discontinuation will be listed separately.

9.4.6.2 Laboratory Data

The number of participants with treatment-emergent (until 3 days after any temporary or permanent interruption of study intervention) abnormal laboratory values above or below the normal range will be tabulated by the laboratory parameter and treatment group.

Summary statistics including changes to baseline will be calculated by treatment group and visit for all quantitative laboratory parameters, e.g., for hematology, HbA1c, clinical chemistry, and urinalysis. Geometric statistics and ratios to baseline will be presented for creatinine instead of arithmetic statistics with changes from baseline. For eGFR, the relative change will be displayed in addition to the absolute change from baseline.

Summary statistics for K^+, eGFR, and serum creatinine will also be repeated by treatment group and visit separately for each level of the stratification factors (UACR category and

eGFR category). As a sensitivity analysis, the analysis on eGFR will be repeated using the updated CKD-EPI formula published in 2021 (Inker et al. 2021).

9.4.6.3 Vital Signs, Including Weight and BMI

At the corresponding visits, 3 measurements of BP will be taken in sitting position with at least a 1-minute interval between each reading. Averages of non-missing values of these 3 measurements will be calculated and used for the statistical analysis. If only 1 of the planned measurements is available, this value will be used.

Vital signs values will be summarized by treatment group and visit using descriptive statistics including absolute changes from baseline. The analysis will be repeated for systolic BP stratified by baseline systolic BP >90 to <130 mmHg, 130 to <160 mmHg, and ≥160 mmHg.

The values and the changes from baseline will be summarized by treatment group and visit using descriptive statistics for weight and BMI.

9.4.6.4 Further Safety Variables

All safety variables are listed at the start of this section. Not covered in the above sections are detailed in the below sections.

For each analysis, the percentage of participants with the respective events (non-stratified) at any time post-baseline (including unscheduled assessments) will be compared between the finerenone and empagliflozin, and the empagliflozin treatment group by applying separate explorative χ^2 tests with continuity correction. If the expected number of participants in at least 1 cell of the 2x2 contingency table is <5, Fisher's exact test will be applied instead of the χ^2 test. Estimates and 2-sided 95% CIs will be provided for each treatment group and the treatment differences. Clopper Pearson CIs will be calculated for each treatment group, while for treatment differences the exact unconditional confidence limits will be calculated. Similarly, this will be repeated for finerenone and empagliflozin, and the finerenone treatment group.

The eGFR endpoints will be analyzed in the FAS population unless otherwise specified in SAP. As a sensitivity analysis, the analysis on eGFR endpoints will be repeated using the updated CKD-EPI formula published in 2021 (Inker et al. 2021).

Time-to-event endpoints will be analyzed using stratified log-rank test with randomization stratification factors. Hazard ratio and 95% CI will be provided using the stratified Cox proportional hazards model. Detailed analysis methods and the plan for type 1 error control for exploratory endpoints will be specified in the SAP.

9.4.6.4.1 Ratio of Change from Baseline in eGFR at 30 days

The endpoint will be analyzed as described for the primary endpoint. As a sensitivity analysis, this will be repeated using the updated CKD-EPI formula published in 2021 (Inker et al. 2021).

9.4.6.4.2 eGFR Decline Greater than 30% at 30 days from Baseline

The percentage of participants with eGFR decline greater than 30% at Day 30 from baseline will be summarized by treatment group. As a sensitivity analysis, this will be repeated using the updated CKD-EPI formula published in 2021 (Inker et al. 2021).

9.4.6.4.3 Ratio of change in eGFR at 180 days and 210 days from Day 30

The endpoint will be analyzed as described for the primary endpoint. As a sensitivity analysis, this will be repeated using the updated CKD-EPI formula published in 2021 (Inker et al. 2021).

9.4.6.4.4 Proportion of Participants with Acute Kidney Injury and Number of AKI Events

The percentage of participants with and total number of events of AKI will be summarized by treatment group.

9.4.6.4.5 Proportion of Participants with Hyperkalemia and Number of Hyperkalemia Events

The percentage of participants with and total number of events of hyperkalemia ($K+ > 5.5$ mmol/L) will be summarized by treatment group.

9.4.6.4.6 Proportion of Participants with Moderate Hyperkalemia and Number of Moderate Hyperkalemia Events

The percentage of participants with and total number of events of moderate hyperkalemia ($K+ > 5.5$ mmol/L to ≤ 6.0 mmol/L) will be summarized by treatment group.

9.4.6.4.7 Proportion of Participants with Severe Hyperkalemia and Number of Severe Hyperkalemia Events

The percentage of participants with and total number of events of severe hyperkalemia ($K+ > 6.0$ mmol/L) will be summarized by treatment group.

9.4.6.4.8 Proportion of Participants with Severe Hypoglycemia Events and Number of Severe Hypoglycemia Events

The percentage of participants with and total number of events of severe hypoglycemia events will be summarized by treatment group.

9.4.6.4.9 Proportion of Participants with Symptomatic Hypotension and Number of Symptomatic Hypotension Events

The percentage of participants reporting symptomatic hypotension and syncope events and total number of symptomatic events will be summarized by treatment group.

9.4.6.4.10 Proportion of Participants with Genital Mycotic Events and Number of Genital Mycotic Events

The percentage of participants with and total number of events of genital mycotic events will be summarized by treatment group.

9.4.6.4.11 Proportion of Participants with Ketoacidosis Events and Number of Ketoacidosis Events

The percentage of participants with and total number of events of ketoacidosis will be summarized by treatment group.

9.4.6.4.12 Proportion of Participants with Necrotizing Fasciitis of the Perineum and Number of Necrotizing Fasciitis of the Perineum Events

The percentage of participants with and total number of events of necrotizing fasciitis of the perineum will be summarized by treatment group.

9.4.6.4.13 Proportion of Participants with Urosepsis and Pyelonephritis and Number of Urosepsis and Pyelonephritis Events

The percentage of participants with and total number of events of urosepsis and pyelonephritis events will be summarized by treatment group.

9.4.7 Missing Data/Dropouts

A participant who has been randomized and discontinues study participation prematurely for any reason, either from study intervention or from follow-up, is defined as a '*dropout*', even if no study intervention has been taken. Dropouts will not be replaced.

Data from participants who prematurely terminated the study will be used to the maximum extent possible.

All efforts will be made to collect complete data for all participants randomized in this study. Participants will be followed until the end of the study, and all required data will be collected, regardless of their compliance with study intervention use or visit schedule.

After randomization, study intervention discontinuation for any reason does not constitute withdrawal from the study and should not lead to the participant being withdrawn from the study. On the contrary, even participants who have stopped taking study intervention are expected to attend all the protocol specified study visits and will be encouraged to perform all assessments as stipulated in the visit schedule.

If it is not possible for a participant who has withdrawn from study intervention to attend any visit(s) in person, the site staff will keep in touch with the participant by means of phone or virtual contact to the participant himself/herself, or to a person pre-designated by the participant, in accordance with the participant's study visit schedule.

Data will continue to be collected about his/her health status, including information on adverse events. This information may be provided either by the participant himself/herself, his/her general practitioner, or a family relative (if allowed in the respective country). Data, such as information on survival and potential protocol specified endpoints, might be also collected from a healthcare provider, from public or medical records, or other sources as available according to local guidelines and as allowed by local regulations. These data will be collected until the study is concluded, even if the participant no longer attends study visits in person, unless he/she withdrew consent and did not agree to release further information.

When an event date is not known, the site investigator will be asked to provide a best estimate as to when the event occurred. Even though the exact date of an event is unknown, the investigator often has some information that would give an approximate date (e.g., the first week of a month, the fall of a year, or the middle of a particular year) or at least the date when the participant was last seen or contacted. This information can be meaningfully incorporated into the estimated date recorded, as this is likely to be closer to the true date than any produced by an uninformed computer algorithm. This estimated date should be the middle date within the period that the event is known to have occurred. If the event is known

to have occurred in the first week of a month, then the date in the middle of that week should be recorded as the estimate. If it occurred in the fall of a year, then the middle date in the fall is the appropriate estimate. If no information is known, then the date in the middle of the plausible period should be given, based on the last contact with the participant prior to the event and the date of contact when information about the event was known.

Data from participants who prematurely terminate the study will be used to the maximum extent possible. All missing or partial data will be presented in the participant data listing as they are recorded on the eCRF. Data are collected primarily through an electronic data capture system, which allows ongoing data entry and monitoring.

For those participants who withdraw consent, sensitivity analyses will be performed to assess the impact of potential informative censoring of such participants. These will include the use of different imputation rules for considering participants without an event of the primary composite endpoint as having an event or being censored.

9.5 Interim Analysis

No interim analysis is planned for this study.

10. Supporting Documentation and Operational Considerations

10.1 Appendix 1: Regulatory, Ethical, and Study Oversight Considerations

10.1.1 Regulatory and Ethical Considerations

- This study will be conducted in accordance with the protocol and with the following:
  - Consensus ethical principles derived from international guidelines including the Declaration of Helsinki and Council for International Organizations of Medical Sciences International Ethical Guidelines
  - Applicable International Council for Harmonization (ICH) GCP Guidelines
  - Applicable laws and regulations.
- The protocol, protocol amendments, ICF, IB, and other relevant documents (e.g., advertisements) must be submitted to an IRB/IEC by the investigator and reviewed and approved by the IRB/IEC before the study is initiated.
- Any amendments to the protocol will require IRB/IEC approval before implementation of changes made to the study design, except for changes necessary to eliminate an immediate hazard to study participants. Any substantial modification of the protocol will be submitted to the competent authorities as substantial amendments for approval, in accordance with ICH GCP and national and international regulations.
- Protocols and any substantial amendments to the protocol will require health authority approval prior to initiation except for changes necessary to eliminate an immediate hazard to study participants.
- The investigator will be responsible for the following:
  - Providing written summaries of the status of the study to the IRB/IEC annually or more frequently in accordance with the requirements, policies, and procedures established by the IRB/IEC
  - Notifying the IRB/IEC of SAEs or other significant safety findings as required by IRB/IEC procedures
  - Providing oversight of the conduct of the study at the site and adherence to requirements of ICH guidelines, the IRB/IEC, and all other applicable local regulations.

10.1.2 Financial Disclosure

Investigators and sub-investigators will provide the sponsor with sufficient, accurate financial information as requested to allow the sponsor to submit complete and accurate financial certification, or disclosure statements to the appropriate regulatory authorities. Investigators are responsible for providing information on financial interests during the study and for 1 year after completion of the study.

10.1.3 Informed Consent Process

- The investigator or his/her representative will explain the nature of the study to the participants or their legally authorized representative and answer all questions regarding the study.
- Participants must be informed that their participation is voluntary. Participants or their legally authorized representative will be required to sign a statement of informed consent that meets the requirements of 21 Code of Federal Reguation 50, local regulations, ICH

guidelines, Health Insurance Portability and Accountability Act requirements, where applicable, and the IRB/IEC or study center.

- The medical record must include a statement that written informed consent was obtained before the participant was enrolled in the study and the date the written consent was obtained. The authorized person obtaining the informed consent must also sign the ICF.
- Participants must be re-consented to the most current version of the ICF(s) during their participation in the study.
- A copy of the ICF(s) must be provided to the participants or their legally authorized representative.

10.1.4 Data Protection

- Participants will be assigned a unique identifier by the sponsor. Any participant records, datasets or biological samples that are transferred to the sponsor will contain the identifier only; participant names or any information which would make the participant identifiable will not be transferred.
- The participant must be informed that his/her personal study-related data will be used by the sponsor in accordance with local data protection law. The level of disclosure must also be explained to the participant who will be required to give consent for their data to be used as described in the informed consent
- The participant must be informed that his/her medical records may be examined by Clinical Quality Assurance auditors or other authorized personnel appointed by the sponsor, by appropriate IRB/IEC members, and by inspectors from regulatory authorities.

10.1.5 Committee Structure

10.1.5.1 Data Monitoring Committee

Ongoing safety monitoring during the conduct of the study will be performed by an external and independent DMC. An independent statistical analysis center (SAC) will be involved in processing unblinded safety data for the DMC. Analysis periods and procedures will be defined in an operational charter (DMC charter) filed in the study file.

The independent DMC will periodically review and assess safety data from the study for imbalances in safety outcomes in an unblinded manner. It is believed that in this way, participant safety can continue to be monitored throughout the duration of the trial, and the integrity of the study maintained. If unexpected safety issues are identified, specific amendments will be implemented based on the recommendation of the DMC.

Following data review, the DMC will provide written recommendations that will be transferred to the chairmen of the SC and the sponsor. DMC opinions and recommendations will be notified by the sponsor as soon as possible to the competent authorities and the IRBs/IECs where they qualify for expedited reporting.

10.1.5.2 Steering Committee

The SC will consist of external experts in the area of nephrology and diabetology. The SC will be blinded to the study data while the trial is ongoing. Their main responsibilities are as follows:

- Provide input to protocol-related issues and protocol amendments that may arise during the study.

- Oversee study progress and provide recommendations to the sponsor in regard to any necessary modifications that may be required in study conduct or study monitoring.
- Transmission of information to individual investigators.
- Serve as resource for scientific review of sub-studies, publications, presentations, and/or educational material as applicable.

10.1.6 Dissemination of Clinical Study Data

Result Summaries of Bayer's sponsored clinical trials in drug development phases 2, 3, and 4, and phase 1 trials in participants are provided in the Bayer Trial Finder application after marketing authorization approval in line with the position of the global pharmaceutical industry associations laid down in the "Joint Position on the Disclosure of Clinical Trial Information via Clinical Trial Registries and Databases". In addition, results of clinical drug trials will be provided on the publicly funded website www.ClinicalTrials.gov and European Union (EU) Clinical Trials Register in line with the applicable regulations.

Bayer commits to sharing upon request from qualified scientific and medical researchers, participant-level clinical trial data, study-level clinical trial data, and protocols from clinical trials in participants for medicines and indications approved in the US and EU on or after 01 JAN 2014 as necessary for conducting legitimate research.

All Bayer-sponsored clinical trials are considered for publication in the scientific literature irrespective of whether the results of the clinical trials are positive or negative.

10.1.7 Data Quality Assurance

- All participant data relating to the study will be recorded on printed or eCRF unless transmitted to the sponsor or designee electronically (e.g., laboratory data). The investigator is responsible for verifying that data entries are accurate and correct by physically or electronically signing the eCRF.
- Guidance on completion of eCRFs will be provided in the eCRF guidelines.
- The investigator must permit study-related monitoring, audits, IRB/IEC review, and regulatory agency inspections and provide direct access to source data documents.
- Quality tolerance limits (QTLs) will be predefined in the Integrated Quality Risk Management Plan to identify systematic issues that can impact participant safety and/or reliability of study results. These predefined parameters will be monitored during the study and important deviations from the QTLs and remedial actions taken will be summarized in the clinical study report.
- Monitoring details describing strategy (e.g., risk-based initiatives in operations and quality such as Risk Management and Mitigation Strategies and Analytical Risk-Based Monitoring), methods, responsibilities and requirements, including handling of noncompliance issues and monitoring techniques (central, remote, or on-site monitoring) are provided in the Monitoring Plan.
- The sponsor or designee is responsible for the data management of this study including quality checking of the data.
- The sponsor assumes accountability for actions delegated to other individuals (e.g., contract research organizations).

- Records and documents, including signed ICFs, pertaining to the conduct of this study must be retained by the investigator for 25 years after study completion unless local regulations or institutional policies require a longer retention period. No records may be destroyed during the retention period without the written approval of the sponsor. No records may be transferred to another location or party without written notification to the sponsor.

10.1.8 Source Documents

- Source documents provide evidence for the existence of the participant and substantiate the integrity of the data collected. Source documents are filed at the investigator's site.
- Data reported on the eCRF or entered in the eCRF that are transcribed from source documents must be consistent with the source documents or the discrepancies must be explained. The investigator may need to request previous medical records or transfer records, depending on the study. Also, current medical records must be available.
- Definition of what constitutes source data can be found in the Source Data Identification Form.
- The investigator must maintain accurate documentation (source data) that supports the information entered in the eCRF.
- Study monitors will perform ongoing source data verification to confirm that data entered into the eCRF by authorized site personnel are accurate, complete, and verifiable from source documents; that the safety and rights of participants are being protected; and that the study is being conducted in accordance with the currently approved protocol and any other study agreements, ICH GCP, and all applicable regulatory requirements.

10.1.9 Study and Site Start and Closure

First Act of Recruitment

The study start date is the date on which the clinical study will be open for recruitment of participants.

The first act of recruitment is the first signed ICF and will be the study start date.

Study/Site Termination

The sponsor or designee reserves the right to close the study site or terminate the study at any time for any reason at the sole discretion of the sponsor. Study sites will be closed upon study completion. A study site is considered closed when all required documents and study supplies have been collected and a study site closure visit has been performed.

The investigator may initiate study site closure at any time, provided there is reasonable cause and sufficient notice is given in advance of the intended termination.

Reasons for the early closure of a study site by the sponsor or investigator may include but are not limited to:

For study termination:

- Discontinuation of further study intervention development.

For site termination:

- Failure of the investigator to comply with the protocol, the requirements of the IRB/IEC or local health authorities, the sponsor's procedures, or GCP guidelines
- Inadequate or no recruitment (evaluated after a reasonable amount of time) of participants by the investigator
- Total number of participants included earlier than expected.

If the study is prematurely terminated or suspended, the sponsor shall promptly inform the investigators, the IECs/IRBs, the regulatory authorities, and any contract research organization(s) used in the study of the reason for termination or suspension, as specified by the applicable regulatory requirements. The investigator shall promptly inform the participant and should assure appropriate participant therapy and/or follow-up.

10.1.10 Publication Policy

- The results of this study may be published or presented at scientific meetings. If this is foreseen, the investigator agrees to submit all manuscripts or abstracts to the sponsor before submission. This allows the sponsor to protect proprietary information and to provide comments.
- The sponsor will comply with the requirements for publication of study results. In accordance with standard editorial and ethical practice, the sponsor will generally support publication of multicenter studies only in their entirety and not as individual site data. In this case, a coordinating investigator will be designated by mutual agreement.
- Authorship will be determined by mutual agreement and in line with International Committee of Medical Journal Editors authorship requirements.

10.2 Appendix 2: Clinical Laboratory Tests

- The test detailed in Table 10-1 will be performed at the times indicated in SoA (see Section 1.3).
- Both local and central laboratories will be used.
- It is important that the samples for central analysis are obtained at the same time than the samples for the local laboratory.
- Additionally, local laboratory results used to make either a study intervention decision or response evaluation must be recorded.
- Protocol-specific requirements for inclusion or exclusion of participants are detailed in Section 5 of the protocol.
- Additional tests may be performed at any time during the study as determined necessary by the investigator or required by local regulations.

Table 10-1: Protocol-Required Laboratory Tests

**Laboratory Tests**	**Parameters**			
Hematology	Platelet count	Red blood cell (RBC) indices:<br>MCV<br>MCH<br>MCHC		White blood cell (WBC) count
	RBC count			
	Hemoglobin			
	Hematocrit			
Clinical chemistry	Blood urea nitrogen (BUN)			Total and direct bilirubin
	Aspartate aminotransferase (AST)	Alanine aminotransferase (ALT)	Alkaline phosphatase (AP)	Gamma glutamyl transpeptidase (GGT)
	Lactate dehydrogenase (LDH)	Creatine kinase (CK)	Albumin	Total protein
	Creatinine	Cystatin C	eGFRcr (CKD-EPI creatinine, Levey et al. 2009) and eGFRcr-cys (CKD-EPI creatinine-cystatin C, Inker et al. 2021)	
	High density lipoprotein (HDL)	Low density lipoprotein cholesterol (LDL-C)	Total cholesterol	Triglycerides
	Potassium (K+)	Bicarbonates	Sodium	
	Glucose (fasting or not fasting)	Glycated hemoglobin (HbA1c)[1]		
Urinalysis	• Urinary albumin-to-creatinine ratio (UACR)<br>• pH, glucose, protein, blood, ketones, by dipstick<br>• Microscopic examination (if blood or protein is abnormal)			
Pregnancy testing	• Highly sensitive serum human chorionic gonadotropin (hCG) pregnancy test (as needed for women of childbearing potential)			
Postmenopausal status confirmation[2]	• Follicle stimulating hormone (FSH)			
	All study-required laboratory assessments will be performed by a central laboratory, with the exception of:<br>• optional pre-screening UACR (local laboratory)<br>• K+ (both at central and local laboratories)<br>• estimated glomerular filtration rate (both at central and local laboratories)			
NOTES:<br>[1] At Day 1.<br>[2] A high FSH level in the postmenopausal range may be used to confirm a postmenopausal state in women not using hormonal contraception or HRT. However, in the absence of 12 months of amenorrhea, confirmation with more than one FSH measurement is required.				

Investigators must document their review of each laboratory safety report.

10.2.1 Biomarkers

Biomarkers associated to the mode-of action of the study intervention and/or functional markers relevant to CKD and CV disease will be evaluated. Biomarkers may include:

- Vasoactive agents (aldosterone, plasma renin activity, etc.), NT-proBNP/BNP, and sodium.
- 'De novo' PD biomarkers identified by OMICS/Multiplex Analysis which may indicate disease progression.
- Other biomarkers deemed relevant to kidney and CV diseases.

The correlation between the measurements of the above indicators and clinical outcomes will be described.

10.2.2 Pharmacokinetics

Finerenone and empagliflozin plasma concentrations might be analyzed in case of specific questions from the DMC, Steering Committee, or sponsor.

10.3 Appendix 3: Disease-Related Event Definitions

10.3.1 Myocardial Infarction

The term MI should be used when there is evidence of myocardial necrosis in a clinical setting consistent with myocardial ischemia.

In general, the diagnosis of MI requires the combination of (Thygesen et al. 2019):

1. Evidence of myocardial necrosis (either changes in cardiac biomarkers or postmortem pathological findings); and
2. Supporting information derived from the clinical presentation, electrocardiographic changes, or the results of myocardial or coronary artery imaging.

The totality of the clinical, electrocardiographic, and cardiac biomarker information should be considered to determine whether a MI has occurred. Specifically, timing and trends in cardiac biomarkers and electrocardiographic information require careful analysis. The diagnosis of MI should also consider the clinical setting in which the event occurs. MI may be diagnosed for an event that has characteristics of a MI, but which does not meet the strict definition because biomarker or electrocardiographic results are not available.

10.3.1.1 Criteria for Myocardial Infarction

Clinical Presentation

The clinical presentation should be consistent with diagnosis of myocardial ischemia and infarction. Other findings that might support the diagnosis of MI should be taken into account because a number of conditions are associated with elevations in cardiac biomarkers (e.g., trauma, surgery, pacing, ablation, congestive HF, hypertrophic cardiomyopathy, pulmonary embolism, severe pulmonary hypertension, stroke or subarachnoid hemorrhage, infiltrative and inflammatory disorders of cardiac muscle, drug toxicity, burns, critical illness, extreme exertion, and chronic kidney disease). Supporting information can also be considered from myocardial imaging and coronary imaging. The totality of the data may help differentiate acute MI (AMI) from the background disease process.

10.3.1.1.1 Criteria for Acute Myocardial Infarction

- Detection of rise and/or fall of cardiac biomarkers (preferably cardiac troponin [cTn]) with at least one value above the 99th percentile of the upper reference limit (URL) or at least 1 value exceeding the local reference limit for non-highly

sensitive methods together with evidence of myocardial ischemia with at least one of the following:

  - Symptoms of ischemia
  - ECG changes indicative of new ischemia (new ST-T changes or new left bundle branch block [LBBB])
  - Development of pathological Q waves in the ECG
  - Imaging evidence of new loss of viable myocardium or new regional wall motion abnormality
  - Identification of an intracoronary thrombus by angiography.

- PCI related MI is arbitrarily defined by elevation of cTn values (>5 x 99th percentile URL) in participants with normal baseline values (≤99th percentile URL) or a rise of cTn values >20% if the baseline values are elevated and are stable or falling. In addition, either (i) symptoms suggestive of myocardial ischemia, or (ii) new ischemic ECG changes, or (iii) angiographic findings consistent with a procedural complication, or (iv) imaging demonstration of new loss of viable myocardium or new regional wall motion abnormality are required.
- Stent thrombosis associated with MI when detected by coronary angiography or autopsy in the setting of myocardial ischemia and with a rise and/or fall of cardiac biomarker values with at least one value above the 99th percentile URL.
- CABG related MI is arbitrarily defined by elevation of cardiac biomarker values (>10 x 99th percentile URL) in participants with normal baseline cTn values (≤99th percentile URL). In addition, either (i) new pathological Q waves or new LBBB, or (ii) angiographic documented new graft or new native coronary artery occlusion, or (iii) imaging evidence of new loss of viable myocardium or new regional wall motion abnormality.

10.3.1.1.2 Criteria for Prior Myocardial Infarction

- Pathological Q waves with or without symptoms in the absence of non-ischemic causes
- Imaging evidence of a region of loss of viable myocardium that is thinned and fails to contract in the absence of a non-ischemic cause
- Pathological findings of a healed or healing myocardium infarction.

10.3.1.1.3 Ischemic Symptoms

Ischemic symptoms are considered to be present if there is discomfort in the chest, upper extremity, mandibular or epigastric region lasting greater than 20 minutes. The discomfort is usually diffuse and may be accompanied by diaphoresis, nausea, or syncope. Atypical symptoms may also occur with an MI such as palpitations or cardiac arrest.

10.3.1.1.4 Cardiac Markers

All pertinent enzyme results (cTn T and I, creatine kinase (CK), CK-MB (CK-myocardial band), lactate dehydrogenase, and others) available will be reviewed.

The preferred biomarker for myocardial necrosis is cTn (I or T), which has nearly absolute myocardial tissue specificity as well as high clinical sensitivity.

An increased value for cTn is defined as a measurement exceeding the 99th percentile of a normal reference population (URL). If troponin assays are not available, the best alternative is CK-MB (measured by mass assay). As with troponin, an increased CK-MB value is defined as a measurement above the 99th percentile URL.

These markers must be evaluated in the absence of known non-ischemic cause.

Enzymes are classified as "incomplete" if no enzyme data (or not enough data) are available to allow classification.

10.3.1.1.5 ECG Changes

ECG changes can be used to support or confirm a MI. Supporting evidence may be ischemic changes and confirmatory information may be new Q waves.

ECG Manifestations of Acute Myocardial Ischemia (in Absence of Left Ventricular Hypertrophy and LBBB)

ST elevation

- New ST elevation at the J point in 2 anatomically contiguous leads with the cut-points: ≥0.2 mV in men ≥40 years (≥0.25 mV in men <40 years) or ≥0.15 mV in women in leads V2-V3 and/or ≥0.1 mV in other leads.

ST depression and T-wave changes

- New horizontal or down-sloping ST depression ≥0.05 mV in 2 contiguous leads and/or new T inversion ≥0.1 mV in 2 contiguous leads with prominent R-wave or R/S ratio >1.

The above ECG criteria illustrate patterns consistent with myocardial ischemia. In patients with abnormal biomarkers, it is recognized that lesser ECG abnormalities may represent an ischemic response and may be accepted under the category of abnormal ECG findings.

ECG Changes Associated with Prior MI

- Any Q-wave in leads V2-V3 ≥0.02 seconds or QS complex in leads V2 and V3
- Q-wave ≥0.03 seconds and ≥0.1 mV deep or QS complex in leads I, II, aVL, aVF, or V4-V6 in any 2 leads of a contiguous lead grouping (I, aVL, V1-V6; II, III, and aVF)
- The same criteria are used for supplemental leads V7-V9, and for the Cabrera frontal plane lead grouping
- R-wave ≥0.04 seconds in V1-V2 and R/S ≥1 with a concordant positive T-wave in the absence of a conduction defect.

10.3.2 Stroke

Stroke is defined as an acute episode of focal or global neurological dysfunction caused by brain, spinal cord, or retinal vascular injury as a result of hemorrhage or infarction, with symptom duration of 24 hours or more. Episodes lasting less than 24 hours can be considered a stroke if there is an intervention to abort the stroke (e.g., thrombolytic therapy), diagnostic confirmation of the stroke, or participant death prior to reaching the 24-hour duration.

Subdural hematomas are intracranial hemorrhagic events and not strokes.

10.3.2.1 Ischemic Stroke

Ischemic stroke is defined as an acute episode of focal cerebral, spinal, or retinal dysfunction caused by an infarction of central nervous system tissue.

Hemorrhage may be a consequence of ischemic stroke. In this situation, the stroke is an ischemic stroke with hemorrhagic transformation and not a hemorrhagic stroke.

10.3.2.2 Hemorrhagic Stroke

Hemorrhagic stroke is defined as an acute episode of focal or global cerebral or spinal dysfunction caused by an intraparenchymal, intraventricular, or subarachnoid hemorrhage.

10.3.2.3 Undetermined Stroke

Undetermined stroke is defined as an acute episode of focal or global neurological dysfunction caused by presumed brain, spinal cord, or retinal vascular injury as a result of hemorrhage or infarctions but with insufficient information to allow categorization as Ischemic or Hemorrhagic.

Note: Given the scope of this study, disability will not be measured.

10.3.3 New Onset of HF

This definition will be used to determine if cases referred to by investigators as HF meet criteria for new onset of HF.

New onset of HF is defined as an event that meets ALL the following criteria:

1. The participant does NOT have a prior history of HF documented.
2. The participant must have all criteria #2 to #4 as noted below in Section 10.3.4.
3. The participant receives initiation of treatment specifically for HF; criteria #5, as indicated in Section 10.3.4 with the difference that the route of administration could be oral and/or intravenous.

10.3.4 Hospitalization for HF

HF requiring hospitalization is defined as an event that meets ALL the following criteria:

1. The participant is admitted to the hospital with a primary diagnosis of HF
2. The participant’s length of stay in hospital extends for at least 24 hours (or a change in calendar date if the hospital admissions and discharge times are unavailable)
3. The participant exhibits documented new symptoms or worsening symptoms due to HF on presentation, including at least ONE of the following:
    a. Dyspnea (dyspnea with exertion, dyspnea at rest, orthopnea, paroxysmal nocturnal dyspnea)
    b. Decreased exercise tolerance
    c. Fatigue
    d. Other symptoms of worsened end-organ perfusion or volume overload end-organ perfusion and volume overload will be clinically determined by the DMC members since these definitions are not protocol defined
4. The participant has objective evidence of worsening HF, consisting of at least TWO physical examination findings OR ONE physical examination finding and at least ONE laboratory criterion, including:

   a. Physical examination findings considered to be due to HF, including new or worsened:
      i. Peripheral edema
      ii. Increasing abdominal distention or ascites (in the absence of hepatic disease)
      iii. Pulmonary rales/crackles/crepitations
      iv. Increased jugular venous pressure and/or hepatojugular reflux
      v. S3 gallop
      vi. Clinically significant or rapid weight gain thought to be related to fluid retention
   b. Laboratory evidence of new or worsening HF, if obtained within 24 hours of presentation, including:
      i. Increased BNP/N-terminal pro-BNP (NT-pro BNP) or mid-regional pro-atrial natriuretic peptide concentrations consistent with decompensation of HF. In participants with chronically elevated natriuretic peptides, a significant increase should be noted above baseline
      ii. Radiological evidence of pulmonary congestion
      iii. Non-invasive or invasive diagnostic evidence of clinically significant elevated left or right sided ventricular filling pressure or low cardiac output. For example, echocardiographic criteria could include E/e >15 or D-dominant pulmonary venous inflow pattern, plethoric inferior vena cava with minimal collapse on inspiration, or decreased left ventricular outflow tract minute stroke distance (time velocity integral) OR right heart catheterization showing a pulmonary capillary wedge pressure (pulmonary artery occlusion pressure) >18 mmHg, central venous pressure >12 mmHg, or a cardiac index <2.2 L/min/m^2

5. The participant receives initiation or intensification of treatment specifically for HF, including **<u>at least ONE</u>** of the following:
   a. Intravenous diuretic, inotrope, or vasodilator therapy
   b. Mechanical or surgical intervention, including:
      i. Mechanical circulatory support (e.g., intra-aortic balloon pump, ventricular assist device)
      ii. Mechanical fluid removal (e.g., ultrafiltration, hemofiltration, dialysis).

10.3.5 Chronic Sustained Decrease in EGFR

Central laboratory values will be used for the definitions described below. Local laboratory values will not be considered. The eGFR from Day 1 will be considered as the baseline value. In cases where the eGFR from Day 1 is missing, the last value measured prior to randomization will be considered as the baseline value.

A chronic sustained decrease in eGFR is defined as a decrease in eGFR (≥40% or ≥57% compared to baseline [Day 1], or to <15mL/min/1.73m^2), observed over at least 4 weeks with at least 2 consecutive central laboratory assessments confirming the decrease.

The confirmatory sample is expected to be collected at least 4 weeks after the initial assessment showing decrease of x% or more. The date of onset of sustained decrease in eGFR ≥x% compared to baseline is the date of the initial sample exceeding the threshold.
If the participant has an initial decrease in eGFR at the EOS visit or before the EOS which has not been confirmed yet at the time of EOS visit, it should be confirmed after 4 weeks from the initial decrease.

10.4 Appendix 4: AEs and SAEs: Definitions and Procedures for Recording, Evaluating, Follow-Up, and Reporting

10.4.1 Definition of AE

AE Definition
• An AE is any untoward medical occurrence in a clinical study participant, associated with the use of study intervention, whether or not considered related to the study intervention.<br>• NOTE: An AE can therefore be any unfavorable and unintended sign (including an abnormal laboratory finding), symptom, or disease (new or exacerbated) associated with the use of study intervention.

Events Meeting the AE Definition
• Any abnormal laboratory test results (hematology, clinical chemistry, or urinalysis) or other safety assessments (e.g., ECG, radiological scans, vital signs measurements), including those that worsen from baseline, considered clinically significant in the medical and scientific judgment of the investigator (i.e., not related to progression of underlying disease).<br>• Exacerbation of a chronic or intermittent pre-existing condition including either an increase in frequency and/or intensity of the condition.<br>• New conditions detected or diagnosed after study intervention administration even though it may have been present before the start of the study.<br>• Signs, symptoms, or the clinical sequelae of a suspected intervention-intervention interaction.<br>o Signs, symptoms, or the clinical sequelae of a suspected overdose of either study intervention or a concomitant medication. Overdose per se will not be reported as an AE/SAE unless it is an intentional overdose taken with possible suicidal/self-harming intent. Such overdoses should be reported regardless of sequelae.<br>o Lack of efficacy or failure of expected pharmacological action per se will not be reported as an AE or SAE. Such instances will be captured in the efficacy assessments. However, the signs, symptoms, and/or clinical sequelae resulting from lack of efficacy will be reported as AE or SAE if they fulfill the definition of an AE or SAE.

Events NOT Meeting the AE Definition

- Any clinically significant abnormal laboratory findings or other abnormal safety assessments which are associated with the underlying disease, unless judged by the investigator to be more severe than expected for the participant's condition.
- The disease/disorder being studied or expected progression, signs, or symptoms of the disease/disorder being studied, unless more severe than expected for the participant's condition.
- Medical or surgical procedure (e.g., endoscopy, appendectomy): the condition that leads to the procedure is the AE.
- Situations in which an untoward medical occurrence did not occur (social and/or convenience admission to a hospital).
- Anticipated day-to-day fluctuations of pre-existing disease(s) or condition(s) present or detected at the start of the study that do not worsen.

10.4.2 Definition of SAE

An SAE is defined as any AE that, at any dose:

a. Results in death

b. Is life-threatening

- The term 'life-threatening' in the definition of 'serious' refers to an event in which the participant was at risk of death at the time of the event. It does not refer to an event, which hypothetically might have caused death, if it were more severe.

c. Requires inpatient hospitalization or prolongation of existing hospitalization

- In general, hospitalization signifies that the participant has been admitted (usually involving at least an overnight stay) at the hospital or emergency ward for observation and/or treatment that would not have been appropriate in the physician's office or outpatient setting. Complications that occur during hospitalization are AEs. If a complication prolongs hospitalization or fulfills any other serious criteria, the event is serious. When in doubt as to whether "hospitalization" occurred or was necessary, the AE should be considered serious.
- Hospitalization for elective treatment of a pre-existing condition that did not worsen from baseline is not considered an AE.

d. Results in persistent or significant disability/incapacity

- The term disability means a substantial disruption of a person's ability to conduct normal life functions.
- This definition is not intended to include experiences of relatively minor medical significance such as uncomplicated headache, nausea, vomiting, diarrhea, influenza, and accidental trauma (e.g., sprained ankle) which may interfere with or prevent everyday life functions but do not constitute a substantial disruption.

e. Is a congenital anomaly/birth defect

f. Other situations:

- Medical or scientific judgment should be exercised by the investigator in deciding whether SAE reporting is appropriate in other situations such as significant medical events that may jeopardize the participant or may require medical or surgical intervention to prevent one of the other outcomes listed in the above definition. These events should usually be considered serious.
- Examples of such events include invasive or malignant cancers, intensive treatment for allergic bronchospasm, blood dyscrasias, convulsions, or development of intervention dependency or intervention abuse.

10.4.3 Recording and Follow-Up of AE and/or SAE

AE and SAE Recording

- When an AE/SAE occurs, it is the responsibility of the investigator to review all documentation (e.g., hospital progress notes, laboratory reports, and diagnostics reports) related to the event.
- The investigator will then record all relevant AE/SAE information.
- It is not acceptable for the investigator to send photocopies of the participant's medical records to the medical monitor in lieu of completion of the AE/SAE CRF required form.
  - There may be instances when copies of medical records for certain cases are requested by the sponsor. In this case, all participant identifiers, with the exception of the participant number, will be redacted on the copies of the medical records before submission to the sponsor.
  - The investigator will attempt to establish a diagnosis of the event based on signs, symptoms, and/or other clinical information. Whenever possible, the diagnosis (not the individual signs/symptoms) will be documented as the AE/SAE.

Assessment of Intensity

- The investigator will make an assessment of intensity for each AE and SAE reported during the study and assign it to 1 of the following categories:
  - Mild: An event that is easily tolerated by the participant, causing minimal discomfort and not interfering with everyday activities.
  - Moderate: An event that causes sufficient discomfort to interfere with normal everyday activities.
  - Severe: An event that prevents normal everyday activities. An AE that is assessed as severe should not be confused with an SAE. Severe is a category utilized for rating the intensity of an event; and both AEs and SAEs can be assessed as severe.
  - An event is defined as 'serious' when it meets at least 1 of the predefined outcomes as described in the definition of an SAE, NOT when it is rated as severe.

Assessment of Causality
• The investigator is obligated to assess the relationship between study intervention and each occurrence of each AE/SAE.<br>• A “reasonable possibility” of a relationship conveys that there are facts, evidence, and/or arguments to suggest a causal relationship, rather than a relationship cannot be ruled out.<br>• The investigator will use clinical judgment to determine the relationship.<br>• Alternative causes, such as underlying disease(s), concomitant therapy, and other risk factors, as well as the temporal relationship of the event to study intervention administration will be considered and investigated.<br>• The investigator will also consult the IB and/or Product Information, for marketed products, in his/her assessment.<br>• For each AE/SAE, the investigator **must** document in the medical notes that he/she has reviewed the AE/SAE and has provided an assessment of causality.<br>• There may be situations in which an SAE has occurred, and the investigator has minimal information to include in the initial report to the sponsor. However, **it is very important that the investigator always make an assessment of causality for every event before the initial transmission** of the SAE data to the sponsor.<br>• The investigator may change his/her opinion of causality in light of follow-up information and send an SAE follow-up report with the updated causality assessment.<br>• The causality assessment is one of the criteria used when determining regulatory reporting requirements.
**Follow-up of AEs and SAEs**
• The investigator is obligated to perform or arrange for the conduct of supplemental measurements and/or evaluations as medically indicated or as requested by the sponsor to elucidate the nature and/or causality of the AE or SAE as fully as possible. This may include additional laboratory tests or investigations, histopathological examinations, or consultation with other health care professionals.<br>• If a participant dies during participation in the study or during a recognized follow-up period, the investigator will provide the sponsor with a copy of any post-mortem findings including histopathology.<br>• New or updated information will be recorded in the originally submitted documents.<br>• The investigator will submit any updated SAE data to the sponsor immediately and no later than 24 hours of receipt of the information.

10.4.4 Reporting of SAEs

SAE Reporting to the sponsor via an Electronic Data Collection Tool

- The primary mechanism for reporting an SAE to the sponsor will be the electronic data collection tool.
- If the electronic system is unavailable, then the site will use the paper SAE data transmission (see next section) to report the event within 24 hours.
- The site will enter the SAE data into the electronic system as soon as it becomes available.
- After the study is completed at a given site, the electronic data collection tool will be taken off-line to prevent the entry of new data or changes to existing data.
- If a site receives a report of a new SAE from a study participant or receives updated data on a previously reported SAE after the electronic data collection tool has been taken off-line, then the site can report this information on a paper SAE form (see next section) or to the sponsor medical monitor by telephone.
- Contacts for SAE reporting can be found in the investigator site file.

SAE Reporting to the sponsor via Paper Data Collection Tool

- Email transmission of the SAE paper data collection tool is the preferred method to transmit this information to the sponsor medical monitor.
- In rare circumstances and if email transmission is not feasible, notification by telephone is acceptable with a copy of the SAE data collection tool sent by overnight mail or courier service.
- Initial notification via telephone does not replace the need for the investigator to complete and sign the SAE data collection tool within the designated reporting time frames.
- Contacts for SAE reporting can be found in the investigator site file.

10.5 Appendix 5: Death Events Definition

10.5.1 Cardiovascular Death

CV death includes death resulting from an AMI, sudden cardiac death, undetermined death, death due to HF, death due to stroke, death due to CV procedures, and death due to other CV causes, as follows:

10.5.1.1 Death due to Acute Myocardial Infarction

Death due to AMI refers to a death by any CV mechanism (e.g., arrhythmia, sudden death, HF, stroke, pulmonary embolus, peripheral artery disease) within 30 days after an MI and related to the immediate consequences of the MI, such as progressive HF or recalcitrant arrhythmia. AMI should be verified to the extent possible by the diagnostic criteria outlined for AMI or by autopsy findings showing recent MI or recent coronary thrombosis.

Death resulting from a procedure to treat a MI (PCI, CABG), or to treat a complication resulting from MI should also be considered death due to AMI.

Death resulting from an elective coronary procedure to treat myocardial ischemia (i.e., chronic stable angina) or death due to an MI that occurs as a direct consequence of a CV investigation/procedure/operation should be considered as a death due to a CV procedure.

Note: If within the 30 days following a MI event, the participant does not die, or dies from a cause that is not directly related to the MI event, then the MI will be classified as non-fatal.

10.5.1.2 Sudden Cardiac Death

Sudden Cardiac Death refers to a death that occurs unexpectedly, not following an AMI, (i.e., not within 30 days of an AMI) and includes the following deaths:

- Death witnessed and instantaneous without new or worsening symptoms
- Death witnessed within 1 hour of the onset of new or worsening cardiac symptoms, unless the symptoms suggest AMI
- Death witnessed and attributed to an identified arrhythmia (e.g., captured on an electrocardiographic [ECG] recording, witnessed on a monitor, or unwitnessed but found on implantable cardioverter-defibrillator review)
- Death after unsuccessful resuscitation from cardiac arrest
- Death after successful resuscitation from cardiac arrest and without identification of a specific etiology
- Unwitnessed death in a participant seen alive and clinically stable ≤24 hours prior to being found dead without any evidence supporting a specific non-CV cause of death (information regarding the participant’s clinical status preceding death should be provided, if available).

10.5.1.3 Undetermined Death

- For participants who were NOT observed alive within 24 hours of death and without any other likely cause of death, undetermined cause of death should be recorded (e.g., a participant found dead in bed but who had not been seen by family members for >24 hours).
- Considering the targeted participant population and the competing causes of death, undetermined cause of death will by default to CV death.

10.5.1.4 Death due to Heart Failure

Death due to HF or cardiogenic shock refers to a death in association with clinically worsening symptoms and/or signs of HF without evidence of another cause and NOT following an AMI. Deaths due to HF can have various etiologies, including single or recurrent MIs (late effect, i.e., >30 days), ischemic or non-ischemic cardiomyopathy, hypertension, or valvular disease. There is no other identified cause of death other than HF.

Death due to HF or cardiogenic shock should include sudden death occurring during an admission for worsening HF as well as death from progressive HF or cardiogenic shock following implantation of a mechanical assist device.

No evidence of AMI or stroke in the previous 30 days.

New or worsening signs and/or symptoms of HF include any of the following:

- New or increasing symptoms and/or signs of HF requiring the initiation of, or an increase in, treatment directed at HF or occurring in a participant already receiving maximal therapy for HF
- HF symptoms or signs requiring intravenous therapy or chronic oxygen administration for hypoxia due to pulmonary edema
- Confinement to bed predominantly due to HF symptoms
- Pulmonary edema sufficient to cause tachypnea and distress not occurring in the context of an AMI, worsening renal function, or as the consequence of an arrhythmia
- Cardiogenic shock not occurring in the context of an AMI or as the consequence of an arrhythmia.

Cardiogenic shock is defined as SBP <90 mm Hg for greater than 1 hour, not responsive to fluid resuscitation and/or heart rate correction, and felt to be secondary to cardiac dysfunction and associated with at least one of the following signs of hypoperfusion:

- Cool, clammy skin or
- Oliguria (urine output <30 mL/hour) or
- Altered sensorium or
- Cardiac index <2.2 L/min/m^2.

Cardiogenic shock can also be defined if SBP <90 mm Hg and increases to ≥ 90 mm Hg in less than 1 hour with positive inotropic or vasopressor agents alone and/or with mechanical support.

For diagnosis of death due to HF, it will be evaluated whether new onset of HF criteria were also met, as indicated in Section 10.3.3.

10.5.1.5 Death due to Stroke

Death due to stroke refers to death within 30 days after a stroke that is either a direct consequence of the stroke or a complication of the stroke. Acute stroke should be verified to the extent possible by the diagnostic criteria outlined for stroke.

Note: If within the 30 days following a stroke event, the participant does not die, or dies from a cause that is not directly related to the stroke event, then the stroke will be classified as non-fatal.

10.5.1.6 Death due Cardiovascular Procedures

Death due to CV procedures refers to death within 30 days caused by the immediate complications of a CV procedure.

10.5.1.7 Death due to Other Cardiovascular Causes

Death due to other CV causes refers to a CV death not included in the above categories (e.g., CV hemorrhage, pulmonary embolism, or peripheral arterial disease). Non-stroke intracranial hemorrhage, non-procedural or non-traumatic vascular rupture (e.g., aortic rupture) or hemorrhage causing cardiac tamponade are considered CV hemorrhages. Any other bleeding should be considered as non-CV.

Note: Death due to CV hemorrhage refers to death related to hemorrhage such as a non-stroke intracranial hemorrhage, non-procedural or non-traumatic vascular rupture (e.g., aortic

aneurysm), or hemorrhage causing cardiac tamponade. Bleeding that does not fall under the mentioned conditions will be considered as non-CV event.

10.5.2 Renal Death

The following events will be classified as renal death when they satisfy the following criteria:

1. The participant dies

AND

2. Renal replacement therapy (RRT) has not been initiated (although clinically indicated, e.g., death due to progressive kidney failure occurs before RRT can be introduced)
    a. If a participant has advanced kidney failure and is denied dialysis or refuses dialysis and dies, then death is eligible to be called renal death
    b. If there is a reason that the participant was denied RRT in the first place, then another more proximal cause of death will be identified. As examples:
        i. Cancer death if the participant refused dialysis due to metastatic cancer
        ii. CV death, because the participant was in shock and the participant did not want dialysis to be done would not be called renal death
        iii. Infection death (e.g., septic shock)

AND

3. There is no likely other cause of death.

Note: In a participant who is already on dialysis and decides to withdraw from dialysis, death is due to withdrawal of dialysis. Such a participant did not die because lack of initiation of dialysis therefore this is not called “renal death” in this study.

General Considerations

The RRT, that is not initiated, must be directly related to the kidney failure to be considered for renal death definition. RRT, that is not initiated, related to another disease condition (e.g., volume overload) and unrelated to kidney failure should NOT be considered in renal death definition. The proximal cause of death must be directly related to kidney failure.

10.5.3 Non-Cardiovascular and Non-Renal Deaths

Non-CV and non-renal death are defined as any death that is not thought to be due to a CV cause or renal cause. Non-CV and non-renal causes of death will be categorized as follows:

- Infection
- Malignancy
- Other (specify).

10.6 Appendix 6: Contraceptive Guidance and Collection of Pregnancy Information

Definitions:

Woman of Childbearing Potential (WOCBP)

A woman is considered fertile following menarche and until becoming postmenopausal unless permanently sterile (see below).

If fertility is unclear (e.g., amenorrhea in adolescents or athletes) and a menstrual cycle cannot be confirmed before first dose of study intervention, additional evaluation should be considered.

Women in the following categories are not considered WOCBP

1. Premenarchal
2. Premenopausal female with 1 of the following:
   - Documented hysterectomy
   - Documented bilateral salpingectomy
   - Documented bilateral oophorectomy.

   For individuals with permanent infertility due to an alternate medical cause other than the above, (e.g., Mullerian agenesis, androgen insensitivity), investigator discretion should be applied to determining study entry.

   Note: Documentation can come from the site personnel's: review of the participant's medical records, medical examination, or medical history interview.
3. Postmenopausal female
   - A postmenopausal state is defined as no menses for 12 months without an alternative medical cause.
     - A high FSH level in the postmenopausal range may be used to confirm a postmenopausal state in women not using hormonal contraception or HRT. However, in the absence of 12 months of amenorrhea, confirmation with more than 1 FSH measurement is required.
   - Females on HRT and whose menopausal status is in doubt will be required to use one of the non-estrogen hormonal highly effective contraception methods if they wish to continue their HRT during the study. Otherwise, they must discontinue HRT to allow confirmation of postmenopausal status before study enrollment.

Contraception Guidance:

WOCBP can only be included in the study if a pregnancy test is negative at the screening visit and if they agree to use adequate contraception during the study and until 8 weeks after last study interventions dose. Adequate contraception is defined as any combination of at least 2 effective methods of birth control, of which at least one is a physical barrier (e.g., condoms with hormonal contraception or implants or combined oral contraceptives, certain intrauterine devices).

Collection of Pregnancy Information for Female Participants who Become Pregnant:

- The investigator will collect pregnancy information on any female participant who becomes pregnant while participating in this study. The initial information will be recorded on the appropriate form and submitted to the sponsor within 24 hours of learning of a participant pregnancy.
- The participant will be followed to determine the outcome of the pregnancy. The investigator will collect follow-up information on the participant and the neonate, and the information will be forwarded to the sponsor. Generally, follow-up will not be required for longer than 6 to 8 weeks beyond the estimated delivery date. Any termination of pregnancy will be reported, regardless of fetal status (presence or absence of anomalies) or indication for the procedure.
- While pregnancy itself is not considered to be an AE or SAE, any pregnancy complication or elective termination of a pregnancy for medical reasons will be reported as an AE or SAE.
- A spontaneous abortion (occurring at <22 weeks' gestational age) or still birth (occurring at >22 weeks' gestational age) is always considered to be an SAE and will be reported as such.
- Any post-study pregnancy-related SAE considered reasonably related to the study intervention by the investigator will be reported to the sponsor as described in Section 8.3.4. While the investigator is not obligated to actively seek this information in former study participants, he or she may learn of an SAE through spontaneous reporting.
- Any female participant who becomes pregnant while participating in the study will discontinue study intervention and be withdrawn from the study after completing ED visit.

10.7 Appendix 7: Calculating the Child Pugh Score

The severity of liver disease (Table 10-2) will determine the Child Pugh score (Table 10-3).

Table 10-2: Grading of Severity of Liver Disease (adapted from *Pugh et al., 1973*)

**Factor**	+1	+2	+3
**Bilirubin (mg/dL)**	<2	2 – 3	>3
**Albumin (g/dL)**	>3.5	2.8 – 3.5	<2.8
**International Normalized Ratio**	<1.7	1.7 – 2.3	>2.3
**Ascites**	None	Mild	Moderate/Severe
**Encephalopathy**	None	Grade I - II	Grade III - IV

Table 10-3: Classification Using the Added Score from Table 10-2

**Child-Pugh Class**	A	B	C
**Points**	5 – 6	7 - 9	10 - 15

10.8 Appendix 8: Guidance on Use of Common CYP Inhibitors and Inducers

Table 10-4 lists the most common medications regarded as potent CYP3A4 inhibitors and moderate or potent inducers. In addition, examples of allowed weak or moderate CYP3A4 inhibitors are given.

Table 10-4: Cytochrome P450: List of Concomitant Medication

Excluded Cytochrome P450 Isoenzyme 3A4 (CYP3A4) inducers	Excluded CYP3A4 inhibitors	Allowed CYP3A4 inhibitors
apalutamide	adagrasib	amlodipine
asunaprevir/beclabuvir/daclatasvir	amprenavir	amiodarone
avasimibe	atazanavir and other inhibitors of human HIV protease	aprepitant
bosentan	boceprevir	berotralstat
carbamazepine	ceritinib	bicalutamide
cenobamate	clarithromycin	chloramphenicol
dabrafenib	cobicistat	cilostazol
efavirenz	danoprevir	cimetidine
elagolix	darunavir	ciprofloxacin
enzalutamide	dasabuvir	conivaptan
etravirine	elvitegravir	crizotinib
fosphenytoin	ensitrelvir	cyclosporine
hypericum perforatum / St John's wort	fosamprenavir	diltiazem
ivosidenib	grapefruit juice	dronedarone
lersivirine	idelalisib	duvelisib
lesinurad	indinavir	erythromycin
lorlatinib	itraconazole	fluconazole
lumacaftor	josamycin	fluvoxamine
mephenytoin	ketoconazole	fosaprepitant
metamizole	lonafarnib	imatinib
methylphenobarbital	lopinavir	isavuconazole
mitotane	nefazodone	istradefylline
modafinil	nelfinavir	ivacaftor
nafcillin	ombitasvir	letermovir
nevirapine	paritaprevir	lomitapide
oxcarbazepine	posaconazole	mifepristone
pexidartinib	ribociclib	norfloxacine
phenobarbital	ritonavir	ranitidine
phenytoin	saquinavir	ranolazine
primidone	telaprevir	rimegepant
rifabutin	telithromycin	tacrolimus
rifapentin	tipranavir	ticagrelor
rifampicin	troleandomycin	tofisopam
semagacestat	tucatinib	verapamil
sotorasib	voriconazole	lapatinib
talviraline		dasatinib
telotristat ethyl		nilotinib
thioridazine		voxelotor
troglitazone		
vemurafenib		

HIV = human immunodeficiency virus

10.9 Appendix 9: Abbreviations

ABPM	Ambulatory blood pressure monitoring
ACEi	Angiotensin-converting enzyme inhibitor
ADA	American Diabetes Association
AE	Adverse event
AKI	Acute kidney injury
ALT	Alanine aminotransferase
AMI	Acute myocardial infarction
ARB	Angiotensin receptor blocker
ARNI	Angiotensin receptor-neprilysin inhibitor
AST	Aspartate aminotransferase
ATC	Anatomical therapeutic chemical
$AUC_{\tau,md}$	Area under the concentration vs. time curve for the expected dosing interval obtained after multiple dose administration
BMI	Body mass index
BNP	B-type natriuretic peptide
BP	Blood pressure
$C_{max,md}$	Maximum drug concentration after multiple dose administration
CABG	Coronary artery bypass graft
CI	Confidence interval
CK	Creatinine kinase
CKD	Chronic kidney disease
CKD-EPI	Chronic Kidney Disease Epidemiology Collaboration
CK-MB	Creatinine kinase-myocardial band
COVID-19	Coronavirus disease 2019
CRF	Case report form
cTn	Cardiac troponin
CV	Cardiovascular
CVD	Cardiovascular disease
CYP3A4	Cytochrome P450 isoenzyme 3A4
DBP	Diastolic blood pressure
DMC	Data Monitoring Committee
DRE	Disease-related event
ECG	Electrocardiogram
eCRF	Electronic Case Report Form
ED	Early discontinuation
e.g.	Exempli gratia, for example
eGFR	Estimated glomerular filtration rate
eGFRcr	Estimated glomerular filtration rate based on creatinine
eGFRcr-cys	Estimated glomerular filtration rate based on creatinine and cystatin C
EOS	End of study
ESKD	End-stage kidney disease
EU	European Union
FAS	Full analysis set
FDA	Food and Drug Administration
FSH	Follicle stimulating hormone
GCP	Good Clinical Practice
GFR	Glomerular filtration rate
HbA1c	Glycated hemoglobin
HF	Heart failure
HFrEF	Heart failure with reduced ejection fraction
HRT	Hormonal replacement therapy
IB	Investigator’s Brochure
ICF	Informed consent form
ICH	International Council for Harmonization
i.e.	Id est, that is
IEC	Independent Ethics Committees

IRB	Institutional Review Board
IWRS	Interactive web response system
K+	Serum/plasma potassium or potassium
LBBB	Left bundle branch block
MedDRA	Medical Dictionary for Regulatory Activities
MI	Myocardial infarction
MR	Mineralocorticoid receptor
MRA	Mineralocorticoid receptor antagonist
NT-proBNP	N-terminal prohormone B-type natriuretic peptide
OD	Once daily
PCI	Percutaneous coronary intervention
PD	Pharmacodynamic(s)
PPS	Per-protocol analysis set
PT	Preferred Term
QTL	Quality tolerance limit
RAASi	Renin-angiotensin-aldosterone system inhibitors
RRT	Renal replacement therapy
SAC	Statistical analysis center
SAE	Serious adverse event
SAF	Safety analysis set
SAP	Statistical analysis plan
SARS-CoV-2	Severe acute respiratory syndrome coronavirus 2
SBP	Systolic blood pressure
SC	Steering Committee
SDv	Standard deviation
SGLT2i	Sodium/glucose cotransporter-2 inhibitor(s)
SGLT-1/2i	Combined SGLT-1 and 2 inhibitor(s)
SMQ	Standardized MedDRA queries
SoA	Schedule of activities
SoC	Standard of care
SOC	System Organ Class
SUSAR	Suspected unexpected serious adverse reaction
T1D	Type 1 diabetes
T2D	Type 2 diabetes
TEAE	Treatment-emergent adverse event
TZD	Thiazolidinediones
UACR	Urinary albumin-to-creatinine ratio
ULN	Upper limit of normal
URL	Upper reference limit
US	United States (of America)
WHO-DD	World Health Organization Drug Dictionary

10.10 Appendix 10: Country/Region-specific Requirements

10.10.1 Japan

10.10.1.1 JPN-1: Country-specific requirements valid for Japan only

JPN-1 (17 DEC 2021)

10.10.1.1.1 Overview of Changes

This local protocol amendment implements country-specific modifications of the original protocol, dated 15 NOV 2021.

The protocol has been revised in the appropriate sections in order to meet requirements of the Japanese health authority (PMDA) regarding reporting of disease-related outcome events when these events meet the definition of SAEs.

Revision of details regarding the documentation and reporting of (S)AEs is clarified in the protocol. In protocol version 1.0, the Disease-Related Events were not to be documented as (S)AEs but only as DREs. In order to comply with Japanese regulatory requirements, the following disease-related events will also be documented as (S)AEs in Japan:

- Kidney failure
- Renal death
- Chronic sustained decrease in eGFR
- Cardiovascular (CV) death
- Non-fatal stroke
- Non-fatal myocardial infarction (MI)
- Hospitalization for heart failure (HF)
- New onset of HF

However, in order to maintain the integrity of the study, SUSARs that derive from any of these outcome events will be waived from unblinding. Blinded SUSAR reports will be sent to the Japanese Authorities in an expedited manner to fulfill local regulatory requirements.

In addition, clarification that Japanese modification of the Chronic Kidney Disease Epidemiology Collaboration (CKD-EPI) equation needs be used in study sites in Japan is added to the protocol. In the equation modified for Japan, the coefficient of 0.813 is to be used as multiplicative factor.

Section # and Name	Description of Change	Brief Rationale
Section 5.1 Inclusion Criteria, Section 8.2.5.1 eGFR, Section 9.3 Analysis Sets, Section 9.4.3.2 Demography and Other Baseline Characteristics, Section 10.2 Appendix 2: Clinical Laboratory Tests, and Section 11 References	Clarification regarding the CKD-EPI creatinine equation (Levey et al. 2009) to be used for Japanese participants.	Japanese modification of CKD-EPI with coefficient of 0.813 (Horio et al 2010) is to be used in Japan.

Section # and Name	Description of Change	Brief Rationale
Section 8.3.7 Disease-Related Events and/or Disease-Related Outcomes Not Qualifying for Expedited Reporting as AE or SAE	A DRE will be recorded as an adverse event (AE) or a serious adverse event (SAE). The standard process for expedited reporting of SAEs will be followed if the event meets the definition of a SAE.	To comply with the guidance about DREs from the Pharmaceuticals and Medical Devices Agency (PMDA) where only some serious outcomes pre-defined as efficacy endpoints can be considered as DREs and not be subject to the normal expedited reporting.

10.10.1.1.2 Changes to the Protocol Text

Clean amended text is provided below.

Section 5.1 Inclusion Criteria

[…]

2. Participant with a clinical diagnosis of CKD and the following:
    - In Part A: eGFR 40-90 ml/min/1.73m^2 (with no more than 20% having an eGFR >75 ml/min/1.73m^2) using Chronic Kidney Disease Epidemiology Collaboration (CKD-EPI) formula* (Levey et al. 2009) at screening visit and at least one historical value of eGFR <60 mL/min/1.73 m^2 within 3 months or have a registered diagnosis of CKD.
    - In Part B: eGFR 30-90 ml/min/1.73m^2 (with no more than 20% having an eGFR >75 ml/min/1.73m^2) using CKD-EPI formula* (Levey et al. 2009) at screening visit and at least one historical value of eGFR <60 mL/min/1.73 m^2 within 3 months or have a registered diagnostic of CKD (see Section 2.2).

*Japanese modified coefficient (0.813; Horio et al. 2010) for the Levey et al. 2009 formula will be used for Japanese participants.

[…]

Section 8.2.5.1 eGFR

eGFR will be automatically calculated by the central laboratory by using the CKD-EPI creatinine equation (Levey et al. 2009). Estimate GFR values calculated with this formula (eGFRcr) will be used for screening/eligibility and primary data analysis. For central and local laboratory CKD-EPI creatinine equation (Levey et al. 2009), Japanese coefficient of 0.813 (Horio et al. 2010) is used for sites located in Japan.

Section 8.3.7 Disease-Related Events and/or Disease-Related Outcomes Not Qualifying for Expedited Reporting as AE or SAE

[…]

Note: These rules will not entirely apply to participants enrolled in Japan. DREs (kidney failure, renal death, chronic sustained decrease in eGFR, CV death, non-fatal stroke, non-fatal MI, hospitalization for HF, and new onset of HF) have to be reported as AE/SAE for Japanese participants. If an event fulfills the definition of SAE, the investigator must report it in a timely manner as provided in Appendix 4.

However, in order to maintain the integrity of the study, SUSARs that derive from any of these above listed CV and kidney events will be waived from unblinding. Blinded SUSAR reports will be sent to the Japanese Authorities in an expedited manner to fulfill local regulatory requirement.

Section 9.3 Analysis Sets

[…]

This assessment will use the 2009 CKD-EPI formula (Levey et al. 2009). Note: the Japanese coefficient of 0.813 [Horio et al. 2010] will be used for participants enrolled in Japan.

[…]

Section 9.4.3.2 Demography and Other Baseline Characteristics

[…]

Other baseline characteristics include baseline UACR, K+, categories for K+ (≤4.5 mmol and >4.5 mmol), eGFR (calculated by CKD-EPI [Levey et al. 2009] formula; the Japanese coefficient of 0.813 [Horio et al. 2010] will be used for participants enrolled in Japan), serum creatinine, HbA1c, and values for vital signs parameters (i.e., systolic BP, diastolic BP, and pulse rate).

[…]

Section 10.2 Appendix 2: Clinical Laboratory Tests

Table 10-1: Protocol-Required Laboratory Tests

Laboratory Tests	Parameters			
[...]				
Clinical chemistry				
[...]				
	Creatinine	Cystatin C	eGFRcr (CKD-EPI creatinine, Levey et al. 2009; Japanese coefficient of 0.813 [Horio et al. 2010] to be applied for participants enrolled in Japan) and eGFRcr-cys (CKD-EPI creatinine-cystatin C, Inker et al. 2021)	
[...]				

Section 11 References

Horio M, Imai E, Yasuda Y, Watanabe T, Matsuo S. Modification of the CKD epidemiology collaboration (CKD-EPI) equation for Japanese: accuracy and use for population estimates. Am J Kidney Dis. 2010;56(1):32-8.

10.10.2 India

In India, in order to comply with Indian regulatory requirements, all disease-related outcome events (DREs) (see Section 8.3.7) that meet the definition of an SAE will be documented as both SAEs and outcome events and will be reported according to the standard process for expedited reporting of SAEs. These DREs will be monitored by an unblinded independent Data Monitoring Committee, on a routine basis.

However, in order to maintain the integrity of the trial, SUSARs that derive from any of these outcome events will be waived from unblinding. Blinded SUSAR reports will be sent to the Indian Authorities in an expedited manner to fulfill local regulatory requirements.

11. References

Afkarian M, Sachs MC, Kestenbaum B, et al. Kidney disease and increased mortality risk in type 2 diabetes. J Am Soc Nephrol 2013; 24: 302–308.

Agarwal R, Anker SD, Bakris G, et al. Investigating new treatment opportunities for patients with chronic kidney disease in type 2 diabetes: the role of finerenone. Nephrol Dial Transplant 2020 Dec 6:gfaa294. doi: 10.1093/ndt/gfaa294. Epub ahead of print. PMID: 33280027.

Agarwal R, Kolkhof P, Bakris G, et al. Steroidal and non-steroidal mineralocorticoid receptor antagonists in cardiorenal medicine. European Heart Journal 2021; Jan 7; 42(2): 152-161.

Agiostratidou G, Anhalt H, Ball D, et al. Standardizing clinically meaningful outcome measures beyond HbA1c for type 1 diabetes: a consensus report of the American Association of Clinical Endocrinologists, the American Association of Diabetes Educators, the American Diabetes Association, the Endocrine Society, JDRF International, The Leona M. and Harry B. Helmsley Charitable Trust, the Pediatric Endocrine Society, and the T1D Exchange. Diabetes Care 2017; 40: 1622–1630.

American Diabetes Association. 2. Classification and Diagnosis of Diabetes: Standards of Medical Care in Diabetes - 2021. Diabetes Care 2021;44(Supplement 1): S15-S33.

American Diabetes Association. Microvascular complications and foot care: Standards of Medical Care in Diabetes - 2021. Diabetes Care 2021;44(Supplement 1): S151-S167.

Bae JH, Park EG, Kim S, et al. Effects of sodium-glucose cotransporter 2 inhibitors on renal outcomes in patients with type 2 diabetes: A systematic review and meta-analysis of randomised controlled trials. Sci Rep 2019;9, 13009.

Bakris GL, Agarwal R, Anker SD, et al. Effect of finerenone on chronic kidney disease outcomes in type 2 diabetes. N Engl J Med 2020; 383: 2219-2229.

Bakris GL, Agarwal R, Chan JC, et al. Effect of finerenone on albuminuria in patients with diabetic nephropathy: a randomised clinical trial. JAMA 2015; 314 (9): 884-894.

Cherney D, Lund SS, Perkins BA, et al. The effect of sodium glucose cotransporter 2 inhibition with empagliflozin on microalbuminuria and macroalbuminuria in patients with type 2 diabetes. Diabetologia 2016; 59 (9): 1860-1870.

Cherney D, Zinman B, Inzucchi SE, et al. Effects of empagliflozin on the urinary albumin-to-creatinine ratio in patients with type 2 diabetes and established cardiovascular disease: an exploratory analysis from the EMPA-REG OUTCOME randomised, placebo-controlled trial. The Lancet Diabetes & Endocrinology 2017; 5(8): 610-621.

Cohen JB, Yang W, Li L, et al. Time-Updated Changes in Estimated GFR and Proteinuria and Major Adverse Cardiac Events: Findings from the Chronic Renal Insufficiency Cohort (CRIC) Study. Am J Kidney Dis. 2021; S0272-6386(21)00629-6.

DeFronzo, RA, Lewin A, Patel S, et al. Combination of empagliflozin and linagliptin as second-line therapy in participants with type 2 diabetes inadequately controlled on metformin. Diabetes Care 2015; 38(3): 384.

Fernandez B, Elewa U, Sanchez-Nino MD, et al. 2012 update on diabetic kidney disease: the expanding spectrum, novel pathogenic insights and recent clinical trials. Minerva Med 2012; 103 (4): 219-34.

Filippatos G, Anker SD, Agarwall R, et al. Finerenone and Cardiovascular Outcomes in Patients with Chronic Kidney Disease and Type 2 Diabetes. Circulation. 2021; 143: 540-552.

Fox CS. Associations of kidney disease measures with mortality and end-stage renal disease in individuals with and without diabetes: a meta-analysis. Lancet 2012; 380 (9854): 1662-1673

GBD Chronic Kidney Disease Collaboration. Global, regional, and national burden of chronic kidney disease, 1990-2017: a systematic analysis for the Global Burden of Disease Study 2017. Lancet 2020; 395 (10225): 709-733.

Go AS, Chertow GM, Fan D, et al. Chronic kidney disease and the risks of death, cardiovascular events, and hospitalization. N Engl J Med 2004; 351 (13): 1296-1305.

Heerspink HJL, de Zeeuw D. Are post-trial observational studies useful? J Am Soc Nephrol. 2014; 25 (10): 2148-50.

Heerspink HJL, Stefánsson BV, Correa-Rotter R, et al. Dapagliflozin in patients with chronic kidney disease. N Engl J Med 2020; 383: 1436-1446.

Heerspink HJL, Greene T, Tighiouart H, et al. Change in albuminuria as a surrogate endpoint for progression of kidney disease: a meta-analysis of treatment effects in randomised clinical trials. Lancet Diabetes Endocrinol 2019; 7(2): 128-139.

Heerspink HJL, Avraham K, Thuresson M, et al. Kidney outcomes associated with use of SGLT2 inhibitors in real-world clinical practice (CVD-REAL 3): a multinational observational cohort study. Lancet Diabetes Endocrinol. 2020; 8: 27-35.

Herrington WG, Preiss D, Haynes R, et al. The potential for improving cardio-renal outcomes by sodium-glucose co-transporter-2 inhibition in people with chronic kidney disease: a rationale for the EMPA-KIDNEY study.CKJ, 2018; 749-761.

Inker LA, Eneanya ND, Coresh J, et al. New Creatinine- and Cystatin C–Based Equations to Estimate GFR without Race. N Engl J Med 2021 Sep 23. doi: 10.1056/NEJMoa2102953. Online ahead of print.

International Diabetes Federation. IDF Diabetes Atlas. 8th edn. Brussels, Belgium: International Diabetes Federation, 2017.

Jardiance® EU SmPC - European Medicines Agency web site: http://www.ema.europa.eu.

Kidney Disease: Improving Global Outcomes (KDIGO) Blood Pressure Work Group. KDIGO Clinical Practice Guideline for the Management of Blood Pressure in Chronic Kidney Disease. Kidney Int Suppl 2012;2:337–414.

Kidney Disease: Improving Global Outcomes (KDIGO) CKD Work Group. KDIGO 2012 clinical practice guideline for the evaluation and management of chronic kidney disease. Kidney Int Suppl 2013; 3: 1–150.

Kidney Disease: Improving Global Outcomes (KDIGO) CKD Work Group. KDIGO clinical practice guideline for acute kidney injury. Kidney Int Suppl 2012; 2: 19–36.

Kolkhof P, Jaisser F, Kim SY, et al. Steroidal and Novel Non-steroidal Mineralocorticoid Receptor Antagonists in Heart Failure and Cardiorenal Diseases: Comparison at Bench and Bedside. Handb Exp Pharmacol. 2017;243:271-305. doi: 10.1007/164_2016_76. PMID: 27830348.

Kolkhof P, Pavkovic M, Hartmann E, et al. Combined efficacy of the novel nonsteroidal and selective mineralocorticoid receptor antagonist finerenone and the SGLT2 inhibitor empagliflozin in a non-diabetic cardiorenal rat model. J Am Soc Nephrol 2021; 31 (243).

Levin A, Perkovic V, Wheeler DC, et al. Empagliflozin and Cardiovascular and Kidney Outcomes across KDIGO Risk Categories. Post Hoc Analysis of a Randomised, Double-Blind, Placebo-Controlled, Multinational Trial. Clin J Am Soc Nephrol 2020; 15 (10): 1433-1444.

Levey AS, Stevens LA, Schmid CH, et al. A new equation to estimate glomerular filtration rate. Ann Intern Med. 2009; 150 (9): 604-12.

Levey AS, Gansevoort RT, Coresh J, et al. Change in albuminuria and GFR as end points for clinical trials in early stages of CKD: A scientific workshop sponsored by the National Kidney Foundation in collaboration with the US Food and Drug Administration and European Medicines Agency. Am J Kidney Dis. 2019; 75(1): 84-104.

Matsushita K, van der Velde M, Astor BC, et al. Association of estimated glomerular filtration rate and albuminuria with all-cause and cardiovascular mortality in general population cohorts: a collaborative meta-analysis. Lancet 2010; 375 (9731): 2073-81.

McGuire DK, Zinman B, Elnzucchi S, et al. Effects of empagliflozin on first and recurrent clinical events in patients with type 2 diabetes and atherosclerotic cardiovascular disease: a secondary analysis of the EMPA-REG OUTCOME trial. Lancet Diabetes Endocrinol. 2020; 8: 959-59.

McMurray JJV, Solomon SD, Inzucchi SE, et al. Dapagliflozin in patients with heart failure and reduced ejection fraction. N Engl J Med 2019; 381(21): 1995-2008.

Molitch ME, Adler AI, Flyvbjerg A, et al. Diabetic kidney disease: a clinical update from Kidney Disease: Improving Global Outcomes. Kidney Int. 2014.

Packer M, Anker SD, Butler J, et al. Cardiovascular and renal outcomes with empagliflozin in heart failure. N Engl J Med 2020; 383(15): 1413-1424.

Perkovic V, Jardine MJ, Neal B, et al. Canagliflozin and renal outcomes in type 2 diabetes and nephropathy. N Engl J Med 2019; 380 (24): 2295-2306.

Pitt B, Kober L, Ponikowski P, et al. Safety and tolerability of the novel non-steroidal mineralocorticoid receptor antagonist BAY 94-8862 in patients with chronic heart failure and mild or moderate chronic kidney disease: a randomised, double-blind trial. European Heart Journal 2013; Aug 14;34(31):2453-2563.

Pitt B, Filippatos G, Agarwal R, et al. Cardiovascular Events with Finerenone in Kidney Disease and Type 2 Diabetes. N Engl J Med 2021; Aug 28. doi: 10.1056/NEJMoa2110956. Online ahead of print.

Pugh RN, Murray-Lyon IM, Dawson JL, et al. Transection of the oesophagus for bleeding oesophageal varices. Br J Surg 1973; 60 (8): 646–649.

Ridderstråle M, Andersen KR, Zeller C, et al. Comparison of empagliflozin and glimepiride as add-on to metformin in patients with type 2 diabetes: a 104-week randomised, active-controlled, double-blind, phase 3 trial. The Lancet Diabetes & Endocrinology 2014; 2(9): 691-700.

Rossing P, Agarwal R, Anker S, et al. Finerenone in Patients with CKD and T2D by SGLT2i Treatment: An Analysis of the FIDELIO-DKD Study (poster abstract). Diabetes 2021; 70(supplement 1): 14-LB.

Shen L, Kristensen SL, Bengtsson O, et al. Dapagliflozin in HFrEF patients treated with mineralocorticoid receptor antagonists: an analysis of DAPA-HF. JACC: Heart Failure 2021; 9 (4): 254-264.

Søfteland E, Meier JJ, Vangen B, et al. Empagliflozin as add-on therapy in patients with type 2 diabetes inadequately controlled with linagliptin and metformin: a 24-week randomized, double-blind, parallel-group trial. Diabetes Care 2017; 40(2): 201-209.

Thygesen K, Alpert JS, Jaffe AS, et al. Fourth universal definition of myocardial infarction. European Heart Journal 2019; 40(3): 237-269.

Tuttle KR, Bakris GL, Bilous RW, et al. Diabetic kidney disease: a report from an ADA Consensus Conference. Diabetes Care 2014; 37 (10): 2864-83.

Wang Y, Hu X, Liu X, Wang Z. An overview of the effect of sodium glucose cotransporter 2 inhibitor monotherapy on glycemic and other clinical laboratory parameters in type 2 diabetes patients. Ther Clin Risk Manag. 2016; 12: 1113-1131.

Wanner C, Inzucchi S, Zinman B. Consistent effects of empagliflozin on cardiovascular and kidney outcomes irrespective of diabetic kidney disease catategories: Insights from the EMPA-REG OUTCOME trial. Diabetes Obes Metab. 2020; 22: 2335-2347.

Zinman B, Wanner C, Lachin JM, et al. Empagliflozin, Cardiovascular Outcomes, and Mortality in Type 2 Diabetes. N Engl J Med 2015; 373: 2117-28.

Title Page

Protocol Title: A parallel-group treatment, Phase 2, double-blind, three-arm study to assess efficacy and safety of finerenone plus empagliflozin compared with either finerenone or empagliflozin in participants with chronic kidney disease and type 2 diabetes.

Protocol Number: 21839

Compound Number: Finerenone / BAY 94-8862

Short Title: Combination of finerenone and empagliflozin in participants with CKD and T2D

Acronym: CONFIDENCE (**CO**mbinatio**N** effect of **FI**nerenone an**D** **E**mpagliflozi**N** in participants with CKD and T2D using an UA**CR** **E**ndpoint study)

Sponsor Name: Bayer AG

Legal Registered Address: Non-US territories: Bayer AG, 51368 Leverkusen, Germany

US territory: Bayer HealthCare Pharmaceuticals Inc., 100 Bayer Boulevard, P.O. Box 915,

Whippany NJ 07981-0915, USA

Regulatory Agency Identifier Number(s): IND: 117847

**Registry**	**ID**
EudraCT	2021-003037-11
Clinicaltrials.gov	NCT05254002

Date: 13 September 2024

Version: Final v1.0

Confidential

The information provided in this document is strictly confidential and is intended solely for the performance of the clinical investigation. Reproduction or disclosure of this document, whether in part or in full, to parties not associated with the clinical investigation or its use for any other purpose without the prior written consent of the sponsor is not permitted.

Throughout this document, symbols indicating proprietary names (®, TM) may not be displayed. Hence, the appearance of product names without these symbols does not imply that these names are not protected.

This Statistical Analysis Plan is produced on a word-processing system and bears no signatures.
The approval of the Statistical Analysis Plan is documented in a separate signature document.

Table of Contents

Title Page ---------- **1**
Protocol Title: ---------- 1
Protocol Number: ---------- 1
Compound Number: ---------- 1
Short Title: ---------- 1
Acronym: ---------- 1
Sponsor Name: ---------- 1
Legal Registered Address: ---------- 1
Regulatory Agency Identifier Number(s): ---------- 1

Table of Contents ---------- **2**

Version History ---------- **4**

1. Introduction ---------- **5**
1.1. Objectives, Endpoints, and Estimands ---------- 6
1.2. Study Design ---------- 9

2. Statistical Hypotheses ---------- **12**
2.1. Multiplicity Adjustment ---------- 12

3. Analysis Sets ---------- **13**

4. Statistical Analyses ---------- **15**
4.1. General Considerations ---------- 15
4.2. COVID-19 Pandemic Related Data ---------- 15
4.3. Handling of Dropouts ---------- 16
4.4. Handling of Missing Data ---------- 16
4.5. Interim Analyses and Data Monitoring ---------- 17
4.6. Data Rules ---------- 18
4.6.1. Baseline values ---------- 18
4.6.2. Change from baseline ---------- 18
4.6.3. Other data handling ---------- 18
4.6.4. GCP Violation by SMO at Site 20003 in Japan ---------- 19
4.7. Blind Review ---------- 19
4.8. Population Characteristics ---------- 20
4.8.1. Disposition ---------- 20
4.8.2. Demography and other baseline characteristics ---------- 20
4.8.3. Medical History ---------- 20
4.8.4. Prior and Concomitant Medications ---------- 20
4.9. Primary Endpoints and Estimands Analysis ---------- 22
4.9.1. Definition of Endpoints ---------- 22
4.9.2. Main Analytical Approach ---------- 22
4.9.3. Sensitivity Analyses ---------- 23
4.10. Secondary Endpoints Analysis ---------- 26
4.10.1. Key Secondary Endpoints ---------- 27
4.11. Exploratory Endpoints Analysis ---------- 27
4.11.1. Pharmacodynamics ---------- 27
4.12. Safety Analyses ---------- 28
4.12.1. Extent of Exposure ---------- 29

4.12.2. Adverse Events ---------- 30
4.12.3. Additional Safety Assessments ---------- 32
4.13. Other Analyses ---------- 36
4.13.1. Other Variables and Parameters ---------- 36
4.13.2. Subgroup Analyses ---------- 36
4.14. Changes to Protocol-planned Analyses ---------- 37
5. Sample Size Determination ---------- 38
6. Supporting Documentation ---------- 39
6.1. Appendix 1: Schedule of Assessments ---------- 39
6.2. Appendix 2: List of Abbreviations ---------- 44
6.3. Appendix 3: GCP Violation of SMO at site 20003 in Japan ---------- 46
7. References ---------- 47

Version History

SAP Version	Date	Change	Rationale
1		Not applicable	Original version

1. Introduction

This is a Statistical Analysis Plan (SAP) for study 21839 (CONFIDENCE) in finerenone.

It is based on the following document(s):

Clinical Study Protocol Amendment 1 (Protocol version 2.0), dated 23 October 2023

Data Monitoring Charter (DMC) Charter version 4.0 dated 29 July 2024

Case Report Form (CRF) version 3.0, dated 01 Feb 2022

This SAP describes the statistical analysis of the study. This is a parallel-group treatment, phase 2, double-blind, three-arm study to assess efficacy and safety of finerenone plus empagliflozin compared with either finerenone or empagliflozin alone in participants with chronic kidney disease (CKD) and Type 2 diabetes (T2D). No statistical interim analysis will be performed. An independent DMC will be involved in the review of data for safety as described in the DMC Charter. Tables, figures and listings specifications are contained in a separate document. There are no changes to the analyses described in the protocol.

Study Rationale

In the FIDELIO-DKD and FIGARO-DKD phase 3 clinical trials, finerenone, a nonsteroidal, selective antagonist of the mineralocorticoid receptor (MRA), has proven its efficacy and safety to reduce risk of kidney disease progression and cardiovascular (CV) events in patients with CKD and T2D.

Empagliflozin is a sodium-glucose cotransporter-2 inhibitor (SGLT2i) indicated to reduce the risk of CV death in adult patients with T2D and established CV disease. Empagliflozin has shown evidence of reduction of kidney disease progression and CV events in patients with or without T2D and CKD in CV trials and is now investigated in a dedicated CKD trial. Other SGLT2i (dapagliflozin and canagliflozin) showed renal and CV efficacy in patients with CKD with and without T2D, which led to a label change and updated guideline recommendations.

In a recent preclinical study, it has been shown that finerenone and empagliflozin have an additive effect on urinary protein-to-creatinine ratio, a predictor of CKD progression, and CV adverse outcomes in CKD patients.

In pooled analysis of FIDELIO-DKD/FIGARO-DKD (FIDELITY; data on file), it has been shown in the subgroup of patients treated with SGLT2i at baseline that there is added benefit in reduction of urinary albumin-to-creatinine ratio (UACR) and prevention of CV and renal events.

This study aims to demonstrate that the initial combined use of finerenone and empagliflozin is superior to either empagliflozin alone, or finerenone alone, in reducing UACR from baseline to 180 days. UACR is a measurement of albuminuria, a predictor of long-term renal and CV adverse outcomes in T2D patients.

1.1. Objectives, Endpoints, and Estimands

Objectives	Endpoints
**Primary**	
• To demonstrate that combination therapy using finerenone and empagliflozin is superior in reducing UACR than either empagliflozin or finerenone alone	Primary Endpoints:<br>• Relative change from baseline in UACR at 180 days in combination therapy group versus empagliflozin alone<br>or<br>• Relative change from baseline in UACR at 180 days in combination therapy group versus finerenone alone
	Summary measures:<br>• Mean ratio of change from baseline to Day 180 in UACR for the combination therapy group, to empagliflozin alone<br>• Mean ratio of change from baseline to Day 180 in UACR for the combination therapy group, to finerenone alone
**Secondary**	
• To further investigate the efficacy of combination therapy using finerenone and empagliflozin versus either finerenone or empagliflozin alone	• Relative change in UACR between end of treatment visit and 30 days after end of treatment visit<br>• Relative change in UACR between 30 days after end of treatment visit and baseline<br>• Relative change in UACR category (>30%, >40%, >50%) at 180 days
• To evaluate the safety of combination therapy using finerenone and empagliflozin versus either finerenone or empagliflozin alone	• Ratio of change from baseline in estimated glomerular filtration rate (eGFR) at 30 days<br>• eGFR decline greater than 30% at 30 days from baseline<br>• Ratio of change in eGFR at 180 days and 210 days from day 30<br>• Proportion of participants with of AKI events<br>• Total number of AKI events<br>• Proportion of participants with hyperkalemia events (moderate hyperkalemia [5.5 <K+ ≤6.0 mmol/L], severe hyperkalemia [K+ >6.0 mmol/L])<br>• Total number of hyperkalemia events (moderate hyperkalemia [5.5 <K+ ≤6.0 mmol/L], severe hyperkalemia [K+ >6.0 mmol/L])<br>• Change from baseline in K+<br>• Proportion of participants with severe hypoglycemia events<br>• Total number of events of severe hypoglycemia events<br>• Proportion of participants with symptomatic hypotension events<br>• Total number of symptomatic hypotension events<br>• Proportion of participants with genital mycotic events<br>• Total number of genital mycotic events<br>• Proportion of participants with ketoacidosis events<br>• Total number of ketoacidosis events<br>• Proportion of participants with necrotizing fasciitis of the

Objectives	Endpoints
	perineum events<br>• Total number of necrotizing fasciitis of the perineum events<br>• Proportion of participants with urosepsis and pyelonephritis events<br>• Total number of urosepsis and pyelonephritis events
**Other exploratory**	
• To further investigate the treatment (finerenone, empagliflozin) and similar drugs (e.g., mode-of-action-related effects, safety) and to further investigate pathomechanisms deemed relevant to CV disease, CKD, diabetes, and associated health problems	• Various biomarkers (e.g., diagnostic, safety, pharmacodynamic, monitoring, or potentially predictive biomarkers)
**Other pre-specified**	
To characterize the pharmacokinetic (PK) of finerenone and empagliflozin when given in combination	• PK of finerenone and empagliflozin in plasma ($C_{max,md}$, $AUC_{\tau,md}$)
Abbreviations: AKI = acute kidney injury; $AUC_{\tau,md}$ = area under the concentration vs. time curve for the expected dosing interval obtained after multiple dose administration; $C_{max,md}$ = maximum drug concentration after multiple dose administration; CV = cardiovascular; CKD = chronic kidney disease; eGFR = estimated glomerular filtration rate; K+ = serum/plasma potassium; PK = pharmacokinetics; UACR = urinary albumin-to-creatinine ratio.	

There are 2 primary endpoints to address the objective of the study: relative change from baseline in UACR at 180 days in combination therapy group versus empagliflozin alone and relative change from baseline in UACR at 180 days in combination therapy group versus finerenone alone. These 2 primary endpoints are not considered as co-primary endpoints.

Primary analysis estimand

The assessment of efficacy will be done in the estimand framework, where the primary objective of the study is to assess the effect of combination therapy on the percentage reduction in UACR from baseline to Day 180 compared to its individual components if the treatment is taken according to the instructions as given in the protocol.

The estimand for assessing this study objective is defined by the following characteristics:

- *Population*: Adult participants with a clinical diagnosis of CKD and T2D.
- *Variable*: Change from baseline to Day 180 in UACR for the combination therapy group, to empagliflozin alone or finerenone alone.
- *Treatment*: Either finerenone (10 or 20 mg OD) and empagliflozin (10 mg OD), or finerenone alone (10 or 20 mg OD), or empagliflozin alone (10 mg OD). All treatments are administered in addition to standard of care (SoC).
- *Summary measure*: Mean ratio of change from baseline to Day 180 in UACR for the combination therapy group, to empagliflozin alone or finerenone alone.
- *Intercurrent events*:

Treatment discontinuation

- Treatment interruption due to reasons related to the treatment, such as hyperkalemia, will be handled using *treatment policy*

Dialysis/kidney transplantation

- Dialysis will be handled using a *hypothetical strategy*
- Kidney transplantation will be handled using a *hypothetical strategy*

Death

- Death will be handled using *a hypothetical strategy*.

Subsequent assessments after the intercurrent event of treatment discontinuation due to reasons related to the treatment will be collected and included within the primary analysis through a treatment policy approach.

Subsequent assessments after the intercurrent events of dialysis/kidney transplantation, or death will not be collected and the results will be estimated under the hypothetical strategy that the event did not occur and that the patient had continued in the study under the mixed model repeated measures (MMRM) statistical model. Further information on this approach is described in Section 4.9.2.

Sensitivity analyses will be performed to observe scenarios in which the imputation for the intercurrent events of dialysis/kidney transplantation or death are handled differently, through separate multiple imputation and tipping point analyses.

An additional sensitivity analysis will be performed where the additional intercurrent event of

Treatment discontinuation due to any reason

- Treatment interruption due to any reason, plus 30 days, regardless of relationship to study treatment

will be treated with a while on-treatment strategy, in addition to the 3 intercurrent events defined for the main analysis. Responses recorded 31 days or more after the last date of study treatment may be collected (depending on the patient's progress in the study) but will be excluded from the analysis.

A further sensitivity analysis will be performed where the additional intercurrent event of

Patient experienced an important deviation leading to exclusion from the Per-Protocol Analysis Set (PPS)

- *Patient experienced an important deviation which is deemed to interfere with the evaluation of efficacy data*

This intercurrent event is used to construct the PPS, and a principal stratum strategy will be used to exclude all patients with such a deviation from this sensitivity analysis. This will be in addition to the 3 intercurrent events defined for the main analysis.

In addition, the two intercurrent events of treatment discontinuation due to any reason and Patient experienced an important deviation leading to exclusion from the PPS will be combined, in addition to the 3 intercurrent events defined for the main analysis, in a further sensitivity analysis.

Further information on all sensitivity approaches is provided in Section 4.9.3.

1.2. Study Design

- Phase 2, randomized, controlled, double-blind (participants and investigators), double dummy, multicenter study in participants with CKD and T2D.
- Assuming a screening failure rate of approximately 50%, 1,614 participants will have to be screened to achieve 807 randomly assigned participants to treatment (approximately 269 participants per group).
- The study will consist of 2 consecutive parts:
  - Part A: Participants will be recruited if their eGFR is between 40 and 90 ml/min/1.73 m2, and they will be equipped with an ambulatory blood pressure monitoring (ABPM) at Visit 2 for a duration of 24 hours. An interactive web response system (IWRS) will allow capping the number of participants as follows:
    - 80% with an eGFR between ≤75 ml/min/1.73 m^2
    - 20% with an eGFR between >75 ml/min/1.73 m^2.
  - Part B: Participants will be recruited if their eGFR is between 30 and 90 ml/min/1.73 m^2, and they will not have an ABPM. The IWRS will allow capping the number of participants as follows:
    - 80% with an eGFR between ≤75 ml/min/1.73 m^2
    - 20% with an eGFR between >75 ml/min/1.73 m^2.
  - The decision to move from Part A to Part B will be taken by the sponsor and the study's steering committee (SC) upon feedback from the DMC. The safety analysis from the first 50 participants in part A, as well as their unblinded review by the independent DMC will be used to confirm the enrollment/recruitment start for Part B. This decision shall be effective immediately or after (institutional review board/independent ethics committees) IRB/IEC and/or local health authority approval, where applicable.
  - Other inclusion/exclusion criteria or study's schedule or procedure should not be affected.
- Participants will be randomized in a 1:1:1 ratio stratified by eGFR at screening (<60, ≥60 mL/min/1.73m^2) and UACR (≤850 mg/g, >850 mg/g), using the baseline median from FIDELIO-DKD study) in one of the 3 parallel groups:
  - Finerenone (10 or 20 mg once daily [OD]) and empagliflozin (10 mg OD)
  - Finerenone (10 or 20 mg OD) and matching placebo to empagliflozin (OD)
  - Empagliflozin (10 mg OD) and matching placebo to finerenone (OD).
  - The starting dose of finerenone will depend on the participant's eGFR level at the screening visit: a lower dose of 10 mg OD if eGFR is between <60 mL/min/1.73m^2, or the higher (target) dose of 20 mg OD if eGFR is ≥60 mL/min/1.73m^2.
  - Finerenone dose will be defined and further adjusted based on K+ and eGFR values obtained from local laboratories, at each study visit. Necessary blood samples may be obtained up to 72 hours before a scheduled visit. Finerenone

dose will also be adjusted, at investigator discretion based on any safety and tolerability concern.

- Participants should be treated for CKD and T2D according to local treatment guidelines. However, participants must not be exposed to an SGLT2i and/or a MRA within at least 8 weeks prior to screening. Participants should also be treated with the clinically maximum tolerated dose, as per investigator judgment, of angiotensin-converting enzyme inhibitor (ACEi) or angiotensin receptor blocker (ARB), but not both, for more than 1 month at screening visit.
- A DMC will review safety data during the study.
- The total study duration for each participant will be approximately 7.5 months (up to 8.5 months if the optional pre-screening is performed).
    - A screening visit will occur up to 2 weeks before randomization, during which eligibility criteria will be checked. Eligibility criteria related to laboratory evaluation (e.g., UACR) will be assessed once results are available. Note: Sites will also have the option of a pre-screening visit to examine UACR. This UACR value will not be valid to determine eligibility and will only be used to assess whether the participant may progress to a formal screening visit.
    - Randomization will occur at Day 1 (baseline). Following visits will occur at Days 14 (±2), 30 (±4), 90 (±5), and 180 (±5; last day of intervention period). A follow-up/end of study (EOS) visit will be scheduled, 30 days after last dose (Day 210 ±5).
    - Note: Following-titration or restart of study drug after interruption of finerenone intake for more than 7 days, the investigator will have to perform an unscheduled visit, 4 weeks (±7 days) after titration or restart, in order to monitor K+ levels and eGFR.
- The period between the participant's last intake of treatment and last visit in the study is referred to as the 'follow-up period'. If a participant withdraws from treatment permanently but does not withdraw from the study, this would apply to the period between the early discontinuation (ED) visit, which should take place as soon as possible following permanent discontinuation of treatment, and the follow-up/EOS visit. In this case, the follow-up period will also last approximately 30 days.
- It is planned that all randomized participants will remain in the study unless one of the following occurs: consent withdrawal, ED of the study by the sponsor.

The general study design as applied to this study is shown in Figure1. There is a pre-screening period and screening period, an intervention period and a safety follow-up period.

Figure1 Overall study design

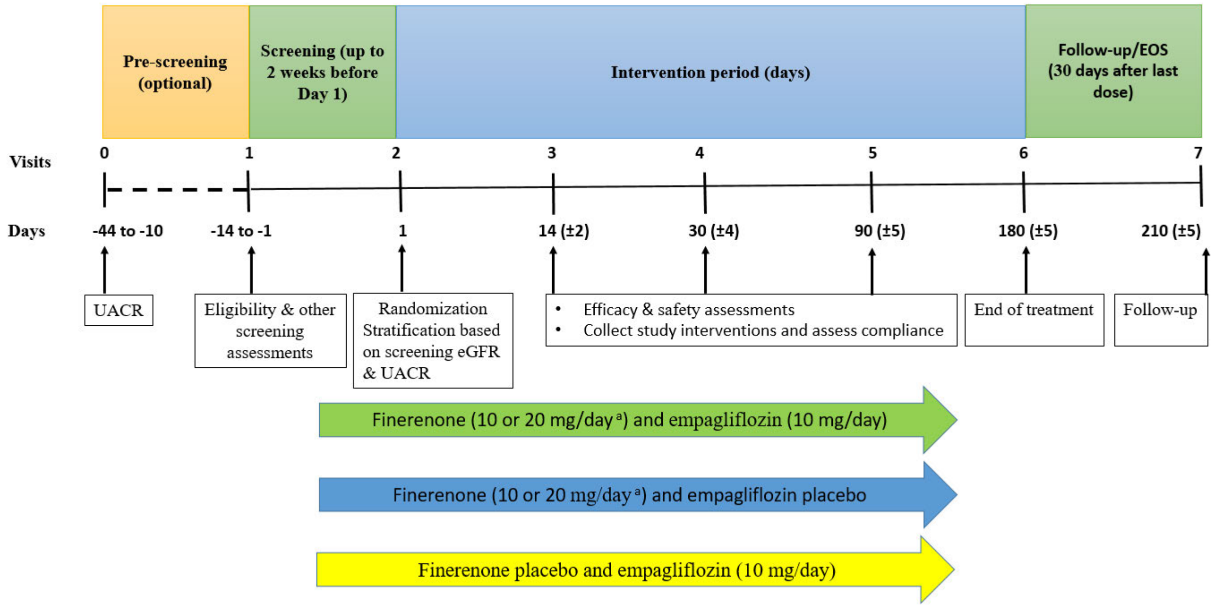

Abbreviations: eGFR = estimated glomerular filtration rate; EOS = end of study; UACR = urinary albumin-to-creatinine ratio.

[a] Up-/down-titration based on eGFR, serum/plasma potassium or potassium, safety, and tolerability.

2. Statistical Hypotheses

The primary analysis population for analyses of the primary endpoints will be the full analysis set (FAS). Define μ_i as the relative change (ratio) from baseline to 180 days in UACR for treatment group i, where i = Comb (finerenone and empagliflozin), Emp (empagliflozin), or Fin (finerenone).

The multiple primary endpoints are:

- Relative change in UACR from baseline at 180 days in combination (finerenone and empagliflozin) versus empagliflozin alone
- Relative change in UACR from baseline at 180 days in combination (finerenone and empagliflozin) versus finerenone alone.

In order to evaluate whether the combination of finerenone and empagliflozin is superior in reducing UACR than either empagliflozin or finerenone alone, we will analyze this using a repeated measures mixed model. However, to be conservative in our estimations we will apply a 2-sided 2-sample t-test of equal variance at an overall two-sided significance level of alpha=0.05. To adjust for the multiple testing of two hypotheses, the Bonferroni-Holm method will be applied. The 2 two-sided p-values are first ordered increasingly. If there is a positive treatment effect on the combination of finerenone and empagliflozin versus treatment i, and lower p-value $P_i<0.025$, the corresponding null hypothesis can be rejected. Thereafter, if there is also a positive treatment effect on the combination of finerenone and empagliflozin versus treatment j, and $P_j<0.05$, the second null hypothesis can also be rejected.

The primary hypotheses to be tested for the primary endpoints are provided below:

- $H0_{P1}$: $\mu_{Comb} - \mu_{Emp} = 0$
- Ha_{P1}: $\mu_{Comb} - \mu_{Emp} \neq 0$
- $H0_{P2}$: $\mu_{Comb} - \mu_{Fin} = 0$
- Ha_{P2}: $\mu_{Comb} - \mu_{Fin} \neq 0$

With two-sided overall significance level = 0.05 (0.025 for the hypothesis associated with the lowest p-value).

2.1. Multiplicity Adjustment

Adjustment for multiplicity are discussed as in Section 2.

3. Analysis Sets

For the purpose of analysis, the following analysis sets are defined:

**Participant Analysis Set**	**Description**
Listing Only Set (LOS)	All other participants screened who did not receive any dose of treatment or for whom no data after beginning of treatment are available will be classified as LOS. Their data will be presented in the individual participant data listings but will not be included in any statistical analysis.
Safety Analysis Set (SAF)	All randomized participants who have taken at least 1 dose of treatment. All participants will be analyzed according to the actual treatment received. For the non-combination therapy, i.e., finerenone, or empagliflozin alone, if a participant receives both treatments due to a bottle error, the treatment received for the majority of the time in the study will be used.<br><br>Patients with GCP violation of SMO at site 20003 in Japan will be excluded from the FAS.
Full Analysis Set (FAS)	All randomized participants, excluding mis-randomized participants who had not taken at least 1 dose of treatment. Mis-randomized participants who had taken at least 1 dose of treatment will be included in the FAS.<br><br>Patients with GCP violation of SMO at site 20003 in Japan will be excluded from the FAS.<br><br>All participants will be analyzed according to the planned treatment (the intent-to-treat principle). For the non-combination therapy i.e., finerenone, or empagliflozin alone, in the unlikely event that a participant receives both treatments due to a bottle error, the participant will be analyzed according to the planned treatment.

Participant Analysis Set	Description
Per-Protocol Analysis Set (PPS)	All participants of the FAS without any important protocol deviations which would interfere with the evaluation of the efficacy data. All participants will be analyzed according to the actual treatment(s) received.<br>Protocol deviations are defined as any change, divergence, or departure from the study design or procedures defined in the study protocol. Important protocol deviations are a subset of protocol deviations which may significantly impact the correctness, accuracy, and / or reliability of the study data or that may significantly affect a patient's rights, safety, or well-being.<br>Criteria which require clinical or medical monitoring interpretation will be reviewed prior to database lock. All important protocol deviations leading to exclusion from the PPS occurring during the study will be reviewed and approved by Bayer prior to database lock and identified before data are unblinded (if blinded study).
Pharmacokinetic Analysis Set	All randomized participants receiving at least 1 dose of treatment and have:<br>1. at least 1 quantifiable concentration, and<br>2. no protocol deviation that would interfere with PK data evaluation.

The validity of patients for allocation to various analysis sets will be assessed in an ongoing manner in protocol deviation meetings during the duration of the study and decisions will be documented in the blind review report prior to unblinding (potential conditional validity for PPS and pharmacokinetic analysis set).

Final decisions regarding the assignment of participants to analysis sets will be made during the review of study data and documented in the final list of important deviations, validity findings and assignment to analysis set(s).

4. Statistical Analyses

4.1. General Considerations

The statistical evaluation will be performed by using the statistical analysis software (SAS) (release 9.4 or higher; SAS Institute Inc., Cary, NC, USA).

All data will be presented in the participant data listing as they are recorded on the CRF, i.e. partially missing data will appear as such.

The analysis will be based on the global standard tables (Version 4.1 or higher) and the clinical pharmacology standards (CLIPS) (Version 3.0 or higher) where appropriate.

The FAS is used to analyze endpoints related to the efficacy objectives and the SAF is used to analyze the endpoints and assessments related to safety.

A log-normal distribution is assumed for serum creatinine, UACR and PK parameters. For all other metric variables, a normal distribution is assumed. The distributional assumptions will be investigated and if necessary, nonparametric methods or transformation of the data will be considered.

All variables will be analyzed by descriptive statistical methods. The number of data available, missing data (if applicable), mean, standard deviation (SD), minimum, lower quartile, median, upper quartile, and maximum will be calculated for metric data. The geometric mean and the SD of the log-transformed data will be provided instead of the arithmetic mean and SD for the variables where log-normal distributions are assumed. Frequency tables will be generated for categorical data.

The laboratory parameter eGFR will be calculated based on the CKD Epidemiology Collaboration (CKD-EPI) formula Version 2009 [1] for all analyses specified in this SAP.

The stratified analyses mentioned in this SAP will be conducted in consideration of the randomization stratification factors, eGFR and UACR category, unless specified otherwise. The strata variables eGFR and UCAR category used in the statistical analysis will be based on the screening UACR and eGFR assessments (as recorded from the vendor dataset). All participants will be analyzed according to their correct stratification category.

In case of stratification errors, the primary analysis will also be repeated based on the stratification category used in the randomization as a sensitivity analysis.

4.2. COVID-19 Pandemic Related Data

Coronavirus Disease 2019 (COVID-19) pandemic related study disruptions will be defined as any of the following:

- Important COVID-19 pandemic related protocol deviations (important deviations)
- Other COVID-19 pandemic related disruptions, such as missed visits, missed assessments/procedures (if not yet covered as important deviations)
- COVID-19 pandemic related premature discontinuations
- COVID-19 pandemic related dose modifications
- COVID-19 adverse events (AEs), i.e. any AEs which are covered in the Standard MedDRA Query (SMQ) “COVID-19” (narrow search)

- Violation of COVID-19 pandemic related inclusion/exclusion criteria (if any, study specific, i.e. needs to be described in the SAP)

Note, COVID-19 vaccinations will not be considered as COVID-19 pandemic related study disruptions.

The onset of COVID-19 pandemic date will use the onset of the COVID-19 pandemic in the study, as the earliest date when a participant in the study was affected by COVID-19 pandemic related study disruption.

Any COVID-19 pandemic related data points reported on the CRF should be displayed as part of the usual data listings [clinical study report (CSR) section 16.2] and might also be reported as part of the usual summary tables (CSR section 14). The final decision should be made by the project and/or study teams, respectively, and should be documented in the Tables, Listing and Figures specifications.

4.3. Handling of Dropouts

A participant who has been randomized and discontinues study participation prematurely for any reason, either from treatment or from follow-up, is defined as a “dropout”, dropouts also include participants who had not taken any treatment. Dropouts will not be replaced.

Data from participants who prematurely terminated the study will be used to the maximum extent possible.

The number of participants discontinuing the epochs, together with the primary reason for discontinuation, will be summarized as described in section 4.8.1.

4.4. Handling of Missing Data

All missing or partial data will be presented in the participant data listing as they are recorded on the CRF.

General rules

When appropriate, the following rules will be implemented so as not to exclude participants or observations from statistical analyses due to missing or incomplete data.

Concomitant medications with missing start and stop date but flagged as being ongoing at end of study will be considered to have started prior to study medication start and end after stop of study medication. The start and end reference period will be imputed as “before” for the medication start and as “during/after” for the medication end.

In case of (partially) missing dates for interruptions or permanent stop of study medication intake, a ‘worst-case’ approach will be applied to impute the start and end dates of study medication intake as the minimum and maximal possible dates, i.e.:

- first exposure to study medication date for a partially missing start date, and
- last month of the year, or last day of the month for a partially missing end date.

If a participant died earlier than the imputed worst study medication end date, the death date will be taken as the study medication end date. However, if these imputations lead to a temporal overlap between different exposure date records, the imputed dates will be adjusted so that no overlap exists and the time on the higher dose is maximized. The date of first exposure to treatment is not expected to be missing as the patients are instructed by the

investigator to take their first dose of study drug directly at Visit 1, but in the very rare case that this date is not recorded, it will be imputed according to the rules outlined above for missing start dates, but not earlier than the randomization date.

When only partial dates are available for the intercurrent events (treatment discontinuation, dialysis/kidney transplantation, death) in the efficacy analysis, a median imputation rule will be used:

- For example if the day is missing and the month is July, then day 16 is chosen.
- If the number of potential values is even, the lower of the 2 middle numbers is taken. For example, if the day is missing and the month is June, then day 15 is imputed. The same rule applies if the day and month are missing, e.g. if the year is 2017 and the day and month are missing, 2nd July is taken.
- In case the range of possible values is further restricted, e.g. because a patient died in the month in which the day is missing, the median in the restricted set of possible values is calculated. For example, if the clinical event occurred in June 2017 and the respective patient died on 11th June 2017, 6th June 2017 is imputed as the date of the clinical event.
  The same principle also applies to partially missing death dates, e.g. if only the year of death is available, but there is a last contact in the given year, then the date of death is imputed as the median of this date and the end of the given year.

A worst case approach will be applied for determining whether an AE with partially missing dates is treatment-emergent or not, i.e. if it is possible that the AE start date is within a period of study drug intake +3 days, then the AE is considered treatment-emergent.

If intensity of the AE is missing, the event will be considered as severe. If the same event is reported as both unrelated and related to the study drug within a participant, the event will be reported as related to study drug. If the drug relationship is missing, the event will be considered as being related to the study drug.

In case a death date is completely missing, it will be imputed on the basis of the last known contact when the participant was still alive and the first known contact when the participant was dead (e.g. from the participant health status follow-up page) as the median of these two dates. As above, if the number of potential values is even, the lower of the two middle numbers is taken.

In case both a non-fatal clinical event and death have partially missing dates, then death takes precedence and will be imputed first according to the rules outlined above. This also applies for non-CV death.

4.5. Interim Analyses and Data Monitoring

No interim analysis is planned for this study.

A detailed plan for the routine DMC safety analyses is covered in the DMC charter, the analysis planned to be provided to the DMC is included as part of the SAP and corresponding data display templates. The DMC will review the data in an unblinded manner. The DMC can recommend stopping the trial or modifying the trial design if there are

significant concerns regarding the safety profile of the investigational product. If unexpected safety issues are identified, specific amendments will be implemented based on the recommendation of the DMC. There are no predefined stopping conditions for the ongoing safety monitoring of this trial. The statistical analysis for the DMC meetings will be performed by an independent statistical analysis center.

4.6. Data Rules

General data rules are described in this section, further data rules for specific parameters or analyses are specified in the respective subsections of section 4.

4.6.1. Baseline values

Baseline values will be defined as the last non-missing measurement before or on the day of randomization (baseline visit, Day 1).

If the last observation available prior to randomization is the measurement from the screening visit, this would be used as the baseline value. This also includes assessments from a local laboratory, in case that prior to randomization, no assessment from the central laboratory is available. Otherwise, baseline will be missing.

If more than one measurement was planned for a scheduled visit used for the baseline visit (Day 1), for example BP measurements and heart rate, the mean value of these measurements per time point prior to randomization will be used as the baseline value.

4.6.2. Change from baseline

Change from baseline will in general be displayed as absolute change from baseline defined as the difference to baseline, i.e.:

Absolute change = post baseline value – baseline value.

Some parameters will be additionally analyzed as relative change defined as:

Relative change = 100 * [(post baseline value – baseline value) / baseline value].

For specific analyses, the relative decrease of a variable will be analyzed instead of the relative change. The relative decrease is equivalent to the negative of the relative change and defined as:

Relative decrease = 100 * [(baseline value – post baseline value) / baseline value].

4.6.3. Other data handling

Only the data provided by the central laboratory will be used for analysis, values from local laboratories will not be used in the statistical analysis and will be listed only unless there are no values from the central laboratory available for baseline.

At all visits starting at Visit 1 (Day 1) and if not stated otherwise, only the values at scheduled visits will be used for analysis. In the unexpected case that more measurements are collected at different timepoints than planned for a scheduled visit, only the first valid value will be used. For a scheduled visit, if there are several measurements planned for one variable, the mean value of these measurements will be documented for this visit.

For the derived visit “any time post-baseline” (applicable for efficacy) this will include any measurement after baseline, including unscheduled assessments. For the derived visit “any treatment-emergent” (applicable for safety), only assessments on or after study medication start date until 3 days after the date of any temporary or permanent interruption of study drug, including unscheduled assessments, will be considered.

For those laboratory values which are < lower limit of quantification (LLOQ), half the value of the LLOQ will be used for analysis. Differences between 2 values <LLOQ will be assigned values of 0. Ratios between 2 values <LLOQ will be assigned a value of 1. For values which are > upper limit of quantification (ULOQ), the ULOQ will be used for analysis. This rule does not apply to the finerenone plasma concentration data.

In case of log-normally distributed data, descriptive statistics other than minimum, maximum and median will only be calculated if at least 2/3 of the individual data were measured and were above the lower limit of quantification. In tables showing descriptive statistics, where values below LLOQ are included, these descriptive statistics will be marked.

Urinalysis is assessed 2 times per visit (except at screening). UACR will be determined 2 times at each visit from first morning void urine samples collected on 3 consecutive days. For the analyses of UACR, the 2 measurements at one visit will be combined provided that at least 2 measurements are available. First, the coefficient of variation will be calculated for the 3 values as follows considering the log-normal distribution for UACR [2]:

$$Coefficient\ of\ Variation = \sqrt{exp\left({SD_{ln}}^2\right) - 1},$$

where SD_{ln} is the standard deviation of the 3 log-transformed UACR values. If the coefficient of variation exceeds 25%, the median from the measurements will be used for the analyses. The median in case of an even number of values will be defined as the geometric mean from the 2 middle values. If the coefficient of variation is 25% at the most, the geometric mean will be used. If 2 or 3 scheduled assessments are missing or invalid and unscheduled assessments have been performed, then the set of unscheduled measurements closest to the planned visit will be used to determine the UACR at the respective visit if performed within 30 days before or after the planned time point for all visits from Visit 6 (180 days) onwards (with the following exception: only samples with 2/3 of values no later than 30 days (for “last on-treatment” analysis) or 3 days (for “last treatment-emergent” analysis) after last study drug intake will be considered). For Visit 1, 2 out of 3 samples have to be on or before the day of randomization and have to lead to non-missing values in order for the set of samples to be valid for baseline calculation; otherwise (also including the case when there is one valid sample at Visit 1), the set of screening samples will be considered for baseline.

4.6.4. GCP Violation by SMO at Site 20003 in Japan

Please refer to the details as found in Appendix 3. As a result of a GCP violation of SMO at site 20003 in Japan, these patient data have to be removed from the SAF, FAS and PPS counts. Details of this decision is documented in a file note filed in the eTMF.

4.7. Blind Review

The results of the blind review meeting will be documented in the blind review report and may comprise decisions and details relevant for statistical evaluation. Any changes to the

statistical analysis prompted by the results of the blinded review meeting will be documented in an amendment and, if applicable, in a supplement to this SAP.

4.8. Population Characteristics

4.8.1. Disposition

The number of participants screened, screen failed, enrolled, randomized, and valid for the SAF, FAS, PPS will be summarized overall and by treatment group, country, and site. This will be similarly summarized for the randomized participants affected by the COVID-19 pandemic.

The number of participants discontinuing the treatment and follow-up epochs, together with the primary reason for discontinuation, will be presented by treatment group and overall, in separate tables. In addition, the number of participants with important protocol deviations and validity findings will be presented overall, by investigator and country for each treatment group, and in total. The frequencies of each important protocol deviation and validity finding will be presented by treatment group and in total. Important protocol deviations associated with COVID-19 pandemic will be summarized by trial unit for the randomized participants.

4.8.2. Demography and other baseline characteristics

Demography includes age, sex, race, ethnicity, region (North America, Europe, and Asia), body weight (kg), body height (cm), BMI (body mass index) (kg/m^2), smoking history, caffeine, alcohol consumption (abstinent, light, moderate, heavy), and Kidney Disease: Improving Global Outcomes (KDIGO) risk factors (prognosis of CKD by GFR and albuminuria categories). Other baseline characteristics include baseline UACR, K+, categories for K+ (≤4.5 mmol and >4.5 mmol), eGFR (calculated by CKD-EPI formula), serum creatinine, glycated hemoglobin (HbA1c), and values for vital signs parameters (i.e., systolic blood pressure (SBP), diastolic blood pressure (DBP), and pulse rate).

All demographic data and baseline characteristics will be tabulated by treatment group and overall. The demographic and other baseline characteristics table will also be presented, separated by each level of the stratification factors, UACR category and eGFR category.

The non-stratified demographic and other baseline characteristics table will be repeated for all other analysis sets (SAF, PPS) if they differ in sample size from the FAS.

Demographics and other baseline characteristics will be presented for the PPS by treatment group and overall.

4.8.3. Medical History

Medical history will be coded using the medical dictionary for regulatory activities (MedDRA) dictionary. Medical history will be presented for each MedDRA primary system organ class (SOC) and preferred term (PT) by treatment group and overall, in a summary table for the FAS. Additional medical history terms by standardized MedDRA queries (SMQ) provided by the Bayer team during the study will also be presented for the FAS.

4.8.4. Prior and Concomitant Medications

Prior medications will not be defined nor presented.

Concomitant medication will be coded using the World Health Organization drug dictionary (WHO-DD).

The number of participants:

- who took at least 1 concomitant medication,
- who took at least 1 concomitant medication that started after start of treatment,
- who took at least 1 concomitant medication that started and ended before administration of treatment,
- who took at least 1 concomitant medication ongoing at baseline (i.e. starting before or on the day randomization and ending at least one day after the day of randomization)

will be presented by treatment group and overall using anatomical therapeutic chemical (ATC) classes and subclasses. A participant will be counted only once within each ATC class/subclass or treatment group, respectively.

The concomitant medication tables will be summarized for FAS.

The concomitant medication tables will be repeated summarizing the number of participants with medications in the Standard or Bayer Drug Groupings of interest.

Non-anti-diabetic concomitant medications of interest:

- ACEIs
- ARBs
- Beta-blockers
- Diuretics
- K+ sparing diuretics
- K+ supplements
- Potassium lowering agents
- Alpha blocking agents
- Calcium channel blockers
- Centrally acting antihypertensives
- Strong CYP3A4 inhibitors
- Moderate CYP3A4 inhibitors
- Weak CYP3A4 inhibitors
- Unclassified CYP3A4 inhibitors
- Trimethoprim
- Trimethoprim-sulfamethoxazole

Anti-diabetic concomitant medications of interest:

- Metformin
- Insulin
- Insulin secretagogues
- SGLT-2 inhibitors

- Combined SGLT-1 and 2i
- GLP-1

A listing will be provided including all medication classified as a weak, moderate, or strong CYP3A4 inhibitor according to the sponsor drug groupings together with the respective classification information.

4.9. Primary Endpoints and Estimands Analysis

4.9.1. Definition of Endpoints

The primary efficacy endpoints are:

- Relative change from baseline in UACR at 180 days in combination (finerenone and empagliflozin) versus empagliflozin alone
- Relative change from baseline in UACR at 180 days in combination (finerenone and empagliflozin) versus finerenone alone.

The corresponding summary measures are:

- Mean ratio of change from baseline to Day 180 in UACR for the combination therapy group, to empagliflozin alone
- Mean ratio of change from baseline to Day 180 in UACR for the combination therapy group, to finerenone alone

4.9.2. Main Analytical Approach

In order to evaluate whether finerenone and empagliflozin is superior in reducing UACR than either empagliflozin or finerenone alone, a two-sided two-group t-test of equal means at a multiplicity adjusted (Bonferroni-Holm) significance level of 0.05/2 = 0.025 will be applied for the initial hypothesis test.

UACR during the study will be summarized descriptively by treatment group and visit including ratios to baseline. These analyses will be performed overall and separated by the stratification factors (UACR category and eGFR category).

The primary efficacy analysis assumes the handling of patients with missing data after treatment discontinuation (including due to dialysis/kidney transplantation or death). Values will be imputed based upon patients with available data through a multiple imputation procedure assuming missing at random (MAR) mechanism.

The log-transformed ratio of UACR to baseline at each visit up to 180 days will be analyzed for each of the 1000 imputed datasets by a MMRM with the factors treatment group, visit, treatment by visit interaction, factors for the 2 stratification levels, UACR category and eGFR category, UACR baseline value as covariate nested within UACR category, and log-transformed UACR baseline value by visit interaction. The results from each model will be recombined using Rubin’s rule to provide pairwise ratios between the finerenone plus empagliflozin, and the empagliflozin treatment group will be calculated and corresponding two-sided 95% CIs and p-values will be computed.

The following example SAS program code for multiple imputation and combining results using Rubin’s rule will be used:

```
/* The intermittent missing data will be imputed using
multiple imputation MCMC procedure */
PROC MI DATA=<dataset> OUT=<dataset2> NIMPUTE=1000
SEED=<seed>;
VAR visit strata1 strata2;
MCMC IMPUTE=monotone CHAIN=multiple;
BY trtpn;
RUN;

PROC MI DATA=<dataset2> OUT=<dataset3> NIMPUTE=1000
SEED=<seed2>
CLASS trtpn visit strata1 strata2;
VAR trtpn visit strata1 strata2;
MONOTONE REG (chg = trtpn visit trtpn*visit strata1 strata2
log(baseline) log(baseline)*visit;
RUN;

PROC MIANALYZE
DATA=<dataset3>;
MODELEFFECTS estimate;
STERR stderr;
ODS OUTPUT PARAMETERESTIMATES = <dataset4>;
RUN;
```

The same analysis will be performed between the finerenone plus empagliflozin, and the finerenone treatment group.

The primary analysis of the primary efficacy variable will be repeated in the PPS as a supportive analysis.

4.9.3. Sensitivity Analyses

MMRM

Sensitivity analyses will be performed to test the primary efficacy analyses without the use of multiple imputation but still using the assumption of MAR. The log-transformed ratio of UACR to baseline at each visit up to 180 days will be analyzed by a MMRM with the factors treatment group, visit, treatment by visit interaction, factors for the 2 stratification levels (UACR category and eGFR category), UACR baseline value as covariate nested within UACR category, and log-transformed UACR baseline value by visit interaction. Pairwise ratios between the finerenone plus empagliflozin, and the empagliflozin treatment group will be calculated and corresponding two-sided 95% confidence intervals (CIs), and p-values will be computed. The model uses a missing at random assumption therefore will assume that the patients missing values would have followed a similar trend to patients who still have information available at the respective timepoint.

The SAS procedure PROC MIXED will be used estimating covariance patterns within participants to adjust for the within participant variance. For each treatment group a separate covariance pattern will be estimated based on an unstructured covariance. In case of

convergence issues, an alternative covariance matrix will be used, with Toeplitz matrix as first choice alternative.

Patients that experience kidney transplant/dialysis/death will be included in the analysis with all measures up to the date of the event. Patients that discontinue treatment will have all measures available included in the analysis.

The same analysis will be performed between the finerenone plus empagliflozin, and the finerenone treatment group.

To perform MMRM, SAS program code corresponding to the following will be used:

```
PROC MIXED DATA=<dataset>;
CLASS trtpn visit strata1 strata2 usubjid;
MODEL chg = trtpn visit trtpn*visit strata1 strata2
log(baseline) log(baseline)*visit;
REPEATED visit / SUBJECT = usubjid TYPE = UN;
LSMEANS trtpn*visit / PDIFF CL;
RUN;


/*
where
dataset = name of sub-dataset including all FAS subjects
randomized
chg = log-transformed ratio of UACR to baseline at each visit
up to 180 days
trtpn = planned treatment variable
visit = visit variable
strata1 = first stratification factor
strata2 = second stratification factor
baseline = UACR baseline
*/
```

Multiple imputation using missing not at random (MNAR)

The evaluation of the sensitivity analysis for the primary endpoints using MMRM (as described above) is relative change in UACR from baseline at 180 days in combination (finerenone and empagliflozin) versus empagliflozin alone and relative change in UACR from baseline at 180 days in combination (finerenone and empagliflozin) versus finerenone alone. This strategy leads to unbiased estimates of the treatment effect only if the missing data are “missing completely at random” (MCAR), i.e. the missingness is independent of both observed and unobserved outcomes. Although this condition might hold approximately, they are unlikely to hold exactly. In accordance with the European Medicines Agency (EMA) “Guideline on missing data in confirmatory clinical trials” other ways of handling missing data will be investigated.

The frequency, proportion and the reasons for dropouts will be tabulated within the main analysis. In addition, the frequency and proportion for time of dropout as well as for the

overall pattern of missing observations will be given. To investigate whether missingness seems to be “missing at random”, the mean UACR at baseline will be summarized for those with and without a post-baseline UACR. These analyses will be done for all patients grouped together and stratified by treatment. For the ratio of change to 180 days analysis, as UACR is only recorded at baseline and then at 180 days, the pattern of UACR over time up to 180 days cannot be systematically investigated further. However, if there are sufficient dropouts with UACR at 180 days, the ratio of change to from baseline to 180 days will be summarized by those still on study treatment and those having terminated study treatment.

MNAR means that missingness depends both on observed and unobserved outcomes. This requires an explicit model for the patient’s statistical behavior after drop-out. A fixed increase above the group average for patients dropping out can be assumed, where the pattern and size of the increase is varied across several sensitivity analyses. This is sometimes called the delta method of sensitivity analysis in a pattern mixture framework.

The following scenarios of no penalty and fixed penalties after drop-out will be investigated, where different values are assigned to the treatment groups:

Penalties after drop-out (ratio baseline : 180 days), based on an expected ratio from baseline to 180 days on finerenone of 0.70.

- Placebo 0 / finerenone 0
- Placebo 0 / finerenone +0.15
- Placebo 0 / finerenone +0.30
- Placebo +0.15 / finerenone +0.15
- Placebo +0.15 / finerenone +0.30
- Placebo +0.30 / finerenone +0.30
- Tipping point analysis: Placebo 0 / finerenone +X, X is the value where the p-value of the difference between placebo and finerenone becomes 0.05 (two-sided).

To properly account for the incompleteness of the data, multiple imputation will be used to draw sets of completed data that will then be modified according to the scenarios given above. Multiple imputation will be done using SAS PROC MI using the following generic code, where stratification group are coded as dummy variables using the first factor in each stratification group as the respective reference groups:

```
PROC MI DATA=<dataset> OUT=<dataset2> NIMPUTE=1000 SEED=<seed> ;
CLASS trtpn visit strata1 strata2;
MONOTONE reg);
MNAR adjust (baseline visit / shift=0 adjustobs=(trtpn=1);
MNAR adjust (baseline visit / shift=0.15 adjustobs=(trtpn=1);
MNAR adjust (baseline visit / shift=0.30 adjustobs=(trtpn=1);
VAR trtpn visit strata1 strata2 baseline month6;
RUN;
```

After modifying the completed data sets according to the scenarios, the MMRM of the main analysis will be performed at day 180 for each completed data set. The results are then combined using SAS PROC MIANALYZE.

For each scenario, the ratio of treatment difference at day 180 from baseline will be given with a 95%-confidence interval and a p-value. In addition, the minimum and maximum of observed treatment differences over the imputed data sets will be presented.

‘On-treatment’ analysis

An ‘on-treatment’ analysis will be performed, including only visit values occurring while taking treatment or until 30 days after stop of treatment. The timing of the assessment (if recorded) and timing of the dose of treatment will not be considered for the ‘on-treatment’ analysis i.e. first treatment date recorded at visit 1 will be considered as on-treatment. This analysis will be performed in the FAS and PPS.

4.10. Secondary Endpoints Analysis

The secondary analysis endpoints are:

- Relative change in UACR between end of treatment visit and at 30 days after end of treatment visit
- Relative change in UACR between 30 days after end of treatment visit and baseline
- Relative change in UACR category (>30%, >40%, and >50%) at 180 days.
- Ratio of change from baseline in eGFR at 30 days
- eGFR decline greater than 30% at 30 days from baseline
- Ratio of change in eGFR at 180 days and 210 days from day 30
- Proportion of participants with of acute kidney injury (AKI) events
- Total number of AKI events
- Proportion of participants with hyperkalemia events (moderate hyperkalemia [5.5 <K+ ≤6.0 mmol/L], severe hyperkalemia [K+ >6.0 mmol/L])
- Total number of hyperkalemia events (moderate hyperkalemia [5.5 <K+ ≤6.0 mmol/L], severe hyperkalemia [K+ >6.0 mmol/L])
- Change from baseline in K+
- Proportion of participants with severe hypoglycemia events
- Total number of events of severe hypoglycemia events
- Proportion of participants with symptomatic hypotension events
- Total number of symptomatic hypotension events
- Proportion of participants with genital mycotic events
- Total number of genital mycotic events
- Proportion of participants with ketoacidosis events
- Total number of ketoacidosis events
- Proportion of participants with necrotizing fasciitis of the perineum events

- Total number of necrotizing fasciitis of the perineum events
- Proportion of participants with urosepsis and pyelonephritis events
- Total number of urosepsis and pyelonephritis events

4.10.1. Key Secondary Endpoints

The key secondary efficacy analysis endpoints are:

- Relative change in UACR between end of treatment visit and at 30 days after end of treatment visit
- Relative change in UACR between 30 days after end of treatment visit and baseline
- Relative change in UACR category (>30%, >40%, and >50%) at 180 days.

4.10.1.1. Main Analytical Approach

Frequency tables will be generated for the number of participants with a relative decrease and increase in UACR of >30%, >40%, and >50% from baseline UACR. This will be summarized for each visit and for any time post baseline, and will also be stratified for each level of the stratification factors (UACR category and eGFR category).

The additional categorical UACR efficacy variables will be summarized for presence or absence of the event (each of the relative decreases and increases in UACR of interest of >30%, >40%, and >50% from baseline UACR), using logistic regression with the factor's treatment group and stratification levels (UACR category and eGFR category). Pairwise differences between the combination versus empagliflozin or combination versus finerenone group will be calculated and corresponding two-sided 95% CIs will be computed. The treatment effect (odds ratio) along with its 95% CI and p-value will be estimated in the model, and presented alongside the proportions.

4.11. Exploratory Endpoints Analysis

Other exploratory objective of the study is:

- To further investigate the treatment (finerenone, empagliflozin) and similar drugs (e.g., mode-of-action-related effects, safety) and to further investigate pathomechanisms deemed relevant to CV disease, CKD, diabetes, and associated health problems

Other exploratory endpoint of the study is:

- Various biomarkers (e.g., diagnostic, safety, pharmacodynamics (PD), monitoring, or potentially predictive biomarkers)

These will be described in a separate protocol and SAP.

4.11.1. Pharmacodynamics

The following sample types will be collected for biomarker analysis:

- Blood (plasma/serum)
- Urine

The exact specimen type for a particular biomarker analysis (e.g., serum or plasma) will be provided in separate documents (e.g., sample handling sheets or lab manual).

Biomarker investigations may be reported separately (e.g., in a biomarker evaluation report) except for plasma renin activity, aldosterone, urinary sodium, and biomarkers.

4.12. Safety Analyses

All analyses on safety data will be performed in the SAF.

The following safety variables will be assessed during the study:

- AEs
- Laboratory data
- Electrocardiogram (ECG) data
- Vital signs, including weight and BMI
- Further safety variables:
    - Change from baseline in eGFR at 30 days
    - eGFR decline greater than 30% at Day 30 from baseline
    - Change from baseline in eGFR at 180 days
    - Change from baseline in eGFR at 210 days
    - Proportion of participants with AKI events
    - Total number of AKI events
    - Change from baseline in K+
    - Proportion of participants with hyperkalemia events
    - Total number of hyperkalemia events
    - Proportion of participants with moderate hyperkalemia events (K+ >5.5 to ≤ 6.0 mmol/L)
    - Total number of moderate hyperkalemia events (K+ >5.5 to ≤6.0 mmol/L)
    - Proportion of participants with severe hyperkalemia events (K+ >6.0 mmol/L)
    - Total number of severe hyperkalemia events (K+ >6.0 mmol/L)
    - Proportion of participants with severe hypoglycemia events
    - Total number of severe hypoglycemia events
    - Proportion of participants with symptomatic hypotension events
    - Total number of symptomatic hypotension events
    - Proportion of participants with genital mycotic events
    - Total number of genital mycotic events
    - Proportion of participants with ketoacidosis
    - Total number of ketoacidosis events

- Proportion of participants with necrotizing fasciitis of the perineum events
- Total number of necrotizing fasciitis of the perineum events.
- Proportion of participants with urosepsis and pyelonephritis events
- Total number of urosepsis and pyelonephritis events.

4.12.1. Extent of Exposure

The analyses described in this section will be repeated for the SAF and PPS if they differ in sample size from the FAS.

Treatment duration will be calculated using the last date of treatment minus the start date of treatment, excluding any treatment interruption. Treatment duration (number of days with treatment intake) will be summarized using descriptive statistics by treatment group and overall. In addition, treatment duration will be categorized to <1 month, 1 to <3 months, 3 to <6 months, and ≥6 months and presented with the corresponding number and percentage of participants by treatment group and overall.

Cumulative treatment duration will be calculated using the last date of treatment minus the start date of treatment, including any treatment interruption. Cumulative treatment duration will be categorized to <1 month, 1 to <3 months, 3 to <6 months, and ≥6 months.

A table will be presented with the absolute and relative frequencies of participants still on the study at each visit.

The extent of exposure to each treatment (total amount of intake in grams) during treatment, and patients with at least one dose modification will be summarized using descriptive statistics by treatment group, also by eGFR category by screening (<60, >=60 $mL/min/1.73m^2$).

The up-titration status (yes/no), regardless of actual or sham up-titration, will be summarized with absolute and relative frequencies per treatment group for each visit as well as participants never up-titrated, up-titrated once, and up-titrated more than once.

Compliance (as a percentage) will be calculated as follows:

- 100 * Number of taken tablets or capsules/Number of planned tablets or capsules.

The number of planned tablets or capsules will be calculated as follows:

- (Days from randomization to last intake of treatment + 1) * Number of planned tablets or capsules per day.

All tablets and capsules, including the dummy placebo tablets and capsules, will be counted.

For participants who withdraw prematurely from the treatment, compliance will be calculated up to the time of last dose.

The compliance will be summarized descriptively by treatment group and overall. In addition, percent of compliance will be categorized into 3 groups, <80%, ≥80% to ≤120%, and >120%, and the categories will be summarized by treatment group and overall.

4.12.2. Adverse Events

AEs will be coded using the MedDRA (latest version available prior to database freeze). A listing will be provided linking the original investigator terms and the coded terms. AEs will also be presented grouped by SMQs.

AEs that started or worsened after the first dose of treatment up to 3 days after any temporary or permanent interruption of treatment will be considered as treatment emergent adverse events (TEAEs).

To further characterize the safety of finerenone in combination with empagliflozin, symptomatic hypotension as well as AKI will be collected as treatment-emergent adverse events of special interest (AESIs).

An overall summary of all AEs, TEAEs and treatment-emergent AESIs will be generated by treatment group.

The number of participants reporting treatment-emergent hyperkalemia AEs; including drug-related, leading to hospitalization, leading to permanent discontinuation of study drug, SAE and leading to death will be summarized by treatment group and overall.

In order to assess whether there is bias from AE reporting during the COVID-19 pandemic, the number of events, the total time at risk (i.e. patient years at risk) and the event rate scaled to 100 patient-years (i.e. number of events / total time at risk * 100) based on the timeframe from onset of COVID-19 pandemic onwards and overall will be presented.

The number of participants, and number of events with TEAEs, post-treatment AEs occurring more than 3 days after stop of treatment, treatment-emergent SAEs, treatment-emergent treatment-related AEs, treatment-emergent treatment-related SAEs, treatment-emergent AESIs, AEs with fatal outcome, TEAEs with fatal outcome, post-treatment AEs with fatal outcomes, TEAEs causing permanent discontinuation of treatment, treatment-emergent non-serious AEs, non-serious AEs, TEAEs by maximum intensity, treatment-emergent SAEs by maximum intensity, treatment-related TEAEs by maximum intensity, treatment-emergent AESIs of maximum intensity, TEAEs by worst outcome, and treatment emergent SAEs by worst outcome, treatment-emergent AESIs by worst outcome will be summarized by treatment group using MedDRA terms grouped by primary SOC and PT.

To comply with local regulatory requirements in Japan, some cardiovascular disease-related events (DREs) will also be documented as (S)AEs in Japan. These will be included in the DRE tables (see section 4.12.2.1), and to avoid double-counting of such events, they will not be included in the AE summary tables or listings. A separate listing will be generated for all AEs excluded from the AE analysis due to double reporting in Japan.

In case of events with different intensity within a participant, the maximum reported intensity will be used. If intensity is missing, the event will be considered as severe. If the same event is reported as both unrelated and related to the treatment within a participant, the event will be reported as related to treatment. If the drug relationship is missing, the event will be considered as being related to the treatment.

Separate tables summarizing TEAEs, and treatment-emergent SAEs, treatment-emergent treatment-related AEs, that occurred in more than 5% of the participants will be provided by PT.

Deaths, SAEs, and AEs leading to permanent treatment discontinuation will be listed separately.

4.12.2.1. Disease-Related Events and/or Disease-Related Outcomes Not Qualifying for Expedited Reporting as AE or SAE

The following DREs are common in participants with CKD in T2D and can be serious/life-threatening:

- Kidney failure defined as:
  - End Stage Kidney Disease: Initiation of chronic dialysis (hemo- or peritoneal dialysis) for at least 30 days or renal transplantation.
  - Decrease of eGFR to less than 15 mL/min/1.73m^2, confirmed by at least one additional standardized measurement at least 4 weeks after the initial measurement.
- Renal death (see Appendix 5 in protocol for definition)
- Chronic sustained decrease in eGFR (see Appendix 3 in protocol for definition)
- CV death (see Appendix 5 in protocol for definition)
- Non-fatal stroke (see Appendix 3 in protocol for definition)
- Non-fatal myocardial infarction (MI) (see Appendix 3 in protocol for definition)
- Hospitalization for heart failure (HF) (see Appendix 3 in protocol for definition)
- New onset of HF (see Appendix 3 in protocol for definition).

Because these events are typically associated with the disease under study, they will not be reported according to the standard process for expedited reporting of SAEs even though the event may meet the definition of an SAE. These events will be recorded immediately of investigator's awareness. These DREs will be monitored by the DMC on a regular basis (see DMC Charter).

However, if either of the following conditions applies, then the event must be recorded and reported as an AE/SAE (instead of a DRE):

- The event is, in the investigator's opinion, of greater intensity, frequency, or duration than expected for the individual participant.

OR

- The investigator considers that there is a reasonable possibility that the event was related to treatment.

Note: These rules will not entirely apply to participants enrolled in Japan where the only events recorded as DREs will be kidney failure, renal death, and chronic sustained decrease in eGFR. CV death, non-fatal stroke, non-fatal MI, hospitalization for HF, and new onset of HF have to be reported as SAE for Japanese participants.

A summary of DREs will be generated by treatment group.

The number of participants with DREs, DREs by maximum intensity, DREs by worst outcome will be summarized by treatment group using MedDRA terms grouped by primary SOC and PT.

4.12.3. Additional Safety Assessments

4.12.3.1. Laboratory Data

The number of participants with treatment-emergent (until 3 days after any temporary or permanent interruption of treatment) abnormal laboratory values above or below the normal range will be tabulated by the laboratory parameter and treatment group.

Summary statistics including changes to baseline will be calculated by treatment group and visit for all quantitative laboratory parameters, e.g., for hematology, HbA1c, clinical chemistry, and urinalysis. Geometric statistics and ratios to baseline will be presented for creatinine and albumin instead of arithmetic statistics with changes from baseline. For eGFR, the relative change will be displayed in addition to the absolute change from baseline.

Boxplots for the absolute values, including the reference ranges for the lower and upper limits for K+, eGFR, serum creatinine and UACR will be produced by treatment group.

Summary statistics for K+, eGFR, and serum creatinine will also be repeated by treatment group and visit separately for each level of the stratification factors (UACR category and eGFR category).

Table 1 below details the tests performed at the times as indicated in the schedule of assessments in the Appendix 1.

Table 1: Protocol-Required Laboratory Tests

Category	Analyte	SI Unit
Hematology	Hemoglobin	g/L
Hematology	Hematocrit	1
Hematology	Red blood cell (RBC) Count	Tl/L
Hematology	Mean corpuscular volume (MCV)	fL
Hematology	Mean corpuscular hemoglobin (MCH)	Pg
Hematology	Mean corpuscular hemoglobin concentration (MCHC)	g/L
Hematology	White blood cell (WBC) Count	G/L
Hematology	Basophils	%
Hematology	Platelet count	Gl/L
Chemistry	Calcium	mmol/L
Chemistry	Magnesium	mmol/L
Chemistry	Phosphate	mmol/L
Chemistry	Sodium	mmol/L
Chemistry	Potassium	mmol/L
Chemistry	Blood Urea Nitrogen (BUN)	mmol/L
Chemistry	Creatinine Kinase	U/L
Chemistry	Creatinine	umol/L
Chemistry	Albumin	g/L
Chemistry	Total CO2	mmol/L
Chemistry	Total Protein	g/L
Chemistry	Bicarbonate	mmol/L
Chemistry	Glucose Fasting	mmol/L
Chemistry	Total Bilirubin	umol/L
Chemistry	Direct Bilirubin	umol/L

Category	Analyte	SI Unit
Chemistry	Aspartate aminotransferase serum glutamic –oxaloacetic transaminase [AST (SGOT)]	U/L
Chemistry	Alanine aminotransferase serum glutamic-pyruvic transaminase [ALT(SGPT)]	U/L
Chemistry	Gamma-glutamyl transferase (GGT)	U/L
Chemistry	Alkaline Phosphatase (ALP)	U/L
Chemistry	Lactate dehydrogenase (LDH)	lU/L
Chemistry	Follicle-stimulating hormone (FSH)	lU/L
Chemistry	High-density lipoprotein cholesterol (HDL-C)	mmol/L
Chemistry	Low-density lipoprotein cholesterol (LDL-C)	nmol/L
Chemistry	Cystatin C	mg/L
Chemistry	Total Cholesterol	mmol/L
Chemistry	HbA1c	%
Chemistry	Triglycerides	mmol/L
Chemistry	eGFRcr (CKD-EPI creatinine, Levey et al. 2009)	mL/min/1.73m2
Chemistry	eGFRcr-cys (CKD-EPI creatinine-cystatin C, Inker et al. 2021)	mL/min/1.73m2
Urinalysis	Potential of hydrogen (pH)	
Urinalysis	Protein	g/L
Pregnancy Testing	Highly sensitive serum human chorionic gonadotropin pregnancy test (as needed for women of childbearing potential) – Quantitative	mIU/mL
Pregnancy Testing	Highly sensitive serum human chorionic gonadotropin pregnancy test (as needed for women of childbearing potential) – Qualitative	
Urinalysis	Microalbumin	mg/L
Urinalysis	Ketones	
Urinalysis	Glucose	
Urinalysis	Blood	
Urinalysis	Microscopic	
Urinalysis	UACR	
Urinalysis	Creatinine Excretion (Urine Creatinine)	nmol/D

All study-required laboratory assessments will be performed by a central laboratory, with the exception of:

- Optional pre-screening UACR (local laboratory)
- K+ (both at central and local laboratories)
- estimated glomerular filtration rate (both at central and local laboratories)

A high FSH level in the postmenopausal range may be used to confirm a postmenopausal state in women not using hormonal contraception or hormonal replacement therapy. However, in the absence of 12 months of amenorrhea, confirmation with more than one FSH measurement is required.

4.12.3.2. Vital Signs

At the corresponding visits, 3 measurements of BP will be taken in sitting position with at least a 1-minute interval between each reading. Averages of non-missing values of these 3 measurements will be calculated and used for the statistical analysis. If only 1 of the planned measurements is available, this value will be used.

Vital signs values will be summarized by treatment group and visit using descriptive statistics including absolute changes from baseline. The analysis will be repeated for SBP stratified by baseline SBP >90 to <130 mmHg, 130 to <160 mmHg, and ≥160 mmHg.

The values and the changes from baseline will be summarized by treatment group and visit using descriptive statistics for weight and BMI.

Figures will be produced for each vital sign parameters mean and standard deviations over time, and changes from baseline values.

4.12.3.3. ABPM

Participants enrolled in Part A will be equipped for 24 hours with an ABPM device 1 hour before the first study intervention intake, at Day 1.

ABPM starts during the visit and finishes approximately 24 hours later on the following day.

The 24-hour profiles will be recorded at the following intervals:

- At 30-min intervals from 06:00 to <22:00 (daytime).
- At 60-min intervals from 22:00 to <06:00 (night time).

The ABPM parameters (SBP, DBP, heart rate, pulse rate, mean arterial pressure) will be summarized by treatment group using descriptive statistics for total, day time and night time intervals.

4.12.3.4. ECG

ECG parameter values (PR Interval, QRS Duration, QT Interval) will be summarized by treatment group and visit using descriptive statistics including absolute changes from baseline.

ECG findings and interpretation will be summarized by treatment group and visit using descriptive statistics.

4.12.3.5. Other Safety analysis

The following safety variables:

- Ratio of change from baseline in eGFR at 30 days
- Ratio of change in eGFR at 180 days and 210 days from baseline
- Ratio of change in eGFR at 180 days and 210 days from day 30
- Change from baseline in K+

will be analyzed as described for the primary endpoint.

As a sensitivity analysis, the eGFR endpoints will be repeated using the updated CKD-EPI formula published in 2021 (Inker et al. 2021).

The following further safety variables will be further assessed by displaying the percentage of participants with the respective treatment-emergent safety events by treatment group. This will also be performed by visit (except for eGFR decline greater than 30% at Day 30 from baseline) and by stratification factor, as required. The summaries will be performed for the number of participants with:

- eGFR decline greater than 30% at Day 30 from baseline
- Proportion of participants with and total number of AKI events

- Proportion of participants with and total number of hyperkalemia events
- Proportion of participants with and total number of moderate hyperkalemia events (K+>5.5 to ≤6.0 mmol/L)
- Proportion of participants with and total number of severe hyperkalemia events (K+>6.0 mmol/L)
- Proportion of participants with and total number of severe hypoglycemia events
- Proportion of participants with and total number of symptomatic hypotension events
- Proportion of participants with and total number of genital mycotic events
- Proportion of participants with and total number of ketoacidosis events
- Proportion of participants with and total number of necrotizing fasciitis of the perineum events
- Proportion of participants with and total number of urosepsis and pyelonephritis events

For each analysis, the percentage of participants with the respective events (non-stratified) at any time post-baseline (including unscheduled assessments) will be compared between the finerenone and empagliflozin, and the empagliflozin treatment group by applying separate explorative χ^2 tests with continuity correction. If the expected number of participants in at least 1 cell of the 3x2 contingency table is <5, Fisher's exact test will be applied instead of the χ^2 test. Estimates and 2-sided 95% CIs will be provided for each treatment group and the treatment differences. Clopper pearson CIs will be calculated for each treatment group, while for treatment differences the exact unconditional confidence limits will be calculated.

Similarly, this will be repeated for finerenone and empagliflozin, and the finerenone treatment group. The eGFR endpoints will be analyzed in the FAS population. As a sensitivity analysis, the analysis on eGFR endpoints will be repeated using the updated CKD-EPI formula published in 2021 (Inker et al. 2021).

```
proc freq data=<dataset>;
   tables trtan*Event /chisq riskdiffc alpha=0.95 expected;
   exact riskdiff;
run;
/*
where
dataset = name of sub-dataset including all SAF subjects
randomized
trtan = Actual treatment variable
*/
```

4.13. Other Analyses

4.13.1. Other Variables and Parameters

Other pre-specified endpoint of the study is:

- PK of finerenone and empagliflozin in plasma ($C_{max,md}$, $AUC_{\tau,md}$)

4.13.1.1. Pharmacokinetics

Finerenone and empagliflozin plasma concentrations might be analyzed in case of specific questions from the DMC, SC, or sponsor.

In the event of a decision to perform this analysis, the plasma concentrations of finerenone and empagliflozin will be determined at different time points using a sparse sampling approach in all participants. The plasma concentration versus time data collected will be evaluated descriptively, separated by dose and visit. Plots will be prepared of all individual plasma concentrations versus actual relative study times (time of sample collection after time of treatment administration).

PK and exposure-response analysis may be performed using population approaches (popPK and popPK/PD, e.g., by non-linear mixed effect modeling). Such evaluations will be described in a separate analysis plan and will be reported separately. Such evaluations may be started prior to database lock. If this is applicable, appropriate measures will be taken to maintain blinding of the study team, e.g., data will be stored separately, and members of the study team will neither have access to the randomization list nor to individual data.

Details about the collection, processing, storage and shipment of samples will be provided separately (e.g., sample handling sheets or laboratory manual).

4.13.2. Subgroup Analyses

Exploratory subgroup analyses are planned for the primary efficacy variable.

This will include descriptive statistics and a statistical test for interaction.

The following subgroups will be considered for exploratory subgroup analyses:

- Region (North America, Europe, and Asia)
- eGFR category at screening (eGFR 30 to <60, 60 to 75, >75 mL/min/1.73m^2)
- eGFR category at baseline (eGFR 30 to <60, 60 to 75, >75 mL/min/1.73m^2)
- History of cardiovascular disease (CVD) (present, absent)
- Baseline K+ (≤4.5 versus >4.5 mmol/L)
- SBP at baseline (>90 to <130, 130 to <160 mmHg, ≥160 mmHg)
- Sex
- Age group (<65 years, ≥65 years).

It is anticipated that in these proposed subgroups for analysis, differences in treatment effects may be observed according to the screening or baseline characteristics defined, due in part to the differences in the risk of clinical events expected in the different subgroups.

4.14. Changes to Protocol-planned Analyses

- Demography and other baseline characteristics mention that drugs, these are not collected in the eCRF and cannot be summarized.
- The PK of finerenone and empagliflozin in plasma ($C_{max,md}$, $AUC_{\tau,md}$) was specified as an optional analysis in the protocol, these are no longer optional as they may be requested by the SC.
- Subgroup analyses for the primary efficacy variable:
    - will be performed by SBP at baseline using the categories >90 to <130, 130 to <160 mmHg, as per protocol, but also by category ≥160 mmHg that was missed from the protocol in error.
    - will be performed by eGFR category at screening with eGFR 30 to <60, 60 to 75, and >75 ml/min/1.73m^2), instead of eGFR 40 to <60, 60 to 75 mL/min/1.73m^2 to cover under 40 and >75 mL/min/1.73m^2
    - will be performed by eGFR category at screening with eGFR 30 to <60, 60 to 75, and >75 ml/min/1.73m^2), instead of eGFR 40 to <60, 60 to 75 mL/min/1.73m^2 to cover under 40 and >75 mL/min/1.73m^2
    - Was indicated to be performed by race in the protocol, but this has been removed in the SAP.
- The FAS population has been updated to exclude any mis-randomized patients who had not taken at least 1 dose of treatment. The reason for exclusion is that these patients do not satisfy an entry criterion that was measured prior to randomization, and therefore did not receive any treatment. The FAS population will include any mis-randomized patients who had taken at least 1 dose of treatment.
- As a result of patients with GCP violations from Site 20003 in Japan, they are to be excluded from the SAF, FAS and PPS.
- KDIGO risk factors has been added to the demography section for summarizing.
- GLP-1 was not mentioned as a concomitant medication of interest.

5. Sample Size Determination

When testing the combination therapy versus finerenone alone, group sample sizes of 226 and 226 achieve 80% power to reject the null hypothesis of equal means when the log transformed population mean difference is $\ln(\mu 1) - \ln(\mu 2) = \ln(0.502 / 0.628) = -0.224$, with a SD for both groups of 0.77 and with a significance level (alpha) of 0.025 using a 2-sided 2-sample -equal variance t-test.

When testing the combination therapy versus SGLT2i alone group sample sizes of 228 and 228 achieve 80% power to reject the null hypothesis of equal means when the log transformed population mean difference is $\ln(\mu 1) - \ln(\mu 2) = \ln(0.56 / 0.7) = -0.223$, with a SD for both groups of 0.77 and with a significance level (alpha) of 0.025 using a 2-sided 2-sample -equal variance t-test.

There will be the same number of participants in each group, so for the combi therapy versus finerenone alone the power will be boosted to approximately 81% due to the extra participants included. Therefore, group sample sizes of 228, 228, and 228 will be sufficient.

Assuming a 15% drop out rate an extra 123 participants are required takes the total sample size to 807. Therefore, group sample sizes of 269, 269, and 269 will be sufficient to detect a 20% further reduction in UACR in the combination arm versus empagliflozin or finerenone.

6. Supporting Documentation

6.1. Appendix 1: Schedule of Assessments

Table 1-1: Schedule of Activities

Procedure	Pre-screening[a]	Screening (up to 2 weeks before Day 1)	Intervention Period [Days]					ED	Follow-up/EOS (30 days after last dose)	Notes
			1[b] (dose)	14±2	30±4	90±5	180±5 (EOT)		210±5	
**Visit**	0	1	2	3	4	5	6		7	
**Study Day**	-44 to -10	-14 to -6	1	12-16	26-34	85-95	175-185		205-215	
Pre-screening consent	X									
Informed consent		X								
Inclusion and exclusion criteria		X	X							Re-check clinical status before randomization
Demography		X								
Complete physical examination including height[c] and weight		X	X	X	X	X	X	X	X	
Vital signs		X	X	X	X	X	X	X	X	
Medical history		X								
Serum pregnancy test (WOCBP only)		X			X	X	X	X		To be performed by central lab
FSH test (to confirm postmenopausal status, when needed)[d]		X								
Central laboratory tests										
UACR (urinary creatinine, albumin)[e,f]		X[i]	X	X	X	X	X	X	X	
Serum K+[e]		X[i]	X	X	X	X	X	X	X	
eGFR (serum creatinine)[e]		X[i]	X	X	X	X	X	X	X	

Table 1-1: Schedule of Activities

Procedure	Pre-screening[a]	Screening (up to 2 weeks before Day 1)	Intervention Period [Days]					ED	Follow-up/EOS (30 days after last dose)	Notes
			1[b] (dose)	14±2	30±4	90±5	180±5 (EOT)		210±5	
**Visit**	**0**	**1**	**2**	**3**	**4**	**5**	**6**		**7**	
**Study Day**	**-44 to -10**	**-14 to -6**	**1**	**12-16**	**26-34**	**85-95**	**175-185**		**205-215**	
Safety laboratory tests		X	X	X	X	X	X	X	X	Clinical chemistry, hematology, urinalysis. HDL, LDL-C, total cholesterol and triglycerides will be measured at screening only
Local laboratory tests[g]										
UACR [f]	X									Urine containers to be supplied by local lab
Serum/plasma K+[h]			X[j]	X[k]	X[k]	X[k]	X[k]	X[k]	X[k]	At visit 2 in case the participant is deemed not eligible based on K+, retest is allowed within 24 hours
eGFR (serum creatinine)[h]			X[j]	X[k]	X[k]	X[k]	X[k]	X[k]	X[k]	At visit 2 in case the participant is deemed not eligible based on eGFR, retest is allowed within 24 hours
PK[l,m]				X	X	X	X	X		
Biomarkers (blood) [l]		X	X		X		X	X		Day 1 sample to be collected prior to start of treatment
Biomarker samples (first morning void urine)			X		X		X	X		
12-lead ECG		X	X				X			
Randomization			X							

Table 1-1: Schedule of Activities

Procedure	Pre-screening[a]	Screening (up to 2 weeks before Day 1)	Intervention Period [Days]					ED	Follow-up/EOS (30 days after last dose)	Notes
			1[b] (dose)	14±2	30±4	90±5	180±5 (EOT)		210±5	
**Visit**	0	1	2	3	4	5	6		7	
**Study Day**	-44 to -10	-14 to -6	1	12-16	26-34	85-95	175-185		205-215	
Dispense urine containers for UACR assessments		X	X	X	X	X	X			
Study intervention Dispensation/Accountability			X[n]		X	X	X[o]			
24-hour ambulatory blood pressure monitoring			X[n]							
Dose adjustment (with unscheduled visits)[p]					X	X				Up-/down-titration, restart after interruption
Adverse Events	X	X	←==→							
Prior/Concomitant medication review		X	←==→							

Abbreviations: ECG = electrocardiogram; ED = early discontinuation; EOS = end of study; EOT = end of treatment; eGFR = estimated glomerular filtration rate; HDL = high density lipoprotein; LDL-C = low density lipoprotein cholesterol; K+ = serum/plasma potassium; PK = pharmacokinetics; UACR = urinary albumin-to-creatinine ratio; WOCBP = woman of childbearing potential.

a) Pre-screening visit is optional (up to 4 weeks before screening).
b) Day 1 = randomization/baseline visit.
c) Height will be measured at screening only.
d) May be used to confirm a postmenopausal state in women not using hormonal contraception or HRT. In the absence of 12 months of amenorrhea, confirmation with more than one FSH measurement is required.
e) Central laboratory values to be used for endpoint statistical analyses.
f) At the optional pre-screening and at Visit 1, first morning void urine samples to be collected on 3 consecutive days at the participant’s home. At the

other visits, the 2 consecutive samples can be collected ±7 days from the visit date.

g) Local laboratory values for safety purposes.
h) Blood samples (for K+ and creatinine [eGFR]) for measurement in the local laboratory may be obtained up to 72 hours before a scheduled visit.
i) One re-assessment (central lab) of eGFR, K+ and/or UACR is allowed at the screening visit.
j) K+, and eGFR values obtained from local laboratory will be used to randomize participants at Day 1.
k) K+ and eGFR values obtained from local laboratory will be used for finerenone up-/down-titration as well as monitoring after down/up-titration and restart.
l) Refer to sampling handling sheets or laboratory manual for sample handling.
m) At Visits 3 and 6, trough (i.e. pre-dose) samples for the determination of finerenone and empagliflozin plasma concentrations will be drawn before intake of study intervention. At this visit, study intervention will be administered at the study site and the exact time of study intervention intake on the day before the visit and on the day of the visit and the exact sampling time will be recorded in the electronic case report form. At Visits 4 and 5 (and ED, if applicable), post-dose blood samples for the determination of finerenone and empagliflozin plasma concentrations will be drawn during the visit, 1.5-10 hours after study intervention intake at home. Note: At all mentioned visits, samples should be taken even if the study interventions were not taken as indicated. In such cases, particular attention will be paid to properly document the actual time of study intervention intake before sampling. These samples will not be analyzed directly but may be analyzed in case of specific questions from the DMC, Steering Committee, or sponsor.
n) First study intervention at study site. The participant from Part A will remain 4 to 6 hours at the study site for office blood pressure monitoring and will then be equipped for 24 hours with an ambulatory blood pressure monitoring device.
o) Accountability only
p) Subsequent to an up-titration or restart of study drug after interruption of finerenone intake for more than 7 days, the investigator should perform an unscheduled visit, 4 weeks (±7 days) after titration or restart, in order to monitor K+ levels and eGFR.

6.2. Appendix 2: List of Abbreviations

**Abbreviation**	**Definition**
AE	Adverse event
AESI	Adverse event of special interest
ABPM	Ambulatory blood pressure monitoring
ACEi	Angiotensin-converting enzyme inhibitor
AKI	Acute kidney injury
ARB	Angiotensin receptor blocker
ALP	Alkaline phosphatase
ALT (SGPT)	Alanine aminotransferase serum glutamic-pyruvic transaminase
AST (SGOT)	Aspartate aminotransferase serum glutamic –oxaloacetic transaminase
ATC	Anatomical Therapeutic Chemical
BMI	body mass index
BP	Blood pressure
BUN	Blood urea nitrogen
CI	confidence interval
CKD	Chronic kidney disease
CKD-EPI	Chronic Kidney Disease Epidemiology Collaboration
CLIPS	Clinical Pharmacology Standards
CO2	Carbon dioxide
COVID-19	Coronavirus Disease 2019
CRF	Case report form
CSR	Clinical study report
CV	Cardiovascular
CVD	Cardiovascular disease
DBP	Diastolic blood pressure
DMC	Data monitoring committee
DRE	Disease-related events
ECG	Electrocardiogram
ED	Early discontinuation
eGFR	Estimated glomerular filtration rate
EOS	End of study
FAS	Full Analysis Set
FSH	Follicle stimulating hormone
GGT	Gamma-glutamyl transferase
HbA1c	Glycated hemoglobin
HDL-C	High-density lipoprotein cholesterol
HF	Heart failure
IB	Investigator’s brochure
ICH	International Council on Harmonization
IRB/IEC	Institutional review board/independent ethics committees
IWRS	Interactive web response system
K+	Potassium
KDIGO	Kidney Disease: Improving Global Outcomes
LDH	Lactate dehydrogenase
LDL-C	Low-density lipoprotein cholesterol
LLOQ	Lower limit of quantification
LOS	Listing only set
LS	Least square
MAR	Missing at random
MedDRA	Medical Dictionary for Regulatory Activities
MCH	Mean corpuscular hemoglobin
MCHC	Mean corpuscular hemoglobin concentration
MCV	Mean corpuscular volume
MI	Myocardial infarction
MMRM	Mixed model repeated measures
MRA	Mineralocorticoid receptor antagonist
OD	Once daily

PD	Pharmacodynamics
pH	Potential of hydrogen
PK	Pharmacokinetics
PPS	Per-protocol analysis set
PT	Preferred Term
RBC	Red blood cell
SAE	Serious adverse event
SAF	Safety analysis set
SAP	Statistical Analysis Plan
SAS	Statistical analysis software
SBP	Systolic blood pressure
SC	Steering Committee
SD	Standard deviation
SGLT2i	Sodium/glucose cotransporter-2 inhibitor(s)
SMQs	standardized MedDRA queries
SOC	System organ class
T2D	Type 2 Diabetes
TEAE	Treatment emergent adverse event
UACR	urinary albumin-to-creatinine ratio
ULOQ	Upper limit of quantification
US	United States of America
WBC	White blood cell
WHO-DD	World Health Organization drug dictionary

6.3. Appendix 3: GCP Violation of SMO at site 20003 in Japan

PDF
EN_Memo_Meeting with MHLW on SMC

7. References

1. Levey AS, Stevens LA, Schmid CH, Zhang YL, Castro AF, 3rd, Feldman HI, et al. A new equation to estimate glomerular filtration rate. Ann Intern Med. 2009;150(9):604-12.
2. Koopmans, L. H., Owen, D. B., Rosenblatt, J. I. (1964): Confidence intervals for the coefficient of variation for the normal and log normal distributions. Biometrika. 51: 25–32.
